# Isatuximab, carfilzomib, lenalidomide and dexamethasone in newly diagnosed multiple myeloma: a randomized phase 3 trial

**DOI:** 10.1038/s41591-026-04282-0

**Published:** 2026-04-06

**Authors:** Francesca Gay, Wilfried Roeloffzen, Meletios A. Dimopoulos, Laura Rosiñol, Marjolein van der Klift, Roberto Mina, Albert Oriol, Eirini Katodritou, Ka Lung Wu, Paula Rodríguez Otero, Roman Hájek, Elisabetta Antonioli, Mark van Duin, Mattia D’Agostino, Joaquín Martínez-López, Elena M. van Leeuwen-Segarceanu, Elena Zamagni, Niels W. C. J. van de Donk, Katja C. Weisel, Luděk Pour, Jakub Radocha, Angelo Belotti, Fredrik Schjesvold, Joan Bladé, Hermann Einsele, Pieter Sonneveld, Mario Boccadoro, Annemiek Broijl

**Affiliations:** 1 Division of Hematology, AOU Città della Salute e della Scienza di Torino, University of Torino and Department of Molecular Biotechnology and Health Sciences, University of Torino, Turin, Italy; 2 Department of Hematology, University Medical Center Groningen, Groningen, the Netherlands; 3 Department of Clinical Therapeutics, National and Kapodistrian University of Athens School of Medicine, Athens, Greece; 4 Department of Medicine, Korea University, Seoul, South Korea; 5 Amyloidosis and Myeloma Unit, Department of Hematology, Hospital Clínic de Barcelona, IDIBAPS, and PETHEMA/GEM, Barcelona, Spain; 6 Department of Internal Medicine, Amphia Hospital, Breda, the Netherlands; 7 Institut Català d’Oncologia and Institut Josep Carreras, Hospital Germans Trias i Pujol, Badalona, Spain; 8 Department of Hematology, Theagenion Cancer Hospital, Thessaloniki, Greece; 9 ZAS Cadix, Antwerp, Belgium; 10 Cancer Center Clínica Universidad de Navarra, Pamplona, Spain; 11 Department of Haematooncology, University Hospital Ostrava, Ostrava, Czech Republic; 12 Department of Haematooncology, Faculty of Medicine, University of Ostrava, Ostrava, Czech Republic; 13 Hematology Unit, AOU Careggi, Florence, Italy; 14 Department of Hematology, Erasmus MC Cancer Institute, Rotterdam, the Netherlands; 15 Hematology Department, Hospital Universitario de Octubre, Medicine Department Complutense University, imas12, CNIO, Madrid, Spain; 16 Department of Hematology, St. Antonius Hospital, Nieuwegein, the Netherlands; 17 IRCCS Azienda Ospedaliero-Universitaria di Bologna, Istituto di Ematologia ‘Seràgnoli’, Bologna, Italy; 18 Department of Medical and Surgical Sciences, Bologna University School of Medicine, Bologna, Italy; 19 Amsterdam UMC, Vrije Universiteit Amsterdam, Department of Hematology, Cancer Center Amsterdam, Amsterdam, the Netherlands; 20 Department of Oncology, Hematology and BMT, University Medical Center of Hamburg-Eppendorf, Hamburg, Germany; 21 Department of Internal Medicine, Hematology and Oncology, University Hospital Brno, Brno, Czech Republic; 22 4th Department of Internal Medicine-Hematology, University Hospital Hradec Kralove, Charles University, Faculty of Medicine in Hradec Kralove, Hradec Kralove, Czech Republic; 23 Department of Hematology, ASST Spedali Civili di Brescia, Brescia, Italy; 24 Oslo Myeloma Center, Department of Hematology, Oslo University Hospital, Oslo, Norway; 25 KG Jebsen Center for B cell malignancies, University of Oslo, Oslo, Norway; 26 Department of Internal Medicine II, University Hospital Würzburg, Würzburg, Germany; 27 European Myeloma Network (EMN), Rotterdam, the Netherlands; 28 European Myeloma Network (EMN), Turin, Italy

**Keywords:** Myeloma, Myeloma

## Abstract

Induction and consolidation with a quadruplet therapy of a CD38-targeting monoclonal antibody, a proteasome inhibitor, an immunomodulatory drug and dexamethasone are a standard-of-care treatment in transplant-eligible (TE) patients with newly diagnosed multiple myeloma (NDMM) with the optimal drugs to be used still under debate. The ongoing, phase 3 EMN24 IsKia trial randomized 302 TE patients with NDMM aged ≤70 years 1:1 to isatuximab–carfilzomib–lenalidomide–dexamethasone (Isa-KRd) versus KRd pretransplant induction and post-transplant consolidation. The primary endpoint was the rate of measurable residual disease (MRD) negativity (sensitivity of 10^−5^ or better) by next-generation sequencing (NGS) after consolidation. Key secondary endpoints were the rates of NGS-MRD negativity after induction and progression-free survival (PFS). MRD negativity rates at higher sensitivity (10^−6^ or better) were exploratory. Post-consolidation MRD negativity was significantly higher with Isa-KRd versus KRd at the 10^−5^ (77% versus 67%; odds ratio (OR) 1.67, *P* = 0.049) and 10^−6^ (68% versus 48%; OR 2.36, *P* = 0.0004) sensitivities. Deep MRD responses were rapid (post-induction Isa-KRd versus KRd: 10^−5^ 46% versus 27%, OR 2.32, *P* = 0.0007; 10^−6^ 28% versus 14%, OR 2.44, *P* = 0.0029) and durable (1-year sustained 10^−6^ MRD negativity 52% versus 38%, OR 1.82, *P* = 0.012). At current follow-up, PFS data were immature. Grade 3–4 non-hematologic adverse events (AEs), treatment discontinuations and deaths due to AEs were similar in the two arms. Isa-KRd significantly improved NGS-MRD negativity in TE patients with NDMM, with a manageable safety profile. ClinicalTrials.gov registration: NCT04483739.

## Abstract (web summary)

In the phase 3 EMN24 IsKia trial, transplant-eligible patients with newly diagnosed multiple myeloma who received isatuximab with carfilzomib, lenalidomide and dexamethasone pretransplant induction and post-transplant consolidation showed higher rates of measurable residual disease negativity after consolidation than patients who received carfilzomib, lenalidomide and dexamethasone.

## Main

In patients with newly diagnosed multiple myeloma (NDMM) eligible for high-dose melphalan and autologous stem-cell transplantation (ASCT), standard-of-care induction and consolidation therapies include a proteasome inhibitor (bortezomib), an immunomodulatory drug (thalidomide or lenalidomide) and dexamethasone, plus a CD38-targeting monoclonal antibody (mAb; daratumumab or isatuximab)^1–7^. Carfilzomib is a second-generation proteasome inhibitor currently approved for the treatment of relapsed/refractory multiple myeloma (RRMM) that showed superiority to bortezomib in the relapse setting and lower rates of peripheral neuropathy (which occurs in up to 54% of patients treated with bortezomib-based regimens)^8^. On the basis of its efficacy in the relapse setting, carfilzomib has been investigated in several studies involving transplant-eligible (TE) patients with NDMM. In the phase 2 UNITO-MM-01/FORTE trial, carfilzomib–lenalidomide–dexamethasone (KRd) plus ASCT resulted in high rates of pre-maintenance measurable residual disease (MRD) negativity (sensitivity of 10^−5^, 62%) and median progression-free survival (PFS; 99 months)^9,10^, with encouraging results also in patients with high-risk cytogenetic abnormalities (HRCA)^11^, thus suggesting that carfilzomib is an even more effective alternative to bortezomib, also for patients with NDMM. Isatuximab (Isa), a chimeric IgG mAb directed against CD38 with several anti-MM effects (including antibody-dependent cellular-mediated cytotoxicity, antibody-dependent cellular phagocytosis, complement-dependent cytotoxicity and direct induction of apoptosis), is currently approved in combination with bortezomib–lenalidomide–dexamethasone (Isa-VRd) for the treatment of TE and transplant-ineligible patients with NDMM^6,12^ and in combination with dexamethasone and either carfilzomib or pomalidomide (Isa-Kd, Isa-Pd) for the treatment of patients with RRMM^13–16^. Recently, the Isa-KRd combination showed high efficacy as induction and consolidation regimen in the phase 3 MIDAS trial and in the phase 2 GMMG-CONCEPT trial for high-risk patients^17–19^.

While modern regimens including an anti-CD38 mAb and ASCT may induce a complete response (CR) in up to 88% of patients, the conventional response assessment has proven to be suboptimal for survival prediction^20^. In this context, MRD status has emerged as a potential surrogate for PFS and overall survival (OS)^21,22^ and has been incorporated as an endpoint into a growing number of clinical trials. Furthermore, on 12 April 2024, the U.S. Food and Drug Administration’s Oncologic Drugs Advisory Committee voted in favor of MRD testing as an early endpoint in MM clinical trials^23^.

We present the efficacy and safety results of the phase 3 EMN24 IsKia trial, which compared Isa-KRd versus KRd as pre-ASCT induction and post-ASCT consolidation treatment of TE patients with NDMM. At the time the EMN24 IsKia trial was designed, the choice to include the KRd combination in the control arm was based on its high efficacy profile in phase 2 studies, which supported its recommendation in the National Comprehensive Cancer Network guidelines^9,10,24,25^. In addition, upfront quadruplet combinations including anti-CD38 mAbs plus bortezomib, immunomodulatory drugs and steroids had not yet been approved. This trial aims to show the superiority of Isa-KRd versus KRd to support the use of a new quadruplet option for the treatment of patients with NDMM, in the absence of randomized trials evaluating quadruplets that combine KRd with anti-CD38 mAbs, as only single-arm studies have been initiated.

## Results

### Patients and treatment

**Fig. 1:**
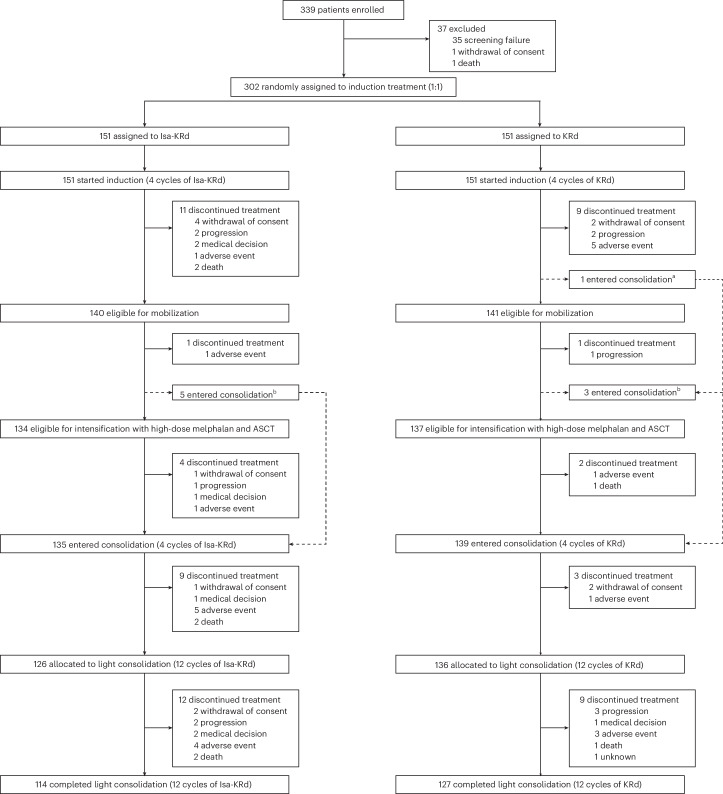
Trial profile. ^a^In the KRd arm, one patient proceeded from induction to consolidation without mobilization and intensification due to patient’s preference. ^b^Eight patients proceeded from mobilization to consolidation without intensification: 5 in the Isa-KRd arm (4 due to mobilization failure and 1 due to the patient’s preference) and 3 in the KRd arm (2 due to mobilization failure and 1 due to the patient’s preference).

A total of 302 TE patients with NDMM were enrolled and randomized between 7 October 2020 and 15 November 2021 (Isa-KRd, *n* = 151; KRd, *n* = 151). Baseline demographics and disease characteristics were well balanced between the two arms (Table 1). Figure 1 shows reasons for discontinuation across different time points. A similar proportion of patients in the two arms entered the ASCT and post-ASCT full-dose consolidation phases. Nine patients discontinued therapy during post-ASCT full-dose consolidation in the Isa-KRd arm (5 due to adverse events (AEs) and 2 due to death) versus 3 in the KRd arm (1 due to AEs). In total, 126 versus 136 patients started light consolidation in the Isa-KRd versus KRd arms. Twelve patients discontinued treatment during light consolidation in the Isa-KRd arm (4 due to AEs and 2 due to death) versus 9 in the KRd arm (3 due to AEs and 1 due to death; see the ‘Safety’ section for details on the causes of discontinuation and death). Patients who completed light consolidation were 114 (75%) versus 127 (84%) in the Isa-KRd versus KRd arms. All patients are currently either receiving maintenance treatment or are in the follow-up phase if observation was selected or if maintenance treatment was discontinued for any reason. The median duration of follow-up was 48 months (interquartile range (IQR) 45–51) from randomization.

**Table 1:** Patient characteristics

	Number (%) of patients^a^	
	Isa-KRd (*n* = 151)	KRd (*n* = 151)
Age (years) Median (IQR)	61 (55–66)	60 (54–63)
Sex Female Male	72 (48) 79 (52)	67 (44) 84 (56)
ECOG performance status^b^ 0 1 2	88 (58) 51 (34) 12 (8)	93 (62) 51 (34) 7 (5)
t(11;14) No Yes Missing	108 (79) 29 (21) 14	111 (81) 26 (19) 14
Cytogenetic risk as per IMWG Standard risk High risk Missing	115 (82) 25 (18) 11	113 (81) 26 (19) 12
Risk as per modified IMS/IMWG criteria Standard risk High risk Missing	107 (76) 34 (24) 10	106 (75) 35 (25) 10
Cytogenetic risk: 0 versus 1 versus 2+ HRCA 0 HRCA 1 HRCA 2+ HRCA Missing	78 (56) 49 (35) 13 (9) 11	75 (54) 49 (35) 15 (11) 12
R-ISS I II III Missing	48 (34) 85 (59) 10 (7) 8	50 (35) 82 (58) 10 (7) 9
R2-ISS I II III IV Missing	34 (24) 45 (32) 52 (37) 8 (6) 12	35 (25) 47 (34) 51 (37) 6 (4) 12

ECOG, Eastern Cooperative Oncology Group; t, translocation; R-ISS, Revised International Staging System stage; R2-ISS, Second Revision of the International Staging System stage.

^a^Unless otherwise indicated. Percentages were calculated on patients with available data.

^b^A score of 0 indicates fully active, able to carry on all pre-disease performance without restriction; a score of 1 indicates restricted in physically strenuous activity but ambulatory and able to carry out work of a light or sedentary nature (for example, light housework or office work).

### Efficacy

Responses were rapid, with a median time to partial response (PR) of 1 cycle in both arms and with 83% (95% confidence interval (CI) 76%–88%) versus 75% (95% CI 68%–82%) of patients in the Isa-KRd versus KRd arms achieving at least a very good (VG) PR after induction. Moreover, clinical responses improved over time (Extended Data Table 1).

MRD compliance throughout the study was excellent, with 96%–100% of samples evaluable at the 10^−5^ and 10^−6^ sensitivity cut-offs by next-generation sequencing (NGS; Extended Data Table 2).

Regarding the primary endpoint, by intention-to-treat (ITT) analysis, the 10^−5^ MRD negativity rate after post-ASCT full-dose consolidation was significantly higher in the Isa-KRd arm versus the KRd arm (116 (77%) versus 101 (67%) patients, odds ratio (OR) 1.67, 95% CI 1.00–2.80, *P* = 0.049). Importantly, an exploratory analysis at a sensitivity of 10^−6^ showed that the 10^−6^ MRD negativity rate was also significantly higher in the Isa-KRd arm (102 (68%) versus 72 (48%) patients; OR 2.36, 95% CI 1.47–3.79, *P* = 0.0004).

Regarding the first key secondary endpoint, by ITT analysis, the post-induction 10^−5^ MRD negativity rate was statistically significantly higher in the Isa-KRd arm versus the KRd arm (69 (46%) versus 41 (27%) patients, OR 2.32, 95% CI 1.43–3.78, *P* = 0.0007). Similarly, the exploratory analysis at the sensitivity of 10^−6^ showed that the MRD negativity rate was statistically significantly higher in the Isa-KRd arm versus the KRd arm (42 (28%) versus 21 (14%) patients, OR 2.44, 95% CI 1.36–4.40, *P* = 0.0029). The MRD negativity rate improved over time. After transplant, 97 (64%) patients treated with Isa-KRd were MRD negative (10^−5^ cut-off) versus 75 (50%) treated with KRd (OR 1.88, 95% CI 1.18–3.00, *P* = 0.0083). Similarly, the 10^−6^ MRD negativity rate was significantly higher in the Isa-KRd arm (79 (52%) patients) versus the KRd arm (41 (27%); OR 3.01, 95% CI 1.86–4.89, *P* < 0.0001). The rate of MRD negativity further improved during the full-dose-consolidation and light-consolidation phases, particularly with regard to the 10^−6^ MRD negativity rate: 111 (74%) patients were MRD negative with Isa-KRd versus 96 (64%) with KRd (OR 1.63, 95% CI 0.99–2.67, *P* = 0.055) at the end of the light consolidation by ITT analysis. This led to a significantly higher rate of 1-year sustained 10^−6^ MRD negativity by ITT analysis with Isa-KRd versus KRd (52% versus 38%, OR 1.82, 95% CI 1.14–2.91, *P* = 0.012) at the current follow-up (Table 2).

**Table 2:** Rates of MRD negativity by NGS according to treatment phase (ITT analysis) and rates of 1-year sustained MRD negativity

	Post induction		Post ASCT		Post-ASCT full-dose consolidation		Post light consolidation		1-year sustained MRD negativity	
	Isa-KRd (*n* = 151)	KRd (*n* = 151)	Isa-KRd (*n* = 151)	KRd (*n* = 151)	Isa-KRd (*n* = 151)	KRd (*n* = 151)	Isa-KRd (*n* = 151)	KRd (*n* = 151)	Isa-KRd (*n* = 151)	KRd (*n* = 151)
**10**^−5^ **MRD (95% CI)**	46% (38%–54%)	27% (20%–35%)	64% (56%–72%)	50% (41%–58%)	77% (69%–83%)	67% (59%–74%)	79% (71%–85%)	74% (66%–81%)	66% (57%–73%)	59% (51%–67%)
**10**^−**6**^ **MRD (95% CI)**	28% (21%–36%)	14% (9%–20%)	52% (44%–60%)	27% (20%–35%)	68% (59%–75%)	48% (40%–56%)	74% (66%–80%)	64% (55%–71%)	52% (44%–60%)	38% (31%–47%)

**Fig. 2:**
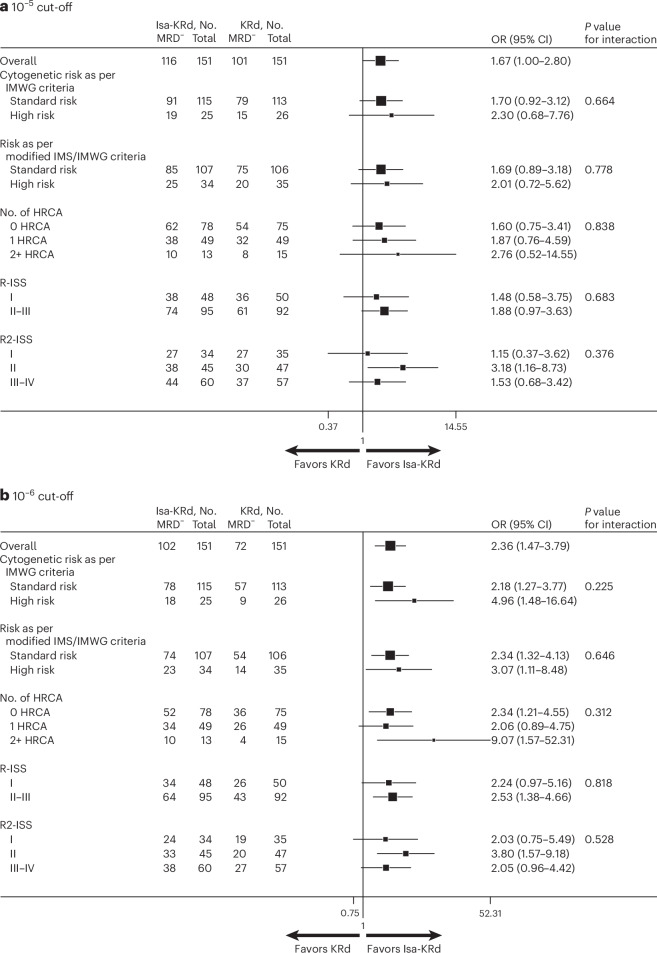
Subgroup analysis of post-ASCT full-dose consolidation MRD negativity rates (assessed by NGS). **a**, 10^−5^ cut-off. **b**, 10^−6^ cut-off. Squares represent the odds ratios for Isa-KRd versus KRd overall and in subgroup variables from a logistic model adjusted for stratification factors. The square size is proportional to the subgroup size. Error bars correspond to 95% CIs. Two-sided Wald test *P* values for interaction between arms and subgroup variables are reported. No multiple-comparison adjustment was applied. 10^−5^, NGS at the sensitivity of 10^−5^; 10^−6^, NGS at the sensitivity of 10^−6^; MRD^−^, measurable residual disease-negative; No., number.

Subgroup analyses of MRD negativity rates were performed. At the post-ASCT full-dose consolidation time point, a consistent benefit in terms of MRD negativity rates, at both the 10^−5^ and 10^−6^ cut-offs, was observed in all patient subgroups, including high-risk and ultra-high-risk patients (Fig. 2). Moreover, in the Isa-KRd arm, the MRD negativity rate was similar in patients with no HRCA (10^−5^: 79%; 10^−6^: 67%), 1 HRCA (10^−5^: 78%; 10^−6^: 69%) and 2+ HRCA (10^−5^: 77%; 10^−6^: 77%). In the KRd arm, patients with 1 HRCA (10^−5^: 65%; 10^−6^: 53%), and especially those with 2+ HRCA (10^−5^: 53%; 10^−6^: 27%), showed inferior rates of MRD negativity, compared with standard-risk patients (10^−5^: 72%; 10^−6^: 48%; Extended Data Table 3). Similarly, in the Isa-KRd arm, the MRD negativity rate was comparable in patients with standard risk (10^−5^: 79%; 10^−6^: 69%) and high risk (10^−5^: 74%; 10^−6^: 68%) as per modified International Myeloma Society/International Myeloma Working Group (IMS/IMWG) criteria^26^. In the KRd arm, patients with high risk as per modified IMS/IMWG criteria (10^−5^: 57%; 10^−6^: 40%) showed inferior rates of MRD negativity, compared with standard-risk patients (10^−5^: 71%; 10^−6^: 51%; Extended Data Table 3).

**Extended Data Fig. 1:**
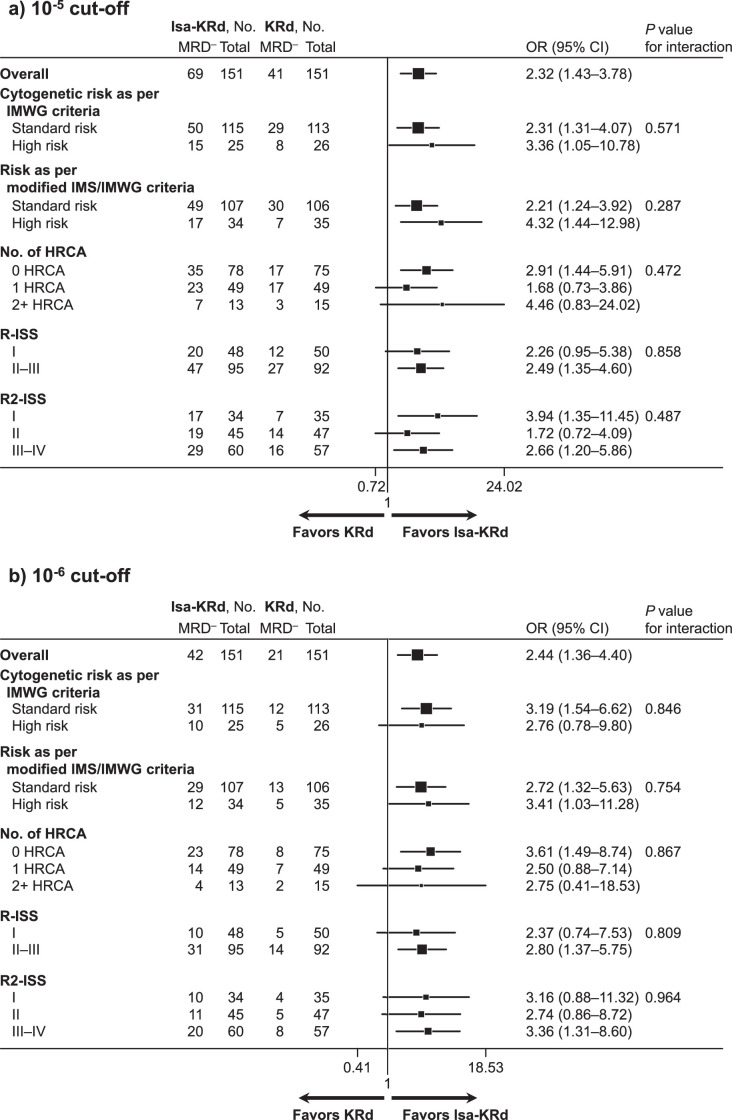
Subgroup analysis of post-induction MRD negativity rates (assessed by NGS). **a**, 10^−5^ cut-off. **b**, 10^−6^ cut-off. *Squares represent the odds ratios for Isa-KRd vs. KRd overall and in subgroup variables from a logistic model adjusted for stratification factors. The square size is proportional to the subgroup size. Error bars correspond to 95% CIs. Two-sided Wald test P for interaction between arms and subgroup variables are reported. No multiple comparison adjustment was applied.* **Abbreviations**. MRD, measurable disease negativity; NGS next-generation sequencing; Isa, isatuximab; K, carfilzomib; R, lenalidomide; d, dexamethasone; No., number; MRD^−^, measurable residual disease-negative; OR, odds ratio; CI, confidence interval; *P*, p-value; IMWG, International Myeloma Working Group; IMS, International Myeloma Society; HRCA, high-risk cytogenetic abnormalities; R-ISS, Revised International Staging System stage; R2-ISS, Second Revision of the International Staging System stage.

**Extended Data Fig. 2:**
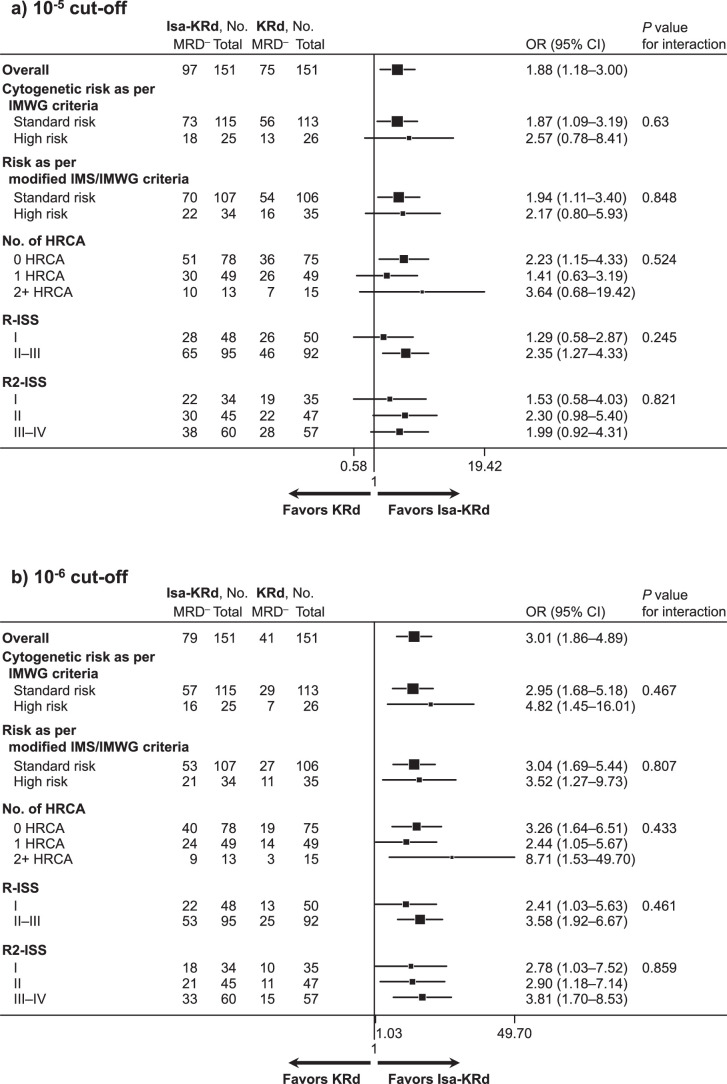
Subgroup analysis of post-ASCT MRD negativity rates (assessed by NGS). **a**, 10^−5^ cut-off. **b**, 10^−6^ cut-off. *Squares represent the odds ratios for Isa-KRd vs. KRd overall and in subgroup variables from a logistic model adjusted for stratification factors. The square size is proportional to the subgroup size. Error bars correspond to 95% CIs. Two-sided Wald test P for interaction between arms and subgroup variables are reported. No multiple comparison adjustment was applied.* **Abbreviations**. ASCT, autologous stem-cell transplantation; MRD, measurable disease negativity; NGS next-generation sequencing; Isa, isatuximab; K, carfilzomib; R, lenalidomide; d, dexamethasone; No., number; MRD^−^, measurable residual disease-negative; OR, odds ratio; CI, confidence interval; *P*, p-value; IMWG, International Myeloma Working Group; IMS, International Myeloma Society; HRCA, high-risk cytogenetic abnormalities; R-ISS, Revised International Staging System stage; R2-ISS, Second Revision of the International Staging System stage.

A consistent benefit in terms of MRD negativity rates, at both the 10^−5^ and 10^−6^ cut-offs, was observed in all patient subgroups also at the post-induction (Extended Data Fig. 1) and post-ASCT (Extended Data Fig. 2) time points.

**Fig. 3:**
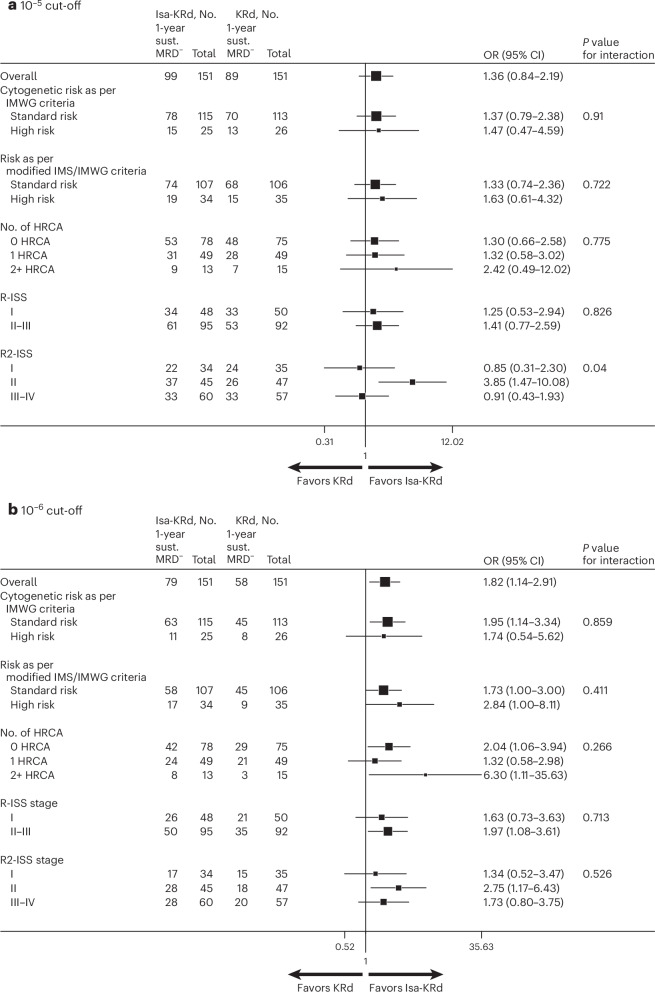
Subgroup analysis of 1-year sustained MRD negativity rates (assessed by NGS). **a**, 10^−5^ cut-off. **b**, 10^−6^ cut-off. Squares represent the odds ratios for Isa-KRd versus KRd overall and in subgroup variables from a logistic model adjusted for stratification factors. The square size is proportional to the subgroup size. Error bars correspond to 95% CIs. Two-sided Wald test *P* values for interaction between arms and subgroup variables are reported. No multiple-comparison adjustment was applied. sust., sustained.

The advantage in terms of 10^−6^ 1-year sustained MRD negativity with Isa-KRd was retained in all subgroups (Fig. 3b). In particular, in ultra-high-risk patients with 2+ HRCA, the rates of 1-year sustained MRD negativity (sensitivity of 10^−6^) were 62% with Isa-KRd versus 20% with KRd (OR 6.30, 95% CI 1.11–35.66; Extended Data Table 4 and Fig. 3). In patients with IMS/IMWG high-risk features, the rates of sustained 10^−6^ MRD negativity were 50% with Isa-KRd versus 26% with KRd (OR 2.84, 95% CI 1.00–8.11; Extended Data Table 4 and Fig. 3). Of interest, the rates of sustained 10^−6^ MRD negativity in high-risk, ultra-high-risk and standard-risk patients treated with Isa-KRd were similar.

A post hoc analysis on patients with t(11;14) showed lower rates of MRD negativity compared with patients without t(11;14). This difference was observed at each time point, including light consolidation, and was more pronounced at the sensitivity of 10^−6^ (Extended Data Table 5a). A trend toward higher MRD negativity rates in the Isa-KRd arm versus the KRd arm was consistently detected also in t(11;14)-positive patients (Extended Data Table 5b).

At the current follow-up, the number of events (progressive disease (PD)/death) was 58, and the 4-year PFS was 80% across both arms. The current number of events does not yet allow for an evaluation of the PFS comparison (the second key secondary endpoint of the trial) according to the trial Redacted Statistical Analysis Plan (included in Supplementary Information).

### Safety

The most common grade 1–2, 3 and 4 AEs related to the study drugs are summarized in Table 3. Toxicities that occurred during standard-of-care treatment with mobilization and melphalan at 200 mg m^−^^2^ plus ASCT (MEL200-ASCT) are not reported, except when they led to protocol discontinuation. During induction and consolidation, the most common grade 3–4 AEs were neutropenia (51 (34%) of 151 patients in the Isa-KRd arm versus 27/151 (18%) in the KRd arm), thrombocytopenia (10/151 (7%) with Isa-KRd versus 12/151 (8%) with KRd) and vascular toxicities, including thrombosis and hypertension (7/151 (5%) with Isa-KRd versus 15/151 (10%) with KRd). Of note, grade 4 AEs were uncommon. Treatment-related serious AEs (SAEs) occurring during the induction and consolidation phases were reported in 34/151 (23%) patients in the Isa-KRd arm versus 31/151 (21%) in the KRd arm. The most common treatment-related SAEs were thromboembolic events (2/151 (1%) patients in the Isa-KRd arm versus 9/151 (6%) in the KRd arm) and respiratory tract infections (2/151 (1%) in the Isa-KRd arm versus 4/151 (3%) in the KRd arm).

**Table 3:** Treatment-emergent adverse events related to study drugs

	Induction and consolidation No. (%) of patients						Light consolidation No. (%) of patients					
	Isa-KRd (*n* = 151)			KRd (*n* = 151)			Isa-KRd (*n* = 126)			KRd (*n* = 136)		
	Grades 1–2	Grade 3	Grade 4	Grades 1–2	Grade 3	Grade 4	Grades 1–2	Grade 3	Grade 4	Grades 1–2	Grade 3	Grade 4
**Hematologic** Anemia Neutropenia Thrombocytopenia	27 (18) 9 (6) 34 (23)	3 (2) 41 (27) 7 (5)	1 (1) 10 (7) 3 (2)	19 (13) 7 (5) 16 (11)	3 (2) 22 (15) 9 (6)	0 5 (3) 3 (2)	8 (6) 11 (9) 10 (8)	0 21 (17) 2 (2)	0 1 (1) 1 (1)	8 (6) 9 (7) 15 (11)	0 20 (15) 2 (1)	0 3 (2) 2 (1)
**Non-hematologic** Gastrointestinal disorders Diarrhea Nausea and vomiting	30 (20) 20 (13)	3 (2) 1 (1)	0 0	21 (14) 15 (10)	2 (1) 0	0 0	26 (21) 3 (2)	2 (2) 1 (1)	0 0	20 (15) 12 (9)	3 (2) 1 (1)	0 0
Infusion-related reactions	27 (18)	5 (3)	0	2 (1)	0	0	1 (1)	0	0	0	0	0
General disorders and administration site conditions Asthenia and fatigue Fever	26 (17) 18 (12)	5 (3) 1 (1)	0 0	35 (23) 21 (14)	4 (3) 1 (1)	0 0	16 (13) 6 (5)	1 (1) 0	0 0	16 (12) 4 (3)	0 0	0 0
Infections and infestations COVID-19 infections^a^ Infections of other type Respiratory tract infection	26 (17) 19 (13) 10 (7)	4 (3) 2 (1) 4 (3)	1 (1) 1 (1) 0	21 (14) 19 (13) 8 (5)	3 (2) 1 (1) 4 (3)	1 (1) 0 0	25 (20) 16 (13) 28 (22)	2 (2) 6 (5) 4 (3)	0 1 (1) 0	33 (24) 13 (10) 14 (10)	2 (1) 2 (1) 5 (4)	0 0 0
Skin and subcutaneous tissue disorders Rash	23 (15)	5 (3)	0	26 (17)	3 (2)	0	2 (2)	0	0	4 (3)	0	0
Metabolism and nutritional disorders	20 (13)	5 (3)	1 (1)	15 (10)	4 (3)	0	5 (4)	1 (1)	1 (1)	4 (3)	0	0
Vascular disorders	19 (13)	7 (5)	0	17 (11)	14 (9)	1 (1)	10 (8)	5 (4)	0	16 (12)	2 (1)	0
Psychiatric disorders	19 (13)	1 (1)	1 (1)	22 (15)	3 (2)	0	9 (7)	1 (1)	0	9 (7)	1 (1)	0
Nervous system disorders Peripheral sensory neuropathy	17 (11)	0	0	16 (11)	0	0	12 (10)	0	0	13 (10)	0	0
Respiratory, thoracic and mediastinal disorders Dyspnea	17 (11)	1 (1)	0	7 (5)	1 (1)	0	3 (2)	1 (1)	0	5 (4)	0	0
Hepatobiliary disorders Liver function test increase	19 (13)	6 (4)	0	8 (5)	3 (2)	0	1 (1)	0	0	2 (1)	1 (1)	0
Musculoskeletal and connective tissue disorders Muscle spasms	8 (5)	0	0	15 (10)	0	0	6 (5)	0	0	6 (4)	1 (1)	0

Only treatment-emergent AEs related to study drugs and reported in at least 10% of patients are included. AEs that occurred during the ASCT phase and are considered as part of the standard-of-care treatment are not included.

^a^All COVID-19 infections are reported regardless of the investigator’s attribution to study treatment.

During light consolidation, the most common grade 3–4 AEs were neutropenia (22/126 (17%) patients in the Isa-KRd arm versus 23/136 (17%) in the KRd arm), infections (12/126 (10%) in the Isa-KRd arm versus 9/136 (7%) in the KRd arm) and gastrointestinal toxicities (5/126 (4%) in the Isa-KRd arm versus 7/136 (5%) in the KRd arm). SAEs occurring during the light-consolidation phase were reported in 15/126 (12%) patients in the Isa-KRd arm versus 16/136 (12%) in the KRd arm. The most common treatment-related SAEs observed during the light-consolidation phase were infections (13/126 (10%) patients in the Isa-KRd arm versus 11/136 (8%) in the KRd arm).

In the Isa-KRd arm, 2 patients were diagnosed with endometrial cancer during induction, 1 with melanoma and 1 with basal cell carcinoma during mobilization, and 1 with myelodysplastic syndrome and 1 with basal cell carcinoma during the follow-up and maintenance phase. In the KRd arm, 1 patient was diagnosed with myelodysplastic syndrome during the light-consolidation phase, and 1 developed a melanoma during the maintenance and follow-up phase.

At least 1 dose reduction of any study drug occurred in 77/151 (51%) patients in the Isa-KRd arm versus 54/151 (36%) in the KRd arm during the induction and consolidation phases and in 35/126 (28%) patients in the Isa-KRd arm versus 40/136 (29%) in the KRd arm during the light-consolidation phase (details are reported in Supplementary Results and Extended Data Table 6).

Rates of treatment discontinuation due to an AE were similar in the two arms: 12 (8%) patients in the Isa-KRd arm versus 10 (7%) in the KRd arm (Extended Data Table 7). During the induction and consolidation phases, 5 patients died: 1 due to PD (in the Isa-KRd arm) and 4 due to infections (3 in the Isa-KRd arm versus 1 in the KRd arm). During the light-consolidation phase, 3 patients died: 1 due to pulmonary embolism and 1 due to ischemic cerebrovascular infarction in the Isa-KRd arm and 1 due to cardiac arrest in the KRd arm.

### Stem-cell mobilization

The proportion of patients proceeding to ASCT was similar in the two arms (134 (89%) in the Isa-KRd arm versus 137 (91%) in the KRd arm). Six patients (4 in the Isa-KRd arm versus 2 in the KRd arm) did not receive ASCT due to mobilization failure.

The median number of CD34^+^ stem cells collected was 5.0 × 10^6^ cells kg^−1^ (IQR 3.7 × 10^6^–6.6 × 10^6^) in the Isa-KRd arm versus 5.5 × 10^6^ cells kg^−1^ (IQR 3.8 × 10^6^–7.8 × 10^6^) in the KRd arm (*P* = 0.14). Among patients undergoing stem-cell collection, 54/140 (39%) in the Isa-KRd arm versus 41/141 (29%) in the KRd arm received plerixafor (*P* = 0.10).

The median number of CD34^+^ transplanted cells was 3.1 × 10^6^ cells kg^−1^ (IQR 2.4 × 10^6^–4.0 × 10^6^) in the Isa-KRd arm versus 3.3 × 10^6^ cells kg^−1^ (IQR 2.7 × 10^6^–4.4 × 10^6^) in the KRd arm (*P* = 0.10).

Details about the median number of aphereses and hematopoietic reconstitution are reported in Supplementary Results.

## Discussion

To the best of our knowledge, this is the first randomized trial to investigate Isa-KRd versus KRd for the upfront treatment of TE patients with NDMM. The 4-drug regimen Isa-KRd significantly increased the rate of MRD negativity by NGS, compared with KRd: in the ITT analysis, 77% of patients in the Isa-KRd arm versus 67% in the KRd arm achieved MRD negativity after post-ASCT full-dose consolidation at the sensitivity of 10^−5^, thus allowing the trial to meet its primary endpoint. Of note, most patients achieved an even deeper response, with 68% being MRD negative at the sensitivity of 10^−6^ (48% in the KRd arm). Although the analysis at the 10^−6^ cut-off was not prespecified in the trial, we performed it, as it is well known that MRD negativity at the sensitivity of 10^−6^ is a strong prognostic biomarker of PFS and OS^27,28^. Moreover, international recommendations state that, in MM trials, MRD assays must have a limit of detection of ≤10^−5^, need to be applicable to >90% of patients and, possibly, need to rely on good analytical performance and sample quality; MRD at a sensitivity <10^−6^ should also be reported^29,30^. Indeed, in the IsKia trial, we showed that not only 10^−5^ but also 10^−6^ MRD negativity was applicable to >90% of patients. In addition, our data are of interest as they suggest that, in the context of highly effective regimens, the 10^−6^ cut-off is more informative than the 10^−5^ cut-off: at the post-ASCT full-dose consolidation time point, the advantage of Isa-KRd versus KRd was less remarkable at the 10^−5^ cut-off (77% versus 67%) while it was substantially better at the 10^−6^ cut-off (68% versus 48%). In line with these findings, light consolidation with Isa-KRd, compared with KRd, was observed to be a prolonged and effective treatment. It was associated with similar rates of 10^−5^ post-light-consolidation MRD negativity and 1-year sustained MRD negativity in both arms, but with significantly different rates when the 10^−6^ cut-off was applied (1-year sustained 10^−6^ MRD negativity 52% versus 38%). The evaluation of the 10^−6^ threshold is important in view of future trials comparing highly effective therapies.

MRD responses were rapid, with a significant benefit associated with Isa-KRd versus KRd already after 4 induction cycles and an almost double rate of 10^−5^ and 10^−6^ MRD negativity in Isa-KRd patients. Responses improved with the sequential approach comprising ASCT and post-ASCT full-dose consolidation. Although cross-trial comparisons are limited by differences in terms of trial design, methodology and inclusion criteria, our findings are in line with those from other phase 2 studies exploring anti-CD38 mAbs plus carfilzomib and lenalidomide^18,31^ and with those from phase 2–3 studies demonstrating the efficacy of anti-CD38 mAbs plus bortezomib and immunomodulatory drugs^2,3,5,32^. Prolonged light consolidation further improved the depth of response in terms of 10^−6^ MRD negativity. To the best of our knowledge, no other trial has implemented a similar approach (except for single-arm studies focusing on high-risk patients)^18,33^.

The benefit observed with the Isa-KRd quadruplet was maintained in all subgroups of patients, including those with high-risk disease, and especially those with ultra-high-risk disease (such as patients with 2+ HRCA and patients at high risk according to the recent IMS/IMWG classification)^26^. Subgroup analyses should be interpreted with caution owing to the small number of patients in high-risk groups. Moreover, subgroup analyses involving updated risk classification methods (such as the IMS/IMWG classification) were exploratory. Nevertheless, two major points are of interest. First, high-quality response rates were already evident after induction treatment and improved in all groups with the sequential approach. Second, the rates of 10^−6^ and 10^−5^ MRD negativity in high-risk and standard-risk patients were similar in those treated with Isa-KRd, whereas they were different in those treated with KRd (lower MRD negativity and 1-year sustained MRD negativity rates were observed when adopting the 10^−6^ cut-off, particularly in ultra-high-risk patients), thus suggesting that the addition of isatuximab to KRd was able to induce and maintain high-quality responses with prolonged treatment, even in ultra-high-risk patients.

MRD negativity is the most important dynamic predictor of outcome, whose strong prognostic role has been consistently reported in many trials. In high-risk disease, achieving and, particularly, maintaining MRD negativity is the only factor that has thus far proven capable of mitigating, if not yet overcoming, the adverse prognostic impact of baseline features^34,35^. The high rate of MRD negativity achieved with Isa-KRd in all subgroups (including high-risk and ultra-high-risk patients), the rapidity of response and the high rate of 1-year sustained MRD negativity suggest the potential role of Isa-KRd in high-risk patients with NDMM, who currently represent an unmet medical need.

Nevertheless, MRD kinetics may change in different biological groups. Indeed, in the subgroup of patients with t(11;14), who are generally considered at standard risk, a progressive improvement in terms of MRD negativity was observed with sequential treatment, but lower MRD negativity rates were observed at each time point compared with patients without t(11;14), thus suggesting a peculiar kinetics and biology of MRD clearance in this subgroup. Although this finding should be considered with caution, as it is based on a post hoc analysis on a limited number of patients, it is in line with results from the MIDAS trial in this specific subset^19^.

Due to the current Statistical Analysis Plan (Supplementary Information), the current follow-up of our trial does not yet allow for the evaluation of data on sustained MRD negativity beyond 1 year and for a comparison of PFS between the two arms. Although this limitation must be acknowledged, it should be noted that MRD negativity was selected as the primary endpoint in this trial, in line with recent ongoing studies^19,36,37^, given the particularly favorable outcomes observed in the control arm. This is likely to result in a very long follow-up being required before differences in long-term outcomes can be detected. More specifically, PFS results are expected to occur 71 months from the date of the last randomization.

Treatment was well tolerated, with similar rates of non-hematologic grade 3–4 toxicities in the two arms. The addition of isatuximab to KRd did not increase the rates of cardiac and vascular toxicities. The rates of infections were also similar in the two arms, despite the higher rates of neutropenia during induction in patients receiving Isa-KRd (in line with other reports on quadruplets in the upfront treatment)^2,32^. In the IsKia trial, although enrollment started during the COVID-19 pandemic, most of the COVID-19 infections were of grade 1–2. Infection prophylaxis and COVID-19 vaccination, once available, were strongly recommended, according to standard international guidelines^38–40^. Furthermore, the participating centers received periodic newsletters highlighting any recent recommendations based on the new evidence. The low rate of severe infections may also be partly explained by the trial population (young patients eligible for ASCT) and the high rate of response already observed after induction (it is well known that infections are more common in intermediate-fit and elderly patients, and in those with suboptimal response to treatment). Overall, treatment discontinuation due to toxicity was reported in 12 patients in the Isa-KRd and 10 patients in the KRd arm. During induction, ASCT and post-ASCT full-dose consolidation, treatment-related deaths (3 patients in the Isa-KRd arm and 1 in the KRd arm) were caused by infections (despite the low overall rate in the trial), which unfortunately are still one of the main causes of morbidity and mortality in MM^2,32^. Three patients (2 in the Isa-KRd arm and 1 in the KRd arm) died from cardiovascular events during light consolidation. However, treatment with isatuximab did not increase the overall rate of cardiovascular toxicity, which remained low in both arms.

In conclusion, pre-ASCT induction and post-ASCT consolidation with Isa-KRd, compared with KRd, provided rapid, clinically meaningful and high-quality responses in TE patients with NDMM. We believe that our data, including those regarding the control arm with KRd, can support the potential role of carfilzomib in the upfront setting, given the high efficacy in terms of MRD negativity. Currently, in the upfront therapy, there is only one standard option for TE patients that is supported by guidelines in the European Union and United States (anti-CD38 mAb—either daratumumab or isatuximab—plus bortezomib–lenalidomide–dexamethasone). The combination of KRd plus an anti-CD38 mAb is not approved in Europe and is recommended as ‘other recommended therapy’ by the National Comprehensive Cancer Network guidelines^41^. In the absence of a direct randomized comparison between VRd plus an anti-CD38 mAb versus KRd plus an anti-CD38 mAb, data supporting the safety and efficacy of this new combination upfront are needed. Myeloma is a very heterogeneous disease, and it has still to be determined whether all patients should receive the same therapy or different patients may benefit from different agents. In our trial, the KRd-based combination was well tolerated in the TE population and showed very low rates of neuropathy compared with bortezomib-based approaches. Isatuximab was administered intravenously; however, current data^42^ support the equivalence of the subcutaneous administration, which reduces the infusion burden for patients and may further improve treatment compliance and tolerability in the future. The favorable tolerability observed in this trial enabled a prolonged multiagent treatment approach, resulting in notable rates of 1-year sustained MRD negativity, retained in high-risk and ultra-high-risk patients, who currently represent an unmet medical need. More specifically, in this high- and ultra-high-risk setting, trials have demonstrated the importance of maintaining adequate treatment intensity to ensure effective disease control^33,34,43,44^. In the rapidly evolving treatment landscape of MM, novel regimens based on KRd plus an anti-CD38 mAb backbone upfront may represent valuable alternatives, given their different toxicity profiles, the efficacy observed both overall and in high-risk and ultra-high-risk subgroups, as well as their potential to offer different treatment plans, with or without prolonged multiagent therapy.

## Methods

### Study design and participants

The EMN24 IsKia trial is a randomized, open-label, phase 3 trial that enrolled patients from 42 European centers in 8 countries (Supplementary Information).

TE patients with symptomatic NDMM aged 18–70 years with measurable disease defined according to standard criteria^45^ were eligible. Other inclusion criteria were Eastern Cooperative Oncology Group (ECOG) Performance Status ≤2, life expectancy >3 months, absolute neutrophil count ≥1 × 10^9^ l^−1^, platelet count ≥75 × 10^9^ l^−1^, left ventricular ejection fraction ≥40% and creatinine clearance ≥30 ml min^−1^. Exclusion criteria included other malignancies within the last 3 years, peripheral neuropathy of grade >2 or grade 2 with pain, unstable angina or myocardial infarction within 4 months before randomization, New York Heart Association functional class III or IV heart failure, uncontrolled angina, uncontrolled hypertension, pulmonary embolism within the last 5 years, history of severe coronary artery disease, severe uncontrolled ventricular arrhythmias, sick sinus syndrome or electrocardiographic evidence of acute ischemia or grade 3 conduction system abnormalities unless the patient had a pacemaker, and any notable clinical condition that placed the patient at a substantial risk if they participated in the trial. A complete list of inclusion and exclusion criteria is reported in the Redacted Trial Protocol (included in Supplementary Information). Information about patient sex was requested at registration and was self-reported; the provided options were ‘male’ or ‘female’. Patients did not receive compensation for their participation in the trial.

### Inclusion and ethics

The EMN24 IsKia trial is sponsored by the European Myeloma Network (EMN), which, in collaboration with the investigators, designed and conducted the trial, including data collection, management, analysis, interpretation of the data, preparation of the paper, decision to submit the paper for publication and approval of the paper. Sanofi and Amgen provided the funding to conduct the trial, with no role in the design and conduct of the study; collection, management, analysis and interpretation of the data; preparation, review or approval of the paper; and decision to submit the paper for publication.

All authors contributed equally to the acquisition, analysis or interpretation of data for this work. All authors critically reviewed the work for important intellectual content, approved the final version to be published and agreed to be accountable for all aspects of the work in ensuring that questions related to the accuracy or integrity of any part of the work are appropriately investigated and resolved. All authors had access to all the data reported in the study and had final responsibility for the decision to submit this paper for publication.

This trial and its Protocol and amendments were approved by the ethics or institutional review boards at each of the participating centers (ethics committee of the coordinating center: Comitato etico territoriale Lombardia 6, Italy). All patients gave written informed consent before participating in the trial, which was conducted in accordance with the Declaration of Helsinki and Good Clinical Practice guidelines.

### Randomization and masking

At enrollment, a computer system randomly assigned patients (1:1) to treatment into one of the two induction and consolidation arms. Patients were stratified according to International Staging System (ISS)^46^ stage (I versus II versus III) and cytogenetic risk assessed by fluorescence in situ hybridization (high risk (presence of t(4;14) and/or t(14;16) and/or del(17p)) versus standard risk or missing (none of these abnormalities)) and then randomized using a web-based, computer-generated procedure completely concealed from study participants. Randomization was performed according to a randomization list, which was created by statisticians, using dynamic sizes of blocks (from 2 to 6) for each stratum.

### Procedures

**Extended Data Fig. 3:**
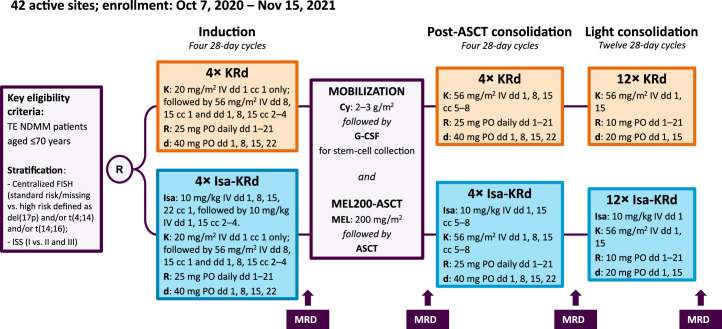
Trial design and MRD time points. **Abbreviations**. MRD, measurable residual disease; TE, transplant-eligible; NDMM, newly diagnosed multiple myeloma; FISH, fluorescence *in situ* hybridization; del, deletion; t, translocation; ISS, International Staging System stage; R, randomization; Isa, isatuximab; K, carfilzomib; R, lenalidomide; d, dexamethasone; IV, intravenous; dd, days; cc, cycles; PO, orally; Cy, cyclophosphamide; G-CSF, granulocyte colony-stimulating factor; MEL, melphalan; ASCT, autologous stem-cell transplantation.

Patients in the KRd arm received 4 28-day induction cycles with KRd (carfilzomib 20 mg m^−2^ intravenously administered (IV) on day 1 of cycle 1, followed by 56 mg m^−2^ IV on days 8 and 15 of cycle 1, then 56 mg m^−2^ IV on days 1, 8 and 15 for all subsequent doses; lenalidomide 25 mg orally administered (PO) on days 1–21; dexamethasone 40 mg on days 1, 8, 15 and 22) followed by stem-cell mobilization with cyclophosphamide (2,000–3,000 mg m^−^^2^ IV on day 1) plus granulocyte colony-stimulating factor (10 µg kg^−1^ subcutaneously administered) from day 5 until stem-cell collection was completed. Thereafter, patients received intensification with melphalan at 200 mg m^−2^ (MEL200) and ASCT and, between days 90 and 120, started full-dose consolidation with 4 KRd cycles (same dose and schedule used during the induction phase). After full-dose consolidation, patients started the ‘light-consolidation phase’: 12 28-day, light-intensity KRd cycles with carfilzomib 56 mg m^−2^ IV on days 1 and 15; lenalidomide 10 mg PO on days 1–21; and dexamethasone 20 mg on days 1 and 15. Patients in the Isa-KRd arm received the same treatment administered in the KRd arm plus isatuximab 10 mg m^−2^ on days 1, 8, 15 and 22 of the first induction cycle; on days 1 and 15 of the induction cycles 2–4 and full-dose consolidation cycles 1–4; and on day 1 of each light-consolidation cycle (Extended Data Fig. 3).

After the end of light consolidation, patients were recommended to receive maintenance treatment with lenalidomide alone as per standard of care.

Details on dose reductions and interruptions and other trial procedures are detailed in the Redacted Trial Protocol (Supplementary Information).

### Outcomes

The primary study endpoint was the rate of MRD negativity (sensitivity of 10^−5^ or better) detected by NGS in the ITT population after post-ASCT full-dose consolidation.

Key secondary endpoints were the rates of NGS-MRD negativity after induction (sensitivity of 10^−5^ or better) and PFS in the ITT population. Other secondary endpoints included overall response rate, MRD negativity after ASCT, MRD negativity after light consolidation, rate of 1-year sustained MRD negativity, safety and comparative analyses in patient subgroups defined according to known prognostic factors. In particular, the analyses focused on high-risk cytogenetics per IMWG criteria (defined as the presence of t(4;14), t(14;16) or del(17p))^47^; number of HRCA (1 HRCA was defined as the presence of one of the following HRCA: del(17p13.1), t(4;14) (p16.3;q32.3), t(14;16) (q32.3;q23), gain(1q21) or amp(1q21); 2+ HRCA was defined as the presence of at least two HRCA); Revised ISS (R-ISS; R-ISS stage I included ISS stage I (serum β2-microglobulin level <3.5 mg l^−^^1^ and serum albumin level ≥3.5 g dl^−^^1^), no HRCA (del(17p) and/or t(4;14) and/or t(14;16)) and normal lactate dehydrogenase (LDH) level (less than the upper limit of normal range); R-ISS stage III included ISS stage III (serum β2-microglobulin level >5.5 mg l^−1^) and HRCA or high LDH level; and R-ISS stage II included all the other possible combinations)^48^; and Second Revision of the ISS (R2-ISS; in which a value was assigned to each risk feature according to their impact on OS (ISS III 1.5, ISS II 1, del(17p) 1, high LDH 1, t(4;14) 1 and 1q + 0.5 points), and patients were stratified into four risk groups according to the total additive score: low (R2-ISS-I, 0 points), low-intermediate (II, 0.5–1 points), intermediate-high (III, 1.5–2.5 points) and high (IV, 3–5 points))^49^. In addition, we aimed to analyze outcomes according to the recent IMS/IMWG Consensus Recommendations^26^. As data on t(14;20), TP53 mutations and differentiation between monoallelic versus biallelic del(1p32) were not available for the current analyses, we defined high risk as per modified IMS/IMWG Consensus Recommendations^26^ as the presence of at least one of these abnormalities: (1) del(17p) with a cut-off of >20% clonal fraction; (2) an IgH translocation including t(4;14) or t(14;16) along with 1q+ and/or del(1p32); (3) del(1p32) along with 1q+; and (4) β2 microglobulin ≥5.5 mg l^−^^1^ with normal creatinine (<1.2 mg dl^−1^). This analysis was exploratory and not prespecified in the trial Protocol, as this risk stratification was very recently published. A post hoc analysis, focusing on patients with t(11;14) as subgroup of interest, was also performed. A disaggregated analysis by sex was not prespecified in the trial Protocol and was not performed because sex is not thought to be a major confounder in the MM field.

Additional planned secondary endpoints not reported in this contribution were time to progression, time to next treatment, PFS 2, OS, total duration of MRD negativity assessed by NGS, duration of response, patient-reported outcomes, maintenance details, subsequent lines of therapy, agreement between MRD techniques (see the Redacted Statistical Analysis Plan in Supplementary Information).

All efficacy analyses were based on the ITT principle: all patients eligible to receive treatment and randomly assigned to one of the treatment arms were included.

### Definition and evaluation of the endpoints

The response rate was defined according to the International Uniform Response Criteria^50^. Response was evaluated after each treatment cycle. MRD evaluation was centralized in the laboratories of the Torino (Italy) and Rotterdam (the Netherlands) sites. The NGS and post-NGS phases were performed by Adaptive Biotechnologies (Seattle, US-WA), as previously reported^51,52^. MRD was tested by NGS and next-generation flow after each treatment phase (Extended Data Fig. 3) in all patients achieving at least a very good partial response (≥VGPR)^27,53,54^.

We aimed to analyze different MRD cut-offs (even though this was not clearly prespecified). Nonetheless, the primary endpoint was based on the prespecified 10^−5^ cut-off only, while the 10^−6^ cut-off was exploratory. NGS-MRD was defined as negative if the disease was confidently detected (equal to or above the limit of detection) but the number of malignant cells per million nucleated cells (PerMillionCount) was below the threshold (10 out of 1,000,000 cells for the analysis at 10^−5^ sensitivity, 1 out of 1,000,000 for the analysis at 10^−6^ sensitivity) or if the disease was not detected (below the limit of detection of the technique) and a sufficient number of cells was assayed to detect MRD at the threshold level (limit of quantification below the threshold). In any other cases, the MRD status was defined as positive. In the ITT analysis, patients were classified as MRD positive if they had only MRD-positive test results; patients missing a MRD evaluation or not achieving a VGPR or better were also considered MRD positive. Single imputation was applied by considering missing data as MRD positive, so that no multiple imputation was used for addressing the presence of missing data. One-year sustained MRD was defined as 2 consecutive MRD-negative evaluations at least 12 months apart. PFS was defined as the time from randomization to the first of either PD or death, whichever came first. OS was defined as the time from randomization to death.

Data from all patients who received at least one dose of any study drug were included in the safety analyses. Treatment-emergent AEs were monitored throughout the study period, graded according to the National Cancer Institute Common Terminology Criteria for Adverse Events (NCI-CTCAE, version 5.0)^55^ and summarized by the worst NCI-CTCAE grade. AEs leading to death or treatment discontinuation, grade 3–4 AEs and SAEs were summarized separately.

### Statistical analysis

The calculation of the sample size for the primary endpoint was done with the following assumptions, considering the ITT population: *α* = 0.05 (two-sided), *β* = 0.10 and post-ASCT full-dose consolidation MRD negativity (sensitivity of 10^−5^, by NGS) rate: Isa-KRd 64% versus KRd 45%. The total number of patients required was 300 (by the *χ*^2^ test with Yates’ continuity correction).

The power of 85% (*β* = 0.15) for the first key secondary endpoint (post-induction MRD negativity rate by NGS) was evaluated with the *χ*^2^ test with Yates’ continuity correction, with the following assumptions, considering the ITT population: *α* = 0.05 (two-sided) and post-induction MRD negativity (sensitivity of 10^−5^; by NGS) rate: Isa-KRd 30% versus KRd 15%. The power of 92% (*β* = 0.08) for the second key secondary endpoint (PFS) was evaluated with the Schoenfeld formula, with the following assumptions, considering the ITT population: *α* = 0.05 (two-sided) and 48-month PFS: Isa-KRd 83% versus KRd: 69% (HR 0.50). According to the latest protocol amendment (see the Redacted Trial Protocol in Supplementary Information), the achievement of 90% of power requires 91 PFS events, which are expected to occur 71 months after the date of the last randomization.

A hierarchical testing procedure was used for the primary and key secondary endpoints to achieve control of the overall familywise type I error rate at a two-sided significance level of 0.05.

Four interim safety analyses were planned upon the completion of each treatment phase for the first 75 patients: the first analysis after induction, the second after mobilization, the third after full-dose consolidation and the fourth after the first 4 cycles of light consolidation. An Independent Data Monitoring Committee reviewed the data and confirmed that the trial could proceed without changes. As safety was not the principal aim of this trial, no statistical correction was made for the sample size or the *α* error of the first four interim analyses. According to the latest protocol amendment (see the Redacted Trial Protocol in Supplementary Information), a fifth interim analysis of PFS is planned upon reaching 68 events. Any amendment involving PFS was made without unblinded access to arm-specific PFS data.

Logistic regression models were used to estimate ORs and their 95% CIs and *P* values for the analyses of binary endpoints, response criteria (as defined by the IMWG)^50^ and MRD rates. All analyses were adjusted according to stratification factors. Clopper–Pearson exact CIs were adopted for computing 95% CIs for rates. Between-group differences in AEs were evaluated using Fisher’s exact tests. We reported two-sided *P* values. Statistical analyses were done with R (v4.2.1).

The data cut-off was 10 September 2025. This trial is registered with ClinicalTrials.gov (NCT04483739). Study recruitment is complete.

### Reporting summary

Further information on research design is available in the Nature Portfolio Reporting Summary linked to this article.

## Online content

Any methods, additional references, Nature Portfolio reporting summaries, source data, extended data, supplementary information, acknowledgements, peer review information; details of author contributions and competing interests; and statements of data and code availability are available at 10.1038/s41591-026-04282-0.

## Supplementary information

Supplementary Information Supplementary results, list of study sites, Redacted Trial Protocol and Redacted Statistical Analysis Plan.

Reporting Summary

## Figures and Tables

**Extended Data Table 1:**
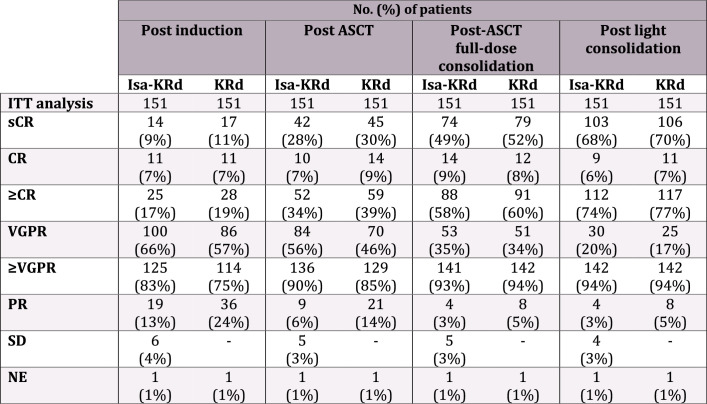
Best response according to treatment phase

**Abbreviations**. No., number; ASCT, autologous stem-cell transplantation; Isa, isatuximab; K, carfilzomib; R, lenalidomide; d, dexamethasone; ITT, intention to treat; sCR, stringent complete response; CR, complete response; VGPR, very good partial response; PR, partial response; SD, stable disease; NE, not evaluable.

**Extended Data Table 2:**
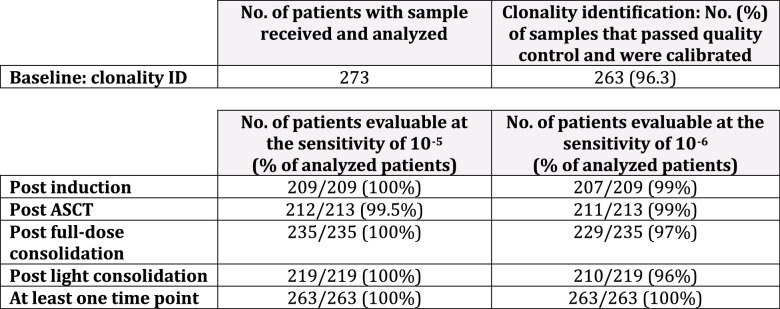
MRD negativity by NGS in terms of compliance and sample quality

**Abbreviations**. MRD, measurable residual disease; NGS, next-generation sequencing; ASCT, autologous stem-cell transplantation.

**Extended Data Table 3:**
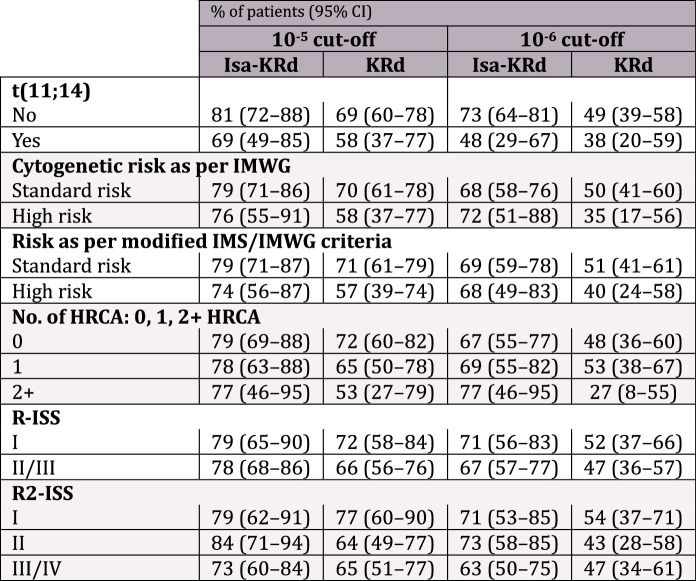
Post-ASCT full-dose consolidation rates of MRD negativity by NGS in subgroups of interest

**Abbreviations**. ASCT, autologous stem-cell transplantation; MRD, measurable residual disease; NGS, next-generation sequencing; CI, confidence interval; 10^−5^, NGS at the sensitivity of 10^−5^; 10^−6^, NGS at the sensitivity of 10^−6^; Isa, isatuximab; K, carfilzomib; R, lenalidomide; d, dexamethasone; t, translocation; IMWG, International Myeloma Working Group; IMS, International Myeloma Society; No., number; HRCA, high-risk cytogenetic abnormalities; R-ISS, Revised International Staging System stage; R2-ISS, Second Revision of the International Staging System stage.

**Extended Data Table 4:**
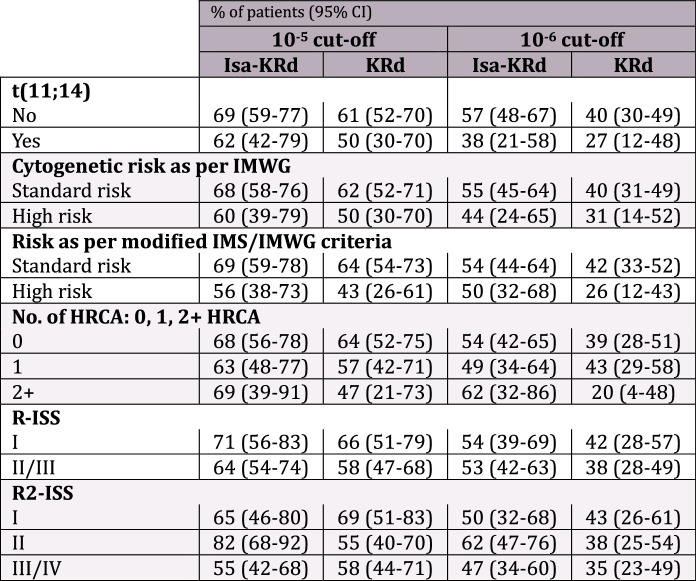
1-year sustained MRD negativity by NGS in subgroups of interest

**Abbreviations**. MRD, measurable residual disease; NGS, next-generation sequencing; CI, confidence interval; 10^−5^, NGS at the sensitivity of 10^−5^; 10^−6^, NGS at the sensitivity of 10^−6^; Isa, isatuximab; K, carfilzomib; R, lenalidomide; d, dexamethasone; t, translocation; IMWG, International Myeloma Working Group; IMS, International Myeloma Society; No., number; HRCA, high-risk cytogenetic abnormalities; R-ISS, Revised International Staging System stage; R2-ISS, Second Revision of the International Staging System stage.

**Extended Data Table 5:**
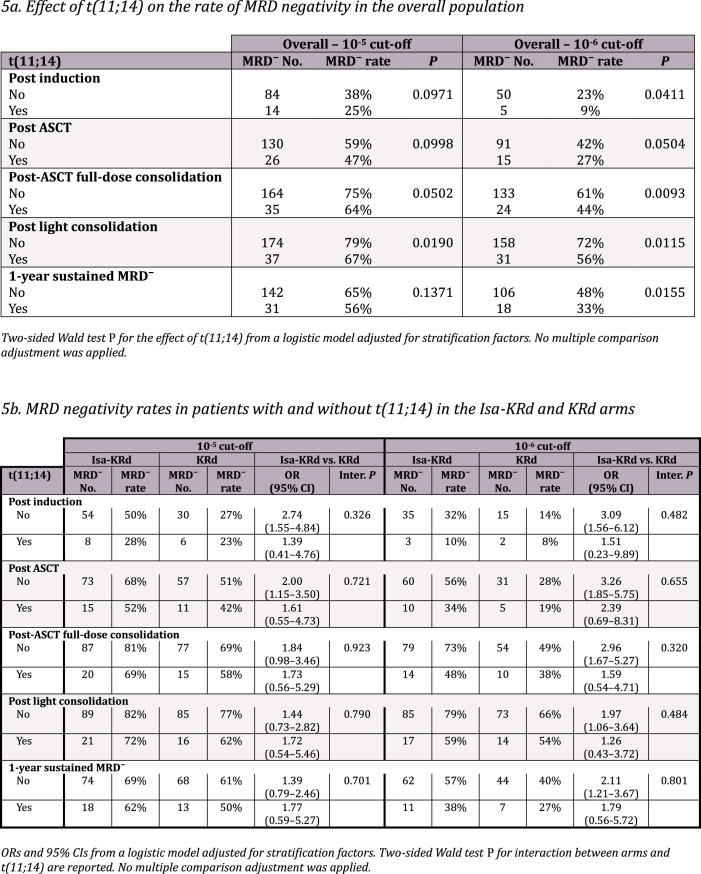
Effect of t(11;14) on the rates of MRD negativity

**a.** Effect of t(11;14) on the rate of MRD negativity in the overall population

*Two-sided Wald test P for the effect of t(11;14) from a logistic model adjusted for stratification factors. No multiple comparison adjustment was applied*.

**b.** MRD negativity rates in patients with and without t(11;14) in the Isa-KRd and KRd arms

*ORs and 95% CIs from a logistic model adjusted for stratification factors. Two-sided Wald test* P *for interaction between arms and t(11;14) are reported. No multiple comparison adjustment was applied.*

**Abbreviations**. t, translocation; MRD, measurable residual disease; 10^−5^, NGS at the sensitivity of 10^−5^; 10^−6^, NGS at the sensitivity of 10^−6^; MRD⁻, measurable residual disease negativity; *P*, p-value; ASCT, autologous stem-cell transplantation; Isa, isatuximab; K, carfilzomib; R, lenalidomide; d, dexamethasone; MRD⁻, measurable residual disease negativity; OR, odds ratio; CI, confidence interval; Inter., interaction.

**Extended Data Table 6:**
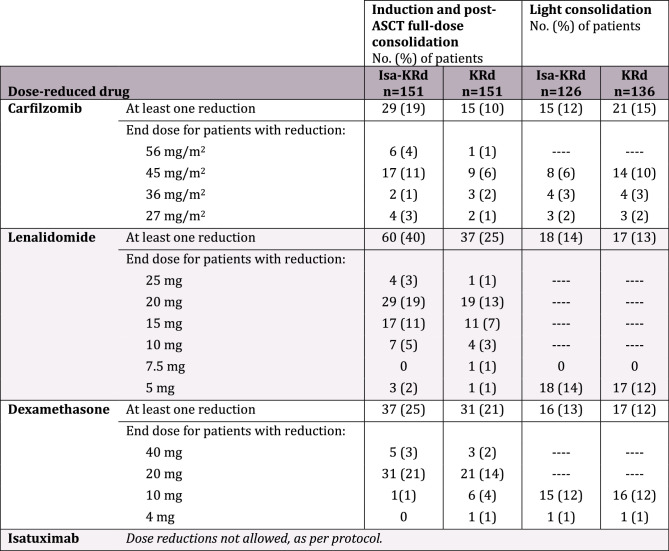
Dose reductions

**Abbreviations**. ASCT, autologous stem-cell transplantation; No., number; Isa, isatuximab; K, carfilzomib; R, lenalidomide; d, dexamethasone.

**Extended Data Table 7:**
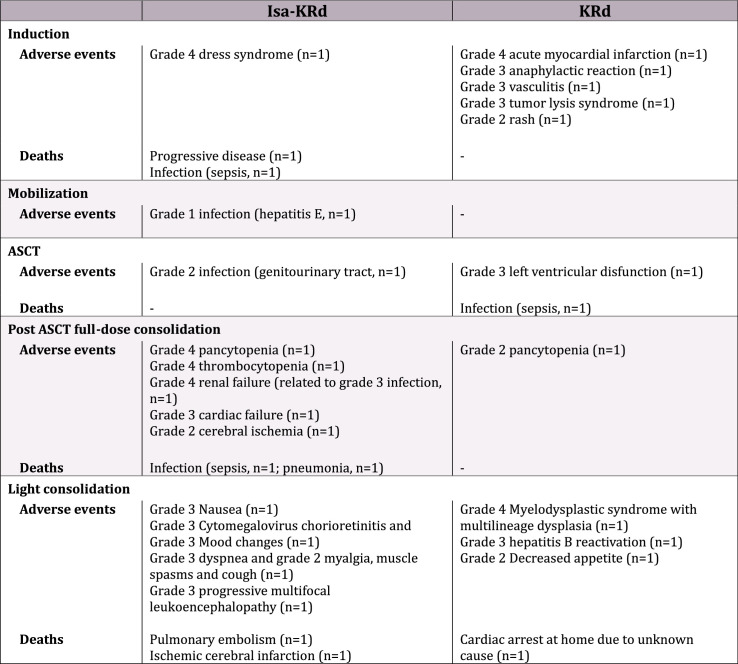
Reasons for discontinuation according to treatment arm

**Abbreviations**. Isa, isatuximab; K, carfilzomib; R, lenalidomide; d, dexamethasone; ASCT, autologous stem-cell transplantation.

## Data Availability

Data supporting this article are part of an ongoing clinical trial and are consequently not publicly available. After the publication of this article, deidentified data collected for this analysis and related documents will be made available upon reasonably justified request, which needs to be written and addressed to the attention of the sponsor of the EMN24 IsKia trial, the European Myeloma Network (EMN), at the following e-mail address: info@emn.life. The EMN is responsible for evaluating and eventually accepting or refusing requests to disclose data and their related documents, in compliance with the ethical approval conditions and with applicable laws and regulations, and in conformance with the agreements in place with the involved subjects, the participating institutions and all the other parties directly or indirectly involved in the participation, conduct, development, management and evaluation of this analysis. Response will typically be given in 3 months. The Redacted Trial Protocol and Statistical Analysis Plan can be found in Supplementary Information.

## Code availability

The EMN24 IsKia trial was conducted using the Electronic Case Report Form (eCRF). Study data were collected and managed using Research Electronic Data Capture (REDCap, developed by the REDCap Consortium)^56,57^ electronic data capture tools. All statistical analyses were performed using R software (v4.2.1).

## References

[1] Dimopoulos, M. A. et al. Multiple myeloma: EHA-ESMO Clinical Practice Guidelines for diagnosis, treatment and follow-up. *Ann. Oncol.* **32**, 309–322 (2021). 33549387 10.1016/j.annonc.2020.11.014

[2] Sonneveld, P. et al. Daratumumab, bortezomib, lenalidomide, and dexamethasone for multiple myeloma. *N. Engl. J. Med.* **390**, 301–313 (2024). 38084760 10.1056/NEJMoa2312054

[3] Moreau, P. et al. Maintenance with daratumumab or observation following treatment with bortezomib, thalidomide, and dexamethasone with or without daratumumab and autologous stem-cell transplant in patients with newly diagnosed multiple myeloma (CASSIOPEIA): an open-label, randomised, phase 3 trial. *Lancet Oncol.* **22**, 1378–1390 (2021). 34529931 10.1016/S1470-2045(21)00428-9

[4] Moreau, P. et al. Bortezomib, thalidomide, and dexamethasone with or without daratumumab before and after autologous stem-cell transplantation for newly diagnosed multiple myeloma (CASSIOPEIA): a randomised, open-label, phase 3 study. *Lancet* **394**, 29–38 (2019). 31171419 10.1016/S0140-6736(19)31240-1

[5] Goldschmidt, H. et al. Addition of isatuximab to lenalidomide, bortezomib, and dexamethasone as induction therapy for newly diagnosed, transplantation-eligible patients with multiple myeloma (GMMG-HD7): part 1 of an open-label, multicentre, randomised, active-controlled, phase 3 trial. *Lancet Haematol.* **9**, e810–e821 (2022). 36328040 10.1016/S2352-3026(22)00263-0

[6] Mai, E. K. et al. Isatuximab, lenalidomide, bortezomib, and dexamethasone induction therapy for transplant-eligible newly diagnosed multiple myeloma: final part 1 analysis of the GMMG-HD7 trial. *J. Clin. Oncol.* **43**, 1279–1288 (2025). 39652594 10.1200/JCO-24-02266 PMC11974620

[7] Moreau, P. et al. Bortezomib, thalidomide, and dexamethasone with or without daratumumab and followed by daratumumab maintenance or observation in transplant-eligible newly diagnosed multiple myeloma: long-term follow-up of the CASSIOPEIA randomised controlled phase 3 trial. *Lancet Oncol.* **25**, 1003–1014 (2024). 38889735 10.1016/S1470-2045(24)00282-1 PMC12375812

[8] Orlowski, R. Z. et al. Carfilzomib–dexamethasone versus bortezomib-dexamethasone in relapsed or refractory multiple myeloma: updated overall survival, safety, and subgroups. *Clin. Lymphoma Myeloma Leuk.* **19**, 522–530.e1 (2019). 31160237 10.1016/j.clml.2019.04.018

[9] Gay, F. et al. Carfilzomib with cyclophosphamide and dexamethasone or lenalidomide and dexamethasone plus autologous transplantation or carfilzomib plus lenalidomide and dexamethasone, followed by maintenance with carfilzomib plus lenalidomide or lenalidomide alone for patients with newly diagnosed multiple myeloma (FORTE): a randomized, open-label, phase 2 trial. *Lancet Oncol.* **22**, 1705–1720 (2021). 34774221 10.1016/S1470-2045(21)00535-0

[10] Gay, F. et al. Carfilzomib induction, consolidation, and maintenance with or without autologous stem-cell transplant: long-term follow-up of the randomised, phase 2 FORTE trial. *Hemasphere* **9**, 220–221 abstr. S194 [EHA 2025 30th Congress] (2025).

[11] Mina, R. et al. Carfilzomib induction, consolidation, and maintenance with or without autologous stem-cell transplantation in patients with newly diagnosed multiple myeloma: pre-planned cytogenetic subgroup analysis of the randomised, phase 2 FORTE trial. *Lancet Oncol.* **24**, 64–76 (2022). 36528035 10.1016/S1470-2045(22)00693-3

[12] Facon, T. et al. Isatuximab, bortezomib, lenalidomide, and dexamethasone for multiple myeloma. *N. Engl. J. Med.* **391**, 1597–1609 (2024). 38832972 10.1056/NEJMoa2400712

[13] Deckert, J. et al. SAR650984, a novel humanized CD38-targeting antibody, demonstrates potent antitumor activity in models of multiple myeloma and other CD38^+^ hematologic malignancies. *Clin. Cancer Res.* **20**, 4574–4583 (2014). 24987056 10.1158/1078-0432.CCR-14-0695

[14] Feng, X. et al. Targeting CD38 suppresses induction and function of T regulatory cells to mitigate immunosuppression in multiple myeloma. *Clin. Cancer Res.* **23**, 4290–4300 (2017). 28249894 10.1158/1078-0432.CCR-16-3192 PMC5540790

[15] Moreau, P. et al. Isatuximab, carfilzomib, and dexamethasone in relapsed multiple myeloma (IKEMA): a multicentre, open-label, randomised phase 3 trial. *Lancet* **397**, 2361–2371 (2021). 34097854 10.1016/S0140-6736(21)00592-4

[16] Attal, M. et al. Isatuximab plus pomalidomide and low-dose dexamethasone versus pomalidomide and low-dose dexamethasone in patients with relapsed and refractory multiple myeloma (ICARIA-MM): a randomised, multicentre, open-label, phase 3 study. *Lancet* **394**, 2096–2107 (2019). 31735560 10.1016/S0140-6736(19)32556-5

[17] Perrot, A. et al. Isatuximab, carfilzomib, lenalidomide, and dexamethasone induction in newly diagnosed myeloma: analysis of the MIDAS trial. *Blood* **146**, 52–61 (2025). 39841461 10.1182/blood.2024026230 PMC12782956

[18] Leypoldt, L. B. et al. Isatuximab, carfilzomib, lenalidomide, and dexamethasone for the treatment of high-risk newly diagnosed multiple myeloma. *J. Clin. Oncol.* **42**, 26–37 (2024). 37753960 10.1200/JCO.23.01696 PMC10730063

[19] Perrot, A. et al. Measurable residual disease-guided therapy in newly diagnosed myeloma. *N. Engl. J. Med.* **393**, 425–437 (2025). 40459097 10.1056/NEJMoa2505133 PMC13292240

[20] Lahuerta, J.-J. et al. Depth of response in multiple myeloma: a pooled analysis of three PETHEMA/GEM clinical trials. *J. Clin. Oncol.* **35**, 2900–2910 (2017). 28498784 10.1200/JCO.2016.69.2517 PMC5568033

[21] Munshi, N. C. et al. A large meta-analysis establishes the role of MRD negativity in long-term survival outcomes in patients with multiple myeloma. *Blood Adv.* **4**, 5988–5999 (2020). 33284948 10.1182/bloodadvances.2020002827 PMC7724898

[22] Landgren, O. et al. EVIDENCE meta-analysis: evaluating minimal residual disease as an intermediate clinical end point for multiple myeloma. *Blood* **144**, 359–367 (2024). 38768337 10.1182/blood.2024024371 PMC11418064

[23] Center for Drug Evaluation and Research *April 12, 2024 Meeting of the Oncologic Drugs Advisory Committee (ODAC) Webcast—Part 2* (U.S. Food and Drug Administration, 2024); https://www.fda.gov/media/177651/download

[24] Jakubowiak, A. J. et al. A phase 1/2 study of carfilzomib in combination with lenalidomide and low-dose dexamethasone as a frontline treatment for multiple myeloma. *Blood* **120**, 1801–1809 (2012). 22665938 10.1182/blood-2012-04-422683 PMC5162553

[25] Jakubowiak, A. J. et al. Treatment outcome with the combination of carfilzomib, lenalidomide, and low-dose dexamethasone (CRd) for newly diagnosed multiple myeloma (NDMM) after extended follow-up. *J. Clin. Oncol.* **31**, abstr. 8543 [ASCO 2013 Annual Meeting] (2013).

[26] Avet-Loiseau, H. et al. International Myeloma Society/International Myeloma working group consensus recommendations on the definition of high-risk multiple myeloma. *J. Clin. Oncol.* **43**, 2739–2751 (2025). 40489728 10.1200/JCO-24-01893 PMC13445576

[27] Paiva, B. et al. Measurable residual disease by next-generation flow cytometry in multiple myeloma. *J. Clin. Oncol.* **38**, 784–792 (2020). 31770060 10.1200/JCO.19.01231

[28] Bahlis, N. J. et al. Daratumumab plus lenalidomide and dexamethasone in relapsed/refractory multiple myeloma: extended follow-up of POLLUX, a randomized, open-label, phase 3 study. *Leukemia* **34**, 1875–1884 (2020). 32001798 10.1038/s41375-020-0711-6 PMC7326710

[29] Costa, L. J. et al. International harmonization in performing and reporting minimal residual disease assessment in multiple myeloma trials. *Leukemia* **35**, 18–30 (2021). 10.1038/s41375-020-01012-4 32778736

[30] Perrot, A. et al. Minimal residual disease negativity using deep sequencing is a major prognostic factor in multiple myeloma. *Blood* **132**, 2456–2464 (2018). 30249784 10.1182/blood-2018-06-858613 PMC6284215

[31] Costa, L. J. et al. Daratumumab, carfilzomib, lenalidomide, and dexamethasone with minimal residual disease response-adapted therapy in newly diagnosed multiple myeloma. *J. Clin. Oncol.* **40**, 2901–2912 (2022). 34898239 10.1200/JCO.21.01935

[32] Voorhees, P. M. et al. Daratumumab, lenalidomide, bortezomib, and dexamethasone for transplant-eligible newly diagnosed multiple myeloma: the GRIFFIN trial. *Blood* **136**, 936–945 (2020). 32325490 10.1182/blood.2020005288 PMC7441167

[33] Kaiser, M. F. et al. Daratumumab, cyclophosphamide, bortezomib, lenalidomide, and dexamethasone as induction and extended consolidation improves outcome in ultra-high-risk multiple myeloma. *J. Clin. Oncol.* **41**, 3945–3955 (2023). 37315268 10.1200/JCO.22.02567

[34] D’Agostino, M. et al. Predictors of unsustained measurable residual disease negativity in patients with multiple myeloma. *Blood* **143**, 592–596 (2024). 38048557 10.1182/blood.2023022080

[35] Guerrero, C. et al. Predictors of unsustained measurable residual disease negativity in transplant-eligible patients with multiple myeloma. *Blood* **143**, 597–603 (2024). 38048552 10.1182/blood.2023022083

[36] Zamagni, E. et al. Phase 3 study of teclistamab (Tec) in combination with lenalidomide (Len) and Tec alone versus Len alone in newly diagnosed multiple myeloma (NDMM) as maintenance therapy following autologous stem cell transplantation (ASCT): safety run-in (SRI) results from the Majestec-4/EMN30 Trial. *Blood* **144**, abstr. 494 [ASH 2024 66th Meeting] (2024).

[37] Broijl, A. et al. EMAGINE/CARTITUDE-6: a randomized phase 3 study of DVRd followed by ciltacabtagene autoleucel versus DVRd followed by autologous stem cell transplant in transplant-eligible patients with newly diagnosed multiple myeloma. *Hemasphere* **7**, 22–23, abstr. P23 [EHA 2023 28th Congress] (2023).

[38] Terpos, E. et al. European Myeloma Network guidelines for the management of multiple myeloma-related complications. *Haematologica* **100**, 1254–1266 (2015). 26432383 10.3324/haematol.2014.117176 PMC4591757

[39] Terpos, E. et al. Management of patients with multiple myeloma in the era of COVID-19 pandemic: a consensus paper from the European Myeloma Network (EMN). *Leukemia* **34**, 2000–2011 (2020). 32444866 10.1038/s41375-020-0876-z PMC7244257

[40] Terpos, E. et al. Management of patients with multiple myeloma and COVID-19 in the post pandemic era: a consensus paper from the European Myeloma Network (EMN). *Leukemia* **37**, 1175–1185 (2023). 37142661 10.1038/s41375-023-01920-1 PMC10157596

[41] *NCCN Clinical Practice Guidelines in Oncology (NCCN Guidelines®). Multiple Myeloma. Version 4.2025 — November 26, 2025* (National Comprehensive Cancer Network, 2025).

[42] Ailawadhi, S. et al. Isatuximab subcutaneous by on-body injector versus isatuximab intravenous plus pomalidomide and dexamethasone in relapsed/refractory multiple myeloma: phase III IRAKLIA study. *J. Clin. Oncol.* **43**, 2527–2537 (2025). 40459178 10.1200/JCO-25-00744

[43] Costa, L. J. et al. Minimal residual disease response-adapted therapy in newly diagnosed multiple myeloma (MASTER): final report of the multicentre, single-arm, phase 2 trial. *Lancet Haematol.* **10**, e890–e901 (2023). 37776872 10.1016/S2352-3026(23)00236-3 PMC10836587

[44] Leypoldt, L. B. et al. Isatuximab, carfilzomib, lenalidomide, and dexamethasone (Isa-KRd) in front-line treatment of high-risk multiple myeloma: interim analysis of the GMMG-CONCEPT trial. *Leukemia* **36**, 885–888 (2022). 34732857 10.1038/s41375-021-01431-x PMC8885414

[45] Rajkumar, S. V. et al. International Myeloma Working Group updated criteria for the diagnosis of multiple myeloma. *Lancet Oncol.* **15**, e538–e548 (2014). 25439696 10.1016/S1470-2045(14)70442-5

[46] Greipp, P. R. et al. International staging system for multiple myeloma. *J. Clin. Oncol.* **23**, 3412–3420 (2005). 15809451 10.1200/JCO.2005.04.242

[47] Sonneveld, P. et al. Treatment of multiple myeloma with high-risk cytogenetics: a consensus of the International Myeloma Working Group. *Blood* **127**, 2955–2962 (2016). 27002115 10.1182/blood-2016-01-631200 PMC4920674

[48] Palumbo, A. et al. Revised international staging system for multiple myeloma: a report from International Myeloma Working Group. *J. Clin. Oncol.* **33**, 2863–2869 (2015). 26240224 10.1200/JCO.2015.61.2267 PMC4846284

[49] D’Agostino, M. et al. Second Revision of the International Staging System (R2-ISS) for overall survival in multiple myeloma: a European Myeloma Network (EMN) report within the HARMONY project. *J. Clin. Oncol.* **364**, 3406–3418 (2022). 10.1200/JCO.21.02614 35605179

[50] Kumar, S. et al. International Myeloma Working Group consensus criteria for response and minimal residual disease assessment in multiple myeloma. *Lancet Oncol.* **17**, e328–e346 (2016). 27511158 10.1016/S1470-2045(16)30206-6

[51] Faham, M. et al. Deep-sequencing approach for minimal residual disease detection in acute lymphoblastic leukemia. *Blood* **120**, 5173–5180 (2012). 23074282 10.1182/blood-2012-07-444042 PMC3537310

[52] Monter, A. & Nomdedéu, J. F. ClonoSEQ assay for the detection of lymphoid malignancies. *Expert Rev. Mol. Diagn.* **19**, 571–578 (2019). 31179776 10.1080/14737159.2019.1627877

[53] Flores-Montero, J. et al. Next generation flow for highly sensitive and standardized detection of minimal residual disease in multiple myeloma. *Leukemia* **31**, 2094–2103 (2017). 28104919 10.1038/leu.2017.29 PMC5629369

[54] *clonoSEQ® Assay Technical Information* (Adaptive Biotechnologies Corporation, 2021); https://ous.clonoseq.com/technical-documents/

[55] *Common Terminology Criteria for Adverse Events (CTCAE) Version 5.0* (US Dept of Health and Human Services, National Institutes of Health, National Cancer Institute, 2017); https://dctd.cancer.gov/research/ctep-trials/for-sites/adverse-events/ctcae-v5-5x7.pdf

[56] Harris, P. A. et al. Research electronic data capture (REDCap)—a metadata-driven methodology and workflow process for providing translational research informatics support. *J. Biomed. Inform.* **42**, 377–381 (2009). 18929686 10.1016/j.jbi.2008.08.010 PMC2700030

[57] Harris, P. A. et al. The REDCap consortium: building an international community of software platform partners. *J. Biomed. Inform.* **95**, 103208 (2019). 31078660 10.1016/j.jbi.2019.103208 PMC7254481

