# Supplementary material for: Isatuximab, carfilzomib, lenalidomide and dexamethasone in newly diagnosed multiple myeloma: a randomized phase 3 trial
Source: Nat Med. 2026 Apr 6;32(5):1773–82. doi: 10.1038/s41591-026-04282-0 (PMC13190300; doi:10.1038/s41591-026-04282-0)
Supplement: Supplementary file 1 — Supplementary results, list of study sites, Redacted Trial Protocol and Redacted Statistical Analysis Plan. [file 41591_2026_4282_MOESM1_ESM.pdf]

nature medicine

Supplementary information https://doi.org/10.1038/s41591-026-04282-0

Isatuximab, carfilzomib, lenalidomide and dexamethasone in newly diagnosed multiple myeloma: a randomized phase 3 trial

In the format provided by the authors and unedited

Summary

Study sites **2**

Supplementary results **3**

Dose reductions and drug discontinuations due to toxicity 3

Isatuximab *3*

Carfilzomib *3*

Lenalidomide *4*

Dexamethasone *4*

Stem-cell mobilization details 5

Redacted trial protocol **6**

Redacted statistical analysis plan **135**

Study sites

The following study sites enrolled at least 1 patient in the EMN24 IsKia trial:

- University Medical Center, Groningen, Groningen, the Netherlands (principal investigator: Wilfried Roeloffzen)
- General Hospital of Athens 'Alexandra' (NKUA), Athens, Greece (Meletios Athanasios Dimopoulos)
- Hospital Clinic de Barcelona, Barcelona, Spain (Laura Rosiñol)
- Amphia Ziekenhuizen, Breda, the Netherlands (Marjolein van der Klift)
- H.U. Germans Trias i Pujol, Badalona, Barcelona, Spain (Albert Oriol)
- Theageneio General Hospital, Thessaloniki, Greece (Eirini Katodritou)
- ZAS Cadix, Antwerp, Belgium (Ka Lung Wu)
- Clínica Universidad de Navarra, Pamplona, Spain (Paula Rodríguez Otero)
- University Hospital Ostrava, Ostrava, Czech Republic (Roman Hájek)
- A.O.U. Careggi, Florence, Italy (Elisabetta Antonioli)
- H.U. 12 de Octubre, Madrid, Spain (Joaquín Martínez-López)
- S. Antonius Hospital, Nieuwegein, the Netherlands (Elena M. van Leeuwen-Segarceanu)
- A.O.U. di Bologna - Policlinico S. Orsola Malpighi, Bologna, Italy (Michele Cavo, Elena Zamagni)
- SSD Clinical Trial in Oncoematologia e Mieloma Multiplo, Department of Oncology, A.O.U. Città della Salute e della Scienza di Torino, University of Torino, Torino, Italy (Francesca Gay)
- Vrije Universiteit Medical Center (VUMC), Amsterdam, the Netherlands (Niels W. C. J. van de Donk)
- University Medical Center Hamburg-Eppendorf, Hamburg, Germany (Katja Weisel)
- University Hospital Brno, Brno, Czech Republic (Luděk Pour)
- University Hospital Hradec Kralove, Czech Republic (Jakub Radocha)
- A.O. Spedali Civili di Brescia, Brescia, Italy (Angelo Belotti)
- Erasmus University Medical Center, Rotterdam, the Netherlands (Annemiek Broijl)
- Policlinico S. Matteo Fondazione IRCCS, Pavia, Italy (Silvia Mangiacavalli)
- Medisch centrum Leeuwarden Zuid, Leeuwarden, the Netherlands (Esther de Waal)
- H.U. Marqués de Valdecilla, Santander, Spain (Enrique Ocio)
- Albert Schweitzer Hospital, Dordrecht, the Netherlands (Mark-David Levin)
- Oslo University Hospital, Oslo, Norway (Fredrik Schjesvold)
- H.U. de Salamanca, Salamanca, Spain (María Victoria Mateos)
- A.O. S. Croce e Carle, Cuneo, Italy (Mariella Grasso)
- Haga Ziekenhuis, Den Haag, the Netherlands (Paula Ypma)
- Canisius Wilhelmina Hospital, Nijmegen, the Netherlands (Susan de Jonge-Peeters)
- S. Olavs University Hospital, Trondheim, Norway (Tobias S. Slørdahl)
- General University Hospital (VFN)-Internal Medicine, Hematology Clinic, Prague, Czech Republic (Jan Straub)
- A.O.U. Maggiore della Carità, Novara, Italy (Gloria Margiotta-Casaluci)
- Noordwest Ziekenhuisgroep, Alkmaar, the Netherlands (Matthijs Westerman)
- Klinikum rechts der Isar der Technischen Universitat Muenche, Munchen, Germany (Florian Bassermann)
- A.O.U. delle Marche, Ancona, Italy (Massimo Offidani)
- A.O.U. Consorziale Policlinico - Ematologia con Trapianto, Bari, Italy (Pellegrino Musto)
- Azienda USL di Pescara P.O. dello Spirito Santo, Pescara, Italy (Anna Maria Morelli, Mauro Di Ianni)
- A.O.U. Ospedali Riuniti di Trieste, Trieste, Italy (Giovanni Maria De Sabbata)
- Geleen-Zuyderland Medical Center, Sittard, the Netherlands (Kon-Siong Jie)
- Freiburg University Hospital, Freiburg, the Netherlands, Germany (Monika Engelhardt)
- Ospedale 'Infermi', Rimini, Italy (Patrizia Tosi)
- Meander Medisch Centrum, Amersfoort, the Netherlands (Josien Regelink)

All patients were enrolled between October 7, 2020 and November 15, 2021.

Supplementary results

Dose reductions and drug discontinuations due to toxicity

Isatuximab

Per protocol, isatuximab dose reductions were not allowed. In the isatuximab plus carfilzomib-lenalidomide-dexamethasone (Isa-KRd arm), 2 (1%) of 151 patients discontinued isatuximab (1 due to respiratory failure during induction and 1 due to infusion-related reaction during the post-autologous stem-cell transplantation [ASCT] full-dose consolidation phase). Discontinuations did not occur during the light consolidation phase.

Carfilzomib

• During the *induction phase*, carfilzomib was reduced in 22 (15%) of 151 and 7 (5%) of 151 patients in the Isa-KRd and KRd arms, respectively. The main reasons for carfilzomib dose reduction were hepatic enzyme elevation (grade 1–2: Isa-KRd, n=5 and KRd n=1; grade 3: Isa-KRd n=4) and hypertension (grade 1–2: Isa-KRd, n=1 and KRd n=1; grade 3: Isa-KRd n=1). During the induction phase, 3 (2%) of 151 patients in the Isa-KRd arm discontinued carfilzomib (cardiac failure, n=1; tubulointerstitial nephritis, n=1; microangiopathic hemolytic anemia, n=1) vs. 0 (0%) of 151 patients in the KRd arm.

• During the *post-ASCT full-dose consolidation phase*, carfilzomib was reduced in 12 (9%) of 135 and 9 (6%) of 139 patients in the Isa-KRd and KRd arms, respectively. The main reasons for carfilzomib dose reduction were liver function test increased (grade 3: Isa-KRd n=1; grade 1–2: KRd n=2) and cardiac toxicity (grade 1–2 coronary artery disease: Isa-KRd n=1; grade 1–2 pericardial effusion and valve incompetence: Isa-KRd n=1; grade 1–2 myocardial infarction: KRd n=1). No patients discontinued carfilzomib during the post-ASCT full-dose consolidation phase. In patients who received a reduced dose of carfilzomib by the end of the post-ASCT full-dose consolidation phase, 45 mg/m^2 was the last administered dose in 17 (11%) of 151 patients in the Isa-KRd arm and in 9 (6%) of 151 patients in the KRd arm; 36 mg/m^2 was the last dose administered in 2 (1%) of 151 patients in the Isa-KRd arm and in 3 (2%) of 151 patients in the KRd arm; 27 mg/m^2 was the last dose administered in 4 (3%) of 151 patients in the Isa-KRd arm and in 2 (1%) of 151 patients in the KRd arm. A carfilzomib dose re-escalation (to 56 mg/m^2) occurred in 6 (4%) of 151 patients in the Isa-KRd arm and in 1 (1%) of 151 patients in the KRd arm (Extended Data Table 5).

• During the *light-consolidation phase*, a carfilzomib dose reduction was required in 15 (12%) of 126 and 22 (16%) of 136 patients in the Isa-KRd and KRd arms, respectively (P=0.38). The main reasons for carfilzomib dose reduction were non-hematologic adverse events (AEs). No specific AE occurred more frequently than others, with each reported in only 1 or 2 patients. Most AEs were of grade 1–2 (12 patients [10%] in the Isa-KRd vs. 10 [7%] in the KRd arm). Grade 3 non-hematologic AEs were the cause of dose reduction in 3 (2%) vs. 6 (4%) patients in the Isa-KRd vs. KRd arms. Only 1 patient in the KRd arm reduced dose due to a grade 4 AE. Seven (6%) of 126 patients in the Isa-KRd arm discontinued carfilzomib (vertigo, n=1; ejection fraction decreased, n=1; pneumonia, n=1; hypertension, n=1; thrombocytopenia, anemia, and neutropenia, n=1; cardiac disorder, n=1; unknown, n=1) vs. 12 (9%) of 136 patients in the KRd arm (thrombotic microangiopathy, n=2; mitral valve disease, n=1; pyrexia, n=1; liver function test increased, n=1; ventricular extrasystoles, n=1; nasopharyngitis, n=1; hypertension, n=1; pyrexia, n=1; electrocardiogram QT prolonged, n=1; retinopathy hypertensive and hypertension, n=1; nausea and/or vomiting, n=1; unknown, n=1). In patients who received a reduced dose of carfilzomib, 45 mg/m^2 was the last administered dose in 8 (6%) of 126 patients

in the Isa-KRd arm and in 14 (10%) of 136 patients in the KRd arm; 36 mg/m^2 was the last dose administered in 4 (3%) of 126 patients in the Isa-KRd arm and in 4 (3%) of 136 patients in the KRd arm; 27 mg/m^2 was the last dose administered in 3 (2%) of 126 patients in the Isa-KRd arm and in 4 (3%) of 136 patients in the KRd arm (Extended Data Table 5).

Lenalidomide

• During the *induction phase*, lenalidomide was reduced in 40 (26%) of 151 and 20 (13%) of 151 patients in the Isa-KRd and KRd arms, respectively. The main reasons for lenalidomide dose reduction were grade 3–4 neutropenia (Isa-KRd, n=14; KRd, n=4), skin rash (grade 1–2: Isa-KRd, n=6 and KRd, n=2; grade 3: Isa-KRd, n=1 and KRd, n=3), hepatic enzyme elevation (grade 1–2: Isa-KRd, n=3; grade 3: IsaKRd, n=2 and KRd, n=1), infections (grade 1–2: Isa-KRd, n=1; grade 3–4: Isa-KRd n=1 and KRd, n=1), and renal failure (grade 1–2: Isa-KRd, n=2 and KRd, n=1; Grade 3: Isa-KRd, n=1). One (1%) of 151 patients in the Isa-KRd arm discontinued lenalidomide (rash, n=1) vs. 0 (0%) of 151 in the KRd arm.

• During the *post-ASCT full-dose consolidation phase*, lenalidomide was reduced in 28 (21%) of 135 and 23 (17%) of 139 patients in the Isa-KRd and KRd arms, respectively. The main reasons for lenalidomide dose reduction were neutropenia (grade 1–2: Isa-KRd, n=2; grade 3–4: Isa-KRd, n=16 and KRd, n=6), and fatigue or asthenia (grade 1–2: IsaKRd, n=2 and KRd, n=3; grade 3: KRd, n=1). No patients discontinued lenalidomide during the post-ASCT full-dose consolidation phase. In patients who received a reduced dose of lenalidomide by the end of the post-ASCT full-dose consolidation phase, 20 mg was the last administered dose in 29 (19%) of 151 patients in the Isa-KRd arm and in 19 (13%) of 151 patients in the KRd arm; 15 mg was the last administered dose in 17 (11%) of 151 patients in the Isa-KRd arm and in 11 (7%) of 151 patients in the KRd arm; 10 mg was the last administered dose in 6 (4%) of 151 patients in the Isa-KRd arm and in 4 (3%) of 151 patients in the KRd arm; 7.5 mg was the last administered dose in 1 (1%) of 151 patients in the KRd arm and in no patient in the Isa-KRd arm; 5 mg was the last administered dose in 3 (2%) of 151 patients in the Isa-KRd and in 1 (1%) of 151 patients in the KRd arm (Extended Data Table 5). A lenalidomide dose re-escalation (to 25 mg) occurred in 4 (3%) of 151 patients in the Isa-KRd arm and in 1 (1%) patient in the KRd arm.

• During the *light-consolidation phase*, a lenalidomide dose reduction was required in 18 (14%) of 126 and 17 (12%) of 136 patients in the Isa-KRd and KRd arms, respectively. The main reasons for lenalidomide dose reduction were neutropenia (Isa-KRd, n=6; KRd, n= 7), thrombocytopenia (Isa-KRd, n=2; KRd, n= 2), diarrhea (Isa-KRd, n=3), and fatigue or asthenia (Isa-KRd, n=1; KRd, n= 4). Six (5%) of 126 patients in the Isa-KRd arm discontinued lenalidomide (neutropenia, n=2; vertigo, n=1; bronchitis, n=1; rash, n=1; cerebrovascular accident, n=1) vs. 1 (1%) of 136 patients in the KRd arm (muscle spasms, n=1). In patients who received a reduced dose of lenalidomide, 5 mg was the last administered dose in 18 (13%) of 136 patients in the Isa-KRd arm and in 17 (13%) of 126 patients in the KRd arm (Extended Data Table 5).

Dexamethasone

• During the *induction phase*, dexamethasone was reduced in 18 (12%) of 151 and 11 (7%) of 151 patients in the Isa-KRd arm and KRd arms, respectively. The main reasons for dexamethasone dose reduction were psychiatric disorders, including insomnia and mood alteration (grade 1–2: Isa-KRd, n=3 and KRd, n=3; grade 3–4: Isa-KRd, n=2 and KRd, n=1) and infections (grade 1–2: Isa-KRd, n=2; grade 4: Isa-KRd, n=1). During the induction phase, 1 (1%) of 151 patients in the Isa-KRd arm discontinued dexamethasone (acute pancreatitis, n=1) vs. 0 (0%) of 151 patients in the KRd arm.

• During the *post-ASCT full-dose consolidation phase*, dexamethasone was reduced in 20 (15%) of 135 and 21 (15%) of 139 patients in the Isa-KRd and KRd arms, respectively. The main reasons for dexamethasone dose reduction were psychiatric disorders, including insomnia and mood alteration (grade 1–2: Isa-KRd, n=4; grade 3–4: Isa-KRd, n=7). No patients discontinued dexamethasone during the post-ASCT full-dose consolidation phase. In patients who received a reduced dose of dexamethasone by the end of the post-ASCT full-dose consolidation phase, 20 mg was the last administered dose in 34 (23%) of 151 patients in the Isa-KRd arm and in 21 (14%) of 151 patients in the KRd arm; 10 mg was the last administered dose in 1 (1%) of 151 patients in the Isa-KRd arm and in 6 (4%) of 151 patients in the KRd arm; 4 mg was the last administered dose in 0 (0%) of 151 patients in the Isa-KRd arm and in 1 (1%) of 151 patients KRd arm (Extended Data Table 5). A dexamethasone dose re-escalation (to 40 mg) occurred in 1 (1%) of 151 patients in the KRd arm.

• During the *light-consolidation phase*, a dexamethasone dose reduction was required in 16 (13%) of 126 and 17 (12%) of 136 patients in the Isa-KRd and KRd arms, respectively (P=1.00). The main reasons for dexamethasone dose reduction during the light-consolidation phase were respiratory tract infection (Isa-KRd, n=2; KRd, n=1), insomnia (Isa-KRd, n= 3; KRd, n= 3), and mood altered (Isa-KRd, n=1; KRd, n=2). One (1%) of 126 patients in the Isa-KRd arm discontinued dexamethasone (respiratory tract infection, n=1) vs. 7 (5%) of 136 patients in the KRd arm (respiratory tract infection n=1; influenza n=1; thrombotic microangiopathy, n=1; edema, n=1; back pain, n=1; thrombotic microangiopathy, n=1; malaise n=1). In patients who received a reduced dose of dexamethasone, 10 mg was the last administered dose in 15 (12%) of 126 patients in the Isa-KRd arm and in 16 (12%) of 136 patients in the KRd arm; 4 mg was the last administered dose in 1 (1%) of 126 patients in the Isa-KRd arm and in 1 (1%) of 136 patients KRd arm (Extended Data Table 5).

Stem-cell mobilization details

The median number of aphereses was 2 (IQR 1–2) in the Isa-KRd and 1 (IQR 1–2) in the KRd arm (P=0.079).

Hematopoietic reconstitution was obtained in 100% of patients, with a median time to neutrophil recovery of 15 (IQR 12–27) vs. 14 days (IQR 12–27; P=0.35) and a median time to platelet recovery of 18 (IQR 14–25) vs. 16 days (IQR 13–24; P=0.12) in the Isa-KRd vs. KRd arms, respectively.

Redacted trial protocol

Phase III study of Isatuximab-Carfilzomib-Lenalidomide-Dexamethasone (Isa-KRd) versus Carfilzomib-Lenalidomide-Dexamethasone (KRd) in newly diagnosed multiple myeloma patients eligible for autologous stem cell transplantation (IsKia TRIAL)

PROTOCOL

Coordinating Investigators: **Francesca Gay and Annemiek Broijl**

Sponsor: Stichting European Myeloma Network (EMN)

EudraCT number: 2019-004844-32

EU CT number: 2024-513422-38-00

CONFIDENTIAL

The information contained in this document is regarded as confidential and, except to the extent necessary to obtain informed consent, may not be disclosed to another party unless such disclosure is required by law or regulations. Persons to whom the information is disclosed must be informed that the information is confidential and may not be further disclosed by them.

COORDINATING INVESTIGATORS SIGNATURE PAGE

[redacted] [redacted]

[redacted]

[redacted] [redacted]

[redacted]

By my signature, I agree to personally supervise the conduct of this study in my affiliation and to ensure its conduct in compliance with the protocol, informed consent, EC procedures, the Declaration of Helsinki, ICH Good Clinical Practices guideline, the EU directive Good Clinical Practice, and local regulations governing the conduct of clinical studies

LOCAL INVESTIGATOR SIGNATURE PAGE

[redacted] ______________________________

[redacted] [redacted]

[redacted]

By my signature, I agree to personally supervise the conduct of this study in my affiliation and to ensure its conduct in compliance with the protocol, informed consent, EC procedures, the Declaration of Helsinki, ICH Good Clinical Practices guideline, the EU directive Good Clinical Practice, and local regulations governing the conduct of clinical studies.

SPONSOR SIGNATURE PAGE

[redacted] [redacted]

[redacted]

[redacted]

By my signature, I agree to personally supervise the conduct of this study in my affiliation and to ensure its conduct in compliance with the protocol, informed consent, EC procedures, the Declaration of Helsinki, ICH Good Clinical Practices guideline, the EU directive Good Clinical Practice, and local regulations governing the conduct of clinical studies

TABLE OF CONTENTS

SUMMARY OF CHANGES – AMENDMENT 3/VERSION 4.0, 14-JAN-2025 8

1. INVESTIGATORS AND STUDY ADMINISTRATIVE STRUCTURE 10

2. SCHEME OF STUDY 11

3. SYNOPSIS 12

4. INTRODUCTION AND RATIONALE 16

4.1 Myeloma specific background and current treatment landscape 16
4.2 Isatuximab 18
4.3 Carfilzomib 23
4.4 Rationale of the study 28

5. STUDY OBJECTIVES 29

5.1 Primary Objective 29
5.2 Secondary Objectives 29

6. STUDY DESIGN 30

7. STUDY POPULATION 30

7.1 Eligibility for registration/randomization 30
Inclusion criteria 30
Exclusion criteria 32

8. TREATMENT 34

8.1 Arm A 34
8.1.1 Induction schedule 34
8.1.2 Transplant (as per Standard of Care) 34
8.1.3 Consolidation schedule 35
8.1.4 Light consolidation schedule 35
8.1.5 Dose adjustments 36
8.1.6 Special precautions and supportive care 36
8.2 Arm B 36
8.2.1 Induction schedule 36
8.2.2 Transplant (as per Standard of Care) 36
8.2.3 Consolidation schedule 37
8.2.4 Light consolidation schedule 37
8.2.5 Dose adjustments 37
8.2.6 Recommended actions for adverse events 39
8.3 Study drug administration 45
8.3.1 Isatuximab administration 45
8.3.2 Carfilzomib administration 47
8.4 Special precautions and supportive care 48
8.4.1. Tumor lysis syndrome 48
8.4.2. Changes in Body Surface Area (BSA) 50
8.4.3 Guidelines for the management of infusion reactions 50
8.4.4. Guidelines for management of allergic and dermatologic reactions to lenalidomide syndrome 51
8.5 Mandatory concomitant medication 51
8.5.1 Pre-medication 51
8.5.2 Pregnancy prevention 53
8.6 Recommended concomitant medications 54
8.6.1 Supportive bone therapy 54
8.6.2 Transfusions/Growth Factors 55
8.6.3 Prevention of Deep Venous Thrombosis 55
8.6.4 Prophylaxis for Herpes Zoster Reactivation 55
8.7 Permitted Therapies 55

8.8 Prohibited therapies 56
8.9 Study drugs information 56
Summary of known and potential risks 56
Preparation and labeling 57
Storage and handling 57
Study drugs supply 57
Drug accountability 58
Study drug return and destruction 58

9. FOLLOW-UP MRD STUDY 58

10. STUDY PROCEDURES 59

10.1 Time of clinical evaluations 59
Screening 59
Treatment phase 60
Continuous Procedures 61
Observation 61
Long Term Follow up (LTFU) 62
10.2 Time and events schedule 63
10.3 MRD 70
10.4 Quality of Life assessment 71
10.5 Central review 72
10.6 Side study 72

11. WITHDRAWAL OF PATIENTS OR PREMATURE TERMINATION OF THE STUDY 73

11.1 Withdrawal of individual patients from protocol treatment 73
11.2 Follow up of patients withdrawn from protocol treatment 73
11.3 Withdrawal of informed consent 74
11.4 Premature termination of the study 74

12. SAFETY 74

12.1 Definitions 74
12.2 Adverse event 75
Reporting of adverse events 75
Anticipated disease-related Events 76
Reporting of anticipated disease-related Adverse Events 76
Follow up of adverse events 76
Adverse Event of Special Interest (AESI) 77
12.3 Serious Adverse Events 77
Reporting of serious adverse events 77
Causality assessment of Serious Adverse Events 78
Follow up of Serious Adverse Events 78
Processing of serious adverse event reports 79
12.4 Reporting Suspected Unexpected Serious Adverse Reactions 79
12.5 Pregnancies 79
12.6 Second Primary Malignancies 80
12.7 Reporting of safety issues 80
12.8 Annual safety report 81
12.9 Independent Data Monitoring Committee 81
12.10 Product Complaints 81

13. ENDPOINTS 81

13.1 Primary endpoint – Rate of MRD negativity after ASCT consolidation treatment 81
13.2 Secondary endpoints 82
Key secondary endpoints 82
Other secondary endpoints 82

14. STATISTICAL CONSIDERATIONS 84

14.1 Patient numbers and power considerations 84
14.2 Statistical analysis 85

Multiplicity 85
Efficacy analysis 86
Toxicity analysis 86
Additional analyses 87
Statistical analysis plan 87
14.3 Interim analysis 87

15. REGISTRATION AND RANDOMIZATION 88

15.1 Regulatory Documentation 88
15.2 Registration and Randomization 88

16. DATA COLLECTION AND QUALITY ASSURANCE 88

16.1 Case Report Forms 88
16.2 Data quality assurance 89

17. ETHICS 89

17.1 Accredited ethics committee 89
17.2 Ethical conduct of the study 89
17.3 Patient information and consent 89
17.4 Benefits and risks assessment 90
17.5 Trial insurance 92

18. ADMINISTRATIVE ASPECTS AND PUBLICATION 92

18.1 Personal data protection 92
18.2 Handling and storage of data and documents 93
Patient confidentiality 93
Filing of essential documents 93
Record retention 93
Storage of samples 94
18.3 Amendments 94
18.4 Annual progress report 94
18.5 End of trial report 95
18.6 Publication policy 95

19. GLOSSARY OF ABBREVIATIONS 96

20. REFERENCES 98

21. APPENDIX 102

A. Criteria for MM and measurable disease 102
B. Response Criteria for Multiple Myeloma 104
C. ZUBROD-ECOG-WHO Performance Status Scale 107
D. Common Terminology Criteria for Adverse Events 108
E. NYHA scoring list 109
F. Modified Diet in Renal Disease Formula 110
G. Corrected calcium Formula 111
H. Quality of Life Questionnaire EORTC QLQ-C30 (version 3) 112
I. EORTC QLQ-MY20 Quality of Life Questionnaire EORTC QLQ-MY20 116
J. Questionnaire EQ-5D-5L 118
K. FACT/GOG ntx questionnaire (version 4.0) 120
L. Correlative studies (central labs) 123
M. Mass spectrometry project 124
N. Humoral and cellular immune response to SARS-CoV-2 vaccine/infection in MM patients treated within the phase III EMN24 trial 127

Summary of changes – Amendment 3/Version 4.0, 14-Jan-2025

Overall reason for the amendment: the main change is the update of the statistical assumptions for the PFS interim analysis and the inclusion of additional MRD timepoints for patients at least in VGPR that have completed or not the light consolidation phase in absence of disease progression or further antimyeloma therapies..

Applicable Sections	Description of Changes
**1.** Rationale: This section has been added to include the additional MRD timepoints applicable for patients at least in VGPR that did not complete the light consolidation treatment. The same MRD timepoints were added in the previous protocol amendment for the patients at least in VGPR that completed the light consolidation phase.	
Section 9 – MRD Follow-Up Study	Patients at least in VGPR that completed or not the light consolidation treatment, will undergo to additional MRD monitoring at 1 year, 2 years and 3 years after the end of light consolidation (NGS + NGF) in absence of disease progression or further anti-myeloma therapies.
Section 10.3 – MRD	The MRD follow-up timepoints have been updated. All the patients at least in VGPR not in progression or in any subsequent anti-myeloma therapy should collect samples for MRD once a year for 3 years after the end of treatment.
**2.** Rationale: the LTFU assumptions have been updated	
Section10.1 – Time of clinical evaluation - LTFU	The LTFU will be performed 9 years after the last patient randomized or after 170 death events, whichever comes first.
**3.** Rationale: to update the process for reporting serious adverse events based on the latest assumptions.	
Section 12.3 – Serious adverse events – Processing of SAEs report	The reference document for carfilzomib and lenalidomide is the SmPC and no longer the IB.
**4.** Rationale: the statistical part for PFS and OS has been updated based on the latest scientific data available.	
Section 14.1 – Patient numbers and power considerations	The rates of 48 months PFS for the 2 arms have been revised and the minimal follow up period to reach the 91 PFS events is 71 months. The overall survival will be performed 9 years after last pt randomized or 170 death events.

Applicable Sections	Description of Changes
Section 14.3 – Interim analysis	The fifth interim analysis for PFS will be performed after approximately 68 events have been reached.

1. Investigators and study administrative structure

**Responsibility**	**Name**	**Affiliation/Address**
Sponsor	Stichting European Myeloma Network (EMN)	PPD [redacted]
Co-sponsor	EMN Trial Office Srl	PPD [redacted]
Coordinating Investigators	Dr. Francesca Gay<br>Dr. Annemiek Broijl	Torino, Italy<br>Rotterdam, The Netherlands
Scientific Coordinators	Prof. Pieter Sonneveld<br>Prof. Mario Boccadoro<br>Prof. Joan Bladé<br>Prof. Meletios A. Dimopoulos<br>Prof. Hermann Einsele<br>Prof. Roman Hájek<br>Dr. Ka Lung Wu<br>Dr. Fredrik Schjesvold	Rotterdam,The Netherlands<br>Torino, Italy<br>Barcelona, Spain<br>Athens, Greece<br>Würzburg, Germany<br>Ostrava, Czech Republic<br>Antwerpen, Belgium<br>Oslo, Norway
Writing Committee	Prof. Pieter Sonneveld<br>Prof. Mario Boccadoro<br>Prof. Joan Bladé<br>Prof. Meletios A. Dimopoulos<br>Prof. Hermann Einsele<br>Prof. Roman Hájek<br>Dr. Ka Lung Wu<br>Dr. Fredrik Schjesvold<br>Dr. Elena Zamagni	Rotterdam, The Netherlands<br>Torino, Italy<br>Barcelona, Spain<br>Athens, Greece<br>Würzburg, Germany<br>Ostrava, Czech Republic<br>Antwerpen, Belgium<br>Oslo, Norway<br>Bologna, Italy
Statistician	Dr. Stefano Spada	Torino, Italy
Serious Adverse Events (SAEs) notification	EMN Safety Office	PPD [redacted]

2. Scheme of study

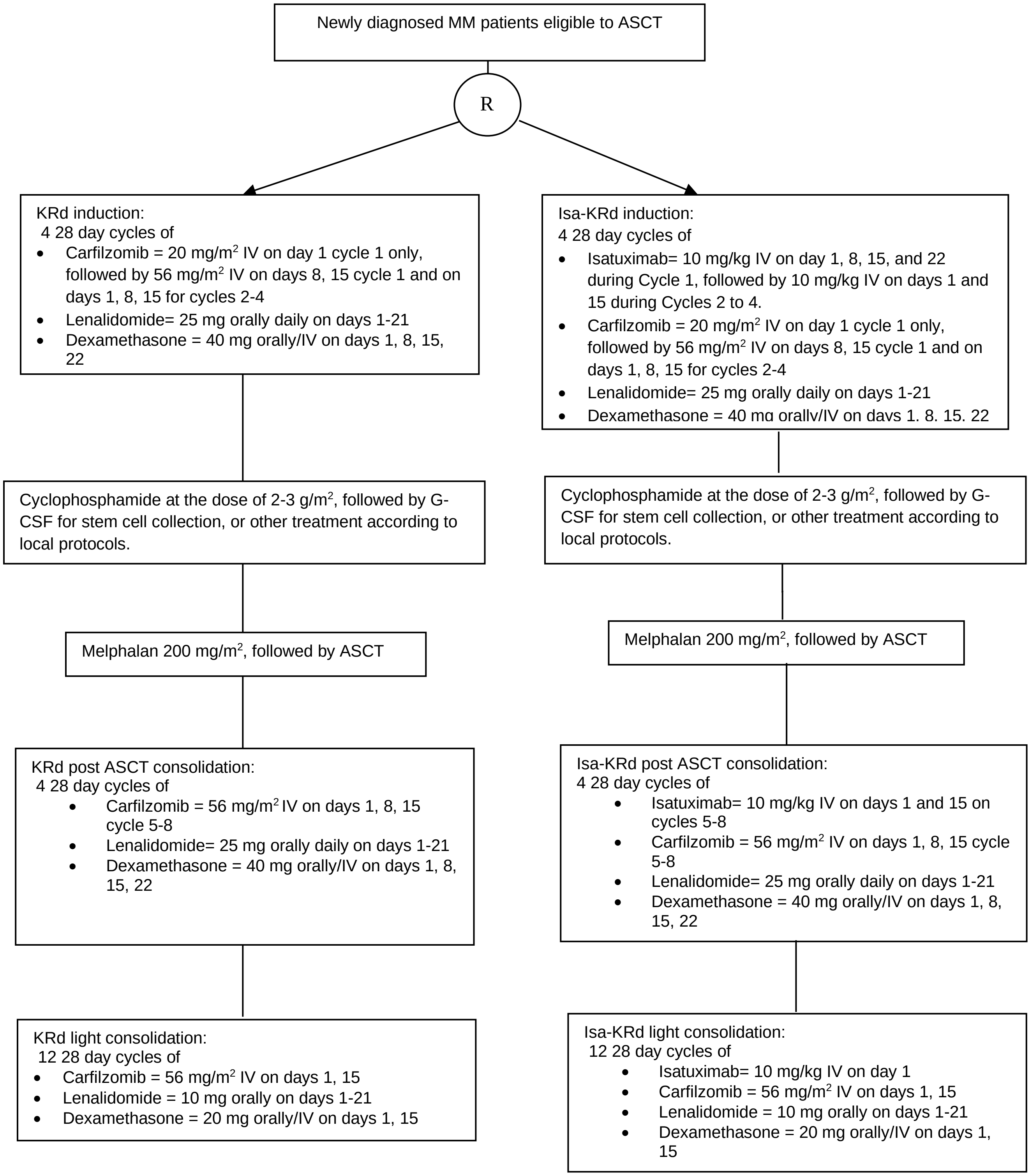

3. Synopsis

Rationale	1. Preclinical data demonstrated that isatuximab activity was enhanced when combined with lenalidomide (Jiang H, et al, Leukemia 2016;30: 309-408)<br>2. A phase Ib trial suggested that isatuximab combined with lenalidomide and dexamethasone is active and tolerated in heavily pretreated patients with RRMM (Martin T, et al, Blood. 2017 Jun 22;129(25):3294-3303)<br>3. Results of KRd combination appear to be improved by incorporation of ASCT with sCR rate of 74% and 3-year PFS of 86% (Jakubowiak A, et al, EHA 2016 Abstract S101; Zimmerman TM, et al, ASH 2016 Abstract 675)<br>4. Results of phase Ib trial with isatuximab in association with Kd in RRMM demonstrated that this drug combination appears safe; toxicity is c/w the AEs of the individual agents with few grade 3/4 AEs. Encouraging anti-myeloma activity (ORR 66%) was seen at all dose levels. Isatuximab 10 mg/Kg QW x 4 then Q2W dosing was selected for an ongoing Phase III trial of Isatuximab + Kd versus Kd (Chari et al Phase I-b study of isatuximab + carfilzomib in relapsed and refractory multiple myeloma (RRMM), DOI: 10.1200/JCO.2018.36.15_suppl.8014).
Study objectives	**Primary Objective:**<br>To compare rate of Minimal Residual Disease (MRD) negativity by NGS between Isa-KRd and KRd in post ASCT consolidation treatment<br>**Secondary Objectives:**<br>Key secondary objectives<br>- Rate of MRD negativity after induction by NGS<br>- To compare progression-free survival (PFS) in the 2 treatment arms.<br>Other secondary objectives<br>- Rate of MRD negativity after light consolidation by NGS<br>- Determine the overall response rate (ORR), VGPR,

CR, sCR rate after induction, ASCT, post ASCT consolidation, light consolidation in the 2 treatment arms

- Determine the rate of MRD negativity (by NGS) after ASCT
- Determine the rate of MRD negativity (by NGF) after induction, ASCT, post ASCT consolidation and light consolidation.
- Determine the duration of response (DOR) in the 2 treatment arms
- Determine the duration of MRD negativity (by NGS and NGF)
- Determine the rate of sustained for 1-year MRD negativity (by NGF and NGS) (from post ASCT consolidation to post light consolidation)
- Determine the time to progression (TTP) in the 2 treatment arms
- Determine the overall survival (OS) in the 2 treatment arms
- Determine the time to next therapy (TNT) in the 2 treatment arms
- Determine the PFS2 in the 2 treatment arms
- Determine whether tumor response and outcome may change in subgroups with different prognosis according to current prognostic factors
- Determine safety in the 2 treatment arms
- Determine the success of stem cell harvest
- Determine the success of engraftment after ASCT
- Determine Quality of life in the two treatment arms

<u>Study design</u>

This protocol is a phase III study designed to compare the efficacy and the safety of Isa-KRd induction, transplant, Isa-KRd post ASCT consolidation and Isa-KRd light consolidation vs KRd induction, transplant, KRd post ASCT consolidation and KRd light consolidation

After confirmation of eligibility criteria patients will be randomized to one of the 2 treatment groups in a 1:1 randomization ratio.

Patient population	Newly diagnosed autologous stem cell transplantation (ASCT) eligible MM patients
Intervention	After confirmation of eligibility criteria patients will be randomized to one of the 2 treatment groups in a 1:1 randomization ratio: Isatuximab-carfilzomib-lenalidomide-dexamethasone (ARM A) or carfilzomib-lenalidomide-dexamethasone (ARM B) for 4 cycles followed by ASCT. Each arm will receive then 4 cycles of post ASCT consolidation followed by 12 cycles of light consolidation with isatuximab-carfilzomib-lenalidomide-dexamethasone (ARM A) or carfilzomib-lenalidomide-dexamethasone (ARM B)
Duration of treatment	About 24 months<br>Subsequently patients will be followed for 9 years after the last subject randomized
Target number of patients	300
Expected duration of accrual	14 months
Main study endpoints	Minimal Residual disease rate evaluated by means of next generation sequencing and by next generation flow cytometry (cut off 10^{-5})<br>Progression-free survival (PFS)<br>Overall response rate (ORR)<br>Duration of response (DOR)<br>Sustained MRD negativity at 1 year<br>Time to progression (TTP)<br>Progression-free survival 2 (PFS2)<br>Overall survival (OS)<br>Time to next therapy (TNT)
Benefit and nature and extent of the burden and risks associated with participation	Patients who decide to participate will undergo blood test and bone marrow aspirate and biopsy as specified in Schedule of Study assessment (paragraph 9.2). These include tests

	according to standard clinical practice, plus MRD monitoring. Volumes drawn are considered adequate
Planned interim analysis and IDMC	Five interim analyses are planned when data are available of: the first 75 patients completing the 4 cycles of induction therapy; the first 75 patients data of stem cell collection; the first 75 patients completing the 4 cycles of post ASCT consolidation therapy; the first 75 patients completing the 4 cycles of light-consolidation therapy; after 75% of PFS events have been observed.<br><br>Results of interim analyses and subsequent analyses will be presented to the principal investigators and to an independent data monitoring committee (IDMC).

4. Introduction and rationale

4.1 Myeloma specific background and current treatment landscape

Multiple myeloma (MM) is the second most common hematologic malignancy, characterized by the malignant proliferation of clonal plasma cells in the bone marrow microenvironment, monoclonal protein in blood or urine, and associated organ dysfunction. The annual incidence rate in Western countries is of 5.6 cases per 100.000 people (1). The median age at diagnosis is 70 years (2). Symptomatic disease requires therapy. It is characterized by organ damage caused by plasma cell proliferation and is defined by the presence of the CRAB features (C:hypercalcemia, R:renal failure, A:anemia and B:bone disease). Presence of Myeloma Defining Events (MDE) is also a criterium to starting therapy (3).

The standard treatment for newly diagnosed MM patients eligible for high dose chemotherapy and autologous transplant (ASCT) consists of an induction therapy using proteasome inhibitor plus immunomodulatory agents (4), followed by stem cell collection and ASCT (standard conditioning regimens Melphalan 200 mg/sqm). Combinations of bortezomib-thalidomide-dexamethasone and bortezomib-lenalidomide and dexamethasone have been widely used based on several trials. Both combinations are included in the ESMO guidelines.

Carfilzomib is a tetrapeptide epoxyketone proteasome inhibitor (PI) that binds selectively and irreversibly to the 20S proteasome, the proteolytic core particle within the 26S proteasome. Consequently, proteasome function after therapy can only be regained by de novo proteasome synthesis. Specifically, carfilzomib inhibits the chymotrypsin-like catalytic activity of the β5 subunit over the caspase-like catalytic activity of the β1 subunit or the trypsin-like catalytic activity of the β2 subunit, resulting in the accumulation of proteasome substrates and ultimately growth arrest and apoptosis (Hoy, 2016). Carfilzomib extensively penetrates all tissues, but the brain. It is metabolized largely extra-hepatically and rapidly cleared from the circulation by biliary and renal excretion (t1/2 = 15 to 30 minutes); < 1% is excreted intact (Kortuem and Stewart, 2013). Carfilzomib is structurally and mechanistically distinct from the dipeptide boronic acid proteasome inhibitor bortezomib (Velcade®). In addition, when measured against a broad panel of proteases including metallo, aspartyl, and serine proteases, carfilzomib demonstrated less reactivity against non-proteasomal proteases when compared to bortezomib (5, 6). Based upon the results of in vitro and in vivo studies, it is anticipated that the more intense and longer duration of proteasome inhibition that can be achieved with carfilzomib will result in enhanced anti-tumor activity compared to bortezomib. Continuous (72 hr) exposure to carfilzomib is associated with potent cytotoxic and pro-apoptotic activity across a broad panel of tumor-derived cell lines in culture (1,7). Incubation of hematologic tumor cell lines with carfilzomib leads to rapid inhibition of proteasome activity followed by accumulation of polyubiquitinated proteins and induction of apoptotic cell death. Carfilzomib has also been demonstrated to be cytotoxic in bortezomib-resistant tumor cell lines (5,8). In the relapse setting, a randomized phase III study compared treatment with Carfilzomib dexamethasone vs bortezomib-dexamethasone, showing the superiority of Carfilzomib in terms of both Progression-free survival and overall survival (9), thus providing the basis to evaluate carfilzomib in combination with other agents, to increase treatment efficacy.

Thalidomide was the first IMID discovered and its encouraging activity prompted the search for more potent and less toxic thalidomide derivates. Novel agents with increased anti-inflammatory activities and a more favorable toxicity profile were created by chemical modification of the structural backbone of thalidomide.

Lenalidomide is an oral glutamic acid derivative with direct anti-proliferative and pro-apoptotic effects. The similar activity of thalidomide is increased in vitro of 50-2000 times, in reducing cytokines production by lipopolysaccharide (LPS)-stimulated peripheral blood mononuclear cells (PBMC).

Thalidomide is limited by the incidence of peripheral neuropathy, whereas Lenalidomide is not. Combination of bortezomib-lenalidomide-dexamethasone was evaluated in patients without an immediate intent to transplant and proved to be well tolerated and superior to lenalidomide dexamethasone alone (10). The same combination has been evaluated also as pre transplant induction and post transplant consolidation in a phase III study, showing very good results in terms of tolerability and efficacy (11).

Based on the efficacy of the combination of first - generation proteasome inhibitor bortezomib plus lenalidomide and dexamethasone, and the potentially higher effectiveness of carfilzomib compared with bortezomib, studies have evaluated the combination of the more efficacious PI carfilzomib with lenalidomide in the upfront setting.

A phase Ib/II study assessed the combination of carfilzomib with lenalidomide and low-dose dexamethasone as a frontline treatment for MM. After 4 cycles transplant-eligible candidates underwent stem cell collection (SCC). The KRd treatment did not have an adverse impact on SCC and responses were rapid, improved with continuous treatment, and durable. Extended treatment in the KRd maintenance phase was also generally well tolerated and showed prolonged PFS. (24). A larger randomized study compared 4 cycles of KRd induction, transplant and 4 cycles of KRd consolidation vs KRd prolonged treatment (12 cycles) vs 4 cycles of induction with Carfilzomib-cyclophosphamide-dexamethasone (KCd), transplant and 4 cycles of KCd consolidation. Data from this trial showed that treatment was well tolerated, with no unexpected toxicity. Rate of response improved progressively with treatment, with 58% of patients receiving KRd achieving MRD negativity after consolidation (12).

Monoclonal antibodies against CD38 have demonstrated high efficacy in the treatment of newly diagnosed and relapse-refractory MM. The first anti CD38 monoclonal antibody to be approved for the treatment of MM is Daratumumab. Based on large phase III studies the combination of Daratumumab-lenalidomide-dexamethasone, Daratumumab bortezomib dexamethasone as well as Daratumumab single agent are approved for the treatment of relapsed refractory MM by EMA and FDA. EMA and FDA approved also upfront treatment with Daratumumab bortezomib melphalan and prednisone in patients with newly diagnosed MM not eligible for ASCT (13, 14, 15, 16). Other studies are underway evaluating Daratumumab in combination with VTD and VRD in the upfront setting in ASCT eligible patients (NCT02541383 and NCT03652064). A phase I study showed also the safety of first line therapy with Daratumumab- KRd (17). All trials available so far showed improved PFS adding the anti-CD38 monoclonal antibody to standards of care, with a very good safety profile (no overlapping toxicity, mainly

infusion reactions as adverse event related to MoAb, generally limited and manageable with common supportive care. Isatuximab is a IgG monoclonal antibody directed against CD38, with a similar mechanism of action of Daratumumab, and a similar safety profile. The drug has been evaluated as single agent and in combination with dexamethasone in the release setting, showing 30% ORR rate (18). Recent data showed also the efficacy and safety of the combination Isatuximab VRD in the upfront setting (19). Isatuximab is also under evaluation in a phase II trial in combination with KRd (NCT03104842) in newly diagnosed high risk MM patients.

4.2 Isatuximab

Isatuximab description

CD38 is highly and uniformly expressed on the malignant clonal populations of myeloma cells, as well as at relatively low levels on normal lymphoid, myeloid and some non hematopoietic tissues and thus represents a useful target treating Myeloma (20).

Isatuximab (SAR650984) is a chimeric monoclonal antibody that binds selectively to a unique epitope on the human surface antigen CD38. Isatuximab's cytotoxic properties are derived from multiple biological mechanisms, antibody-dependent cellular-mediated cytotoxicity (ADCC), complement-dependent cytotoxicity (CDC), antibody-dependent cellular phagocytosis (ADCP), direct induction of apoptosis (pro-apoptosis) without crosslinking, and inhibition of CD38 enzymatic activity.

The structural differences between isatuximab (chimeric mouse/human) and the other anti-CD38 antibody daratumumab (human) may account for distinct functional interactions with the IgG Fc receptors (FcRs). The epitope recognized by isatuximab encompasses the catalytic domain of the molecule and is different from that of daratumumab. The outcome of the interactions of the two different antibodies may explain the differences observed in terms of membrane dynamics, with isatuximab leading to internalization and daratumumab to generation of membrane-derived vesicles. The particular benefit provided by isatuximab is its sensitivity to the number of CD38 molecules present on target cells. Isatuximab saturates membrane CD38 and can be internalized. Isatuximab is reportedly one of the most efficient inhibitors of the enzymatic features exerted by CD38 (36).

Isatuximab nonclinical and clinical data

Please refer to the Investigator's Brochure for preclinical data of isatuximab administered in monotherapy and in combination with proteasome inhibitors or immunomodulator agents (bortezomib or lenalidomide) and for clinical data of isatuximab in single agent and in combination.

Isatuximab in combination with lenalidomide and dexamethasone (Study TCD11863)

The combination of isatuximab with standard doses of lenalidomide and dexamethasone was evaluated in the completed TCD11863 trial. Two schedules of isatuximab administration were evaluated, once every 2 weeks (Q2W) and once a week (QW)/Q2W. Fifty-seven patients were enrolled in this study.

The first part of the trial evaluated 3 doses of isatuximab 3, 5 and 10 mg/kg using the Q2W schedule of administration; among the patients treated at 10 mg/kg (n=24), the overall response rate (ORR) was 62.5% with PR rate of 20.8%, very good partial response (VGPR) 33.3% and stringent complete response (sCR) of 8.3%. Among the patients treated in the second part of the trial (QW/Q2W schedule), the ORR was 50% for both 10 mg/kg (n=12) and 20 mg/kg (n=10) QW/Q2W. At 10 mg/kg QW/Q2W, best overall response was PR for 2 patients and VGPR for 4 patients, whereas at the 20 mg/kg QW/Q2W dose, all responses were VGPR.

The most common TEAEs (reported in ≥20% of the patients), with the exclusion of TEAEs consistent with laboratory abnormalities consisted of infusion related reaction (56.1%; Grade 3-4: 8.8%), diarrhea (52.6%; Grade 3-4: 0), fatigue (49.1%; Grade 3-4: 7.0%), upper respiratory tract infection (40.4%; Grade 3-4: 0), nausea (35.1%; Grade 3-4: 0), insomnia (31.6%; Grade 3-4: 1.8%), pyrexia (31,6%; Grade 3-4: 0), dyspnea (28.1%; Grade 3-4: 3.5%), cough (26.3%; Grade 3-4: 0), headache (22.8%; Grade 3-4: 0), muscle spasms (22.8%; Grade 3-4: 0), vomiting (22.8%; Grade 3-4: 0), and nasal congestion (21.1%; Grade 3-4: 0).

For more details please refer to Investigator’s Brochure.

Isatuximab dose and regimen

Isatuximab as a single agent has shown efficacy with doses between 3 mg/kg and 10 mg/kg and above (21).

The recommended dose of isatuximab when administered in combination with other regimens has been determined based on Study TCD11863 (isatuximab in combination with Rd), and then used with Study TCD14079 and Study ICARIA (isatuximab in combination with pomalidomide and dexamethasone in RRMM), Study TCD13983 (isatuximab in combination with either bortezomib, cyclophosphamide, and dexamethasone or bortezomib, lenalidomide and dexamethasone). In all of these studies, exposure of isatuximab when administered in combination, was within the range of exposure reported for single agent therapy, for all the explored dose levels.

Although no evident difference was seen for tolerability, the available data of isatuximab in combination with lenalidomide or pomalidomide do not demonstrate major differences in efficacy between 10 and 20 mg/kg with comparable response rates in heavily pretreated patients. Pharmacokinetic/pharmacodynamic (PDy) analyses performed with TCD11863 data including trial

simulations and simulations of serum monoclonal (M)-protein profiles showed higher predicted ORR and reduction in M-protein at 8/12 weeks at doses ≥10 mg/kg. However, the benefit in terms of ORR increase or in terms of serum M-protein reduction appeared limited when increasing the dose from 10 to 20 mg/kg QW × 4 followed by Q2W. Therefore, based on clinical efficacy, safety, PK simulations, and PK/PDy analyses, the dose selected for further isatuximab combination studies is 10 mg/kg QW × 4 administrations followed by 10 mg/kg Q2W (please refer to investigator's brochure).

In the Phase Ib TCD13983 trial isatuximab was associated with bortezomib, lenalidomide and dexamethasone in NDMM patients not eligible for transplant (37). The primary objective was to evaluate safety and preliminary efficacy of this drug combination. Minimal residual disease (MRD) was evaluated using next generation sequencing (NGS) and flow cytometry (NGF) at a sensitivity of 10^{-6} in pts achieving at least a VGPR. 22 patients received at least 1 dose of isatuximab and 14 pts were eligible for preliminary efficacy analyses. Median age was 71 years. At data cut-off (Mar 22, 2018), the median number of cycles was 5.5. Three pts discontinued treatment: 2 pts due to isatuximab-related IRs (Grade2/3) and 1 pt withdrew consent; the remaining 19 (86%) pts are continuing treatment. Treatment emergent AEs (TEAEs) occurred in 19 pts. Most frequent TEAEs were constipation, IRs and peripheral edema, asthenia, diarrhea, and peripheral sensory neuropathy, hypotension, fatigue and respiratory tract infection, cough and dyspnea. Gr ≥3 AEs were reported in 46% and serious AEs (SAEs) in 18% pts. Treatment-related SAEs occurred in 2 pts (IR and pancreatitis). Gr 3/4 laboratory hematologic abnormalities: lymphopenia (8/22), neutropenia (4/22), thrombocytopenia (4/22). All responders had VGPR or CR except 1 pt with PR. Median time to first response was 1.4 months (end of C1) and, with a median follow-up of 7.49 months (at cut-off date), no pt has progressed, with all except 3 pts continuing on therapy. Five (38.5%) of 13 pts achieved MRD-negative status (by NGF and NGS, or NGS only). The preliminary data suggest that induction phase with isatuximab plus VRd followed by maintenance with isatuximab and Rd is well tolerated with a high ORR of 93%. Quality of CR may have been underestimated due to ISA interference which could be resolved with an interference assay.

The Phase III ICARIA trial evaluated the clinical benefit of isatuximab in combination with pomalidomide and low-dose dexamethasone compared with pomalidomide and low-dose dexamethasone alone in RRMM patients (38). Patients were randomly assigned in a 1:1 ratio to either isatuximab (10 mg/kg IV on Days 1, 8, 15, and 22 in the 1st cycle; Days 1 and 15 in subsequent cycles) plus pomalidomide (4 mg on Days 1–21) and dexamethasone (40 mg for patients < 75 years of age and 20 mg for patients ≥75 years of age, on Days 1, 8, 15, and 22) or pomalidomide and dexamethasone, and continued therapy until disease progression. The primary endpoint is progression-free survival (PFS), whereas key secondary endpoints include overall response rate and overall survival (OS). 307 pts were randomized and analyzed (ITT). At median follow-up of 11.6 months, median PFS was 11.5 months in the isatuximab arm vs 6.5 months in the control arm. PFS benefit was consistent across all major subgroups. Overall response rate (≥ partial response) was 60.4% for the isatuximab arm vs 35.3% in the control arm. VGPR rate or better was 31.8% in the experimental arm vs 8.5%, and MRD negativity

(evaluated with NGS, 10^{-5}) was seen in 5.2% in the isatuximab arm vs 0%. At analysis date, overall survival (OS) was immature (99 events) but a trend to OS improvement in isatuximab arm was observed (HR 0.687; 95% CI 0.461-1.023). Median treatment duration was 41 weeks for the experimental arm vs 24 weeks. Grade ≥3 AEs were observed in 86.8% in the experimental arm vs 70.5%; the % of pts that discontinued due to AEs is 7.2% in the isatuximab arm and 12.8% in the control arm and 7.9% of isatuximab arm and 9.4% pts in the control arm died due to AEs. IRs were reported in 38.2% of the experimental arm. Grade ≥3 infections were seen in 42.8% pts of isatuximab arm and 30.2% in the control arm, grade ≥3 neutropenia in 84.9% of pts treated with isatuximab and 70.1% of pts enrolled in the control arm. The addition of isatuximab to pomalidomide and low-dose dexamethasone significantly improved PFS and ORR with a manageable safety profile.

Based on the data above, the dose of isatuximab 10 mg/kg is the selected dose for combination therapies.

Isatuximab in combination with carfilzomib

One Investigator-sponsored Phase Ib study (TCD12795) is currently ongoing in US in patients with RRMM (NCT02332850) with the objective to determine the maximum tolerated dose of isatuximab when combined with carfilzomib. Three dose levels (DL)/schedules of isatuximab are being assessed: 10 mg/kg Q2W (DL1), 10 mg/kg QW/Q2W (DL2), and 20 mg/kg QW/Q2W (DL3). Isatuximab is combined to a fixed dose of carfilzomib 20 mg/m2 on Day 1 and Day 2 and then 27 mg/m2 on Days 8, 9, 15, and 16 on Cycle 1; twice weekly, 3 weeks out of 4 from Cycle 2 to 8 and twice every 2 weeks after Cycle 8. Dexamethasone 20 mg is given as premedication prior to first infusion of isatuximab, and then 10 to 20 mg prior to each infusion. In addition, dexamethasone 4 mg is given as premedication prior to each carfilzomib infusion at Cycle 1 and then is given at the Investigator discretion.

Recruitment is ongoing and preliminary data of this ongoing study were presented at American Society of Hematology meeting in 2016 (22), on the first 11 treated patients: 3, 3, and 5 patients treated at DL1, DL2, and DL3, respectively. The median number of prior lines was 4.5 (2 to 8), all patients were previously treated with lenalidomide, and 10 patients previously received bortezomib. Ten patients were refractory to their previous therapy. The median number of cycles administered was 6 (minimum [min]-maximum [max]: 1 to 20). No dose-limiting toxicity was reported. Infusion reaction was reported in 6 (54%) patients (4 patients with Grade 1 and 2 patients with Grade 2), and all infusions were completed. The other most frequent treatment-emergent adverse events (TEAEs) were dyspnea (45%), fatigue (45%), nausea (45%), pain (back, chest wall, and pelvis; 36%), peripheral neuropathy (36%), hypertension (27%), cough (27%), anorexia (27%), gastroesophageal reflux disease (18%), constipation (18%), diarrhea (18%), nasal congestion (18%), and hypokalemia (18%). Grade 3/4 non-hematological TEAEs were reported in fewer than 5% of patients. Hematological abnormalities were

mild; 9% Grade 3/4 anemia, 9% Grade 3/4 neutropenia, and 64% Grade 3/4 lymphopenia. The most frequent serious adverse event (SAE) was Grade 3 pneumonia (18%).

Interesting preliminary efficacy results were reported in 10 patients evaluable for response: 2 patients had very good partial response (VGPR), 6 patients had partial response (PR), 1 patient had minimal response (MR), and 1 patient had stable disease (SD).

The pharmacokinetics (PK) of isatuximab analyzed in 10 patients is not modified by the co-administration of carfilzomib (unpublished data).

Chari et al. (39) presented at ASCO 2018 preliminary results of the association of isatuximab, carfilzomib and dexamethasone in RRMM. The primary objective was to assess the maximum tolerated dose of isatuximab + carfilzomib in RRMM, and the secondary objectives were assessment of safety, PK, immunogenicity, and efficacy. A 3+3 dose escalation design was used. 3 dosing levels were tested: isatuximab 10 mg/kg Q2W, isatuximab 10 mg/kg QW for 4 cycles then Q2W and isatuximab 20 mg/kg QW for 4 cycles hen Q2W in combination with carfilzomib at the standard dose of 27 mg/m^2 and schedule. 15 pts were treated in the dose escalation and 18 pts were enrolled in the expansion cohort during the second dosing level, for a total of 33 pts treated. The median age was 61 yrs. Pts received a median of 3 prior lines and all pts were IMId and PI exposed. Median follow-up is 6.5 months, 29 pts are evaluable for response. The median number of study cycle was 3, the overall response rate (ORR) was equal to 66% and clinical benefit rate (CBR) is 86%. The median progression free survival has not been reached. No DLT or severe toxicity has been observed. Common adverse events (AEs-all grades, incidence ≥ 15%), were thrombocytopenia (66%), pain (60%), upper respiratory infection (56%), diarrhea (40%), fatigue (40%), anemia (33%), cough (33%), elevated creatinine (30%), nausea (30%), neutropenia (27%), headache (27%), dyspnea (16.7%) and fever (16.7%). Serious AEs occurred in 9 pts and < 5% of AEs were grade 3/4. Infusion reactions (IRs) were the most common isatuximab-related AE: 17 IRs (Grade 1 or 2) occurred in the 50% of pts. Results indicate that the combination of isatuximab with carfilzomib appears safe, since toxicity is consistent with the AEs of the individual agents with few grade 3/4 AEs.

In the ongoing phase II GMMG-CONCEPT trial (NCT03104842) in patients with primary diagnosed high-risk MM aiming to evaluate the MRD negativity after consolidation, isatuximab is administered in combination with carfilzomib, lenalidomide and dexamethasone. Carfilzomib doses are the following ones: for the first induction cycle 20 mg/m^2 in days 1-2, 36 mg/m^2 on days 8, 9, 15, 16, and 36 mg/m^2 on days 1, 2, 8, 9, 15 and 16 in cycles 2-6; for the consolidation phase 27 mg/m^2 on days 1, 2, 8, 9, 15 and 16 of cycle 1, increased to 36 mg/m^2 in the same days of cycles 2-4 and for the maintenance period carfilzomib is administered at the 70 mg/m^2 C1 in days 1 and 15. Results presented at IMW 2019 by Katja Weisel about the safety-run in phase show that the first 10 patients were enrolled to assess dose-limiting toxicities during the first two I-KRd cycles and contributed to the analysis. All patients experienced at least one treatment-emergent adverse event (TEAE), in total 49 TEAE were reported,

15 were classified as Grade 1, 14 as Grade 2, 17 as Grade 3 and 3 as Grade 4. Main ≥ Grade 3 toxicities were hematologic (neutropenia, leukopenia, lymphopenia, anemia and thrombocytopenia). Non-hematological toxicities Grade ≥ 3 were cerebral vascular disorders, self limiting ventricular tachycardia and diarrhea. 3 patients experienced infusion reaction grade 2 during the first Isatuximab infusion. In total, 5 SAE occurred. The 2 cerebral events were classified as non-related due to preexisting comorbidities. 90% of the patients completed 6 cycles of induction and all the patients had documented responses during induction phase, since all patients achieved ≥ VGPR. Results referred to the 4-drug combination of IKRd indicate that toxicity was manageable with an overall safety profile consistent with prior experience with KRd and anti-CD38 antibody treatment and that the preliminary response rates are encouraging(46).

4.3 Carfilzomib

Carfilzomib Background

Carfilzomib is a tetrapeptide epoxyketone proteasome inhibitor (PI) that binds selectively and irreversibly to the 20S proteasome, the proteolytic core particle within the 26S proteasome. Consequently, proteasome function after therapy can only be regained by de novo proteasome synthesis. Specifically, carfilzomib inhibits the chymotrypsin-like catalytic activity of the β5 subunit over the caspase-like catalytic activity of the β1 subunit or the trypsin-like catalytic activity of the β2 subunit, resulting in the accumulation of proteasome substrates and ultimately growth arrest and apoptosis (Hoy, 2016). Carfilzomib extensively penetrates all tissues, but the brain. It is metabolized largely extra-hepatically and rapidly cleared from the circulation by biliary and renal excretion (t1/2 = 15 to 30 minutes); < 1% is excreted intact (Kortuem and Stewart, 2013).

Carfilzomib is structurally and mechanistically distinct from the dipeptide boronic acid proteasome inhibitor bortezomib (Velcade®). In addition, when measured against a broad panel of proteases including metallo, aspartyl, and serine proteases, carfilzomib demonstrated less reactivity against non-proteasomal proteases when compared to bortezomib (5,6).

Carfilzomib entered clinical studies in September 2005. On 20 July 2012, Kyprolis® (carfilzomib for injection) was granted accelerated approval by the US Food and Drug Administration (FDA) for the treatment of patients with multiple myeloma who have received at least 2 prior therapies, including bortezomib and an immunomodulatory agents (IMiD), and have demonstrated disease progression on or within 60 days of completion of the last therapy. The initial accelerated approval was based on the results of the phase 2 PX-171-003-A1 study in the United States. Subsequent full approval in the United States and globally were based on 2 phase 3 studies: PX-171-009 ASPIRE and 2011-003 ENDEAVOR. Following these approvals, Kyprolis in combination with either lenalidomide and dexamethasone or dexamethasone alone is indicated for the treatment of RRMM. The exact indication wording varies by region. As of 19 July 2018, an estimated 4132 subjects have been treated with carfilzomib in company-

sponsored clinical studies since the beginning of the development program and approximately 88 964 patients have been exposed to carfilzomib in the post marketing setting.

Carfilzomib in Combination with Lenalidomide and Dexamethasone

Carfilzomib twice weekly in combination with lenalidomide and dexamethasone is approved for use in several regions and one of the standards of care for the treatment of relapsed/refractory MM. This is based on the results of the ASPIRE trial that showed prolonged PFS and OS with KRd vs Rd (23). Studies have explored this combination in newly diagnosed multiple myeloma (NDMM) in both the non-transplant setting and the pre (induction) transplant and post (consolidation) transplant settings.

A phase Ib/II study assessed the combination of carfilzomib with lenalidomide and low-dose dexamethasone as a frontline treatment for MM. After 4 cycles transplant-eligible candidates underwent stem cell collection. Results on efficacy and safety were promising (24), and provided the basis for testing the combination in a randomized large study.

The FORTE Study randomized 474 patients to receive KRd induction followed by ASCT and 4 cycles of KRd consolidation, vs KRd for 12 cycles, vs KCd induction followed by ASCT and 4 cycles of KCd consolidation. Patients randomized to KRd-ASCT-KRd achieved high rate of response (≥ VGPR 89%; ≥ CR 60%; sCR 44%); on ITT analysis, 58% of patients achieved MRD negativity (sensitivity 10^{-5}). Response rate and MRD negativity in the KRd-ASCT-KRd arm were significantly superior to the KCd-ASCT-KCd arm (45). In results updated(ASCO 2019) multivariate regression analysis showed that, KRd_ASCT_KRd vs KRd12 reduced the risk of early progression (OR 0.42; P=0.021); R-ISS Stage 2 (OR 3.6; P=0.001) and R-ISS Stage 3 (OR 4.85; P=0.003) increased the risk compared with R-ISS 1.

A dose of carfilzomib administered in the NDMM setting in combination with lenalidomide and dexamethasone of 36 mg/sqm twice weekly on days 1,2,8,9,15,16, is based on the results of a phase I/II study (24). The same dose proved to be well tolerated in other studies in the NDMM setting (12, 35). To improve the compliance of patients and the quality of life, a weekly schedule of carfilzomib in combination with lenalidomide and dexamethasone has been evaluated in a phase Ib study carfilzomib administered once a week only along with lenalidomide and dexamethasone in a group of patients with both relapsed and newly diagnosed multiple myeloma (Biran et al, ASH 2019). In this study patients received carfilzomib (30-minute infusion; 56 or 70 mg/m^2) on days 1, 8, and 15; lenalidomide 25 mg on days 1-21; and dexamethasone 40 mg on days 1, 8, 15, and 22 (day 22 omitted for cycles 9+) of 28-day cycles (41). Primary objective was safety/tolerability; efficacy was a secondary objective. 22 patients were enrolled during dose evaluation (56-mg/m^2 , n = 10; 70-mg/m^2 , n = 12) and 34 during dose expansion (all initiated dosing at 70 mg/m^2). After 2 fatal adverse events (AEs) during 70-mg/m^2 dose expansion, dosage reduction to 56 mg/m^2 was permitted. Results are referred to carfilzomib 56-mg/m^2 (n = 10) and 70-mg/m^2 groups (dose evaluation/expansion; n = 46). Median carfilzomib dose was 53.2 mg/m^2 (56-mg/m^2 group) and 62.4 mg/m^2 (70-mg/m^2 group). Grade ≥3 AE rates were 70.0% (56 mg/m^2) and 69.6% (70 mg/m^2). Similar response rates were shown with carfilzomib 56 mg/sqm (90%) days 1,8,15 and 70 mg/sqm (89.1%).

These data provided the rationale for selecting 56 mg/sqm days 1,8,15 as the Carfilzomib dose going forwards in combination with lenalidomide 25 mg days 1-21 and weekly dexamethasone 40 mg days 1,8,15,22 during the induction and consolidation phase.

Richez et al. (42) evaluated KRd weekly regimen in 28 early RRMM patients. Patients received carfilzomib (30-minute infusion on days 1,8,15, starting dose, 20mg/m^2 on day 1 of cycle 1; target dose, 56mg/m^2 thereafter), lenalidomide (25mg PO days 1-21) and dexamethasone (40mg PO days 1, 8, 15 and 22) until progression disease or until occurrence of unacceptable toxic effects. With a median follow up of 8 months, 3 pts relapsed, and one patient died. The median number of KRd cycles administered was 6.5. Overall response rate was 93%, with 89% ≥VGPR and 61% ≥CR. The mean time to a response was 1.6 months. The median TTP and OS at 12 months were 89% and 95%, respectively. 29% of pts have discontinued treatment, with solely 50% due to adverse events (AEs). Hematologic AEs ≥ grade 3 were reported in 57% and non hematologic AEs ≥ grade 3 in 36%. No pts died related to AEs. Overall AEs ≥ grade 3 seen in ≥10% of pts was neutropenia, thrombocytopenia, vomiting and pyrexia. No pts experienced any severe cardiovascular AEs, including cardiac failure or any severe cardiac issues or thromboembolic events. Results indicate that KRd weekly at 20/56mg/m^2 is effective and safe to early RRMM pts.

The tolerability and efficacy of daratumumab in combination with KRd in newly diagnosed MM pts was examined in the MMY1001trial. Newly diagnosed pts regardless of transplantation eligibility were enrolled. Pts received daratumumab 16 mg/kg QW for Cycles 1-2, Q2W for Cycles 3-6, and Q4W thereafter. Carfilzomib (K) was administered on Days 1, 8 and 15 of each 28-day cycle (20 mg/m^2 on C1D1, 36 or 70 mg/m^2 subsequently based on tolerability of first dose) for ≤13 cycles or elective discontinuation for ASCT. Lenalidomide 25 mg was given on Days 1-21 and dexamethasone 20-40 mg per week. Twenty-two pts with a median age of 60 years old were enrolled and received a median of 8 treatment cycles. Nineteen pts escalated K dose to 70 mg/m^2 by C1D15. Median duration of follow-up was 7.4 months. 27% pts discontinued treatment; SAEs occurred in 46% of pts, and 14% were possibly related to daratumumab; 82% experienced a grade 3/4 TEAE. The most common grade 3/4 TEAEs (>10%) were lymphopenia and neutropenia; 5% cardiac grade 3 TEAE was observed (congestive heart failure) which resolved; pt quickly resumed study treatment with reduced K dose. No grade 5 TEAE was reported. All daratumumab associated infusion reactions (which occurred in 27% of pts) were grade ≤2. Treatment with daratumumab-KRd yielded an ORR (≥partial response) of 100% (5% complete response, 86% ≥very good partial response) in 21 response-evaluable pts. The 6-month PFS rate was 100%. In conclusion, the addition of daratumumab to KRd was well tolerated; the overall safety profile was consistent with that previously reported for KRd, with no additional toxicity observed with the addition of daratumumab. Deep and durable responses were observed. These data support further investigation of DARA-KRd as a frontline treatment regimen (47).

Costa et al. at ASH 2019 presented data from the MASTER trial - Monoclonal Antibody-Based Sequential Therapy for Deep Remission in Multiple Myeloma (48). Treatment cycles consisted of daratumumab 16 mg/kg IV days 1,8,15,22, carfilzomib 56 mg/m^2 IV days 1,8,15, lenalidomide 25 mg PO days 1-21 and dexamethasone 40 mg PO/IV days 1,8,15,22 repeated every 28 days. Patients received 4 cycles of Dara-KRd as induction, autologous transplantation, and received 0, 4 or 8 cycles of Dara-KRd consolidation, according to MRD status at each phase of therapy. MRD was evaluated by NGS-MRD at end of induction, post-transplant, and during each 4-cycle block of Dara-KRd consolidation. Primary endpoint was achievement of MRD negative remission (<10^{-5}) as defined by IMWG consensus. Secondary endpoints included MRD <10^{-6}, complete response (CR) by IMWG criteria at end of induction and upon completion of consolidation, and rate of imaging (assessed by PET/CT scan) plus MRD-negative CR. Patients received therapy until achievement of two consecutive MRD reads <10^{-5}. Confirmed MRD-negative pts received no further therapy and were observed with surveillance for MRD resurgence 6 and 18 months after treatment discontinuation. Patients completing consolidation without confirmed MRD-negative remission received standard lenalidomide maintenance. Currently 69 pts have been enrolled, 38 have completed induction and 22 have completed post-transplant assessment. Median age was 61 years, 19% had ISS 3, and 29% had high-risk chromosomal abnormalities [del17p, t(4;14) or t(14;16)]. All patients responded by end of induction cycle 2, 92% of pts obtained VGPR or better after induction and 91% of patients who have reached transplant obtained CR/sCR as best response on therapy. MRD-negative remission (<10^{-5}) rate was 34%, 70% and 80% after induction, transplant and at best response respectively. Rates of MRD <10^{-6} were 28%, 45% and 65% respectively. No patient discontinued therapy due to toxicity. Most common grade 3 and 4 AEs were neutropenia, infection, insomnia, hyperglycemia and rash. There were 15 serious AEs including pneumonia, fever and neutropenia, pulmonary embolism, and atypical hemolytic uremic syndrome. All 11 patients who have achieved confirmed MRD-negative remission and discontinued therapy also achieved imaging plus MRD-negative CR and none had relapse or resurgence of MRD with short follow up (0.8-7.3 months). Results indicate that Dara-KRd induction, autologous transplant and Dara-KRd consolidation guided by MRD is feasible, safe and leads to high proportion of patients achieving CR/sCR, IMWG MRD-negative CR, imaging plus MRD-negative CR and MRD <10^{-6}. This approach can form the basis for clinical efforts to reduce the burden of continuous therapy in those with confirmed MRD-negative remissions.

Landgren et al. presented at ASH 2019 data from the treatment of NDMM with daratumumab and KRd weekly or biweekly. The treatment schedule of weekly carfilzomib (41 pts) is the following one: 8 cycles of treatment; 28-day cycles with carfilzomib 20/56 mg/m^2 days 1, 8, and 15; lenalidomide 25 mg days 1-21; dexamethasone 40 mg weekly cycles 1-4, 20 mg after cycle 4; and daratumumab 16 mg/kg days 1, 8, 15, and 22 cycles 1-2, days 1 and 15 cycles 3-6, and day 1 cycles 7-8. The bi-weekly carfilzomib (N=41): 8 cycles of treatment; 28-day cycles with carfilzomib 20/36 mg/m2 days

1, 2, 8, 9, 15 and 16; lenalidomide, dexamethasone, and daratumumab are given at the same doses/schedules as the weekly cohort. For fit patients, stem cell collection is recommended after 4 to 6 cycles of therapy; Dara-KRd therapy is resumed after collection to a total of 8 cycles Dara-KRd. Currently, 29 patients meeting eligibility criteria were enrolled. Baseline characteristics include median age 59 years, 41% patients had high-risk FISH/SNP signature defined as one or more of the following: 1q+, t(4;14), t(14;16), t(14;20), and 17p-. 28 patients have completed one or more cycles weekly Dara-KRd; among these, 10 patients have completed therapy. The median number of cycles delivered is currently 6. Seven of the 10 patients who have completed study treatment are MRD negative. So far, additional 8 patients have become MRD negative while on therapy. Thus, among patients treated on the weekly cohort and who were evaluable for the MRD primary endpoint at this analysis, we found 83% to be MRD negative. The biweekly carfilzomib cohort shows similar results to the weekly cohort and no added major clinical toxicities. Therefore with a comparable efficacy and safety profile coupled with a substantial reduction of the number of infusions (total of 51 vs 27 infusions with bKRd-D vs wKRd-D, respectively), we conclude that the weekly dosing may offer an attractive treatment modality for newly diagnosed multiple myeloma patients (49).

4.4 Rationale of the study

The VRD combination is widely used as induction therapy before ASCT and endorsed in current ESMO guidelines (4). VRD has been used also as post transplant consolidation in 2 trials, one of them showing a benefit in terms of PFS in patients receiving VRD consolidation vs no consolidation (27). Carfilzomib, a second generation PI has demonstrated superior efficacy in the upfront newly diagnosed myeloma setting, for both PFS and OS (9). Building on this data, trials have been undertaken to evaluate Carfilzomib efficacy in the upfront newly diagnosed setting. Carfilzomib in combination with Lenalidomide and Dexamethasone (KRd) in the FORTE study given for 4 cycles of induction followed by ASCT and 4 cycles of consolidation delivered high response rates and in particular high rate of MRD negativity (12). Based on these assumptions we designed a phase III study for newly diagnosed ASCT eligible patients treated, in the control arm, with 4 cycles of KRd induction, ASCT and 4 cycles of KRd consolidation post ASCT. Data in the relapse setting showed that prolonged Carfilzomib therapy is safe and effective (9, 23). Data from phase I/II study in the upfront setting (24) showed similar findings. Therefore, to maximize efficacy, in the trial we include prolonged carfilzomib therapy (light consolidation) for 1 year, after the post ASCT consolidation KRd therapy. Carfilzomib is administered intravenously, with a standard schedule consisting on infusions twice a week for 3 consecutive weeks. To improve patient compliance and quality of life, once weekly administration of carfilzomib was evaluated in the relapse and in the newly diagnosed setting, proving to be efficacious and safe (26). Based on these data, a once weekly infusion of carfilzomib at a dose of 56 mg/sqm in the induction, post ASCT consolidation phases, and in the light consolidation phase, were included.

Anti CD38 MoAbs showed to be able to improve PFS and OS in association with all the combinations including standards of care evaluated so far, with a very good safety profile. Therefore, in the experimental arm of the trial, anti CD38 MoAb Isatuximab was added to KRd induction consolidation and KRd light consolidation to further improve outcome.

5. Study objectives

5.1 Primary Objective

To compare rate of Minimal Residual Disease (MRD) negativity by NGS between Isa-KRd and KRd in post ASCT consolidation treatment.

5.2 Secondary Objectives

Key secondary objectives

- Rate of MRD negativity after induction by NGS
- To compare progression-free survival (PFS) in the 2 treatment arms.

Other secondary objectives

- Rate of MRD negativity after light consolidation by NGS;
- Rate of 1 year sustained MRD negativity by NGS (from post ASCT consolidation to post light consolidation);
- Determine the overall response rate (ORR), VGPR, CR, sCR rate after induction, ASCT, post ASCT consolidation, light consolidation in the 2 treatment arms;
- Determine the rate of MRD negativity (by NGS) after ASCT;
- Determine the rate of MRD negativity (by NGF) after induction, ASCT, post ASCT consolidation and light consolidation;
- Determine the duration of response (DOR) in the 2 treatment arms;
- Determine the duration of MRD negativity (by NGS and NGF);
- Determine the rate of sustained for 1-year MRD negativity (by NGF) (from post ASCT consolidation to post light consolidation);
- Determine the time to progression (TTP) in the 2 treatment arms;
- Determine the overall survival (OS) in the 2 treatment arms;
- Determine the time to next therapy (TNT) in the 2 treatment arms;
- Determine the progression-free survival 2 (PFS2) in the 2 treatment arms;
- Determine whether tumor response and outcome may change in subgroups with different prognosis according to current prognostic factors;
- Determine safety in the 2 treatment arms;
- Determine the success of stem cell harvest;
- Determine the success of engraftment after ASCT;
- Quality of life.

6. Study design

This is a open-label randomized phase III study that enrolls newly diagnosed MM patient eligible for high-dose chemotherapy and ASCT. Patients will be randomized at enrolment (1:1, stratification according to ISS Stage [3 levels: I vs II vs III] and cytogenetic risk FISH [2 levels: high-risk vs standard risk/missing] based on presence of t(4;14), t(14;16), and/or del 17p)) into 2 treatment arms: -ARM A: induction with 4 cycles of Isatuximab-Carfilzomib-Lenalidomide-dexamethasone (Isa-KRd) followed by cyclophophamide and stem cell collections, chemotherapy with Melphalan 200 mg/m^2 followed by ASCT (Mel200-ASCT), 4 cycles of Isa-KRd post ASCT consolidation and 12 cycles of Isatuximab-Lenalidomide-Carfilzomib-dexamethasone (IsaKRd) light consolidation; ARM B: induction with 4 cycles of Carfilzomib-Lenalidomide-dexamethasone (KRd) followed by cyclophophamide and stem cell collections, chemotherapy with Melphalan 200 mg/m^2 followed by ASCT (Mel200-ASCT), 4 cycles of KRd post ASCT consolidation and 12 cycles of Carfilzomib-Lenalidomide-dexamethasone (KRd) light consolidation. Details of all treatments (dose and schedule) are given in paragraph 8. After light consolidation patients are allowed to receive Lenalidomide maintenance as per standard of care.

7. Study population

7.1 Eligibility for registration/randomization

Patients with newly diagnosed multiple myeloma, eligible for high dose chemotherapy and ASCT will be enrolled. All patients must be registered/randomized before start of treatment and must meet all of the following eligibility criteria.

Inclusion criteria

1. Patient with newly diagnosed multiple myeloma and eligible to ASCT, for whom the standard treatment it is not, according to investigator, the best treatment available.
2. Patient is, in the investigator’s opinion, willing and able to comply with the study visits and procedures required per protocol.
3. Patient has provided written informed consent in accordance with federal, local, and institutional guidelines prior to initiation of any study-specific activities or procedures. Subject does not have kind of condition that, in the opinion of the Investigator, may compromise the ability of the subject to give written informed consent and patient is, in the investigator(s) opinion, willing and able to comply with the protocol requirements.
4. Monoclonal plasma cells in the bone marrow ≥10% or presence of a biopsy proven plasmacytoma and documented multiple myeloma satisfying at least one of the calcium, renal, anemia, bone (CRAB) criteria or biomarkers of malignancy criteria:

   <u>CRAB criteria:</u>

- Hypercalcemia: serum calcium >0.25 mmol/L (>1 mg/dL) higher than upper limit of normal (ULN) or >2.75 mmol/L (>11 mg/dL)
- Renal insufficiency: creatinine clearance <40mL/min or serum creatinine >177 µmol/L (>2 mg/dL)
- Anemia: hemoglobin >2 g/dL below the lower limit of normal or hemoglobin <10 g/dL
- Bone lesions: one or more osteolytic lesions on skeletal radiography, CT, or PET-CT

Biomarkers of Malignancy:

- Clonal bone marrow plasma cell percentage ≥60%
- Involved: uninvolved serum FLC ratio ≥100
- >1 focal lesion on magnetic resonance imaging (MRI) studies

5. Patient is 18 - 70 years old and is eligible for autologous stem cell transplantation
6. Patient has measurable disease as defined by any one of the following:

- Serum monoclonal paraprotein (M-protein) level ≥1.0 g/dL or urine M-protein level ≥200 mg/24 hours; or
- Light chain multiple myeloma without measurable disease in the serum or the urine: Serum immunoglobulin FLC ≥10 mg/dL and abnormal serum immunoglobulin kappa lambda FLC ratio.

7. Life expectancy ≥ 3 months
8. ECOG status ≤2
9. Clinical laboratory values meeting the following criteria during the Screening Phase:
   - Adequate hepatic function, with serum (alanine aminotransferase) ALT ≤ 2.5 times the upper limit of normal (ULN), AST (aspartate transaminase) ≤ 2.5 x the ULN
   - Serum direct bilirubin ≤ 1.5 ULN) (except in subjects with congenital bilirubinemia, such as Gilbert syndrome, direct bilirubinemia ≤ 1.5 ULN)
   - Absolute neutrophil count (ANC) ≥ 1.0 × 10^9/L
   - Platelet count ≥ 75× 10^9/L (≥ 50× 10^9/L if myeloma involvement in the bone marrow is > 50%) and no platelet infusion in the 1 week prior to screening platelet count
   - Creatinine clearance (CrCl) ≥ 30 mL/minute. Creatinine clearance should be calculated using eGFR (Modified Diet in Renal Disease [MDRD])
   - Corrected serum calcium ≤ 13.5 mg/dL (3.4 mmol/L)
   - LVEF ≥ 40%. 2-D transthoracic echocardiogram (ECHO) is the preferred method of evaluation. Multigated Acquisition Scan (MUGA) is acceptable if ECHO is not available.
10. Females of childbearing potential (FCBP)* complies with the conditions of the Pregnancy Prevention Plan, including confirmation that she has an adequate level of understanding and must agree to ongoing pregnancy testing and to practice contraception or true abstinence. FCBP must use a highly effective and an additional barrier contraception method simultaneously for 4 weeks before starting therapy, during treatment and dose interruptions and for 5 months after the last dose of study drugs.

11. Male subjects must agree to practice contraception if sexually active with FCBP during the treatment and for at least 5 months after the last dose of study drugs. Males must agree to refrain from donating sperm for at least 90 days after the last dose of carfilzomib and for at least 5 months after the last dose of isatuximab.

*Note 1: a FCBP is a woman who:

1) has achieved menarche at some time point,

2) has not undergone a hysterectomy or bilateral oophorectomy or,

3) has not been naturally postmenopausal (amenorrhea following cancer therapy does not rule out childbearing potential) for at least 24 consecutive months (ie, has had menses at any time in the preceding 24 consecutive months).

Note 2: true abstinence is acceptable when this is in line with the preferred and usual lifestyle of the patient. Periodic abstinence (eg, calendar, ovulation, symptothermal, post-ovulation methods) and withdrawal are not acceptable methods of contraception.

Exclusion criteria

1. Previous treatment with anti-myeloma therapy (does not include radiotherapy, bisphosphonates, or a single short course of steroid ≤ to the equivalent of dexamethasone 40 mg/day for 4 days).
2. Patients with non-secretory MM unless serum free light chains are present and the ratio is abnormal or a plasmacytoma with minimum largest diameters of > 2 cm.
3. Patients with plasma cell leukemia, amyloidosis, Waldenstrom Disease, POEMS syndrome
4. Meningeal involvement of multiple myeloma
5. Patient ineligible for autologous transplantation
6. Pregnant or lactating females
7. Acute active infection requiring treatment (systemic antibiotics, antivirals, or antifungals) within 14 days prior to randomization
8. Known human immunodeficiency virus infection (HIV)
9. Active hepatitis A, B or C infection. Hepatitis C infection (subjects with hepatitis C that achieve a sustained virologic response after antiviral therapy are allowed), or hepatitis B infection (subjects with hepatitis B surface antigen or core antibody that achieve sustained virologic response with antiviral therapy are allowed). Tests to be performed if required per local country regulations (in Czech Republic testing for HIV and hepatitis B and C is required at screening). In fact it is not possible to avoid the risk of virological reactivation with the study treatments.
   Uncontrolled or active HBV infection: Patients with positive HBsAg and/or HBV DNA
   - Patient can be eligible if anti-HBc IgG positive (with or without positive anti-HBs) but HBsAg and HBV DNA are negative. If anti-HBV therapy in relation with prior infection was started before initiation of IMP, the anti-HBV therapy and monitoring should continue throughout the study treatment period.

- Patients with negative HBsAg and positive HBV DNA observed during screening period will be evaluated by a specialist for start of anti-viral treatment: study treatment could be proposed if HBV DNA becomes negative and all the other study criteria are still met.

Active HCV infection: positive HCV RNA and negative anti-HCV

- Patients with antiviral therapy for HCV started before initiation of IMP and positive HCV antibodies are eligible. The antiviral therapy for HCV should continue throughout the treatment period until seroconversion.

- Patients with positive anti-HCV and undetectable HCV RNA without antiviral therapy for HCV are eligible.

10. Unstable angina or myocardial infarction within 4 months prior to randomization, NYHA Class III or IV heart failure, uncontrolled angina, uncontrolled hypertension, (Uncontrolled hypertension, defined as an average systolic blood pressure ≥ 160 mmHg or diastolic ≥ 100 mmHg despite optimal treatment (measured following European Society of Hypertension/European Society of Cardiology 2013 guidelines), in the last 5 years pulmonary embolia, history of severe coronary artery disease, severe uncontrolled ventricular arrhythmias, sick sinus syndrome, or electrocardiographic evidence of acute ischemia or Grade 3 conduction system abnormalities unless subject has a pacemaker
11. Non-hematologic or hematologic malignancy within the past 3 years with the exception of a) adequately treated basal cell carcinoma, squamous cell skin cancer, or thyroid cancer; b) carcinoma in situ of the cervix or breast; c) prostate cancer of Gleason Grade 6 or less with stable prostate-specific antigen levels; or d) cancer considered cured by surgical resection or unlikely to impact survival during the duration of the study, such as localized transitional cell carcinoma of the bladder or benign tumors of the adrenal or pancreas
12. Significant neuropathy (Grades 3–4, or Grade 2 with pain) within 14 days prior to randomization as defined by National Cancer Institute Common Toxicity Criteria (NCI CTCAE) 5.0
13. Known history of allergy to Captisol® (a cyclodextrin derivative used to solubilize carfilzomib) and to PS80; prior hypersensitivity to sucrose, histidine (as base and hydrochloride salt), or known intolerance or hypersensitivity to infused protein products or any of the components (active substance or excipients) of study treatments that are not amenable to premedication with steroids, or H2 blockers, that would prohibit further treatment with these agents.
14. Contraindication to any of the required concomitant drugs or supportive treatments, including hypersensitivity to all anticoagulation and antiplatelet options, antiviral drugs, or intolerance to hydration due to preexisting pulmonary or cardiac impairment
15. Any other clinically significant medical disease or condition that, in the Investigator’s opinion, may interfere with protocol adherence or a subject’s ability to give informed consent
16. Received any investigational drug within 14 days or 5 half-lives of the investigational drug, prior to initiation of study intervention, whichever is longer.

17. Pregnant or breastfeeding woman or woman who intends to become pregnant during the participation in the study. FCBP unwilling to prevent pregnancy by the use of 2 reliable methods of contraception for ≥4 weeks before the start of study treatment, during treatment (including dose interruptions), and for at least 28 days following discontinuation of study lenalidomide, or 30 days following discontinuation of carfilzomib or for 5 months after discontinuation of isatuximab treatment, whichever occurs last,
18. Male participants who disagree to practice true abstinence or disagree to use a condom during sexual contact with a pregnant woman or a FCBP while participating in the study, during dose interruptions, and for at least 28 days following discontinuation of study lenalidomide, or 3 months following discontinuation of carfilzomib, or for 5 months after discontinuation of isatuximab treatment, whichever occurs last, even if he has undergone a successful vasectomy.

8. Treatment

8.1 Arm A

8.1.1 Induction schedule

Table 1: Induction phase dose and schedule in the Isa KRd arm

Agent	Dose/day	Route of administration	Days
Isatuximab	10 mg/kg	IV	1, 8, 15, and 22 Cycle 1; days 1 and 15 Cycles 2 to 4.
Carfilzomib	20 mg/m^2	IV	1 cycle 1
Carfilzomib	56 mg/m^2	IV	8, 15 cycle 1 and on days 1, 8, 15 for cycles 2-4
Lenalidomide	25 mg	OS	1-21
Dexamethasone	40 mg	OS/IV	1, 8, 15, 22

Repeat for 4 28-day cycles of induction

8.1.2 Transplant (as per Standard of Care)

All patients will be given Cyclophosphamide at the dose of 2 to 3 g/m^2, followed by G-CSF for stem cell collection, or other treatment according to local protocols.

Cyclophosphamide will start 4-6 weeks after day 21 of cycle 4.

Stem cell collection will be performed as soon as CD34+ cells are present in peripheral blood, which is usually between 9-14 days after first day of Cyclophosphamide. Stem cells will be harvested at a minimum of 4 x 10^6 CD34+ cells/kg and cryopreserved. A second course of mobilization with

Cyclophosphamide or other agents will be performed as per local clinical practice. Plerixafor is allowed according to local standard of care.

Subjects will receive melphalan 200 mg/m^2 as conditioning therapy, according to the standard of care. Melphalan may be given at a lower dose of 140mg/m^2, per institutional standards, if the subject has renal insufficiency.

Engraftment after ASCT will be evaluated according to the following definition:

Absolute Neutrophil Count (ANC): 3 consecutive days with at least ANC 0.5 109/L (the last day)

Platelet count: 7 consecutive days with at least Platelet count 20 109/L without transfusion or 3 consecutive days with at least Platelet count 50 109/L without transfusion (the last day).

8.1.3 Consolidation schedule

Table 2: Consolidation phase dose and schedule in the Isa KRd arm

**Agent**	**Dose/day**	**Route of administration**	**Days**
Isatuximab	10 mg/kg	IV	1, 15
Carfilzomib	56 mg/m^2	IV	1, 8, 15
Lenalidomide	25 mg	OS	1-21
Dexamethasone	40 mg	OS/IV	1, 8, 15, 22

Repeat for 4 28-day cycles of consolidation.

Consolidation will start 6-8 weeks after autologous transplant.

8.1.4 Light consolidation schedule

Table 3: Light consolidation phase dose and schedule in the Isa KRd arm

**Agent**	**Dose/day**	**Route of administration**	**Days**
Isatuximab	10 mg/kg	IV	1
Carfilzomib	56 mg/m^2	IV	1, 15
Lenalidomide	10 mg	OS	1-21
Dexamethasone	20 mg	OS/IV	1, 15

Repeat for 12 28-day cycles.

After light consolidation physicians are advised to continue with lenalidomide maintenance as per standard of care, since the current standard of care after ASCT with or without consolidation is lenalidomide maintenance.

8.1.5 Dose adjustments

Refer to paragraph 8.2.5

8.1.6 Special precautions and supportive care

Refer to paragraph 8.4

8.2 Arm B

8.2.1 Induction schedule

Table 4: Induction phase dose and schedule in the KRd arm

Agent	Dose/day	Route of administration	Days
Carfilzomib	20 mg/m^2	IV	1 cycle 1
Carfilzomib	56 mg/m^2	IV	8, 15 cycle 1 and on days 1, 8, 15 for cycles 2-4
Lenalidomide	25 mg	OS	1-21
Dexamethasone	40 mg	OS/IV	1, 8, 15, 22

Repeat for 4 28-day cycles of induction

8.2.2 Transplant (as per Standard of Care)

All patients will be given Cyclophosphamide at the dose of 2 to 3 g/m^2, followed by G-CSF for stem cell collection, or other treatment according to local protocols. Cyclophosphamide will start 4-6 weeks after day 21 of cycle 4.

Stem cell collection will be performed as soon as CD34+ cells are present in peripheral blood, which is usually between 9-14 days after first day of Cyclophosphamide. Stem cells will be harvested at a minimum of 4 x 10^6 CD34+ cells/kg and cryopreserved, as per institutional guidelines. A second course of mobilization with Cyclophosphamide or other agents as per local clinical practice. Plerixafor is allowed according to local standard of care.

Subjects will receive melphalan 200 mg/m^2 as conditioning therapy, according to the standard of care. Melphalan may be given at a lower dose of 140mg/m^2, per institutional standards, if the subject has renal insufficiency.

Engraftment after ASCT will be evaluated according to the following definition:

Absolute Neutrophil Count (ANC): 3 consecutive days with at least ANC 0.5 10^9/L (the last day)

Platelet count: 7 consecutive days with at least Platelet count 20 10^9/L without transfusion or 3 consecutive days with at least Platelet count 50 10^9/L without transfusion (the last day).

8.2.3 Consolidation schedule

Table 5: Consolidation phase dose and schedule in the KRd arm

Agent	Dose/day	Route of administration	Days
Carfilzomib	56 mg/m^2	IV	1, 8, 15
Lenalidomide	25 mg	OS	1-21
Dexamethasone	40 mg	OS/IV	1, 8, 15, 22

Repeat for 4 28-day cycles of consolidation.
Consolidation will start 6-8 weeks after autologous transplant.

8.2.4 Light consolidation schedule

Table 6: Light consolidation phase dose and schedule in the KRd arm

Agent	Dose/day	Route of administration	Days
Carfilzomib	56 mg/m^2	IV	1, 15
Lenalidomide	10 mg	OS	1-21
Dexamethasone	20 mg	OS/IV	1, 15

Repeat for 12 28-day cycles.
After light consolidation physicians are advised to continue with lenalidomide maintenance as per standard of care, since the current standard of care after ASCT with or without consolidation is lenalidomide maintenance.

8.2.5 Dose adjustments

No dose reductions for Isatuximab will be permitted.
The following dose reductions will be allowed for Lenalidomide, Carfilzomib and dexamethasone.

Dose levels

Table 7: Dose reductions levels for carfilzomib, lenalidomide and dexamethasone

	Lenalidomide	Carfilzomib	Dexamethasone
Baseline dose	25 mg daily<br>In case of renal impairment:<br>- Moderate (CrCl 30-49 ml/min): 10 mg daily;<br>- Severe (CrCl<30 ml/min without dialysis): 15 mg every 48 hrs;	56 mg/m^2 (20 mg/m^2 on Cycle 1, Day 1)<br>Baseline dose in case of moderate/severe hepatic impairment: 15 mg/m^2	40 mg

	- End stage (CrCl <30 ml/min and dialysis): 5 mg daily		
One level dose reduction	20 mg daily<br>In case of renal impairment:<br>- Moderate (CrCl 30-49 ml/min): 5 mg daily;<br>- Severe (CrCl<30 ml/min without dialysis): 5 mg every 48 hrs;<br>- End stage (CrCl <30 ml/min and dialysis): 5 mg every 48 hrs	42 mg/m^2	20 mg
Two level dose reduction	15 mg daily	36 mg/m^2	10 mg
Three level dose reduction	10 mg daily	27 mg/m^2	4 mg
Four level dose reduction	5 mg daily		0

Patients may have isatuximab/carfilzomib/dexamethasone dose omission within a cycle if toxicity occurs and does not recover or returns to Grade 1 on the day of planned infusion or within the following day for the weekly administration, or within the following 3 days for every 2 weeks and 4 weeks administrations. Within a cycle, these delays in cases of unresolved toxicity at the time of planned re-administration are permitted, otherwise infusion is omitted. Patients will receive the next infusion after recovering from the toxicity. No dose reductions for isatuximab will be permitted. Carfilzomib and Dexamethasone may be reduced according to toxicity (see table 7).

Lenalidomide may be stopped in case of toxicity, and resume when the toxicity resolves. If a patient has to stop lenalidomide during a cycle, patient can resume treatment when the toxicity resolves. Lenalidomide treatment will be stopped at day 21 of each given cycle, regardless of temporary stop during cycle or not (example: patient experiences a toxicity on day 7, and according to the toxicity patient has to stop lenalidomide on day 7. In case of toxicity that resolves at day 11, patients will continue treatment from day 11 and stop anyway on day 21).

Starting Dose in Hepatic Insufficiency

For subjects with baseline chronic hepatic impairment (mild, moderate), reduce the starting and subsequent doses of carfilzomib by 25% (Brown et al, 2017):15 mg/m^2 day 1 of cycle 1 and 42 mg/m^2 day 8 of cycle 1 and thereafter.

If hepatic function returns to normal, the dose may be re-escalated to 56 mg/m^2.

Starting Dose in Renal Impairment

In case of moderate renal impairment (CrCl between 30-49 mL/min) lenalidomide should be given at the dose of 10 mg on days 1-21 during cycles 1-8 and at the dose of 5 mg on days 1-21 during light-consolidation.

In case of severe renal impairment (CrCl <30 mL/min without dialysis) lenalidomide should be given at the dose of 15 mg every 48 hours during cycles 1-8 and at the dose of ~~10~~5 mg every 48 hours on days 1-21 during light-consolidation.

In case of end-stage renal disease (CrCl <30 mL/min and dialysis) lenalidomide should be administered at a dose of 5 mg on days 1-21 in cycle 1-8 and at the dose of 5 mg every 48 hours during light consolidation. On dialysis days, administer lenalidomide after dialysis.

8.2.6 Recommended actions for adverse events

In the table below major AE and recommended actions are summarized. In case AE correlation with one specific drug is not clear, after resolution of the previous toxicity patients may start treatment with one of the suspected drugs, and the other one can be added subsequently, to better discriminate drug relation and avoid stopping all drugs if correlation is not clear. Patients are allowed to stop one or more drugs and continue with the others.

Table 8: Recommended actions for adverse events study drug/s related:

Body System	NCI-CTC Adverse Event and or Symptom and Category	Isatuximab	Carfilzomib	Lenalidomide	Dexamethasone
**Allergic reactions**	Allergic reaction or hypersensitivity Grade 2 OR 3	Hold all therapy.<br>If the toxicity resolves to ≤ Grade 1, restart isa-KRd. Reduce by 1 dose-level the suspected medication(s) AND implement appropriate anti-allergic prophylaxis therapy.<br>If the reaction was anaphylactic in nature, do not resume Isa-KRd.<br>NOTE: If the reaction was cutaneous in nature, refer to the cutaneous category below.			
	Allergic reaction or hypersensitivity Grade 4	Discontinue Isa-KRd.			
**Cardiovascular**	Fluid Retention (ie, edema defined as >30% inter-limb discrepancy in volume, gross deviation from normal anatomic contour, limiting self care ADL) >Grade 3 (limiting function and unresponsive to therapy or anasarca)				Administer diuretics as needed, and decrease dexamethasone dose by 1 dose-level; if edema persists despite above measures, decrease dose another dose-level. Permanently discontinue dexamethasone if symptoms persist despite second dose reduction.
	Congestive Heart Failure ≥ Grade 3 (Decreased LVEF <39-20% with drop greater or equal than 20% from baseline)	Fall in LVEF at Day 1 of cycle: Delay Day 1 administration until LVEF returns to >40%, or if held due to a drop to >55% until returns to within 15% of baseline and resume full dose isatuximab, lenalidomide and dexamethasone at the same dose level, and based on benefit risk assessment to re-start carfilzomib with dose reduced by 1 dose level or to stop carfilzomib.<br>Fall in LVEF within cycle: Hold study treatment until returns to >40%, or until returns to within 15% of baseline and resume full dose isatuximab, lenalidomide and dexamethasone at the same dose level and based on benefit risk assessment to re-start carfilzomib with dose reduced by 1 dose level or to stop carfilzomib.<br>If delay is >3 days, omit the dose.<br>Discontinue carfilzomib and/or lenalidomide (according to the suspect drug correlation) if recurrence despite 1 dose reduction.<br>Onset of clinical congestive heart failure: any subject with congestive heart failure, whether or not drug related, must have the dose held until resolution or return to baseline. Appropriate medical management should be initiated. If no resolution after 4 weeks, carfilzomib will be permanently discontinued.<br>< Grade 3 Once congestive heart failure resolves or returns to baseline, resume at full dose.<br>Grade ≥ 3 Once congestive heart failure resolves or returns to baseline, treatment may continue at one dose level reduction			
	Myocardial infarction	Discontinue Isatuximab, carfilzomib and/or lenalidomide<br>Day 1 of cycle: Delay Day 1 administration until recovery, resume full isatuximab dose at the same dose level if and when clinically appropriate.<br>Within cycle: After recovery, resume full isatuximab dose at the same dose level if and when clinically appropriate.<br>If associated with carfilzomib or lenalidomide consider permanent discontinuation of the responsible drug, or resume only after full recovery, dose of carfilzomib and lenalidomide should be reduced by one dose level			
**Constitutional**	Fatigue[a] ≥ Grade 3 (ie, severe fatigue interfering with activities of daily living)		Hold the dose until resolved to Grade ≤2. Consider reduction of lenalidomide or carfilzomib or dexamethasone by 1 dose-level.		

Cutaneous	Non-blistering rash Grade 2	Consider holding Isatuximab, if potentially related to isatuximab.	Hold carfilzomib therapy if potentially related to carfilzomib.	Consider holding lenalidomide if potentially related to lenalidomide.	
	Non-blistering rash ≥ Grade 3 or 4	Hold carfilzomib and lenalidomide and Isatuximab therapy, based on the potential correlation with one or more drugs.<br>Begin treatment with antihistamines and/or low-dose steroids as per institutional practice.<br>If the toxicity resolves to ≤ Grade 1, reduce dose by 1 level and restart carfilzomib and lenalidomide and continue antihistamines and/or low-dose steroids as per institutional practice. For grade 4 toxicity permanently discontinue Carfilzomib and or lenalidomide and or isatuximab permanently.			
	Desquamating (blistering) rash- any grade or erythema multiform ≥ Grade 3	Discontinue isatuximab carfilzomib and lenalidomide permanently.<br>Hold other therapies. Begin treatment with antihistamines and/or low-dose steroids as per institutional practice. If the toxicity resolves to ≤ Grade 1, restart all the medications after dose reduction.			
	Dyspepsia, gastric or duodenal ulcer, gastritis Grade 1-2 (requiring medical management)				Treat with histamine-2 blockers, sucralfate, or proton pump inhibitor.<br>If symptoms persist despite above measures, decrease dexamethasone dose by 1 dose-level.
	Dyspepsia, gastric or duodenal ulcer, gastritis ≥ Grade 3 (requiring hospitalization or surgery)	Day 1 of cycle: Delay Day 1 administration until recovery. Then, restart full dose isatuximab and same dose level of carfilzomib and lenalidomide, and decrease dexamethasone by 1 dose level of current dose along with concurrent therapy with H2 blockers, sucralfate, or omeprazole.<br>Within cycle: Hold study treatment until symptoms adequately controlled. Then, restart full dose isatuximab and same dose level of carfilzomib and lenalidomide, and decrease dexamethasone by 1 dose level of current dose along with concurrent therapy with H2 blockers, sucralfate, or omeprazole.<br>If delay >3 days, omit the dose.<br>If symptoms persist despite above measures, dexamethasone permanently discontinued.			
	Acute Pancreatitis	Day 1 of cycle: Delay Day 1 administration until recovery and re-start full dose isatuximab and same dose level of carfilzomib/lenalidomide if considered not drug related.<br>Within cycle: Hold all study treatment until recovery and re-start full dose isatuximab and same dose level of carfilzomib/lenalidomide if considered not drug-related.<br>If delay is >3 days, omit the dose.			Permanently discontinue dexamethasone.
Hematological	Neutropenia Grade 3 (without complications)	Hold Isatuximab, Lenalidomide, dexamethasone and Carfilzomib. Follow CBC weekly. Consider G-CSF support.<br><br>Returns to ≥ 1.0 x 10^9/L Resume Lenalidomide at next dose level reduction.<br>Resume Isatuximab at full dose and Carfilzomib and dexamethasone at the same dose level.			
	Grade 3 neutropenia associated with fever (≥38.5°C) or Grade 4 neutropenia	Day 1 of cycle: Hold Isatuximab, Lenalidomide, dexamethasone and Carfilzomib until recovery. Follow CBC weekly. Consider G-CSF support.<br>Returns to ≥ 1.0 x 10^9/L Resume Lenalidomide at next dose level reduction.<br>Resume Isatuximab at full dose and carfilzomib and dexamethasone at the same dose level.<br><br>Within cycle: Full dose isatuximab and dexamethasone and omit dose carfilzomib and lenalidomide until neutrophil counts improve to ≥1 x 10^9/L and then re-start with reduction of carfilzomib and lenalidomide by 1 dose level and same dose of isatuximab and dexamethasone.			

	Platelet count <25,000/μL (ie, Grade 4) or Grade 3 thrombocytopenia with bleeding		Day 1 of the cycle: Hold therapy with all drugs until recovery to baseline OR ≤Grade 2. Upon recovery, Resume Lenalidomide at next dose level reduction, resume Carfilzomib and Isatuximab at the same dose level.<br>Within cycle: Hold isatuximab, carfilzomib, lenalidomide and dexamethasone until platelets counts improve to ≥10 x 10^9/L and/or bleeding is controlled, and then re-start all study treatment at the same dose level.<br>If delay is >3 days, omit the dose.			
**Hypertension**	Grade 2 Adult: Systolic BP 140 - 159 mm Hg or diastolic BP 90 - 99 mm Hg if previously WNL; change in baseline medical intervention indicated; recurrent or persistent (>=24 hrs); symptomatic increase by >20 mm Hg (diastolic) or to >140/90 mm Hg; monotherapy indicated initiated;<br><br>Grade 3 Adult: Systolic BP >=160 mm Hg or diastolic BP >=100 mm Hg; medical intervention indicated; more than one drug or more intensive therapy than previously used indicated;		If attributed to Carfilzomib:<br>- < Grade 3: Continue at same dose if initiation of appropriate treatment controls hypertension (see Appendix XX for guidance);<br>- ≥ Grade 3: Hold carfilzomib until resolution to normal or baseline. Initiate appropriate antihypertensive therapy prior to resuming carfilzomib at 1 dose decrement.			
**Infection**	Herpes Zoster[d] activation or reactivation<br>ANY grade		Hold ALL therapies until lesions are dry. If not already started, begin antiviral treatment<br>Once the infection is resolved all medications can be restarted without a dose reduction; however, continued antiviral prophylaxis is required.			
**Musculoskeletal**	Muscle weakness >Grade 2 (symptomatic and interfering with function +/- interfering with activities of daily living)					Decrease dexamethasone dose by 1 dose-level. If weakness persists despite above measures, decrease dose by 1 *further* dose-level. If symptoms *still* persist, permanently discontinue dexamethasone.
**Metabolic**	Hyperglycemia ≥ Grade 3					Treatment with insulin or oral hypoglycemics. If uncontrolled despite above measures, decrease dose by 1 dose-level until levels are satisfactory.
**Neurological[e]**	Peripheral Neuropathy (Sensory or Motor) and/or Neuropathic Pain	Grade 1 (paresthesia and/or loss of reflexes) without pain or loss of function				
		Grade 1 with pain or Grade 2 (interfering with function but not with activities of daily living)				
		Grade 2 with pain or Grade 3 (interfering with activities of daily living)	Day 1 of cycle: Delay Day 1 administration until improvement to Grade 2 without pain and administer isatuximab and dexamethasone at the same dose level, and reduce carfilzomib and/or lenalidomide by 1 dose level, based on the correlation.<br>Within cycle: Full dose isatuximab, dexamethasone at the same dose level, and hold carfilzomib and/or lenalidomide based on the correlation until improvement to Grade 2 without pain and reduce by 1 dose level.<br>Discontinue carfilzomib if recurrence despite 3 dose reductions.			

		Grade 4 (permanent sensory loss that interferes with function) and/or severe autonomic neuropathy	Hold isatuximab and dexamethasone. When improvement at least Grade 2 with pain or Grade 3, resume isatuximab and dexamethasone at the same dose level Discontinue Carfilzomib and Lenalidomide.			
**Neuro-psychological**	Confusion or mood alteration >Grade 2 (interfering with function +/- interfering with activities of daily living)					Hold dexamethasone until symptoms resolve. Restart with 1 dose-level reduction. If symptoms persist despite above measures, permanently discontinue dexamethasone.
**Thromboembolic**	Venous and /or pulmonary thrombo-embolism ≥ Grade 3 [Deep vein thrombosis or cardiac thrombosis intervention indicate; eg: anticoagulation, lysis, filter, invasive procedure.]			Stop until toxicity resolves and therapeutic anticoagulation therapy to be started. Restart carfilzomib lenalidomide and dexamethasone at full dose after adequate anticoagulation.		
**Renal Impairment**	Moderate renal impairment-CrCl[b] 30-49 mL/min				Lenalidomide should be given at a dose of 10 mg daily[c] for Cycles 1-8. In Cycles 9-20 lenalidomide should be given at a dose of 5 mg daily.	
	Severe renal impairment-CrCl[b]<30 mL/min (not requiring dialysis)		Creatinine clearance <15 mL/min: Day 1 of cycle: Delay Day 1 administration until CrCl returns to ≥15 mL/mins. Within cycle: Omit isatuximab, carfilzomib, and dexamethasone. When CrCl returns to ≥15 mL/mins, resume full dose isatuximab, and same dose level of carfilzomib and dexamethasone. If delay is >3 days, omit the dose		Lenalidomide should be given at a dose of 15mg every 48 hrs in cycles 1-8. In cycles 9-20 lenalidomide should be given at a dose of 5 mg every 48 hrs.	
	End-stage renal disease- CrCl[b]<30 mL/min (requiring dialysis)				Lenalidomide should be given at a dose of 5mg daily for Cycles 1-8. In cycles 9-20 lenalidomide should be given at a dose of 5 mg every 48 hrs On dialysis days, administer dose after dialysis.	

**Hepatic function**	Mild to moderate liver dysfunction:<br>defined as 2 consecutive values, at least 28 days apart, of:<br>(1) total bilirubin (> 33% direct) > 1x ULN to < 3x ULN<br>OR<br>(2) an elevation of AST and/or ALT with normal bilirubin<br>Grade ≥ 3 AST, ALT, or total bilirubin Grade ≥3	**Carfilzomib:**<br>Mild to Moderated dysfunction: 25% dose reduction.<br>Dose may be re-escalated if liver function tests return to normal and drug-induced hepatotoxicity is excluded<br><br>Carfilzomib ≥ Grade 3 AST/ALT: Hold carfilzomib until resolution to baseline. Monitor any abnormality weekly. Resume carfilzomib with a 25% dose reduction if drug-induced hepatotoxicity is excluded.<br><br>Carfilzomib ≥ Grade 3 bilirubin increase: Hold carfilzomib until resolution to baseline. Monitor total bilirubin and direct bilirubin weekly. Upon resolution of total bilirubin to normal, resume carfilzomib dosing with a 25% dose reduction if drug-induced hepatotoxicity is excluded.<br><br>Discontinue Carfilzomib in the event of Drug-induced hepatotoxicity (attributable to carfilzomib)<br><br>**Isatuximab, Lenalidomide and Dexamethasone:**<br>Day 1 of cycle: Delay Day 1 administration until improvement to Grade 2 and then resume Isatuximab at the same dose level and reduce lenalidomide or dexamethasone by 1 dose level, according to drug-relation.<br>Within cycle: Full dose isatuximab, at the same dose level, and hold lenalidomide and dexamethasone until improvement to Grade 2 and resume carfilzomib lenalidomide and dexamethasone with dose reduction by 1 dose level according to drug relation.<br>If delay is >3 days, omit the dose.<br>Discontinue lenalidomide/dexamethasone if recurrence despite 3 dose reductions according to drug relation.
**Pulmonary hypertension**		Day 1 of cycle: Delay Day 1 administration until recovery or return to baseline value and then resume full isatuximab dose and lenalidomide and dexamethasone at the same dose level, and based on benefit risk assessment re-start carfilzomib with dose reduced by 1 dose level or stop carfilzomib.<br>Within cycle: Hold study treatment until resolution or return to baseline value and resume Lenalidomide and dexamethasone at the same dose level and based on benefit risk assessment to re-start carfilzomib with dose reduced by 1 dose level or to stop carfilzomib.<br>If delay is >3 days, omit the dose.<br>Discontinue carfilzomib if recurrence despite 1 dose reduction.
**Tumor Lysis Syndrome**	(≥ 3 of the following:<br>≥50% increase in creatinine, uric acid, or phosphate;<br>≥30% increase in potassium;<br>≥20% decrease in calcium; or 2-fold increase in LDH	Hold isatuximab, carfilzomib, lenalidomide and dexamethasone until all abnormalities in serum chemistries have resolved. Resume at full dose
**Pneumonitis/interstitial disease (as ADRS were associated with these symptoms)**	Including acute distress respiratory syndrome, acute respiratory failure	Day 1 of cycle: Delay Day 1 administration until Grade ≤1 or return to baseline, and resume full isatuximab dose and dexamethasone at the same dose level. Based on benefit risk assessment to re-start carfilzomib and lenalidomide at dose reduced by 1 dose level.<br>Within cycle: Hold study treatment until Grade ≤1 and resume full isatuximab dose and dexamethasone at the same dose level, and based on benefit risk assessment to re-start carfilzomib and<br>Lenalidomide with dose reduced by 1 dose level or to stop carfilzomib.<br>If delay is >3 days, omit the dose. Discontinue carfilzomib/lenalidomide if recurrence despite 1 dose reduction.
**Thrombotic microangiopathy**	Including thrombotic thrombocytopenic purpura and hemolytic uremic syndrome	Day 1 of cycle: Delay Day 1 administration until Grade ≤1, and resume full isatuximab dose and dexamethasone at the same dose level. Based on benefit risk assessment to re-start lenalidomide with dose reduced by 1 dose level. Based on risk benefit assessment consider stopping lenalidomide.<br>If the diagnosis of TMA is suspected, hold carfilzomib and manage per standard of care including plasma exchange as clinically appropriate. If TMA is confirmed, permanently discontinue carfilzomib. If the diagnosis is excluded, carfilzomib can be restarted<br><br>Within cycle: Hold study treatment until Grade ≤1 and resume isatuximab dose and dexamethasone at the same dose level, and based on benefit risk assessment to re-start carfilzomib and lenalidomide with dose reduced by 1 dose level or to stop carfilzomib/lenalidomide.<br>If delay is >3 days, omit the dose. Discontinue carfilzomib/lenalidomide if recurrence despite 1 dose reduction

**Posterior reversible encephalopathy syndrome (PRES)**	Symptom can include headaches, altered mental status, seizures, visual loss, and hypertension	Discontinue Isatuximab carfilzomib and lenalidomide.<br>Day 1 of cycle: Delay Day 1 administration until recovery, resume full isatuximab dose and dexamethasone at the same dose level if and when clinically appropriate.<br>Within cycle: After recovery, resume full isatuximab dose and dexamethasone at the same dose level if and when clinically appropriate.<br>If delay is >3 days, omit the dose.<br>If PRES is suspected, hold carfilzomib. Consider evaluation with neuroradiological imaging, specifically MRI, for onset of visual or neurological symptoms suggestive of PRES. If PRES is confirmed, permanently discontinue carfilzomib. If the diagnosis of PRES is excluded, carfilzomib administration may resume at same dose, if clinically appropriate.			
**Edema excluding infusion associated reaction and excluding edema from cardiac origin**	Grade ≥3 (limiting function and unresponsive to therapy or anasarca)				Diuretics as needed, and decrease dexamethasone dose by 1 dose level; if edema persists despite above measures, decrease dose another dose level. If symptoms persist despite second reduction, dexamethasone permanently discontinued.
**Other toxicities**	Any reported ≥ Grade 3	Determine drug attribution of the toxicity and hold the therapy(ies) as appropriate.<br>If toxicity resolves to ≤ Grade 1, resume therapy with 1 level of dose reduction for suspect drug.			
	[a] Determine if fatigue is possibly not medication-related but due to an underlying cause (eg, infection, progression of disease, diarrhea, anemia, depression) and treat these symptoms/causes as appropriate.<br>[b] CrCl = creatinine clearance. Estimated by creatinine clearance as calculated by the eGFR (MDRD) formula to assess renal function.<br>[c] Consider escalating dose to 15mg daily after 2 cycles if well tolerated.				

Lenalidomide re-escalation is allowed in case dose reduction occurred due to renal failures, if renal failures recovered. If lenalidomide dose is reduced to 5 mg in the induction or full-consolidation phase, no dose increase should be considered for the light-consolidation phase, which will start with a dose equal to 5 mg. Only in case the dose was reduced to 5 mg for renal impairment which totally recovered before the start of light-consolidation, lenalidomide dose can be re-escalated to 10 mg for the light consolidation phase.

Carfilzomib re-escalation is allowed, except for cardiac failure G3, drug-induced liver toxicity and hypersensitivity.

In case carfilzomib dose is reduced in the induction or full-consolidation phase, no dose increase should be considered in the light consolidation phase, and the last tolerated dose should be administered.

The start of a new cycle may be delayed for maximum of 28 days. In case of delay, the criteria responsible for delay have to be checked every week. If the start of a cycle is delayed more than 2 weeks, dose reduction of the study drug related to the toxicity causing the cycle delay should occur.

In case of delay of more than 4 weeks, treatment should be stopped.

8.3 Study drug administration

8.3.1 Isatuximab administration

- First infusion: initiate infusion at 25 mL/hour. In the absence of IRs after 1 hour of infusion, increase infusion rate by 25 mL/hour increments every 30 minutes, to a maximum of 150 mL/hour. In case of

grade 2 IR during first infusion, infusion could be restarted at one-half (12.5 mL/hour) of the initial infusion rate when the IR improves to Grade ≤1. If symptoms do not recur after 30 minutes, the infusion rate may be increased by 25 mL/hour increments every 30 minutes up to a maximum of 150 mL/h, until the total volume is infused.

- Second infusion: Initiate infusion at 50 mL/hour. In the absence of grade 2 IR after 30 minutes of infusion, increase rate to 100 mL/hour for 30 minutes, then, to 200 mL/hour until the total volume is infused. In case of grade 2 IR during second infusion, infusion could be restarted at one-half (25 mL/hour) of the initial infusion rate when the IR improves to Grade ≤1. If symptoms do not recur after 30 minutes, the infusion rate may be increased by 50 mL/hour increments every 30 minutes up to a maximum of 200 mL/h, until the total volume is infused.
- Third and subsequent infusions: Initiate infusion at a fixed infusion rate of 200 mL/hour, until the total volume is infused. In case of grade 2 IR during third infusion, infusion could be restarted at one-half (100 mL/hour) of the infusion rate when the IR improves to Grade ≤1. If symptoms do not recur after 30 minutes, the infusion rate may be increased by 50 mL/hour increments every 30 minutes up to a maximum of 200 mL/h, until the total volume is infused. The day of isatuximab administration, following premedication is recommended:

- Acetaminophen 650 mg to 1000 mg PO.
- Equivalent of ranitidine 50 mg IV or PO.
- Diphenhydramine 25 mg to 50 mg IV or PO (or equivalent).
- Dexamethasone 20 mg PO or IV at investigator discretion.

The recommended premedication agents should be administered 15-60 minutes prior to starting a isatuximab infusion. Patients who do not experience an IR upon their first 4 administrations of isatuximab may have their need for subsequent premedication reconsidered.

Patients are required to be monitored for 3 hours after isatuximab infusions for hypersensitivity reactions at least during the first cycle.

In case of dexamethasone intolerance or premature dexamethasone discontinuation, steroid premedication can be considered with methylprednisolone 100 mg IV if IAR infusion associated reactions premedication is still needed for isatuximab and/or carfilzomib according to investigator judgment. There is no minimum dose of dexamethasone required for isatuximab premedication (or equivalent). Indeed, lower dose than 20 mg dexamethasone (or 100 methylprednisolone) have not been tested, therefore if no IR occurs, premedication can be decreased or even stopped after 4 infusions if steroids are not tolerated, according to investigator judgment.

8.3.2 Carfilzomib administration

Carfilzomib is administered intravenously with an infusion time of 30 minutes that remains consistent throughout the regimen; adequate hydration is required before carfilzomib infusion during the first cycle of treatment.

For patients enrolled in the experimental arm, the carfilzomib infusion should follow the isatuximab infusion and should begin just after the end of the isatuximab infusion. The dose of carfilzomib will be escalated from 20 mg/m^2 to 56 mg/m^2 on Day 8 and for further administrations if the patient did not experience any toxicity Grade >2 (except noncomplicated hematological toxicity (toxicity meaning related to study treatment) or recovered tumor lysis syndrome).

Patients are required to be monitored for 3 hours after carfilzomib infusions for hypersensitivity reactions at least during the first cycle.

In case of dexamethasone being prematurely stopped and other study treatment being continued, steroid premedication can be considered with methylprednisolone 100 mg IV or at most 4 mg dexamethasone if IR premedication is still needed for isatuximab or carfilzomib according to investigator judgment.

Before carfilzomib infusion, the following actions should occur:

1. Hydration and Fluid Monitoring:

Hydration: All subjects must be well hydrated (i.e., volume replete). Begin oral hydration equal to approximately 30 mL/kg/day (~6–8 cups of liquid per day), starting 48 hours prior to the planned first dose of carfilzomib. Subjects may receive IV prehydration (normal saline or other appropriate IV fluid) prior to each carfilzomib infusion during cycle 1. Investigators must consider IV prehydration in subjects at high-risk for tumor lysis or renal toxicity. All subjects must be monitored for fluid overload and hydration should be tailored to individual needs and carfilzomib prehydration administered only if the subject’s condition and/or risk factors require it. Total volume will be determined at the discretion of clinician and volume status of patient. Adjust hydration per treating physician discretion for high risk of tumor lysis (increase hydration), and high risk of fluid overload or cardiac failure (decrease hydration). If lactate dehydrogenase (LDH) or uric acid is elevated at Cycle 1 day 8, then the recommended IV hydration should be administered on repeated for Cycle 1 day 8. The goal of the hydration program is to maintain robust urine output, (e.g., ≥ 2 L/day). Subjects should be monitored periodically during this period for evidence of fluid overload. Subjects should also be monitored for symptoms of fluid overload and treat per institution guidelines. Patients with a history of cardiac disease (such as CHF and cardiomyopathy) or pulmonary edema should be monitored closely for signs of fluid overload.

a. Total volume will be determined at the discretion of clinician and volume status of patient. If lactate dehydrogenase (LDH) or uric acid is elevated at Cycle 1 day 8, then the recommended IV hydration should be repeated for Cycle 1 day 8. The goal of the hydration program is to maintain robust urine output, (e.g., ≥ 2 L/day). Subjects should be monitored periodically during this period for evidence of fluid overload. Total volume of hydration can be less than 500 mL (no less than 250mL) or kept at 500 mL but administered on a longer time for patients with borderline

LVEF (above, but close to the eligibility criteria threshold of 40%) and/or for whom there is a risk of cardiac decompensation according to investigator's judgement.

b. In subjects considered to be still at risk for TLS at completion of Cycle 1, hydration should be continued into subsequent cycles if clinically indicated.

2. Laboratory Monitoring:

a. Appropriate chemistries, including creatinine, and complete blood counts (CBC) with platelet count should be obtained and reviewed prior to carfilzomib dosing. Results of laboratory studies must be reviewed and deemed resolved prior to administering the carfilzomib. Subjects with laboratory abnormalities consistent with lysis of tumor cells (e.g., serum creatinine ≥ 50% increase, LDH ≥ 2-fold increase, uric acid ≥ 50% increase, phosphate ≥ 50% increase, potassium ≥ 30% increase, calcium ≥ 20% decrease) prior to dosing should not receive the scheduled dose

3. Clinical Monitoring:

a. Signs and symptoms indicative of TLS, such as fevers, chills/rigors, dyspnea, nausea, vomiting, muscle tetany, weakness, or cramping, seizures, and decreased urine output.

b. Patients will be closely monitored while receiving Cycle 1 Days 1 and day 8 of therapy.

4. Management of TLS: (refer to Section 8.4.1).

8.4 Special precautions and supportive care

8.4.1. Tumor lysis syndrome

Subjects should be monitored for symptoms of tumor lysis syndrome (table 9).

Management of tumor lysis syndrome, including increasing hydration and treating hyperkalemia, hyperuricemia, and hypocalcemia, is highly recommended. It is also recommended that high-risk subjects, ie, those with a high tumor burden, be treated prophylactically in accordance with local standards (eg, increased hydration; allopurinol 300 mg daily and medication to increase urate excretion). Tumor lysis syndrome has to be managed according to site usual practice. The following table provides some parameters to be checked in case of TLS suspicion and high level recommendations for TLS management. After recovery, study treatment can be re-administered as planned at the same dose.

Table 9: Management of Tumor Lysis Syndrome

TLS main possible diagnosis criteria	Recommended action
**Laboratory TLS:** ≥2 simultaneous abnormalities within 3 days prior to and up to 7 days after treatment start:<br>•Uric acid >8 mg/dL (>475.8 µmol/L)<br>•Potassium >6.0 mmol/L<br>•Phosphorus >4.5 mg/dL (>1.5 mmol/L)<br>•Corrected calcium <7.0 mg/dL (<1.75 mmol/L), ionized calcium <1.12 mg/dL (<0.3mmol/L)[a]	Omit study treatment until all serum chemistries have resolved.<br>Ensure adequate hydration, correct laboratory abnormalities, fluid overload, uric acid lowering agents (such as allopurinol or rasburicase), electrolyte, or acid-base deviation.<br>Monitor TLS complications including renal functions. Reinstitute study treatment at full dose after resolution.

**Clinical TLS:** Laboratory TLS in addition to 1 of the following complications:<br>•Acute kidney injury: Increase in the serum creatinine level of 0.3 mg/dL (26.5 µmol/L) or the presence of oliguria, defined as an average urine output of <0.5 mL/kg/hour for 6 hours.<br>•Seizures, cardiac dysrhythmia, neuromuscular irritability (tetany, paresthesia, muscle twitching, carpopedal spasm, Trousseau's sign, Chvostek's sign, laryngospasm, bronchospasm), hypotension, or heart failure probably or definitely caused by hypocalcemia.<br>•Dysrhythmias probably or definitely caused by hyperkalemia.	Ensure adequate hydration, correct laboratory abnormalities, fluid overload, uric acid lowering agents (such as rasburicase), electrolyte, or acid-base deviation.<br>Monitor TLS complications including renal functions. Reinstitute study treatment at full dose after resolution.

[a] The corrected calcium level in milligrams per deciliter = measured calcium level in milligrams per deciliter + 0.8 x (4-albumin in grams per deciliter). Adapted from Hovard et al. (40)

Flowchart for the management of TLS

a. Monitoring: If TLS occurs, cardiac rhythm, fluid balance, and serial laboratory monitoring should be instituted (electrolites, renal function, uric acid)
b. Administer therapeutic and supportive care: ensure adequate hydration, correct laboratory abnormalities, fluid overload, uric acid lowering agents (such as rasburicase), electrolyte, or acid base deviation. Include dialysis, as clinically indicated.
c. Omit study treatment until all serum chemistries have resolved.
d. Monitor TLS complications including renal functions

Management of high risk TLS patients:

a. High risk definition: MM subjects with high tumor burden (e.g., Durie-Salmon or ISS Stage II/III) or rapidly increasing M-protein or light chains or compromised renal function (CrCl < 50 mL/min) should be considered to be at particularly high risk).
b. Allopurinol prophylaxis: is optional and will be prescribed at the Investigator's discretion, but is recommended given the potential for TLS in all patients. These subjects may receive allopurinol 300 mg PO BID (Cycle 1 Day -2, Day -1), continuing for 2 days after Cycle 1 Day 1 (total of 4 days), then reduce dose to 300 mg PO QD, continuing through Day 17 of Cycle 1. Allopurinol dose should be adjusted according to the package insert.
c. Other agents: such as febuxostat, might be used in subjects who do not tolerate allopurinol. .
d. Rasburicase: is recommended in patients with high tumor burden and high uric acid levels at baseline, to prevent TLS, and in patients with laboratory/clinical signs of TLS
e. Day 1 of cycle 1 in HIGH RISK OF TLS: possibility to start at D1 with administration of a single drug, if no TLS, add the other ones within max 3 days. If TLS, delay administration of the other drugs until all abnormalities in serum chemistries have resolved.

8.4.2. Changes in Body Surface Area (BSA)

Dose adjustments for carfilzomib do not need to be made for weight gains/losses of ≤ 20%.
Each dose will consist of carfilzomib for Injection administered on an mg/m^2 basis and should be based on the subject's actual calculated BSA. Subjects with a BSA of 2.2 m^2 or higher receive a dose based upon 2.2 m^2 BSA.

8.4.3 Guidelines for the management of infusion reactions

Patients should receive premedications prior to isatuximab and carfilzomib infusion as detailed in Section 8.5.1 – Mandatory Premedication to reduce the risk and severity of IRs commonly observed with mAbs and with carfilzomib.
Please refer to the current edition of the Investigator's brochure for IRs manifestations reported in patients treated with isatuximab and refer to carfilzomib product information for IRs manifestation reported with carfilzomib. In case of IRs while receiving or after isatuximab/carfilzomib administration, additional medication can be provided for symptom treatment as per Investigator judgement including diphenhydramine 25 mg IV (or equivalent) and methylprednisolone 100 mg IV, IV fluids, vasopressors, oxygen, bronchodilators, and acetaminophen or paracetamol. These patients must be informed of the potential risk of recurrent IRs. Further treatment with isatuximab (subsequent infusions) is to be started at 175 mg/hour and follow the same rule in case of IRs.
Once a Grade 2 IRs leading to interruption has improved to Grade ≤1, the isa infusion may be restarted at one half (87.5 mg/h) of the initial infusion rate. If symptoms do not recur after 30 minutes, the infusion rate may be increased in 50 mg/hour increments every 30 minutes, to a maximum of 400 mg/hour. Patients with a Grade 4 IRs must have the causative study treatment permanently discontinued and appropriate supportive therapy should be administered. If there is no possibility to distinguish which drug induced the IRs, study treatment should be permanently discontinued.

In case of grade 3 or 4 IRs occurring during isatuximab infusion, carfilzomib infusions will be postponed by one day (eg, planned D1 will be done on D2) assuming that the IRs improved to grade ≤ 1.
If a Grade 4 IRs occurs, the infusion must be stopped and treatment with isatuximab will be permanently discontinued for that patient.
If a Grade 3 IRs occurs, the isatuximab infusion must be interrupted, and the patient must be observed carefully and treated as needed until the resolution of the AE or until the AE improves to Grade 1. Only then, the infusion may be restarted at the investigator's discretion; if so, the infusion rate should be half of the infusion rate before the interruption, and it may be increased subsequently, at the investigator's discretion.

If the severity of an IRs returns to Grade 3 after the restart of the infusion, the same procedure described above may be repeated at the investigator's discretion. If a Grade 3 IRs occurs for a 3rd time, treatment with isatuximab will be permanently discontinued for that patient.

Table 10: Management of infusion reactions

NCI-CTCAE v5.0 criteria definition	Intervention recommendation
**Mild (Grade 1)**<br>Infusion interruption or intervention not indicated	Continuation of isatuximab/carfilzomib infusion per the judgment of the Investigator following close direct monitoring of the patient's clinical status. Isatuximab infusion may be stopped at any time if deemed necessary. If stopped, IR will be classified as Grade 2 as per NCI-CTCAE v5.0 and infusion will be re-started at half of the initial infusion rate.
**Moderate (Grade 2)**<br>Therapy or infusion interruption indicated, but responds promptly to symptomatic treatment (eg, antihistamines, NSAIDs, narcotics, IV fluids); prophylactic medications indicated for ≤24 hours	Stop isatuximab/carfilzomib infusion. Give additional premedication with diphenhydramine 25 mg IV (or equivalent) and/or methylprednisolone 100 mg IV (or equivalent) as needed. Isatuximab/carfil may be resumed only after patient recovery, at half of the initial infusion rate isatuximab and with close monitoring.
**Severe or life-threatening (Grade 3 or 4)**<br>Grade 3: prolonged (eg, not rapidly responsive to symptomatic medication and/or brief interruption of infusion); recurrence of symptoms following initial improvement; hospitalization indicated for clinical sequelae<br>Grade 4: life-threatening consequences; urgent intervention indicated	Stop isatuximab/carfilzomib infusion. Give additional premedication with diphenhydramine 25 mg IV (or equivalent) and/ or methylprednisolone 100 mg IV (or equivalent) and/or epinephrine as needed. Only then, if previous Grade 3 the infusion may be restarted at the Investigator's discretion; if so, the infusion rate should be half of the infusion rate before the interruption, and it may be increased subsequently, at the Investigator's discretion. If the severity of an infusion-related AE returns to Grade 3 after the restart of the infusion, the same procedure described above may be repeated at the Investigator's discretion. If a Grade 3 infusion-related AE occurs for a 3rd time, treatment with isatuximab will be definitively discontinued for that participant<br>Definitive treatment discontinuation in case of Grade 4.

Note: infusion should be completed within 16 hours from the end of infusion preparation or a new infusion should be prepared with the remaining dose to be administered the same day.

Abbreviations: AE = adverse event; IR = infusion reaction; IV = intravenous; NCI-CTCAE = National Cancer Institute Common Terminology Criteria for Adverse Events; NSAIDs = nonsteroidal anti-inflammatory drugs.

8.4.4. Guidelines for management of allergic and dermatologic reactions to lenalidomide syndrome

Lenalidomide must be discontinued for angioedema, grade 4 rash, exfoliative or bullous rash, or if Stevens-Johnson syndrome (SJS), toxic epidermal necrolysis (TEN) or Drug Reaction with Eosinophilia and Systemic Symptoms (DRESS) is suspected and should not be resumed following discontinuation from these reactions.

8.5 Mandatory concomitant medication

8.5.1 Pre-medication

Both isatuximab and carfilzomib can induce IRs and premedication is required prior to their administration. Patients should routinely receive premedications, which also includes dexamethasone, prior to isatuximab infusion to reduce the risk and severity of IRs commonly observed with mAbs and with carfilzomib. Dexamethasone should be administered prior to carfilzomib for patients allocated to

the KRd arm, and for patients allocated to Isa-KRd arm, dexamethasone should be administered prior to carfilzomib administration when there is no isatuximab infusion (otherwise prior to isatuximab). Detailed for management of IRs are provided in Section 8.4.3.

In case of dexamethasone being prematurely stopped and other study treatment being continued, steroid premedication can be considered with methylprednisolone 100 mg IV if IR premedication is still needed for isatuximab and/or carfilzomib according to investigator judgment.

Isatuximab premedication

Patients allocated to the Isa-KRd arm should routinely receive premedication prior to isatuximab infusion to reduce the risk and severity of IRs commonly observed with mAbs. The recommended premedication agents are diphenhydramine 25 to 50 mg IV (or equivalent), dexamethasone IV/PO (dose defined below), equivalent of ranitidine 50 mg IV, and acetaminophen 650 to 1000 mg PO 15 to 30 minutes (but no longer than 60 minutes) prior to isatuximab infusion. Once the premedication regimen is completed, the isatuximab infusion must start immediately.

The day of isatuximab administration, the following order is recommended:

- Acetaminophen (paracetamol) 650 mg to 1000 mg PO or IV,
- Equivalent of ranitidine 50 mg IV or PO,
- Diphenhydramine 25 mg to 50 mg IV or PO (or equivalent),
- Dexamethasone 20 mg IV or PO (which is also part of study treatment).

In countries where there is no IV formulation of diphenhydramine or equivalent, per os formulation is allowed from the first isatuximab infusion. In this case, it should be taken one to two hours prior to isatuximab infusion start.

For the patients who do not experience an IR upon 4 consecutive administrations of isatuximab, the Investigator may reconsider the need of specific isatuximab premedication for IRs.

A leukotriene receptor antagonist (LRA) such as montelukast is an optional pre-medication agent that can also be used to mitigate the risk of IRs in the first and second isatuximab administrations only, based on disease and patient’s characteristics.

Carfilzomib premedication

Details for hydration are provided in Section 8.3.2. When carfilzomib is administered without isatuximab (patients allocated to the KRd arm and on days 8 in induction cycles 2-4, day 8 full consolidation and on days 15 of the light consolidation phase for patients allocated to the IKRd arm), dexamethasone is to be administered at least 30 minutes prior to carfilzomib infusion.

- Equivalent of ranitidine: Commercial supplies of equivalent of ranitidine (other approved H2 antagonists, oral proton pump inhibitors) will be used for this study.
- Diphenhydramine or equivalent: Diphenhydramine is presented as a solution for IV infusion. Commercial supplies of diphenhydramine or equivalent (cetirizine, promethazine) will be used for this study.

- Acetaminophen or equivalent: Commercial supplies of acetaminophen or equivalent will be used for this study.

8.5.2 Pregnancy prevention

Celgene's Pregnancy Prevention Plan (PPP) for clinical trials is applicable for all subjects in this trial. The effects of isatuximab, carfilzomib and lenalidomide on the developing human fetus are unknown. For this reason and because immunomodulatory agents as well as other therapeutic agents used in this trial are known to be teratogenic, women of child-bearing potential and men must agree to use adequate contraception.

Isatuximab pregnancy prevention

FCBP or male subjects with FCBP shall be required to use effective contraceptive methods (intrauterine device, oral contraception or abstinence) starting 2 weeks before first isatuximab administration, while on therapy and for 5 months after the last dose of isatuximab.

The following highly effective methods of contraception are accepted:

- Established use of oral, intravaginal, or transdermal combined (estrogen and progestogen containing) hormonal contraception associated with inhibition of ovulation.
- Established use of oral, injectable, or implantable progestogen-only hormonal contraception associated with inhibition of ovulation.
- Placement of an intrauterine device or intrauterine hormone-releasing system.
- Male sterilization (provided that the partner is the sole sexual partner of the patient and that the sterilized partner has received medical assessment of the surgical success).
- Sexual abstinence.

If a woman were to become pregnant while taking isatuximab, treatment with isatuximab should be discontinued and the pregnancy should be followed until its outcome.

Males sexually active with FCBP must practice contraception during the treatment and for at least 5 months after the last isatuximab dose.

Carfilzomib pregnancy prevention

Carfilzomib could decrease the effectiveness of contraceptive methods taken by mouth. There is an increase in the risk of developing a blood clot (venous thromboembolism) with the use of carfilzomib. This is also a risk with some oral contraceptives. You should be aware of this risk when choosing a method of birth control.

Female patients must practice abstinence (not have sex) or always use highly effective methods for the entire duration of the treatment and for at least 30 days after the last carfilzomib dose.

Highly effective methods of birth control for Female Participants include:

- Combined (estrogen and progestogen) hormonal methods (pills, vaginal ring, or skin patch) ovary [pills, shots/injections, implants (placed under the skin by a healthcare provider)]

- Intrauterine device (IUD)
- Intrauterine hormonal-releasing system (IUS)
- Surgery to tie both fallopian tubes (bilateral tubal ligation/occlusion)
- Your male partner has had a vasectomy and testing shows there is no sperm in the semen
- Sexual abstinence (not having sex)

Male patients must practice abstinence (not have sex) or always use a condom with spermicide during treatment and for an additional 90 days after the last dose of carfilzomib.
Male patients must not donate sperm during treatment and for an additional 90 days after the last dose of carfilzomib.

Lenalidomide pregnancy prevention

Females of childbearing potential (FCBP) must have a negative serum or urine pregnancy test within 10 – 14 days and again within 24 hours prior to prescribing lenalidomide for Cycle 1 (prescriptions must be filled within 7 days) and must either commit to continued abstinence from heterosexual intercourse or begin TWO acceptable methods of birth control, one highly effective method and one additional effective method AT THE SAME TIME, at least 28 days before she starts taking lenalidomide. FCBP must also agree to ongoing pregnancy testing. Men must agree to use a latex condom during sexual contact with a FCBP during lenalidomide treatment and for at least 28 days after lenalidomide last dose, even if they have had a successful vasectomy. All patients must be counseled at a minimum of every 28 days about pregnancy precautions and risks of fetal exposure.

8.6 Recommended concomitant medications

8.6.1 Supportive bone therapy

A bone targeted therapy is strongly recommended for all subjects with evidence of lytic destruction of bone or with osteopenia. Either agent, a Bisphosphonate or monoclonal antibody can be used, the same class of bone modifying therapy should be maintained throughout the study and continued per treatment guidelines (43). Commercially available IV bisphosphonates (pamidronate and zoledronic acid) or monoclonal antibody are preferred, when available, and should be used according to the manufacturer's prescribing information, for subjects with osteolytic or osteopenic myelomatous bone disease. Oral bisphosphonates may be used as alternatives, if IV therapies are not available at the study site. It is preferred that investigators use the same route of bisphosphonate therapy for all subjects at their sites.
Subjects with evidence of lytic destruction of bone or with osteopenia who are not using bone modifying agent at the time of randomization should start a bone modifying agent as soon as possible during Cycle 1 or 2 of treatment. Investigators should not start bone targeted therapy during the study, unless it has been agreed with the sponsor that there is no sign of disease progression.

8.6.2 Transfusions/Growth Factors

1. Subjects may receive RBC or platelet transfusions if clinically indicated. The class of CD38 mAbs could interfere with blood bank serologic tests and thereby cause delays in issuing RBC units to patients receiving these agents. Complete blood phenotyping (isa arm only) if not already available (C,c; E,e; Kell. Kidd; Duffy; S,s is recommended, if not available follow site's standard) and antibody screening (Indirect Coombs Test or Indirect Antiglobulin Test [IAT]), after randomization and prior to study treatment initiation.
2. Colony-stimulating factors may be used if neutropenia occurs but should not be given prophylactically.

8.6.3 Prevention of Deep Venous Thrombosis

Lenalidomide has been associated with an increased risk of deep vein thrombosis and pulmonary embolism. Therefore, prophylaxis of venous thromboembolism (VTE) for all subjects is recommended according to IMWG guidelines (27). Both individual and myeloma-related risks of VTE should be considered in determining the type of thromboprophylaxis. In summary:

- Myeloma Risk factors:

All subjects for whom any myeloma therapy-related risk factor is present, low molecular weight heparin (LMWH) (equivalent of 40 mg enoxaparin once daily) or full-dose warfarin (target international normalized ratio [INR] 2-3) is recommended.

- Individual Risk factors:

If no risk factor, or any one risk factor is present, aspirin 81-325 mg once daily is recommended or dose per institutional standards.

If 2 or more risk factors are present, LMWH (equivalent of enoxaparin 40 mg once daily) or full-dose warfarin, INR 2-3, is recommended.

8.6.4 Prophylaxis for Herpes Zoster Reactivation

Prophylaxis for herpes zoster reactivation is recommended during the Treatment Phase and continue for 3 months following treatment. Initiate antiviral prophylaxis to prevent herpes zoster reactivation within 1 week after starting study treatment and continue for 3 months following study treatment. Acceptable antiviral therapy includes acyclovir (eg, 400 mg given PO 3 times a day, or 800 mg given PO 2 times a day or per institutional standards), famcyclovir (eg, 125 mg given, twice a day or per institutional standards), or valacyclovir (eg, 500 mg given PO, twice a day or per institutional standards.

8.7 Permitted Therapies

Subjects are to receive full supportive care during the study. The following medications and supportive therapies are examples of support therapies that may be used during the study:

- Colony stimulating factors, erythropoietin, and transfusion of platelets and red cells;
- Loperamide is recommended for the treatment of diarrhea, starting at the time of the first watery stool. The loperamide dose and regimen is according to institutional guidelines. Prophylactic loperamide is not recommended;
- Bile acid binders for bile acid diarrhea due to lenalidomide treatment;
- Prevention of constipation (eg, adequate hydration, high-fiber diet, and stool softeners, if needed);
- Adequate hydration is recommended for prevention of myeloma-related kidney disease;
- Prophylactic antiemetics, except for corticosteroids;
- An emergency short course of corticosteroid (equivalent of dexamethasone 40 mg/day for a maximum 4 days) is permitted before treatment;
- Palliative radiation therapy is permitted if clinically indicated.

Antibiotic prophylaxis

Antibiotic prophylaxis with ciprofloxacin or other fluoroquinolone or with trimethoprim/sulfamethoxazole (co-trimoxazole) is recommended, but not mandatory.

8.8 Prohibited therapies

- Other agents that target CD38
- Medications used for other indications that have anti-myeloma properties (for example, interferon and clarithromycin) (28-30)
- Approved or investigational treatments for multiple myeloma (including but not limited to conventional chemotherapies, immunomodulatory drugs [IMiDs], or proteasome inhibitors).
- Concomitant administration of investigational agents is prohibited, including administration of commercially available agents with activity against or under investigation for multiple myeloma.
- Systemic corticosteroids (>10 mg prednisone per day or equivalent) other than those given for IRs. Non-steroidal anti-inflammatory agents should be avoided as they may exacerbate myeloma-related kidney disease.
- Drugs known to prolong QT corrected (QTc) interval should be avoided unless deemed medically necessary.

8.9 Study drugs information

Summary of known and potential risks

Benefit-risk assessment of isatuximab, carfilzomib, lenalidomide, and dexamethasone (Isa-KRd) vs

carfilzomib, lenalidomide, and dexamethasone (KRd) will be conducted by comparing between-treatment differences of key efficacy and safety endpoints. Efficacy endpoints may include MRD negativity rate at different timepoints, PFS, TTP, TNT, PFS2, OS, duration of response and ORR. Safety endpoints may include serious infections, atrial fibrillation, diarrhea, infusion reactions and the following AEs/SAEs: hematological toxicities, cardiac failures, myocardial infarction, thromboembolic events and secondary primary malignancies. Safety endpoints that show no between-treatment differences will be noted, but may be excluded from the benefit-risk analyses.

Preparation and labeling

Carfilzomib and Isatuximab will be shipped to trial sites in containers labeled as an Investigational Medicinal Product. Carfilzomib and Isatuximab will be prepared and labeled in compliance with GMP and other applicable regulatory requirements.
Lenalidomide and Dexamethasone with commercial packaging will be used, according to national practice. Lenalidomide and Dexamethasone will be labeled in compliance with GMP and other applicable regulatory requirements for the trial and will be shipped to trial sites.

Storage and handling

Investigators or other authorized persons (eg, Pharmacists) are responsible for storing isatuximab and carfilzomib in a secure and safe place with restricted access in accordance with local regulations, labeling specifications, policies, and procedures.
Isatuximab is to be stored at +2°C to +8°C (36°F to 46°F). All vials must be kept in their box until use. No protection from light is required for storage in the infusion bags
Lyophilized Carfilzomib for Injection must be stored at 2–8ºC under the conditions outlined in the separate “Instructions for storage and use of lyophilized Carfilzomib for injection” in a securely locked area to which access is limited to appropriate study personnel.
Lenalidomide is to be stored at 25°C (77°F); excursions permitted to 15–30°C (59–86°F).
Dexamethasone is to be stored at controlled room temperature 20 to 25ºC (68 to 77ºF). Consult the package insert of the respective product for additional storage and usage instructions.

Study drugs supply

Isatuximab will be supplied to participant sites by Sanofi. Isatuximab drug product will be presented as a concentrate for solution for infusion in vials containing 20 mg/mL (500 mg/25 mL and 100 mg/5 mL) of isatuximab in 20 mM histidine, 10% (w/v) sucrose and 0.02% (w/v) polysorbate 80 at pH 6.0. It is packed in 30 and 6 mL glass vials fitted with elastomeric closure. Each vial will contain a nominal content of 500 or 100 mg isatuximab. The fill volume has been established to ensure removal of 25 or 5 mL. Each glass vial will be labeled as required per country requirement.
Carfilzomib will be supplied to participant sites by Amgen.
Lenalidomide will be supplied to study sites by the Sponsor. Lenalidomide commercial drug will be provided as blisters containing 21 capsules (3 blisters with 7 capsules each in every carton).

Dexamethasone will be supplied by the sponsor, unless applicable national laws and regulations allow the use of commercially available dexamethasone.

No investigational medicinal product will be shipped until the sponsor has verified that all regulatory required documents and approvals for the site are available.

Drug accountability

The investigator, or a pharmacist or other appropriate individual who is designated by the investigator, should maintain records of the product's delivery to the trial site, the inventory at the site, the use by each patient, and the return to the sponsor or alternative disposition of unused product(s). These records should include dates, quantities, batch/serial numbers, expiration dates (if applicable), and the unique code numbers assigned to the investigational product(s) and trial patients (if applicable). Investigators should maintain records that document adequately that the patients were provided the doses specified by the protocol and reconcile all investigational product(s) received from the sponsor. The investigator should also collect and count remaining medication, empty boxes and blisters of medication to check that the patient has taken the assigned dose.

Study drug return and destruction

Partially used investigational medicinal product should not be redispensed to either the same or another patient after it has been returned.

Used, unused or partially used study drug containers should be destroyed after drug accountability records have been completed according to national procedure and after the sponsor greenlight for destruction has been received, as applicable. Destruction or study return should be documented.

At the end of the trial or after expiry of the product, unused investigational medicinal product should be destroyed according to national procedure. Destruction or study return should be documented.

9. Follow-up MRD study

Apart from subjects that already experienced disease progression or received further ant-myeloma therapies, all the patients at least in VGPR still in the study (with or without completion of light consolidation treatment) will undergo to additional MRD monitoring.

MRD by NGF and NGS will be evaluated once a year for a 3-years period, as explained below:

- 1 year after the end of treatment (NGS + NGF)
- 2 years after the end of treatment (NGS + NGF)
- 3 years after the end of treatment (NGS + NGF)

At those timepoints, bone marrow and peripheral blood samples will be collected and sent to the study central labs.

For details on long term FU, please see section 10.

10. Study procedures

10.1 Time of clinical evaluations

Screening

The pre-randomization phase is meant to screen subjects for eligibility. Maximum duration of screening is 28 days.

The following evaluations will be performed:

- Obtain informed consent before any study-specific assessments are performed;
- Medical history including multiple myeloma diagnosis;
- Pregnancy testing in females of child-bearing potential;
- Female patients of child-bearing potential and male patients with partners of child-bearing potential must have contraceptive measures addressed at screening, sufficient time to employ required contraceptive measures prior to Day 1, and confirmation of adequate contraceptive measures;
- Complete physical examination, including vital signs (blood pressure, heart rate, temperature, oxygen saturation and respiratory rate);
- ECOG and WHO Performance Status evaluation;
- Radiological tests to document baseline size within 42 days before randomization; CT or MRI (total body MRI or spine and pelvis MRI) are recommended according to investigator discretion;
- PET-CT in case of extramedullary plasmacytoma should be repeated at least after each treatment phase (at the end of induction, after ASCT, at the end of consolidation and at the end of light-consolidation); if not possible and extramedullary disease size has been evaluated in detail at the diagnosis (CT/MRI measure of lesions diameter) MRI or CT could also been used. The methodology used for evaluation of each disease site should be consistent across all visits;
- Electrocardiogram;
- ECHO cardio;
- Forced expiratory volume test (subjects with COPD or asthma) for patients >65 years and younger patients with a medical history positive for pulmonary disease;
- Bone marrow aspiration and/or biopsy for morphology, immunophenotype and FISH assessment must be performed. Morphology analysis should be performed locally. MRD baseline evaluation, immunophenotype and FISH analysis will be centralized in two labs (Torino and Rotterdam). Sample will be used for MRD baseline evaluation by NGS and immunophenotype and centralized (Torino and Rotterdam Labs);
- Bone marrow samples and peripheral blood will be collected at regular timepoints for correlative studies (see Appendix L);
- Clinical chemistry: calcium, alanine transaminase (ALT), aspartate transaminase (AST), acid uric, potassium, magnesium, glucose, sodium, bilirubin, creatinine clearance, albumin, phosphate, urea, alkaline phosphatase, lactic acid dehydrogenase (LDH);
- Hematology tests: hemoglobin, white blood cell count, platelet count, ANC;

- For pts randomized in the Isa-KRd arm, complete blood phenotype available (C,c; E,e; Kell. Kidd; Duffy; S,s is recommended, if not available follow site's standard) and antibody screening (Indirect Coombs Test or Indirect Antiglobulin Test [IAT];
- Serum immunoglobulin quantitation and M-protein quantitation and immunofixation;
- 24-hour urine collections for quantification of urine protein, M-protein electrophoresis and quantification and immunofixation;
- FLC levels to evaluate involved serum FLC;
- Urinalysis: specific gravity, pH, glucose, bilirubin, protein, ketones and blood;
- Thrombosis assessment (clinical evaluation);
- Neurotoxicity assessment (clinical evaluation);
- β2-microglobulin, C-reactive protein;
- HBsAg, HBsAb, HBcAb; in patients with positive HBcAb HBV-DNA evaluation is mandatory: these patients can be enrolled if HBV-DNA is negative. Patients with positive HBsAb, negative HBcAb and a medical history of Vaccination can be enrolled without HBV-DNA evaluation, if normal AST/ALT/GGT values;
- HCV Ab and, if positive, HCV-RNA;
- HIV Ab;
- Completion of QOL questionnaires.

Patients can be "re-screened" at the discretion of the Investigator. Re-screening is restricted to one attempt per patient and can be performed within 3 months from the screening failure, in case the patient previously failed to be eligible due to any eligibility criteria that has been solved. Re-screened patients must sign and date a new ICF and will receive a new identification unique number and a new eCRF must be completed. In case bone marrow and radiological examinations were performed in the previous 3 months, sponsor should be counseled and these should not be repeated upon sponsor agreement.

Treatment phase

7 days can occur between the randomization and the start of treatment.

- Pregnancy testing in females of child-bearing potential before start each cycle (day -1 or 1) at least every four weeks;
- Symptom-directed physical examination and ECOG evaluation (day 1);
- Vital signs (blood pressure, heart rate, oxygen saturation and respiratory rate) on each day of Carfilzomib and Isatuximab dosing;
- Weight is to be measured on day 1 of each cycle and at the end of treatment. If weight or BSA changes by more than 10% from baseline, the dose of all study treatments will be re-calculated;
- Clinical chemistry: calcium, alanine transaminase (ALT), aspartate transaminase (AST), acid uric, potassium, magnesium, glucose, potassium, sodium, bilirubin, creatinine clearance, albumin, phosphate; test should be performed and results reviewed prior to each infusion, at least weekly, during the first two cycles, then at least every two weeks. Blood tests should be repeated more frequently if clinically indicated, according to investigator discretion;

- Hematology: hemoglobin, white blood cell count, platelet count, ANC; test should be performed and results reviewed prior to each infusion, at least weekly during the first two cycles, then at least every two weeks. Blood tests should be repeated more frequently if clinically indicated, according to investigator discretion;
- Serum M-protein quantitation with additional immunofixation in case of negative SPEP (day 1 each cycle);
- 24-hour urine collections for urine protein, M-protein electrophoresis and quantitation with additional immunofixation in case of negative UPEP (day 1 each cycle);
- Serum FLC to evaluate response and to confirm stringent CR for patients with baseline involved serum FLC levels > 10 mg/dl (day 1 each cycle);
- Thrombosis assessment (day 1 each cycle) clinical evaluation;
- Neurotoxicity assessment (day 1 each cycle) clinical evaluation;
- Completion of QOL questionnaires.

Acceptable window for all the above tests:

- From induction to post ASCT dose consolidation (included): 3 days
- During light consolidation: 7 days

- Bone Marrow and Peripheral blood (centralized, Torino and Rotterdam). In patients who achieved at least VGPR, MRD by NGF and NGS will be performed after induction, after ASCT and after post ASCT consolidation, after light consolidation and during the observation period for 3 years.
- In patients that achieved at least VGPR, PET/CT will be performed after post ASCT consolidation and after light consolidation to monitor MRD outside the bone marrow. PET/CT will be performed in patients that do not achieve at least a VGPR for plasmacytoma evaluation.

Acceptable window for MRD- bone marrow and PET:

- Induction and post ASCT consolidation (included) 2 weeks
- Post ASCT, during light consolidation and during observation: 4 weeks

Continuous Procedures

- Record concomitant medications and transfusions since last visit;
- Assess and record adverse events for each study visit or when reported spontaneously between visits from signature of the informed consent;
- Record laboratory values indicative of the onset of an adverse event, the most abnormal value observed during the adverse event, and the resolution of the adverse event;
- Record hospitalization days and reason for hospitalization since last visit.

Observation

Light Consolidation treatment completion (before progression).

Data on response assessment and treatment administered (Maintenance therapy), should be provided every 2 months (acceptable window 2 weeks, local evaluation). During this phase, maintenance and response forms of the "Observation" phase should be filled-out.

Long Term Follow up (LTFU)

Before progression

Subjects who will drop out from treatment for any reason other than disease progression and light consolidation treatment completed, should collect data on response assessment (Unscheduled Clinical Response Form), every 2 months (acceptable window 2 weeks, local evaluation) until the development of PD. After PD they should enter LTFU.

NOTE: for participants who discontinue study intervention without disease progression and light consolidation not completed, disease response assessments should continue at least every two months until disease progression, withdrawal of consent, initiation of subsequent anticancer therapy (except for maintenance therapies), participant lost to follow-up, death or defined end of study.

After progression

After progression is documented, documentation of subsequent treatment and survival status is required. This should be done at least every 90 days (acceptable window 4 weeks) by adding Follow Up Forms. If the information on subsequent treatment and survival status is obtained without having the subject visit to the study center, written documentation of the communication must be available for review in the source documents. Information about PFS2 should be collected when available, since it is a secondary objective. LTFU should continue until approximately 9 years after the last randomized subject or approximately 77 deaths, whichever occurs first.

10.2 Time and events schedule

Table 11: Study procedures and assessments required per protocol

Required investigations at entry, during treatment and during follow up

	Screening ≤ 28 days from Baseline	Baseline C1D1 ≤ 7 days after randomization	Induction cycle 1-4 (±3 days)	End of cycle 4 (±3 days)	Cycle 5-8 (±3 days)	Before start light consolidation (±3 days)	During light consolidation (±7 days)	At the end of light consolidation or at the end of treatment (whatever it occurs)	Observation (until PD, every 2 months ± 2 weeks. Window for BM samples is ± 1 month)	Long term follow up (after PD every 90 days ± 4 weeks)
**Informed consent**	X (before the enrollment)									
**Medical history**	X	X								
**Urine or serum pregnancy test (for FCBP only)[a]**	X	X	X	X	X	X	X	X		
**Pregnancy counseling (as required per Lenalidomide PPP)**	X	X	X		X	X	X	X		
**Physical examination (including physical evaluation of extramedullary plasmacytoma)***	X	X	X	X	X	X	X	X		
**Vital signs***	X	X	X	X	X	X	X	X		
**ECOG**	X	X	X							
**PET-CT**	X[5]					X[5]		X[5]		
**Imaging/CT/MRI[b]**	X[3]			X[3]		X[3]		X[3]		X[3]
**X-thorax[b]**	X									
**12 Lead ECG [b 7]**	X		X[7]		X[7]		X[7]			
**ECHO [8]**	X				X[8]		X[8]			
**Forced expiration volume test[b]**	X									
**Bone marrow aspirate**	X[4]									
**Bone marrow biopsy**	X									
**Cytogenetic analysis /FISH**	X[4]									

	Screening ≤ 28 days from Baseline	Baseline C1D1 ≤ 7 days after randomization	Induction cycle 1-4 (±3 days)	End of cycle 4 (±3 days)	Cycle 5-8 (±3 days)	Before start light consolidation (±3 days)	During light consolidation (±7 days)	At the end of light consolidation or at the end of treatment (whatever it occurs)	Observation (until PD, every 2 months ± 2 weeks. Window for BM samples is ± 1 month)	Long term follow up (after PD every 90 days ± 4 weeks)
PB/BM sampling for Cryopreservation – only in pts with VGPR or better	X[4]			X[4]	X[4]	X[4]		X[4]	X[4]	
BM sampling for immunophenotype at baseline, MRD (NGF and NGS) – only in pts with VGPR or better	X[4]			X[4]	X[4]	X[4]		X[4]	X[4]	
PB sampling for correlative studies and for isatuximab M-protein interference test – only in pts with VGPR or better	X[9]			X[9]	X[9]	X[9]		X[9]		
Clinical chemistry[c*]	X	X	X[1]		X[1]	X[1]	X[1]	X[1]		
Hematology[c*]	X	X	X[1]		X[1]	X[1]	X[1]	X[1]		
Blood group and type and indirect antiglobulin test[d]	X[c]									
Serum Immunoglobulin*, M-protein	X	X	X		X		X	X	X	
24-hours urine collection and M-protein	X	X	X[2]		X[2]		X[2]	X	X	
FLC	X	X	X[2]		X[2]		X[2]			
Urinalysis	X									
Thrombosis assessment**	X	X[2]	X[2]		X[2]		X[2]			
Neurotoxicity assessment	X	X[2]	X[2]		X[2]		X[2]			
β2-microglobulin, C-reactive protein	X									
HBsAg, HBsAb, HBcAb	X									
HCV and HIV Ab	X									
Response evaluation			X	X	X	X	X[2]	X	X	
Quality of Life	X[6]	X[6]	X[6]		X[6]		X[6]		X[6]	X[6]
Concomitant medication	X	X	X	X	X	X	X	X		

	Screening ≤ 28 days from Baseline	Baseline C1D1 ≤ 7 days after randomization	Induction cycle 1-4 (±3 days)	End of cycle 4 (±3 days)	Cycle 5-8 (±3 days)	Before start light consolidation (±3 days)	During light consolidation (±7 days)	At the end of light consolidation or at the end of treatment (whatever it occurs)	Observation (until PD, every 2 months ± 2 weeks. Window for BM samples is ± 1 month)	Long term follow up (after PD every 90 days ± 4 weeks)
**Adverse event and Seriousadverse event**	X	X	X	X	X	X	X	X	X	
**Subsequent treatment**									X	X
**Survival status**									X	X

o.i. on indication

1. during cycle 1-2 every week, from cycle 2 on, every 2 weeks, or more frequent as clinically indicated
2. day 1 each cycle
3. in case of **extramedullary plasmacytoma**, the imaging should be repeated at least at the end of each treatment phase (at the end of induction, after ASCT, at the end of consolidation and at the end of light-consolidation).
   Imaging will be performed at disease progression. During the treatment phase and before disease progression is confirmed, imaging should be performed whenever clinically indicated based on symptoms, to document response or progression.
   The methodology used for evaluation of each disease site should be consistent across all visits.
4. at central lab (Turin/Rotterdam). BM sampling referred to the cycle 5-8 will be collected within 14 days prior to the start of cycle 5. Screening MRD will be performed by NGS and by diagnostic immunophenotype. After the end of treatment, BM and PB samples will be collected once a year until 3 years from the end of treatment (± 1 month), according to the follow-up MRD study, section 9. BM and PB samples should be sent to the central lab (Turin/Rotterdam) at disease progression.
   Bone marrow aspirate to be repeated locally when a complete response is suspected.
5. within 42 days before randomization; Low-Dose CT, MRI and/or X ray according to investigator discretion. PET/CT for MRD evaluation in pts that achieve at least a VGPR, should be performed within 2 weeks after the end of the consolidation phase and within 4 weeks after the end of the light-consolidation phase.

6 EORTC QLQ-C30, EORTC QLQ-MY20, EQ5D-5Lwill be collected every 2 months in the “observation period”, then every 3 months after PD, until death or study termination.
7 ECG should be repeated every other cycle during treatment.
8 ECHO should be repeated every 6 months
9 Isatuximab M-protein interference test for the confirmation of the CR/sCR should be performed only in pts at least in VGPR treated with isatuximab and with isotype IgGK at the timepoints specified in the above table. The M-protein interference assay will be performed by Torino and Rotterdam central labs.

[a] FCBP must perform pregnancy test during screening 10-14 days prior to the start of therapy and repeated within 24 hours prior to drug administration. During the study, weekly pregnancy test must be repeated during cycle 1 and every day 1 for the following cycles (every 2 weeks for FCBP with irregular menstrual cycle). Pregnancy test should be repeated at the end of treatment and 28 days after the last lenalidomide administration, For FBCP with irregular menstrual cycle the pregnancy test should be repeated at the end of treatment, 14 and 28 days after the last lenalidomide dose.

[b] Acceptable for screening if performed as part of SoC within 42 days before randomization.

[c] At screening acceptable to meet CRAB criteria if performed as part of SoC within 42 days before randomization. May be repeated up to 3 days before study drug administration day. Results must be evaluated before drug administration.

[d] Exams to be performed only for pts enrolled in the experimental arm (Isa-KRd). Exams will be performed locally once before the first administration of isatuximab.

* Results of vital signs, physical examination, hematology, serum immunoglobulin and clinical chemistry will not be collected in e-CRF after screening. Any abnormal results of vital signs, physical examination, hematology and serum chemistry should be reported in the eCRF form on the AEs or SAEs page.

** Thrombosis assessment will be reported in eCRF only in case of abnormal assessment results.

Medical history

Standard medical history, with special attention to symptoms of myeloma and relevant co-morbidities:

- Bone pain;
- Infections;
- Bleeding tendency;
- Constipation;
- Polyneuropathy.
- Cardiovascular disease;
- Risk factor assessment.

Only at entry:

- Occupational history;
- Prior and present other diseases;
- Antecedent hematological or oncological diseases;
- Prior chemotherapy or radiotherapy.

Physical examination

A complete physical examination (including neurological examination) should be performed during the Screening Phase and at the beginning of each cycle. Height will be measured at screening only; weight will be measured regularly as specified in the Time and Events Schedule.

Thereafter, only a symptom and disease directed physical examination is required. Abnormalities will be recorded in the appropriate sections of the eCRF.

Vital signs (blood pressure, heart rate, temperature, oxygen saturation and respiratory rate) will be performed as specified in the Time and Events Schedule. It is recommended that blood pressure (sitting) and heart rate measurements be preceded by at least 5 minutes of rest in a quiet setting without distractions (eg, television, cell phones). Only vital signs taken at Screening or associated with an AE will be recorded in the eCRF; all measurements will be recorded in the source documents.

Electrocardiogram (ECG)

12-lead ECGs will be performed as specified in the Time and Events Schedule. Whenever possible, ECGs should be taken immediately before chemistry assessment. During the collection of ECGs, subjects should be in a quiet setting without distractions (e.g. television, cell phones). Subjects should rest in a supine position for at least 5 minutes before ECG collection and should refrain from talking or moving arms or legs. If blood sampling or vital sign measurement is scheduled for the same time point as ECG recording, then the procedures should be performed in the following order: ECG(s), vital signs, blood draw. ECG should be repeated every other cycle.

ECHO or MUGA for LVEF assessment.

Hematology

- Hemoglobin
- white blood cell (WBC) count
- Platelets count
- absolute neutrophil count

Clinical chemistry

- urea
- creatinine clearance
- sodium
- albumin
- glucose
- AST
- ALT
- magnesium
- alkaline phosphatase
- lactic acid dehydrogenase (LDH)
- potassium
- calcium
- uric acid
- bilirubin (total bilirubin and direct bilirubin)
- phosphate

Myeloma Protein Measurements in Serum and Urine

Blood and 24-hour urine samples for M-protein measurements will be analyzed locally.

- IgG, IgA, IgM, IgD and IgE at Screening.
- Serum M-protein quantitation by electrophoresis (SPEP)
- Serum immunofixation (SIFE) at screening and thereafter when M-protein is non-quantifiable up to confirmation of CR
- 24-hour urine M-protein quantitation by electrophoresis (UPEP)
- Urine immunofixation (UIFE) at screening and thereafter when a M-protein is non-quantifiable up to confirmation of CR
- Free light chain assessment for subjects with light chain only myeloma AND for all subjects to confirm sCR

The isatuximab M-protein interference test by serum immunofixation electrophoresis (IFE) will be performed centrally in patients with isotype IgGK treated with isatuximab and that reached at least a VGPR.

M-protein follow-up by IFE is part of the International Myeloma Working Group (IMWG) criteria to assess treatment response. Therefore, it is crucial that the isatuximab band is not confused with the endogenous M-protein of the patient during IFE interpretation.

For subjects with suspected isatuximab interference on serum IFE, the SEBIA HYDRASHIFT 2/4 isatuximab IFE test will be used to specifically measure the endogenous M-protein. Subjects that meet all other IMWG criteria for CR, and for whom negative immunofixation is confirmed after using the

HYDRASHIFT isatuximab test, will be considered complete responders. This will define the final CR rate as per IMWG criteria.

Blood and 24-hour urine samples will be collected as specified in the Time and Events Schedule every 4 weeks during treatment until full dose consolidation, then every 8 weeks during light consolidation until PD occurs.

HBV-DNA Tests:

Subjects who are positive for antiHBc or antiHBs will undergo testing for hepatitis B DNA by PCR. Subjects who are positive for antiHBs antibodies due to prior immunization are not required to be monitored by HBV DNA by PCR. During and following study treatment, subjects who have history of HBV infection will be closely monitored for clinical and laboratory signs of reactivation of HBV according to the JSH Guidelines for Prevention of HBV Reactivation in Patients Receiving Immunosuppressive Therapy or Chemotherapy (JSH Guidelines for the Management of HBV Infection 2014). Where required by local law, the result of HBV testing may be reported to the local health authorities.

Bone marrow

Bone marrow aspiration (obligatory) and biopsy (optional) at entry.

Centralized bone marrow aspirate sample is required at baseline for FISH assessment, and MRD baseline evaluation.

Bone marrow aspirate

- at entry for:
  - Morphology
  - FISH analysis: FISH analysis will be performed for deletion 1p32.3, the presence of 14q32 abnormalities (t(4;14)(t11;14) and t(14;16)), deletion of p53 (17p13), gain and amp 1q21
  - Immunophenotyping for baseline evaluation
  - MRD in NGS evaluation
- at response evaluation for confirmation of (s)CR, flowcytometry analysis to determine MRD as highlighted in schedule of study assessment and molecular and immunological studies (appendix K)
- once a year after the end of treatment as described in the schedule of study assessment, according to the follow-up MRD study (section 9).

Radiographic assessment

FDG-PET-CT before the start of treatment and after reaching ≥ VGPR post ASCT dose consolidation and light consolidation.

MRI, LD CT, X Ray at investigator discretion and as per standard guidelines.

Serum Pregnancy Test

Women of childbearing potential only. Urine pregnancy test allowed if results from a serum pregnancy test will not be promptly available. Lenalidomide is contraindicated for use during pregnancy, as even a single dose can induce a high frequency of severe and life-threatening birth defects. Guidelines presented in the Celgene lenalidomide pregnancy prevention program as per local lenalidomide labelling must be followed. If pregnancy does occur, then study treatment should be discontinued immediately and the subject should be referred to an obstetrician experienced in reproductive toxicity for further evaluation and counselling.

Additional/Specific investigations

– Serum ß2-microglobulin
– ECOG

10.3 MRD

Methods and timing of analysis

MRD evaluation by NGS clonotypic analysis of immunoglobulin heavy chain (IgH) VDJ gene rearrangement will be performed by employing the ClonoSEQTM assay (Adaptive Biotechnologies, Seattle) at sensitivity thresholds until ≥10^{-5}. The rate of MRD negativity (≥10^{-5} sensitivity level) will be determined as primary objective to compare efficacy of the 2 treatment arms of study protocol (Isa-KRd vs KRd) and as secondary objective to evaluate the rate of MRD negativity at different timepoints during treatment course.

Immunophenotype by NGF will be performed by central study laboratory using the EuroFlow Consortium guidelines: the method is based on a (standardized) lyse-wash-and-stain sample preparation protocol, the measurement of high numbers of BM cells (≥$5x10^6$ cells/tube) and an optimized 8-color, 2-tubes, antibody panel, for accurate identification of BM plasma cells (PCs) and discrimination between phenotypically aberrant (aPC) and normal PC (nPC).

Baseline bone marrow aspirate samples will be evaluated by a NGS assay to establish the myeloma clone (calibration) and for MRD monitoring. NGS is an emerging tool in the assessment of MRD in patients with multiple myeloma(33). Several studies have demonstrated that MRD status is correlated with PFS and OS (34). In this study, bone marrow samples will be collected when a bone marrow aspirate is performed at Screening and at the subsequent timepoints outlined in and Time and Events Schedule

Results obtained from NGF and NGS analysis will be compared, in order to understand the role of these two approaches in the detection of MRD.

MRD time points

Time-points for MRD assessment are listed in Time and schedule section. BM samples will be collected and stored for every patient at baseline, at the moment of relapse/progression and at a number of well-defined MRD time points, after having achieved at least VGPR:

1. Baseline: Screening (NGS)
2. after induction therapy (before ASCT) (NGS + NGF)
3. after ASCT (NGS + NGF)
4. after post ASCT consolidation (NGS + NGF)
5. after the end of light consolidation (NGS + NGF)
6. 1 year after the end of treatment (NGS + NGF)
7. 2 years after the end of treatment (NGS + NGF)
8. 3 years after the end of treatment (NGS + NGF)

In order to be able to compare results obtained from NGF and NGS MRD analyses and to avoid data misinterpretation, BM samples will be carefully processed at the central lab.

10.4 Quality of Life assessment

Quality of Life (QoL)

Subjects health-related quality of life (HRQoL), symptoms, functioning, and general well-being will be captured using 3 PRO measures: the European Organization for Research and Treatment of Cancer Quality of Life Questionnaire (EORTC QLQ-C30) (Appendix H), European Organization for Research and Treatment of Cancer Multiple Myeloma Module (EORTC QLQ-MY20) (Appendix I), and the EQ-5D-5L (Appendix J). These measures will be administered according to the Time and Events Schedule to understand how subjects self-reported health state changes over time and the difference between-treatment arms during induction, consolidation, maintenance, and post progression. The hypothesis that treatment with daratumumab maintains a subject's HRQoL when added to a triplet regimen will be tested using established meaningful change thresholds and statistical significance between groups.

The PRO measures will be provided in the local language. If a subject requires assistance completing the PRO, a study coordinator may assist but should not prompt the subject in selecting their response. At completion, the study coordination should check that the questionnaires are completed or document why they are missing. Full training documentation will be provided to site coordinators before the start of data collection.

EORTC QLQ-C30 version 3 includes 30 items resulting in 5 functional scales (physical functioning, role functioning, emotional functioning, cognitive functioning, and social functioning), 1 Global Health Status (GHS) scale, 3 symptom scales (fatigue, nausea and vomiting, and pain), and 6 single items (dyspnea, insomnia, appetite loss, constipation, diarrhea, and financial difficulties). The recall period is 1 week ("past week Item") and responses are reported using a verbal rating scale. The item and scale scores are transformed to a 0 to 100 scale. A higher score represents greater HRQoL, better functioning, and

more (worse) symptoms. The EORTC QLQ-C30 has been widely used among patients with multiple myeloma. Reliability, validity, and clinically meaningful change have been demonstrated (Wisloff 1996, Wisloff 1997). The EORTC Multiple Myeloma Module (QLQ-MY20) has been designed to use alongside the EORTC QLQ-C30 to address issues of more relevance to myeloma patients (31). The 20-items make up 4 scales: disease symptoms, side effects of treatment, future perspective, and body image. Recall, response options, and interpretation is similar to the EORTC QLQ-C30. Together the EORTC QLQ-C30 and the EORTC QLQ-MY20 administration time is less than 30 minutes. Key PRO endpoints include the global health status (GHS), physical functioning, fatigue, and pain scales from the EORTC QLQ-C30 and the Disease Symptoms scale from the EORTC QLQ-MY20.

The EQ-5D-5L is a generic measure of health status. The EQ-5D-5L is a 5-item questionnaire that assesses 5 domains including mobility, self-care, usual activities, pain/discomfort and anxiety/depression plus a visual analog scale rating "health today" with anchors ranging from 0 (worst imaginable health state) to 100 (best imaginable health state) (32). The scores for the 5 separate questions are categorical and are cannot be analyzed as cardinal numbers. However, the scores for the 5 dimensions are used to compute a single utility score ranging from zero (0.0) to 1 (1.0) representing the general health status of the individual. The EQ-5D-5L asks respondents to select their response based on their current health ("today") and takes less than 5 minutes to complete.

The EQ-5D-5L, the EORTC QLQ-C30 and the EORTC QLQ-MY20 will be performed until death or study end. Following disease progression, sites should attempt to administer the EQ-5D-5L, the EORTC QLQ-C30 and the EORTC QLQ-MY20 every 3 months, unless death or study end occurs first. Subjects who visit the site for the follow-up assessments should complete the EQ-5D-5L questionnaire at that time. If the EQ-5D-5L is conducted via a telephone call with the subject, then the subject's questionnaire responses will be read over the telephone to the site staff who will record the data in the eCRF. If the subject is unable to complete the EQ-5D-5L, the reason for not completing the questionnaire will be documented (ie, too ill, subject refused). This can be done by interview as part of the telephone contact documented in the eCRF.

10.5 Central review

Serum samples for confirmation of at least VGPR in the experimental arm will be stocked and subsequently analyzed (see schedule of study assessment).

Responses will be reviewed centrally for consistency in both arms at the same time points, based on data included in eCRF (SPEP, UPEP, IMMUNOFIX, FLC).

10.6 Side study

Biomarker Analyses

To explore the impact of the KRd regimen on Isatuximab’s mechanisms of action, whole blood samples may be used to evaluate Isatuximab’s immunomodulatory MoA, where specific subsets of immune cells such as cytotoxic T cells, regulatory T cells, and activated NK cells may be evaluated by FACS or cytometry/time-of-flight mass spectrometry (CyTOF) and T-cell receptor sequencing. Proteomic

analysis may also be used to evaluate changes in proteins in circulation to evaluate potential markers of clinical response. Description of other correlative studies are described in Appendix K.

11. Withdrawal of patients or premature termination of the study

11.1 Withdrawal of individual patients from protocol treatment

Patients should be withdrawn from protocol treatment if any of the following criteria for withdrawal are met:

- Death
- Patient not eligible in hindsight
- Progression/relapse during treatment
- Suspected pregnancy

Patients can leave the study at any time for any reason if they wish to do so without any consequences. The investigator can also decide to withdraw a patient from protocol treatment for other reasons than the criteria described above. Examples of such reasons for withdrawal from protocol treatment are:

- Excessive toxicity;
- Refusal of patient to continue protocol treatment;
- No compliance of the patient: patient is unable or unwilling to adhere to the treatment schedule and/or procedures required by the protocol;
- At physician discretion;
- Intercurrent illness or worsening of a chronic condition.

Patients who are withdrawn from protocol treatment will receive medical care according to local practice.

End of Treatment reason AE vs Death: For fatal SAEs occurring while the subject is still on study treatment, the primary reason for discontinuing treatment (AE vs. Death) should take into account whether there was an active decision by the investigator to discontinue treatment. If the investigator actively decided to discontinue study treatment due to AE before the AE became fatal, then the primary reason for treatment discontinuation should be ‘AE’. If the investigator did not actively decide to discontinue study treatment before the AE became fatal, then the primary reason for treatment discontinuation should be ‘Death’.

11.2 Follow up of patients withdrawn from protocol treatment

Patients who are withdrawn from treatment for other reasons than death will be followed as described in section 10.2 for follow up. SAE information will be collected as described in section 12.

Patients withdrawn before PD will be followed for response assessment, and patients withdrawn after PD will be followed for survival and second line therapy data.
However, for patients who are withdrawn from treatment because in hindsight they did not fulfill the eligibility criteria (see section 7) at time of registration, data will be collected until 30 days after the last protocol treatment given. SAE information will be collected as described in section 12.

11.3 Withdrawal of informed consent

If a patient states that he or she withdraws the consent to participate in the trial, the investigator should attempt to verify the patients intent and record this in the patients medical file:

- The patient can refuse further treatment and/or procedures according to protocol, while still consenting with further follow up data collection;
- The patient can refuse further treatment and/or procedures according to protocol, and withdraw consent for further follow up data collection.

If the patient intent is to withdraw consent for further data collection, the investigator should inform EMN so appropriate actions can be taken.
If the patient's intent cannot be verified, further follow up data will be collected for this patient as described in 10.2 for follow up.

11.4 Premature termination of the study

The sponsor may decide to terminate the study prematurely based on the following criteria:

- ♦ There is evidence of an unacceptable risk for study patients (i.e. safety issue);
- ♦ There is reason to conclude that continuation of the study cannot serve a scientific purpose following confirmation of the IDMC;
- ♦ The IDMC recommends to end the trial based on viable arguments other than described above.

The sponsor will promptly notify all concerned investigators, the Ethics Committee(s) and the regulatory authorities of the decision to terminate the study. The sponsor will provide information regarding the time lines of study termination and instructions regarding treatment and data collection of enrolled patients.

12. Safety

12.1 Definitions

Adverse event (AE)
An adverse event (AE) is any untoward medical occurrence in a patient or clinical study subject administered a medicinal product and which does not necessarily have a causal relationship with the treatment.

An AE can therefore be any unfavorable and unintended sign (including an abnormal laboratory finding), symptom, or disease temporally associated with the use of a medicinal (investigational) product, whether or not related to the medicinal (investigational) product.

Serious adverse event (SAE)

A serious adverse event is defined as any untoward medical occurrence or effect that at any dose:

- Results in death;
- Is a life-threatening event (i.e. the patient was at immediate risk of death at the time the reaction was observed);
- Requires hospitalization or prolongation of an existing hospitalization;
- Results in significant or persistent disability or incapacity;
- Is a congenital anomaly or birth defect;
- Is an important medical event (i.e. important adverse events that are not immediately life threatening or do not result in death or hospitalization but may jeopardize the patient or may require intervention to prevent one of the above characteristics/consequences, including suspected transmission of infectious agents by a medicinal product).

Suspected unexpected serious adverse reaction (SUSAR)

All suspected Adverse Reactions which occur in the trial and that are both unexpected and serious.
Suspected adverse reactions (AR) are those AEs of which a reasonable causal relationship to any dose administered of the investigational medicinal product and the event is suspected. Unexpected adverse reactions are adverse reactions, of which the nature, or severity, is not consistent with the applicable product information (e.g. Investigator’s Brochure for an unapproved IMP or Summary of Product Characteristics (SPC) for an authorized medicinal product).
An expected AE with a fatal outcome should be considered unexpected unless the Reference Safety Information specifically states that the AE might be associated with a fatal outcome.

12.2 Adverse event

Reporting of adverse events

Adverse events will be reported from the first study-related procedure until 30 days following the last dose of any drug from the protocol treatment schedule or until the start of subsequent systemic therapy for the disease under study, if earlier.
Adverse events occurring after 30 days should also be reported if considered at least possibly related to the investigational medicinal product by the investigator.
Adverse events have to be reported on the Adverse Events CRF. Adverse events will be scored according to the NCI Common Terminology Criteria for Adverse Events, version 5.0 (see appendix D).
Pre-existing conditions will be collected on the baseline concomitant diseases CRF, i.e. active (symptomatic) diseases of CTCAE grade ≥ 2 diseases under treatment, chronic diseases and long term effects of past events as present at the time of baseline assessment.

All adverse events have to be reported, **with the exception of**:

- ♦ A pre-existing condition that does not increase in severity; the pre-existing condition should be reported on the baseline concomitant diseases CRF
- ♦ Abnormal laboratory values that have been recorded as being not clinically significant by the investigator in the source documents
- ♦ Relapse/Progression of the disease under study; complications as a result of disease progression remain reportable adverse events

Anticipated disease-related Events

An anticipated disease-related event is an adverse event (serious or non-serious) that commonly occurs as a consequence of the underlying disease or condition under investigation (disease-related).
For the purposes of this study the following events will be considered disease-related adverse events, unless definitely related to another cause:

- Anaemia
- Bleeding
- Bone diseases
- Hypercalcemia
- Hyperuricemia
- Hyper viscosity syndrome
- Infection
- Neutropenia
- Renal failure or insufficiency
- Thrombocytopenia

Reporting of anticipated disease-related Adverse Events

All adverse events will be recorded in the eCRF regardless of whether considered to be anticipated disease-related events and will be reported to the sponsor. Any anticipated disease-related AE that meets serious adverse event criteria will be reported to the sponsor as described in the protocol section, Serious Adverse Events. Adverse events are considered unexpected when their nature or severity is not consistent with the reference safety information. If SAEs are unexpected and related to the study therapy, they are subject to expedited reporting as individual single cases to Health Authorities.
Anticipated Adverse Events that meet the criteria of Serious Adverse Events should be communicated within 24 hours same as per non- anticipated Serious AE.

Follow up of adverse events

All adverse events will be followed clinically until they have been resolved, or until a stable situation has been reached. Depending on the event, follow up may require additional tests or medical procedures as indicated, and/or referral to the general physician or a medical specialist.

Follow up information for grade 3 or 4 adverse events considered at least possibly related to the investigational medicinal product by the investigator should be reported on the AE CRF until recovery or until 6 months after the last dose of IMP, whichever comes first.
Follow up information for all other adverse events should be reported on the AE CRF until recovery or until 30 days after the last dose of any drug from the protocol treatment schedule, whichever comes first.

Adverse Event of Special Interest (AESI)

AESI in this trial consist of: grade 3-4 infusion associated reactions (IARs); pregnancies; overdoses; and second primary malignancies. An overdose, accidental or intentional, with the study treatment is an adverse event suspected by the Investigator or spontaneously notified by the patient –not based on systematic pills count– that is defined as an increase of at least 30% of the dose intended to be administered in the specified duration, or if the dose is administered in less than half the recommended duration of administration.

12.3 Serious Adverse Events

Reporting of serious adverse events

Serious Adverse Events (SAEs) will be reported from the first study-related procedure until 30 days following the last dose of any drug from the protocol treatment schedule or until the start of subsequent systemic therapy for the disease under study, if earlier.
Serious adverse events occurring after 30 days should also be reported if considered at least possibly related to the investigational medicinal product by the investigator.

SAEs must be reported to CRO/ EMN Data Center via e-CRF **within 24 hours** after the event was known to the investigator, using the SAE report form provided. This initial report should contain a minimum amount of information regarding the event, associated treatment and patient identification, as described in the detail in the instructions for the SAE report form. Complete detailed information should be provided in a follow-up report within a further 2 business days, if necessary
The following events do not require to be reported as a serious adverse event:

- Relapse/Progression of the disease under study; **death or complications as a result of disease progression remain reportable serious adverse events**.
- Hospitalization for protocol therapy administration. Hospitalization or prolonged hospitalization for a complication of therapy administration will be reported as a serious adverse event.
- Hospitalization for diagnostic investigations (e.g., scans, endoscopy, sampling for laboratory tests, bone marrow sampling) that are not related to an adverse event. Hospitalization or prolonged hospitalization for a complication of such procedures remains a reportable serious adverse event.

- Prolonged hospitalization for technical, practical, or social reasons, in absence of an adverse event.
- Hospitalization for a procedure that was planned prior to study participation (i.e. prior to registration or randomization). This should be recorded in the source documents. Prolonged hospitalization for a complication of such procedures remains a reportable serious adverse event.

Causality assessment of Serious Adverse Events

The investigator will decide whether the serious adverse event is related to trial medication, i.e. any of the products from the protocol treatment schedule. The decision will be recorded on the serious adverse event report. The assessment of causality is based on likelihood of relations, temporal relation with the event and exclusion of other causes and is made by the investigator using the following:

RELATIONSHIP	DESCRIPTION
UNRELATED	There is no evidence of any causal relationship
UNLIKELY	There is little evidence to suggest there is a causal relationship (e.g. the event did not occur within a reasonable time after administration of the trial medication). There is another reasonable explanation for the event (e.g. the patient's clinical condition, other concomitant treatments).
POSSIBLE	There is some evidence to suggest a causal relationship (e.g. because the event occurs within a reasonable time after administration of the trial medication). However, the influence of other factors may have contributed to the event (e.g. the patient's clinical condition, other concomitant treatments).
PROBABLE	There is evidence to suggest a causal relationship and the influence of other factors is unlikely.
DEFINITELY	There is clear evidence to suggest a causal relationship and other possible contributing factors can be ruled out.
NOT ASSESSABLE	There is insufficient or incomplete evidence to make a clinical judgment of the causal relationship.

Adverse events that have a reasonable possibility of a causality relationship (possible, probable, definitely) should be considered as Adverse reaction (AR) and or Serious Adverse Reaction (SAR).

Follow up of Serious Adverse Events

All serious adverse events will be followed clinically until they are resolved or until a stable situation has been reached. Depending on the event, follow up may require additional tests or medical procedures as indicated, and/or referral to the general physician or a medical specialist.

Follow up information on SAE's should be reported monthly until recovery or until a stable situation has been reached. The final outcome of the SAE should be reported on a final SAE report.

Processing of serious adverse event reports

The EMN safety desk or its delegate will forward all SAE reports within 24 hours of receipt to the Principal Investigator, Sanofi and Amgen
The safety desk and medical monitor will evaluate if the SAR qualifies as a suspected unexpected serious adverse reaction (SUSAR).
The IB for Isatuximab will be used as a reference document for expectedness assessment. The SmPC for Carfilzomib, Lenalidomide and Dexamethasone will be used.
Where reporting of SAE's to the Ethics Committee is required by national laws or regulations or by the procedures of the Ethics Committee, EMN Data Center will report those SAE's by means of a six-monthly SAE line listing.

12.4 Reporting Suspected Unexpected Serious Adverse Reactions

The EMN Safety Desk will ensure the reporting of any SUSARs to the Ethics Committees (EC), the Competent Authorities (CA), Sanofi and Amgen and the investigators in compliance with applicable laws and regulations, and in accordance with any trial specific agreements between the sponsor and a co-sponsor.

Expedited reporting of SUSARs will occur no later than 15 days after CRO/EMN Data Center had first knowledge of the serious adverse event. For fatal or life-threatening cases this will be no later than 7 days for a preliminary report, with another 8 days for a complete report.
The manner of SUSAR reporting will be in compliance with the procedures of the Ethics Committees and Health Authorities involved.

12.5 Pregnancies

Pregnancies of a female subject or the female partner of a male subject, occurring while the subject is on protocol treatment or within 30 days following the last dose of any drug from the protocol treatment schedule, should be reported to the sponsor. Pregnancies must be reported to CRO/EMN Data Center via e-CRF within 24 hours after the event was known to the investigator, using the pregnancy report form provided. Amgen will be sent any reports /outcomes associated to pregnancy or lactation exposure to the Amgen drug that are considered serious within 1 business day of Sponsor awareness and any reports/outcomes associated to pregnancy or lactation exposure to the Amgen drug that are considered non-serious, not to exceed 15 calendar days of Sponsor awareness.

The investigator will follow the female subject until completion of the pregnancy and must notify the sponsor of the outcome of the pregnancy within 5 days or as specified below. Additionally, for

pregnancies with potential exposure to lenalidomide infants must be followed for 1 year after birth. The investigator will provide this information as a follow-up to the initial pregnancy report. If the outcome of the pregnancy meets the criteria for classification as a SAE (i.e., spontaneous or therapeutic abortion, stillbirth, neonatal death, or congenital anomaly - including that in an aborted fetus), the investigator should follow the procedures for reporting SAEs. In the case of a live “normal” birth, the sponsor should be informed as soon as the information is available. All neonatal deaths that occur within 30 days of birth should be reported, without regard to causality, as SAEs. In addition, any infant death after 30 days that the investigator suspects is related to the *in utero* exposure to the investigational medicinal product(s) should also be reported.

The investigator is encouraged to provide outcome information of the pregnancy of the female partner of a male subject, if this information is available to the investigator and the female partner gives her permission.

12.6 Second Primary Malignancies

Second primary malignancies (SPM) will be monitored as events of special interest and must be reported as serious adverse events. This includes any second primary malignancy, regardless of causal relationship to any study drug, occurring at any time for the duration of the study, from the time of signing informed consent until 5 years after registration in the trial or until completion of maintenance therapy for patients who are still on maintenance at 5 years after registration.

Events of second primary malignancy are to be reported using the SAE report form and must be considered an “Important Medical Event” even if no other serious criteria apply. Documentation on the diagnosis of the second primary malignancy must be provided at the time of reporting as a serious adverse event (e.g. pathology report).

The incidence of second primary malignancies is also monitored via a separate form (Second Primary Malignancy Report Form). This form should be filled out, dated and signed by the responsible investigator and filled in the eCRF within 24 hours after establishment of a second primary malignancy. SPM must also be documented in the other appropriate page(s) of the CRF (e.g. Adverse Event Form and Follow up Form).

12.7 Reporting of safety issues

The sponsor will promptly notify all concerned investigators, the Ethics Committee(s) and the regulatory authorities of findings that could affect adversely the safety of patients, impact the conduct of the trial, increase the risk of participation or otherwise alter the EC's approval to continue the trial.

In the occurrence of such an event the sponsor and the investigators will take appropriate urgent safety measures to protect the patients against any immediate hazard. The local investigator will inform the patients and local ethics or review committees according to hospital policy. The sponsor will inform any other parties that are involved in the trial.

12.8 Annual safety report

The sponsor will submit once a year a safety report to the Ethics Committees and Competent Authorities of the concerned Member States. The first report is sent one year after the first approval date of the trial. Subsequent reports are sent annually until end of trial. The content of the annual safety report will be according to the EU guidance document.
Before issuing the final version of the report, the sponsor shall provide draft copy of the report to Sanofi and Amgen for review and comments be provided no later than day 45 after data lock point. Sanofi and Amgen will review and send back comments no later 1 calendar week after receipt. A final copy of any Periodic reports (e.g. Development Safety Update Report (DSUR)), shall be transmitted to Sanofi and Amgen at the time of submission to Regulatory Authority.

12.9 Independent Data Monitoring Committee

An IDMC, consisting of 2 clinicians and 1 statistician who are independent experts not otherwise participating in the study, will be established to review safety results (see section 14.3 - interim analysis) In addition, the IDMC will review cumulative safety data on a regular basis before the primary MRD analysis and will also review efficacy results. After each of these reviews, the IDMC will make recommendations regarding the continuation of the study. The details will be provided in a separate IDMC charter.

12.10 Product Complaints

Please also inform the EMN Data Center of your complaint. Note that product complaints in and of themselves are not AEs. If a product complaint results in a SAE, a SAE form should be completed and sent to EMN Safety Office according to the SAE reporting timelines. Sponsor shall provide Amgen with PCs related to Amgen's drug, whether associated with an ADR or not, immediately but not to exceed one (1) business day of the Sponsor's awareness.

13. Endpoints

13.1 Primary endpoint – Rate of MRD negativity after ASCT consolidation treatment

The rate of MRD negativity is determined as the proportion of patients with MRD negativity ($\geq 10^{-5}$ sensitivity level) after ASCT consolidation treatment using ITT principle. For patients who withdraw from the study or are lost to follow up before four post ASCT consolidation cycles, the best MRD assessment will be considered. Patients will be classified as MRD positive if they have only MRD positive test results or do not undergo MRD assessment.

13.2 Secondary endpoints

Key secondary endpoints

The rate of MRD negativity after induction is determined as the proportion of patients with MRD negativity (≥10^{-5} sensitivity level, NGS) after the induction phase using ITT principle. Patients will be classified as MRD positive if they have only MRD positive test results or do not undergo MRD assessment/sample not adequate.

PFS will be measured from the date of randomization to the date of first observation of PD, or death from any cause as an event. Subjects who have not progressed or who withdraw from the study will be censored at the time of the last complete disease assessment. All subjects who were lost to FU will also be censored at the time of last complete disease assessment

Other secondary endpoints

The rate of MRD negativity after light consolidation is determined as the proportion of patients with MRD negativity (≥10^{-5} sensitivity level, NGS) after light consolidation phase using ITT principle. Patients will be classified as MRD positive if they have only MRD positive test results or do not undergo MRD assessment/sample not adequate. Patients who withdraw from the study or are lost to follow up before MRD evaluation, the best MRD assessment will be considered.

Rate of 1 year sustained MRD negativity by NGS (from post ASCT consolidation to post light consolidation) will be also evaluated.

Response rate (sCR, CR, VGPR, PR, ORR) will be evaluated according to IMWG Response criteria after induction, ASCT, post ASCT consolidation and light consolidation

PFS2 will be measured from the date of randomization to the date of observation of second disease progression (i.e. progression after the second line of therapy) or death to any cause as an event. In case of date of second progression is not available, date of start of third line treatment can be used. Subjects who have not progressed or who withdraw from the study will be censored at the time of the last complete disease assessment. All subjects who were lost to follow-up prior to the end of the study, have not progressed, and are still alive will also be censored at the time of last contact.

TTP will be measured from the date of randomization to the date of first observation of PD, or deaths for PD. Subjects who have not progressed or who withdraw from the study or die from causes other than PD will be censored at the time of the last complete disease assessment. Subjects lost to FU will also be censored at the time of last complete disease assessment.

DOR is defined as time between first documentation of response (achievement of at least a PR) and PD with deaths owning to causes other than progression not counted, but censored. Responders without disease progression at the cut-off date of final analysis will be censored either at the time of lost to FU, at the time of death due to other cause than PD, or at the at the time of last contact.

OS is defined as the time between randomization and death, regardless cause of death. Subjects who withdraw consent will be censored at the time of withdrawal. Subjects who are still alive at the cut-off date of final OS analysis will be censored at the cut-off date. Subjects lost to FU will also be censored at the time of last contact.

TNT will be measured from the date of randomization to the date of next anti-myeloma therapy. Death due to any cause before starting therapy will be considered an event. Subjects who have not progressed or who withdraw from the study will be censored at the time of the last complete disease assessment. Subjects lost to FU will also be censored at the time of last contact.

Determine safety in the 2 treatment arms (through the analysis of AEs and laboratory abnormalities) in the different phases

Determine whether tumor response and outcome (PFS, PFS2, TTP, TNT and OS) may change in subgroups with different prognosis according to current prognostic factors.

The rate of MRD negativity after ASCT is determined as the proportion of patients with MRD negativity (≥10^{-5} sensitivity level), NGS using ITT principle. For patients who withdraw from the study or are lost to follow up before ASCT, the best MRD assessment will be considered. Patients will be classified as MRD positive if they have only MRD positive test results or do not undergo MRD assessment.

The rate of MRD negativity (by NGF) after induction, ASCT, post ASCT consolidation and light consolidation are determined as the proportion of patients with MRD negativity (≥10^{-5} sensitivity level) after the specific phase using ITT principle. Patients will be classified as MRD positive if they have only MRD positive test results or do not undergo MRD assessment. Patients who withdraw from the study or are lost to follow up before MRD evaluation phase, the best MRD assessment will be considered.

The duration of MRD Negativity (by NGS and NGF) is defined as time between first MRD Negativity and first MRD positivity. Patients without MRD positivity will be censored at last complete assessment.

Determine the rate of sustained for 1-year MRD negativity (by NGF) (from post ASCT consolidation to post light consolidation)

Determine the success of stem cell harvest according to baseline characteristics; stem cells will be harvested at a minimum of 4 x 10^6 CD34+ cells/kg

Determine the success of engraftment after ASCT, defined by the time needed to achieve:
Absolute Neutrophil Count (ANC): 3 consecutive days with at least ANC 0.5 10^9/L (the last day)
Platelet count: 7 consecutive days with at least Platelet count 20 10^9/L without transfusion or 3 consecutive days with at least Platelet count 50 10^9/L without transfusion (the last day).

Quality of life defined by EORTC QLQ-C30, EORTC QLQ-MY20 and EQ5D-5L.

14. Statistical considerations

14.1 Patient numbers and power considerations

The calculation of the sample size for primary endpoint was done with the following assumptions, consider ITT population:

- α= 0.05 (two sided)
- β= 0.10
- Allocation ratio: 1:1
- post ASCT consolidation MRD negativity (10^{-5}) (NGS) rate ARM A(Isa-KRd): 64%
- post ASCT consolidation MRD negativity (10^{-5}) (NGS) rate ARM B(KRd): 45%

The total number of patients required is 300 (by the X2 test with Yates' continuity correction).

A hierarchical testing procedure (using the Fixed-Sequence Method) will be used for the key secondary endpoints to achieve control of the overall familywise Type I error rate at a two-sided significance level of 0.05. The details of the testing
procedure will be prespecified in multiplicity section.

The power of 85% (β= 0.15) for first key endpoint (MRD negativity rate after induction by NGS) was done with the following assumptions by the X2 test with Yates' continuity correction, consider ITT population:

- α= 0.05 (two sided)
- Allocation ratio: 1:1
- post induction MRD negativity (10^{-5}) (NGS) rate ARM A(Isa-KRd): 30%
- post induction MRD negativity (10^{-5}) (NGS) rate ARM B(KRd): 15%

The power of 90% (β= 0.10) for second key endpoint (PFS) was done with the following assumptions by Schoenfeld formula, consider ITT population:

- α= 0.05 (two sided)
- Allocation ratio: 1:1
- 48 months PFS ARM A(Isa-KRd): 83%
- 48 months PFS ARM B(KRd): 69% (HR:0.50)
- Accrual time: 10 months
- Minimum follow-up time: 71 months
- Lost to follow-up: 5%/year exponentially distributed

To achieve 90% power, 91 PFS events are needed, expected after 71 months of last randomized patient. The PFS event count takes into account the fifth interim analysis (PFS) for superiority and non-binding futility.

The OS analysis will be performed upon reaching 77 events or at 9 years after the last subject is randomized (approximately 77 deaths), whichever occurs first. This will provide at least 39% power to detect a reduction of the risk of death (HR = 0.68, 4 years OS in the control arm 86%) with a 2-sided alpha of 0.05 by Schoenfeld formula.

14.2 Statistical analysis

All main analyses will be according the intention to treat principle, restricted to eligible patients.
Patients initially registered but considered ineligible afterwards based on information that should have been available before registration, will be excluded from all analyses.
Descriptive statistics (median with the interquartile range) will be calculated for continuous variables. For qualitative variables, absolute frequencies and percentages will be provided. Summary statistics will be presented according to treatment arms.

Multiplicity

A hierarchical testing procedure will be used for primary (H1) and key secondary endpoints (H2 as MRD and H3 as PFS) to achieve control of the overall familywise Type I error rate at a two-sided significance level of 0.05.

H1 will be tested at the two-sided 0.05 alpha level; if H1 fails not other test will be performed. If H1 will be significant, H2 will be tested at the two-sided 0.05 alpha level; if H2 fails not other test will be performed. If H2 will be significant, H3 will be tested at the two-sided 0.05 alpha level.

Efficacy analysis

For the main analysis on MRD Negativity rate, logistic regression model will be used to compare the two arms, with adjustment to the stratification factor (ISS Stage I vs II vs III and cytogenetic risk FISH high-risk (defined as having t(4;14), t(14;16) and/or del17p) vs low risk (not having these FISH abnormalities)/ missing)) to estimate adjusted odds ratios (ORs), the 95% confidence intervals (CIs) and p value.

Time-to-event endpoints will be analyzed comparing survival curves of the two arms, using the Kaplan–Meier method; median, 95% Confidence Interval (CI) and probability will be summarized according to treatment arms. Arms will be compared with the log-rank test. The Cox proportional hazards models adjusted for stratification factor will be used to estimate adjusted hazard ratios (HRs), the 95% confidence intervals (CIs) and 2-sided p value. Grambsch and Therneau test will be used for testing the proportional hazard assumption (44).

Proportions will be compared between treatment groups by using Pearson's chi-square test or Fisher's exact test or logistic regression model.
The analysis of primary endpoint will be conducted when data about cycles 1-4 of consolidation therapy will be available.
The analysis of first key secondary endpoint will be conducted at the same time of primary endpoint.
In the fifth interim analysis (PFS), the analysis of second key secondary endpoint of PFS will be conducted for superiority and non-binding futility when the approximately 68 PFS event (corresponding to 75% information fraction) is achieved (46 months from last randomized patient).
The analysis of secondary endpoints will also be conducted in the fifth interim analysis when second key secondary endpoint (PFS) will be tested.
Primary analysis of PFS when the total of 91 events are observed.
The OS analysis will be performed upon reaching 77 events or at 9 years from last randomized patient (approxaimetely 77 deaths), whichever occurs first.

Toxicity analysis

In the safety analysis all randomized patients who received at least one dose of study treatment will be included. The analysis of safety will be done primarily by tabulation of the incidence of Adverse Events reported by CTCAE Grade, Serious Adverse Event and relation with study drugs. In the by-subject analysis, a subject having the same event more than once will be counted only once. Adverse events will be summarized by worst CTCAE grade.
Safety analysis will be done in all different treatment phases.

Additional analyses

Subgroup analysis will be analyzed using interaction term between treatment arm and prognostic factors, in addition to stratification factor.
A forest plot will be provided based on the HRs or ORs for each subgroup.
Subgroup analysis will be performed for primary endpoint with logistic regression model and for secondary endpoints (PFS, PFS2, TTP, TNT and OS) with cox models.

Statistical analysis plan

A Statistical Analysis Plan (SAP) will be prepared by the trial statistician and approved by the principal investigator before each interim analysis and each analysis to be tested. It will describe in detail the analyses to be performed.

14.3 Interim analysis

One interim analysis is planned, primarily to describe adverse events observed during induction. This analysis will be conducted when complete data of the first 75 patients regarding the 4 cycles of induction therapy are available. Data on stem cell collection, after mobilization, will be also analyzed after the first 75 patients completed that phase (second interim analysis).

A third interim analysis is planned, primarily to describe adverse events observed during post ASCT consolidation. This analysis will be conducted when complete data of the first 75 patients regarding the 4 cycles of post ASCT consolidation therapy are available.

A fourth interim analysis is planned, primarily to describe adverse events observed during light-consolidation. This analysis will be conducted when complete data of the first 75 patients regarding the 4 cycles of light-consolidation therapy are available.

Since safety is not the principal aim of the study, for interim analysis 1-4 no statistical correction of the sample size or of the alpha error have been done.

A fifth interim analysis is planned for PFS. The interim analysis will be performed when approximately 68 PFS events have occurred (it corresponds to 75% of the total planned PFS events). The significance level for superiority and futility (non-binding) at the interim analyses for PFS will be determined based on the observed number of PFS events at the interim analysis, using the O'Brien and Fleming alpha spending function. The 2-sided alpha to be spent at the interim is 0.012, if 68 events will be observed as planned.

Results of interim analyses will be presented to the principal investigators and to an independent data monitoring committee.

15. Registration and Randomization

15.1 Regulatory Documentation

Required regulatory and administrative documents must be provided to the EMN Data Center before registration of the first patient. This will always include an Ethics Committee approval for the investigational site. The EMN Data Center will provide each investigator with an overview of the required documents. Each investigational site will be notified when all requirements are met and enrolment can start.

15.2 Registration and Randomization

Eligible patients should be registered in eCRF before start of treatment.

The following information will be requested at registration:

- Sex
- Year of birth
- Date written informed consent
- Specific items patient gives consent for (see ICF)
- Eligibility criteria
- Stratification factors (ISS Stage and cytogenetic risk FISH)

All patients eligible for randomization can be randomized in a 1:1 ratio to receive Isatuximab - carfilzomib-lenalidomide-dexamethasone (Isa-KRd – Arm A) or carfilzomib-lenalidomide-dexamethasone (KRd – Arm B).

Patients will be stratified according to ISS Stage [3 levels: I vs II vs III] and cytogenetic risk FISH [2 levels: high-risk (defined as having t(4;14), t(14;16) and/or del17p) vs low risk/missing (not having these FISH abnormalities)] and then randomized using a web-based, computer generated, procedure completely concealed to study participants.

Randomization will be performed according to a randomization list provided by statisticians. In particular, the randomization list is created using dynamic sizes of blocks, from 2 to 6, for each stratum. Each patient will be given a unique patient study number (a sequence number according to order of registration in the trial).

16. Data collection and quality assurance

16.1 Case Report Forms

Data will be collected on electronic Case Report Forms (CRF) to document eligibility, safety and efficacy parameters, compliance to treatment schedules and parameters necessary to evaluate the study endpoints. Data collected on the CRF are derived from the protocol and will include at least:

- Inclusion and exclusion criteria;

- ♦ Baseline status of patient including medical history and stage of disease;
- ♦ Timing and dosage of protocol treatment;
- ♦ Baseline concomitant diseases and adverse events;
- ♦ Parameters for response evaluation;
- ♦ Any other parameters necessary to evaluate the study endpoints;
- ♦ Survival status of patient;
- ♦ Reason for end of protocol treatment.

Each CRF page will be identified by a trial number, and a combination of patient study number (assigned at registration) and hospital name.

The e-CRF will be completed on site by the local investigator or sub-investigator or an authorized staff member. The CRF must be signed by the local investigator or sub-investigator upon completion by means of an electronic signature. All CRF entries must be based on source documents.

16.2 Data quality assurance

Steps to be taken to ensure the accuracy and reliability of data include the selection of qualified investigators and appropriate study centers, review of protocol procedures with the investigator before the study, and site visits by the sponsor.

Data collected on the CRF will be verified for accuracy. If necessary, queries will be sent to the investigational site to clarify the data on the CRF. The investigator should answer data queries within the specified time line.

17. Ethics

17.1 Accredited ethics committee

An accredited Ethics Committee will approve the study protocol and any substantial amendment.

17.2 Ethical conduct of the study

The study will be conducted in accordance with the ethical principles of the Declaration of Helsinki, the ICH-GCP Guidelines, the EU Clinical Trial Directive (2001/20/EG), and applicable regulatory requirements. The local investigator is responsible for the proper conduct of the study at the study site.

17.3 Patient information and consent

Written informed consent of patients is required before registration in the trial and before any study related procedure takes place.

The investigator will follow ICH-GCP and other applicable regulations in informing the patient and obtaining consent. The investigator should take into consideration if the patient is capable of giving informed consent. Before informed consent may be obtained, the investigator should provide the patient

ample time and opportunity to inquire about details of the trial and to decide whether or not to participate in the trial. All questions about the trial should be answered to the satisfaction of the patient.
There is no set time limit for the patient to make a decision. The investigator should inform each patient if there is a specific reason why he/she must decide within a limited time frame, for example if patients condition necessitates start of treatment or if the trial is scheduled to close for enrolment.

The content of the patient information letter, informed consent form and any other written information to be provided to patients will be in compliance with ICH-GCP and other applicable regulations and should be approved by the Ethics Committee in advance of use.
The patient information letter, informed consent form and any other written information to be provided to patients will be revised whenever important new information becomes available that may be relevant to the patient's consent. Any substantially revised informed consent form and written information should be approved by the Ethics Committee in advance of use. The patient should be informed in a timely manner if new information becomes available that might be relevant to the patient's willingness to continue participation in the trial. The communication of this information should be documented.

17.4 Benefits and risks assessment

Please refer to the Investigator's Brochure for preclinical data of isatuximab administered in monotherapy and in combination with proteasome inhibitors or immunomodulatory agents and for clinical data of isatuximab in as single agent and in combination.
Isatuximab (SAR650984) is a chimeric monoclonal antibody that binds selectively to a unique epitope on the human surface antigen CD38. Isatuximab's cytotoxic properties are derived from multiple biological mechanisms, antibody-dependent cellular-mediated cytotoxicity, complement-dependent cytotoxicity, direct induction of apoptosis (pro-apoptosis) without crosslinking, and inhibition of CD38 enzymatic activity. While the predominant potential toxicities may be severe in some cases, they are largely reversible and can be managed by routine clinical monitoring and standard medical interventions, which may include dose reductions and supportive care.
The most frequent AEs reported to date in combination with lenalidomide and or proteasome inhibitor were hematological toxicities (neutropenia, leukopenia, lymphopenia, anemia and thrombocytopenia), infusion related reactions, diarrhea, fatigue, upper respiratory tract infection, nausea, insomnia, pyrexia, dyspnea, cough, headache, muscle spasms, vomiting and nasal congestion. For more details please refer to Investigator's Brochure. However, it is possible that Isatuximab will have toxicities that were not previously observed in or predicted from such sources. Patients will be monitored closely for anticipated toxicities.

In the ongoing phase II GMMG-CONCEPT trial (NCT03104842) in patients with primary diagnosed high-risk MM aiming to evaluate the MRD negativity after consolidation, isatuximab is administered in combination with carfilzomib, lenalidomide and dexamethasone. 90% of the patients completed 6 cycles

of induction and all the patients had documented responses during induction phase, since all patients achieved ≥ VGPR. Results referred referring to the 4-drug combination of IKRd indicate that toxicity was manageable with an overall safety profile consistent with prior experience with KRd and anti-CD38 antibody treatment and that the preliminary response rates are encouraging. Despite the fact that the control arm carfilzomib, lenalidomide, and dexamethasone (KRd) is not standard of care in first line treatment of multiple myeloma, this is a well-known combination and already standard of care in second line treatment. Furthermore, KRd has been studied in newly diagnosed multiple myeloma patients, as described in the introduction and rational section 4.1.

Potential subjects will be fully informed of the risks and requirements of the study and, during the study, subjects will be given any new information that may affect their decision to continue participation. They will be informed that their consent to participate in the study is voluntary and may be withdrawn at any time with no reason given and without penalty or loss of benefits to which they would otherwise be entitled. Only subjects who are fully able to understand the risks, benefits, and potential AEs of the study, and provide their consent voluntarily will be enrolled.

Blood volumes drawn and all the study procedure for all phases of the study are provided in section 10.2. The total blood volume to be collected is considered to be acceptable for subjects participating in a cancer clinical study and reasonable over the time frame of the study.

Bone marrow aspiration is an invasive, potentially painful examination, however with a low risk of complication. Bone marrow aspirations will be drawn at multiple time points: after induction, after ASCT, after consolidation and after light consolidation as well as at progression of disease. These include 3 aspirations more than the ones performed outside a trial. These bone marrow aspirations will be done to measure minimal residual disease (MRD). MRD is the most important parameter in the study, with the first endpoint being MRD after consolidation. MRD can only be determined in the bone marrow through next generation sequencing and flow cytometry, achieving a sensitivity of at least 10^{-5}. MRD after induction to evaluate induction is one of the secondary endpoints, as well as MRD after light consolidation. MRD and persistent MRD are predictors for PFS and OS. Newly diagnosed multiple myeloma patients nowadays show a median PFS of around 4-6 years on first line treatment, accordingly PFS results will take a longer time to be available. Therefore MRD measurements in bone marrow are used as a prompt and clear-cut method to define the most effective treatment strategy.

Patients will be informed in advance about these extra bone marrow aspirations, about possible complications including pain of the used method, and possible development of a hematoma. These bone marrow examinations for MRD are described as an essential part of the study. However patients have the right to refuse a bone marrow aspiration. Refusing to undergo these extra bone marrow examinations will be discussed internally on a case by case basis on how to proceed. Blood volumes drawn and all the study procedures for all phases of the study are provided in section 10.2. The total blood volume to be collected is considered to be acceptable for subjects participating in a cancer clinical study and reasonable over the time frame of the study.

PET-CTs are not obligatory, but in case of extramedullary plasmacytoma or if used at baseline as screening method for osteolytic lesions, should be repeated at least after each treatment phase. Moreover, PET-CT data proved to provide additional prognostic information, as well as they proved to be informative for response rate, with addition data if compared with bone marrow MRD evaluation (50).

This study will be conducted in compliance with the protocol, good clinical practice (GCP), applicable regulatory requirements, and International Conference on Harmonization (ICH) guidelines.

17.5 Trial insurance

Prior to the start of the trial, the sponsor will ensure that adequate insurance for patients is in place covering losses due to death or injury resulting from the trial, in accordance with applicable laws and regulations in each country where the trial is conducted. The sponsor will take out an insurance policy or delegate this responsibility to a national co-sponsor. Proof of insurance will be submitted to the Ethics Committee.
In addition, the sponsor will ensure that adequate insurance is in place for both investigator(s) and sponsor to cover liability pertaining to death or injury resulting from the trial.

18. Administrative aspects and publication

18.1 Personal data protection

Sponsor in order to prevent unauthorised access, disclosure, dissemination, modification or loss of the information and personal data processed, has implemented measures relating to, inter alia:

- use of the PC,
- network use,
- authentication management,
- use of magnetic media,
- use of electronic mail,
- Internet use and related services,
- network and device security,
- data encryption,
- document security and paper archives,
- anti-virus and anti-malware software,
- staff education and training,
- adoption of corporate data protection regulations.

Sponsor has implemented measures to ensure the confidentiality of the data and personal data of the subjects involved in the clinical trials, as well as the security of the processing such as, for example

- the pseudonymisation and encryption of personal data;
- the ability to ensure the confidentiality, integrity, availability and resilience of processing systems and services on an ongoing basis;
- the ability to promptly restore the availability and access of personal data in the event of a physical or technical incident (e.g. Backup and Disaster Recovery Plan…);
- a procedure to verify the effectiveness of the technical and organisational measures to ensure the security of the processing (e.g. Audit, Penetration Test…).

In the event of a data security breach, Sponsor, in order to mitigate possible adverse events, puts in place measures such as:

- total system isolation
- verification of access to systems
- verification of updates and consequent vulnerabilities of the systems
- verification of the correct use of the company's systems.

18.2 Handling and storage of data and documents

Patient confidentiality

Each patient is assigned a unique patient study number at registration. In trial documents the patient's identity is coded by patient study number as assigned at registration.
The local investigator will keep a subject registration and identification log that contains the key to the code, i.e. a record of the personal identification data linked to each patient study number. This record is filed at the investigational site and should only be accessed by the investigator and the supporting hospital staff, and by representatives of the sponsor or a regulatory agency for the purpose of monitoring visits or audits and inspections.

Filing of essential documents

Essential Documents are those documents that permit evaluation of the conduct of a trial and the quality of the data produced. The essential documents may be subject to, and should be available for, audit by the sponsor's auditor and inspection by the regulatory authority(ies)
The investigator should file all essential documents relevant to the conduct of the trial on site. The sponsor will file all essential documents relevant to the overall conduct of the trial. Essential documents should be filed in such a manner that they are protected from accidental loss and can be easily retrieved for review.

Record retention

In compliance with the ICH/GCP guidelines, the investigator/institution will maintain all CRFs and all source documents that support the data collected from each patient, as well as all study documents as specified in ICH/GCP Section 8, Essential Documents for the Conduct of a Clinical Trial, and all study

documents as specified by the applicable regulatory requirement(s). The investigator/institution will take measures to prevent accidental or premature destruction of these documents.
Essential documents (paper and electronic) should be retained for a period not shorter than 25 years after the end of the trial, unless the Sponsor provides written permission to dispose of them earlier or requires their retention for an additional period of time, because of applicable laws, regulations and/or guidelines. The patients' medical files will be archived in accordance with the national laws. If the responsible investigator retires, relocates, or for other reasons withdraws from the responsibility of keeping the study records, custody must be transferred to a person who will accept the responsibility.

Storage of samples

Biological samples should only be stored for the purpose of additional research if the patient has given consent. If no informed consent was obtained, samples should be destroyed after the patient has completed all protocol treatment and procedures, and the samples are no longer needed for the study. Storage of biological samples on site is subject to the site's guidelines; samples may be labeled with the patients identifying information (e.g. name, hospital record number)
Samples that are shipped to another facility (e.g. a central laboratory) for a purpose as described in this protocol or for additional scientific research, should be stripped from any identifying information and labeled with a code (trial name or number and patient study number as assigned at registration).

18.3 Amendments

A 'substantial amendment' is defined as an amendment to the terms of the Ethics Committee application, or to the protocol or any other supporting documentation, that is likely to affect to a significant degree:

- the safety or physical or mental integrity of the patients of the trial;
- the scientific value of the trial;
- the conduct or management of the trial; or
- the quality or safety of any intervention used in the trial.

All amendments will be submitted to the Ethics Committee and to the Competent Authority.

18.4 Annual progress report

The sponsor will submit a summary of the progress of the trial to the accredited Ethics Committee once a year. The first report is sent one year after the first approval date of the trial. Subsequent reports are sent annually until end of trial. Information will be provided on the date of inclusion of the first patient, numbers of patients included and numbers of patients that have completed the trial, serious adverse events/ serious adverse reactions, other problems, and amendments.

18.5 End of trial report

The sponsor will notify the accredited Ethics Committee and the Competent Authority of the end of the trial within a period of 90 days, or according to local regulation. The end of the study is defined as the last patient's last visit.

In case the study is ended prematurely, the sponsor will notify the accredited Ethics Committee and the competent authority within 15 days or according to local regulation, including the reasons for the premature termination.

Within one year after the end of the trial, the sponsor will submit an end of study report with the results of the study, including any publications/abstracts of the study, to the accredited Ethics Committee and the Competent Authority. Sponsor shall provide Amgen with a final submission copy of the end of Study Report no later than one (1) calendar year after Study completion.

18.6 Publication policy

Trial results will always be submitted for publication in a peer reviewed scientific journal regardless of the outcome of the trial – unless the trial was terminated prematurely and did not yield sufficient data for a publication.

All and any publications of (interim) trial results are subject to the EMN Publication Policy.

19. Glossary of abbreviations

(in alphabetical order)
Add and remove as applicable

AE	Adverse Event
AESI	Adverse Event of Special Interest
AL	Amyloid Light-chain
ALT	Alanine Aminotransferase
ASCT	Autologous Stem Cell Transplantation
AST	Aspartate Transaminase
ANC	Absolute Neutrophil Count
BJ	Bence Jones
BM	Bone Marrow
Ca	Calcium
CA	Competent Authority
CKTO	Commissie voor Klinisch Toegepast Onderzoek'
CR	Complete Remission
CRi	Complete Remission with incomplete blood count recovery
CRF	Case Report Form
CrCl	Creatinine clearance
CRP	C-Reactive Protein
CTCAE	Common Terminology Criteria for Adverse Events
DFS	Disease Free Survival
DOR	Duration of Response
ECHO	Echocardiogram
ECG	Electrocardiogram
ECOG	Eastern Cooperative Oncology Group
EBMT	European Group for Blood and Marrow Transplantation
EFS	Event Free Survival
EMN	European Myeloma Network
FFS	Failure Free Survival
FISH	Fluorescence In Situ Hybridization
FLC	Free Light Chain
GCP	Good Clinical Practice
G-CSF	Granulocyte-Colony Stimulating Factor
GI	Gastro-intestinal
Hb	Hemoglobin
HIV	Human Immunodeficiency Virus
HLA	Human Leukocyte histocompatibility Antigen
ICH	International Conference on Harmonization of technical requirements for registration of pharmaceuticals for human use
IDMC	Independent Data Monitoring Committee
IMID	Immunomodulatory drug
IMP	Investigational Medicinal Product
ISS	International Staging System
ITT	Intention To Treat
IU	International Units
KCl	Potassium chloride
LDH	Lactate Dehydrogenase
LFEV	Left Ventricular Ejection Fraction
METC	Medical Ethical Review Committee
MM	Multiple Myeloma
MRD	Minimal Residual Disease
MUGA	Multigated Acquisition Scan

NaCl	Sodium Chloride
NCI	National Cancer Institute
NGF	Next Generation Flow
NGS	Next Generation Sequencing
NYHA	New York Heart Association
ORR	Overall Response Rate
OS	Overall Survival
PB	Peripheral Blood
PD	Progressive Disease
PET	Positron Emission Tomography
PFS	Progression Free Survival
PFS2	Progression Free Survival 2
PO	Per Os
PPP	Pregnancy Prevention Plan
PR	Partial Response
QoL	Quality of Life
SAE	Serious Adverse Event
SAP	Statistical Analysis Plan
SC	Subcutaneous
sCR	Stringent Complete Response
SD	Stable Disease
SPEP	Serum protein electrophoresis
SUSAR	Suspected Unexpected Serious Adverse Reaction
TMA	Tissue Micro Array
TNT	Time to next treatment
TTP	Time to progression
ULN	Upper Limit of Normal
UPEP	Urine protein electrophoresis
VAD	Vincristine, Doxorubicin (Adriamycin), Dexamethasone
VGPR	Very Good Partial Response
WHO	World Health Organization
WMO	Wet Medisch-Wetenschappelijk Onderzoek met mensen

20. References

1. Altekruse SF, Kosary CL, Krapcho M, et al. (eds).SEER Cancer Statistics Review, 1975-2007, National Cancer Institute. Bethesda, MD,http://seer.cancer.gov/csr/1975_2007/, based on November 2009 SEER data submission, posted to the SEER website 2010
2. Palumbo A, Anderson K, Multiple Myeloma, N Engl J Med, 2011; 364:1046-60.
3. Rajkumar SV, Dimopoulos MA, Palumbo A, et al. International Myeloma Working Group updated criteria for the diagnosis of multiple myeloma. Lancet Oncol. 2014 Nov;15(12):e538-48
4. Moreau P, San Miguel J, Sonneveld P, et al. ESMO Guidelines Committee. Multiple myeloma: ESMO Clinical Practice Guidelines for diagnosis, treatment and follow-up. Ann Oncol. 2017 Jul 1;28(suppl_4):iv52-iv61.
5. Demo SD, Kirk CJ, Aujay MA, et al. Anti-tumor activity of PR 171, a novel irreversible inhibitor of the proteasome. Cancer Res. 2007; 67(13):6383-91.
6. Arastu-Kapur S, Shenk K, Parlati F and Bennett M. Non-Proteasomal Targets of Proteasome Inhibitors Bortezomib and Carfilzomib. Blood (ASH Annual Meeting Abstracts), Nov 2008; 112: 2657
7. Kirk CJ, Jiang J, Muchamuel T, et al. The Selective Proteasome Inhibitor Carfilzomib Is Well Tolerated in Experimental Animals with Dose Intensive Administration. Blood (ASH Annual Meeting Abstracts), Nov 2008; 112: 2765.
8. Kuhn DJ, Chen Q, Voorhees PM, et al. Potent activity of carfilzomib, a novel, irreversible inhibitor of the ubiquitin-proteasome pathway, against preclinical models of multiple myeloma. Blood. 2007 Nov 1;110(9):3281-90
9. Dimopoulos MA, Moreau P, Palumbo A, et al. ENDEAVOR Investigators. Carfilzomib and dexamethasone versus bortezomib and dexamethasone for patients with relapsed or refractory multiple myeloma (ENDEAVOR): a randomised, phase 3, open-label, multicentre study. Lancet Oncol. 2016 Jan;17(1):27-38.
10. Durie BG, Hoering A, Abidi MH, et al. Bortezomib with lenalidomide and dexamethasone versus lenalidomide and dexamethasone alone in patients with newly diagnosed myeloma without intent for immediate autologous stem-cell transplant (SWOG S0777): a randomised, open-label, phase 3 trial. Lancet. 2017 Feb 4;389(10068):519-527.
11. Attal M, Lauwers-Cances V, Hulin C, et al. Lenalidomide, bortezomib, and dexamethasone with transplantation for myeloma. N Engl J Med 376:1311-1320, 2017
12. Gay F, Cerrato C, Rota Scalabrini D, et al. Carfilzomib-Lenalidomide-Dexamethasone (KRd) Induction-Autologous Transplant (ASCT)-Krd Consolidation Vs KRd 12 Cycles Vs Carfilzomib-Cyclophosphamide-Dexamethasone (KCd) Induction-ASCT-KCd Consolidation: Analysis of the Randomized Forte Trial in Newly Diagnosed Multiple Myeloma (NDMM). Blood 2018 132:121
13. Spencer A, Lentzsch S, Weisel K, et al. Daratumumab plus bortezomib and dexamethasone versus bortezomib and dexamethasone in relapsed or refractory multiple myeloma: updated analysis of CASTOR. Haematologica. 2018 Sep 20.

14. Dimopoulos MA, San-Miguel J, Belch A, et al. Daratumumab plus lenalidomide and dexamethasone versus lenalidomide and dexamethasone in relapsed or refractory multiple myeloma: updated analysis of POLLUX. Haematologica. 2018 Sep 20.
15. Lonial S, Weiss BM, Usmani SZ, et al. Daratumumab monotherapy in patients with treatment-refractory multiple myeloma (SIRIUS): an open-label, randomised, phase 2 trial. Lancet. 2016 Apr 9;387(10027):1551-60.
16. Mateos MV, Dimopoulos MA, Cavo M, et al; ALCYONE Trial Investigators. Daratumumab plus Bortezomib, Melphalan, and Prednisone for Untreated Myeloma. N Engl J Med. 2018 Feb 8;378(6):518-528.
17. Jakubowiak AJ, Chari A, Lonial S, et al. Daratumumab (DARA) in combination with carfilzomib, lenalidomide, and dexamethasone (KRd) in patients (pts) with newly diagnosed multiple myeloma (MMY1001): An open-label, phase 1b study. Journal of Clinical Oncology 35, no. 15_suppl (May 20 2017)
18. Richardson Richardson P, et al: OP-106 Horizon—Melflufen therapy for RRMM patients refractory to daratumumab and/or pomalidomide. 2018 ASH Annual Meeting & Exposition. Abstract 600.
19. Ocio EM, Rodriguez Otero P, Bringhen S, et al. Preliminary Results from a Phase I Study of Isatuximab (ISA) in Combination with Bortezomib, Lenalidomide, Dexamethasone (VRd), and in Patients with Newly Diagnosed Multiple Myeloma (NDMM) Non-Eligible for Transplant. Blood 2018 132:595
20. Ocio EM, Mitsiades CS, Orlowski RZ and Anderson KC. Future agents and treatment direction in Multiple myeloma. Expert Rev Hematol. 2014;7(1):127-41
21. Richter JR, Martin TG, Vij R, Cole C, Atanackovic D, Zonder JA, et al. Updated data from a Phase II dose finding trial of single agent isatuximab (SAR650984, anti-CD38 mAb) in relapsed/refractory multiple myeloma (RRMM). J Clin Oncol. 2016;34(suppl;abstr 8005)
22. Thomas G. Martin III, Gabriel N Mannis, Ajai Chari, et al. Phase Ib Study of Isatuximab and Carfilzomib in Relapse and Refractory Multiple Myeloma. Blood 2016 128:2111.
23. Stewart AK, Rajkumar SV, Dimopoulos MA, et al; ASPIRE Investigators. Carfilzomib, lenalidomide, and dexamethasone for relapsed multiple myeloma. N Engl J Med. 2015 Jan 8;372(2):142-52.
24. Jakubowiak AJ, Dytfeld D, Griffith KA, et al. A phase 1/2 study of carfilzomib in combination with lenalidomide and low-dose dexamethasone as a frontline treatment for multiple myeloma. Blood. 2012 Aug 30;120(9):1801-9.
25. Berdeja JG, Rifkin RM, Lyons R, et al. Once-Weekly Carfilzomib with Dexamethasone Demonstrated Promising Safety and Efficacy in Patients with Relapsed or Refractory Multiple Myeloma Regardless of Age and Prior Bortezomib Exposure. Blood 2016 128:2129.
26. Moreau P, Mateos MV, Berenson JR, et al. Once weekly versus twice weekly carfilzomib dosing in patients with relapsed and refractory multiple myeloma (A.R.R.O.W.): interim analysis results

of a randomised, phase 3 study. Lancet Oncol. 2018 Jul;19(7):953-964. doi: 10.1016/S1470-2045(18)30354-1. Epub 2018 Jun 1. Erratum in: Lancet Oncol. 2018 Aug;19(8):e382.

27. A. Palumbo, S.V. Rajkumar, M.A. Dimopoulos, International Myeloma Working Group, et al. Prevention of thalidomide and lenalidomide associated thrombosis in myeloma Leukemia, 22 (2008), pp. 414-423
28. Ghosh N, Tucker N, Zahurak M, et al. Addition of clarithromycin to lenalidomide and dexamethasone (BiRd) is effective in MM after progression on lenalidomide and dexamethasone. Session 653. Myeloma: Therapy, excluding Transplantation. American Society of Hematology (ASH) 2013 meeting. Abstract 1960.
29. Rossi A, Tomer M, Jayabalan D, et. al. BiRd (clarithromycin, lenalidomide, dexamethasone): an update on long-term lenalidomide therapy in previously untreated patients with multiple myeloma. Blood. 2013 121:1982-1985.
30. Niesvizky R, Jayabalan DS, Christos P, et. al. BiRD (Biaxin [clarithromycin]/Revlimid [lenalidomide]/dexamethasone) combination therapy results in high complete- and overall-response rates in treatmentnaive symptomatic multiple myeloma. Blood. 2008;111: 1101-1109.
31. Cocks K, Cohen D, Wisløff F, et. al. An international field study of the reliability and validity of a disease-specific questionnaire module (the QLQ-MY20) in assessing the quality of life of patients with multiple myeloma. Eur. J. Cancer. 2007;43:1670-1678.
32. Herdman M, Gudex C, Lloyd A, et al. Development and preliminary testing of the new five-level version of the EQ-5D (EQ-5D-5L). Qual Lif Res. 2011;20:1727-1736
33. Ladetto M, Brüggemann M, Monitillo L, et. al. Next-generation sequencing and real-time quantitative PCR for minimal residual disease detection in B-cell disorders. Leukemia. 2014;28(6):1299-1307.
34. Martinez-Lopez J, Lahuerta JJ, Pepin F, et al. Prognostic value of deep sequencing method for minimal residual disease detection in multiple myeloma. Blood. 2014. DOI 10.1182/blood-2014-01-550020
35. Korde N, Roschewski M, Zingone A et al. Treatment With Carfilzomib-Lenalidomide-Dexamethasone With Lenalidomide Extension in Patients With Smoldering or Newly Diagnosed Multiple Myeloma. JAMA Oncol. 2015; 1(6):746-54.
36. Malvasi F, Corso Faini A. Mechanism of action of a new anti-CD38 antibody: enhancing myeloma immunotherapy. CCR; 25(10) May 15, 2019
37. Ocio M et al. Preliminary Results from a Phase I Study of Isatuximab (ISA) in Combination with Bortezomib, Lenalidomide, Dexamethasone (VRd) in Patients with Newly Diagnosed Multiple Myeloma (NDMM) Non-Eligible for Transplant" – Blood 2018 132:595
38. Richardson PG et al. A phase III randomized, open label, multicenter study comparing isatuximab, pomalidomide, and low-dose dexamethasone versus pomalidomide and low-dose dexamethasone in patients with relapsed/refractory multiple myeloma (RRMM). 2019 Asco Annual Meeting – Abstract n. 8004

39. Chari et al. Phase I-b study of isatuximab + carfilzomib in relapsed and refractory multiple myeloma (RRMM). Journal of Clinical Oncology 36, no. 15_suppl (May 20 2018)
40. Howard SC, Jones DP, Pui C-H. The tumour lysis syndrome. N Engl J Med. 2011;364(19):1844-54.
41. Biran N, Siegel D et al. Weekly carfilzomib, lenalidomide, and dexamethasone in relapsed or refractory multiple myeloma: A phase 1b study. Am J Hematol. 2019 Apr 25. doi: 10.1002/ajh.25498
42. Richez V, Gruchet C et al. Carfilzomib Weekly 20/56mg/m^2, Lenalidomide and Dexamethasone for Early Relapsed Refractory Multiple Myeloma. AJH 2019, DOI: 10.1002/ajh.25327
43. Moreau P, San Miguel J, Sonneveld P et al. Multiple myeloma: ESMO Clincial Practice Guidelines for diagnosis, treatment and follow-up. Annals of Oncology 28 (Supplement 4): iv52–iv61, 2017 doi:10.1093/annonc/mdx096
44. Grambsch P, Therneau T. Proportional hazards tests and diagnostics based on weighted residuals. Biometrika 1994; 81: 515–26.
45. Gay F, Cerrato C, Petrucci MT et al. Efficacy of carfilzomib lenalidomide dexamethasone (KRd) with or without transplantation in newly diagnosed myeloma according to risk status: Results from the FORTE trial. Journal of Clinical Oncology 37, no. 15_suppl (May 20, 2019) 8002-8002.
46. Weisel K, Asemissen AM, Schieferdecker A et al. Isatuximab, Carfilzomib, Lenalidomide and Dexamethasone (I-KRd) in front-line treatment of high-risk Multiple Myeloma: Results of the Safety Run-In cohort in the phase II, multicenter GMMG-CONCEPT trial. 17th International Myeloma Workshop, Abstract no. OAB-023
47. Jakubowiak A, Chari A et al. Daratumumab (DARA) in combination with carfilzomib, lenalidomide, and dexamethasone (KRd) in patients (pts) with newly diagnosed multiple myeloma (MMY1001): An open-label, phase 1b study. Journal of Clinical Oncology 35, no. 15_suppl (May 20, 2017) 8000-8000.
48. Costa L, Chhabra S et al. Daratumumab, Carfilzomib, Lenalidomide and Dexamethasone (Dara-KRd) Induction, Autologous Transplantation and Post-Transplant, Response-Adapted, Measurable Residual Disease (MRD)-Based Dara-Krd Consolidation in Patients with Newly Diagnosed Multiple Myeloma (NDMM). Abstract no 860, ASH 2019.
49. Landren O, Hultcrantz M et al. Weekly Carfilzomib, Lenalidomide, Dexamethasone and Daratumumab (wKRd-D) Combination Therapy Provides Unprecedented MRD Negativity Rates in Newly Diagnosed Multiple Myeloma: A Clinical and Correlative Phase 2 Study. Abstract no 862, ASH 2019.
50. Zamagni E, Nanni E, Gay F, et al. MRD evaluation by PET/CT according to Deauville criteria combined with bone marrow techniques in newly diagnosed transplant eligible multiple myeloma patients enrolled in the phase II FORTE trial. Abstract S207, EHA 2020.

21. Appendix

A. Criteria for MM and measurable disease

S.V. Rajkumar et al. (The Lancet Oncology, 2014: 15; e538-e548)

Criteria for MM

Clonal bone marrow plasma cells ≥10% or biopsy-proven bony or extramedullary plasmacytoma[1]
AND any one or more of the following myeloma defining events:

Evidence of end organ damage that can be attributed to the underlying plasma cell proliferative disorder, specifically:

- Hypercalcemia: serum calcium >0.25 mmol/L (>1 mg/dL) higher than ULN or >2.75 mmol/l (>11 mg/dL)
- Renal insufficiency: creatinine clearance[2] < 40mL/min or serum creatinine >177 µmol/L (>2 mg/dL)
- Anemia: hemoglobin >2 g/dL (1.2 mmol/L) below the lower limit of normal or hemoglobin <10 g/dL (6.2 mmol\L)
- Bone lesions: one or more osteolytic lesions on skeletal radiography, CT, or PET-CT[3]

or one or more of the following biomarkers of malignancy:

- Clonal bone marrow plasma cell percentage[1] ≥60%
- Involved: uninvolved serum free light chain ratio[4] ≥100
- >1 focal lesion[5] on MRI studies

Footnotes:

1. Clonality should be established by showing light-chain restriction on flow cytometry, immunohistochemistry, or immunofluorescence. Bone marrow plasma cell percentage should preferably be estimated from a core biopsy specimen; in case of a disparity between the aspirate and the core biopsy, the highest value should be used.
2. Measured or estimated by validated equations.
3. If bone marrow has less than 10% clonal plasma cells, more than one bone lesion is required to distinguish from solitary plasmacytoma with minimal marrow involvement.
4. These values are based on the serum Freelite assay (The Binding Site Group, Birmingham UK). The involved free light chain must be ≥100mg/L.
5. At least 2 focal lesions must be 5 mm or more in size.

Criteria for measurable disease

Serum M-protein > 10 g/l or

Urine M-protein > 200 mg/24 hours or

Abnormal FLC ratio with involved free light chain (FLC) > 100 mg/l or

Proven plasmacytoma by biopsy *

* If plasmacytoma is the only measurable parameter, the patient is not allowed to be included in the study, because of difficult response evaluation.

International Staging System for Multiple Myeloma (ISS stage)

International Staging System for Multiple Myeloma of the International Myeloma Working Group (J Clin Oncol 2005: 23; 3412-3420).

Stage	Criteria
I	Serum β2-microglobulin < 3.5 mg/L **and**
	Serum albumin ≥ 3.5 g/dL
II	Neither stage I nor stage III*
III	Serum β2-microglobulin ≥ 5.5 mg/L

* There are two categories for stage II: serum β2-microglobulin < 3.5 mg/L but serum albumin < 3.5 g/dL; or serum β2-microglobulin 3.5 to < 5.5 mg/L irrespective of the serum albumin level.

B. Response Criteria for Multiple Myeloma

(International Myeloma Working Group consensus criteria for response and minimal residual disease assessment in multiple myeloma. SV Rakjumar, B Paiva, KC Anderson et al. Lancet Oncol. 2016 Aug;17(8):e328-e346.)

RESPONSE CRITERIA

Response subcategory	*Response criteria*[a]
sCR*	CR as defined below plus<br>▪ Normal FLC ratio and<br>▪ Absence of clonal cells in bone marrow[b] by immunohistochemistry or immunophenotyping[c]
CR	▪ Negative immunofixation on the serum and urine and<br>▪ Disappearance of any soft tissue plasmacytomas and<br>▪ < 5% plasma cells in bone marrow[b]
VGPR	Serum and urine M-protein detectable by immunofixation but not on electrophoresis or 90% or greater reduction in serum M-protein plus urine M-protein level < 100 mg per 24 h<br>If the serum and urine M-protein are unmeasurable[d] a ≥ 90% decrease in the difference between involved and uninvolved FLC levels is required in place of the M-protein criteria
PR	≥ 50% reduction of serum M-protein and reduction in 24-h urinary M-protein by ≥ 90% or to < 200 mg per 24 h<br>If the serum and urine M-protein are unmeasurable[d] a ≥ 50% decrease in the difference between involved and uninvolved FLC levels is required in place of the M-protein criteria<br>If serum and urine M-protein are unmeasurable, and serum free light assay is also unmeasurable, ≥ 50% reduction in plasma cells is required in place of M-protein, provided baseline bone marrow plasma cell percentage was ≥ 30%<br>In addition to the above listed criteria, if present at baseline, a ≥ 50% reduction in the size of soft tissue plasmacytomas is also required
SD[d]	Not meeting criteria for CR, VGPR, PR or progressive disease

* will only be determined in case the FLC assay is available in the participating hospitals

Abbreviations: CR, complete response; FLC, free light chain; PR, partial response; SD, stable disease; sCR, stringent complete response; VGPR, very good partial response.

[a] All response categories require two consecutive assessments made at anytime before the institution of any new therapy; all categories also require no known evidence of progressive or new bone lesions if radiographic studies were performed.

[b] Confirmation with repeat bone marrow examination not needed.

[c] Presence/absence of clonal cells is based upon the κ/λ ratio. An abnormal k/l ratio by immunohistochemistry and/or immunofluorescence requires a minimum of 100 plasma cells for analysis. An abnormal ratio reflecting presence of an abnormal clone is κ/λ of > 4:1 or < 1:2.

[d] not recommended for use as an indicator of response; stability of disease is best described by providing the time to progression estimates

NOTE: Once (s)CR is established, response remains (s)CR until relapse is documented.

RELAPSE CRITERIA

Relapse subcategory	*Relapse criteria*
Progressive disease[a]<br><br>To be used for calculation of time to progression and progression-free survival end points for all patients including those in CR (includes primary progressive disease and disease progression on or off therapy)	Progressive disease: required one or more of the following:<br>Increase of ≥ 25% from nadir in<br>Serum M-component and/or (the absolute increase must be ≥ 0.5 g/dl)[e]<br>Urine M-component and/or (the absolute increase must be ≥ 200 mg/24 h<br>Only in patients without measurable serum and urine M-protein levels: the difference between involved and uninvolved FLC levels. The absolute increase must be >10 mg/dl.<br>Bone marrow plasma cell percentage: the absolute % must be ≥ 10%[f]<br>Definite development of new bone lesions or soft tissue plasmacytomas or definite increase in the size of existing bone lesions or soft tissue plasmacytomas.<br>Development of hypercalcemia (corrected serum calcium ≥ 11.5 mg/dl or2.65 mmol/l) that can be attributed solely to the plasma cell proliferative disorder
Clinical relapse[a]	Clinical relapse requires one or more of:<br><br>Direct indicators of increasing disease and/or end organ dysfunction (CRAB features)[b]. It is not used in calculation of time to progression or progression-free survival but is listed here as something that can be reported optionally or for use in clinical practice<br>1. Development of new soft tissue plasmacytomas or bone lesions<br>2. Definite increase in the size of existing plasmacytomas or bone lesions. A definite increase is defined as a 50% (and at least 1 cm) increase as measured serially by the sum of the products of the cross-diameters of the measurable lesion<br>3. Hypercalcaemia (> 2.65 mmol/l) [11.5 mg/dl]<br>4. Decrease in hemoglobin of ≥ 1.25 mmol/l [2 g/dl]<br>5. Rise in serum creatinine by 177 µmol/l or more [2 mg/dl or more]
Relapse from CR[a]<br><br>(To be used only if the end point studied is DFS)[d]	Any one or more of the following:<br>▪ Reappearance of serum or urine M-protein by immunofixation or electrophoresis<br>▪ Development of ≥ 5% plasma cells in the bone marrow[c]<br>▪ Appearance of any other sign of progression (i.e., new plasmacytoma, lytic bone lesion, or hypocalcaemia see above)

Abbreviations: CR, complete response; DFS, disease-free survival.

[a] All relapse categories require two consecutive assessments made at anytime before classification as relapse or disease progression and/or the initiation of any new therapy.

[b] For progressive disease, serum M-component increases of ≥ 10 g/l are sufficient to define relapse if M-component is ≥ 50 g/l.

[c] Relapse from CR has the 5% cutoff versus 10% for other categories of relapse.

[d] For purposes of calculating time to progression and progression-free survival, CR patients should also be evaluated using criteria listed above for progressive disease

PRACTICAL DETAILS OF RESPONSE EVALUATION

Laboratory tests for measurement of M-protein

- Serum M-protein level is quantitated using densitometry on SPEP except in cases where the SPEP is felt to be unreliable such as in patients with IgA monoclonal proteins migrating in the beta region. If SPEP is not available or felt to be unreliable (e.g., in some cases of IgA myeloma) for routine M-protein quantitation during therapy, then quantitative immunoglobulin levels on nephelometry or turbidometry can be accepted. However, this must be explicitly reported, and only nephelometry can be used for that patient to assess response and SPEP and nephelometric values cannot be used interchangeably.
- Urine M-protein measurement is estimated using 24-h UPEP only. Random or 24 h urine tests measuring kappa and lambda light chain levels are not reliable and are not recommended

Follow-up to meet criteria for PR or SD

- It is recommended that patients undergoing therapy will be tracked monthly for the first year of new therapy and every other month thereafter
- Except for assessment of CR, patients with measurable disease restricted to the SPEP will need to be followed only by SPEP; correspondingly, patients with measurable disease restricted to the UPEP will need to be followed only by UPEP[a]
- Patients with measurable disease in either SPEP or UPEP or both will be assessed for response only based on these two tests and not by the FLC assay. FLC response criteria are only applicable to patients without measurable disease in the serum or urine, and to fulfill the requirements of the category of stringent CR
- To be considered CR, both serum and urine immunofixation must be carried out and be negative regardless of the size of baseline M-protein in the serum or urine; patients with negative UPEP values pretreatment still require UPEP testing to confirm CR and exclude light chain or Bence–Jones escape
- Skeletal survey is not required for assessment of response unless clinically indicated, but is recommended once a year in clinical practice; bone marrow is required only for categorization of CR, and for patients with non-secretory disease

Abbreviations: CR, complete response; FLC, free light chain; PR, partial response; SD, stable disease; SPEP, serum protein electro-phoresis; UPEP, urine protein electrophoresis.
[a] For good clinical practice patients should be periodically screened for light chain escape with UPEP or serum FLC assay.

C. ZUBROD-ECOG-WHO Performance Status Scale

0 Normal activity
1 Symptoms, but nearly ambulatory
2 Some bed time, but to be in bed less than 50% of normal daytime
3 Needs to be in bed more than 50% of normal daytime
4 Unable to get out of bed
5 Death

D. Common Terminology Criteria for Adverse Events

The grading of adverse events will be done using the NCI Common Terminology Criteria for Adverse Events, CTCAE version 5.0. A complete document may be downloaded from the following sites:

https://ctep.cancer.gov/protocoldevelopment/electronic_applications/ctc.htm

http://www.eortc.be/Services/Doc/CTC

E. NYHA scoring list

The New York Heart Association functional and therapeutic classification applied to dyspnoea

Grade 1	No breathlessness
Grade 2	Breathlessness on severe exertion
Grade 3	Breathlessness on mild exertion
Grade 4	Breathlessness at rest

F. Modified Diet in Renal Disease Formula

For creatinine in mg/dL, the estimated glomerular filtration rate (e-GFR) for the modified diet in renal disease
(MDRD) formula is:

e-GFR (MDRD) mL/min per 1.73m^2 = 175 × [serum creatinine (mg/dL)]$^{-1.154}$ × [age]$^{-0.203}$ × [1.212 if black] × [0.742 if female]

For creatinine in µmol/L, the estimated glomerular filtration rate (e-GFR) for the modified diet in renal disease
(MDRD) formula is:

e-GFR (MDRD) mL/min per 1.73m^2 = 175 x [serum creatinine (µmol/L)/88.4]$^{-1.154}$ × [age]$^{-0.203}$ × [1.212 if black] × [0.742 if female]

Source: Levey 2006

G. Corrected calcium Formula

[Ca] + (0.02*(40-([Albumin(g/dL)]*10)))

H. Quality of Life Questionnaire EORTC QLQ-C30 (version 3)

We are interested in some things about you and your health. Please answer all of the questions yourself by circling the number that best applies to you. There are no "right" or "wrong" answers. The information that you provide will remain strictly confidential.

Please fill in your initials: __ __ __ __

Your birthdate (Day, Month, Year): __ __ __ __ __ __ __ __

Today's date (Day, Month, Year): __ __ __ __ __ __ __ __

	**Not at All**	**A Little**	**Quite a Bit**	**Very Much**
1. Do you have any trouble doing strenuous activities, like carrying a heavy shopping bag or a suitcase?	1	2	3	4
2. Do you have any trouble taking a long walk?	1	2	3	4
3. Do you have any trouble taking a short walk outside of the house?	1	2	3	4
4. Do you need to stay in bed or a chair during the day?	1	2	3	4
5. Do you need help with eating, dressing, washing yourself or using the toilet?	1	2	3	4

During the past week:

	**Not at All**	**A Little**	**Quite a Bit**	**Very Much**
6. Were you limited in doing either your work or other daily activities?	1	2	3	4
7. Were you limited in pursuing your hobbies or other	1	2	3	4

leisure time activities?				
8. Were you short of breath?	1	2	3	4
9. Have you had pain?	1	2	3	4
10. Did you need to rest?	1	2	3	4
11. Have you had trouble sleeping?	1	2	3	4
12. Have you felt weak?	1	2	3	4
13. Have you lacked appetite?	1	2	3	4
14. Have you felt nauseated?	1	2	3	4
15. Have you vomited?	1	2	3	4

During the past week:

	**Not at All**	**A Little**	**Quite a Bit**	**Very Much**
16. Have you been constipated?	1	2	3	4
17. Have you had diarrhea?	1	2	3	4
18. Were you tired?	1	2	3	4

19. Did pain interfere with your daily activities?	1	2	3	4
20. Have you had difficulty in concentrating on things, like reading a newspaper or watching television?	1	2	3	4
21. Did you feel tense?	1	2	3	4
22. Did you worry?	1	2	3	4
23. Did you feel irritable?	1	2	3	4
24. Did you feel depressed?	1	2	3	4
25. Have you had difficulty remembering things?	1	2	3	4
26. Has your physical condition or medical treatment interfered with your family life?	1	2	3	4
27. Has your physical condition or medical treatment interfered with your social activities?	1	2	3	4
28. Has your physical condition or medical treatment caused you financial difficulties?	1	2	3	4

For the following questions please circle the number between 1 and 7 that best applies to you

29. How would you rate your overall health during the past week?

1 2 3 4 5 6 7

Very poor Excellent

30. How would you rate your overall quality of life during the past week?

1 2 3 4 5 6 7

Very poor Excellent

© Copyright 1995 EORTC Study Group on Quality of Life. All rights reserved. Version 3.0

I. EORTC QLQ-MY20 Quality of Life Questionnaire EORTC QLQ-MY20

Patients sometimes report that they have the following symptoms. Please indicate the extent to which you have experienced these symptoms during the past weeks. Please answer by circling the answer that best applies to you.

During the past week:

	**Not at All**	**A Little**	**Quite a Bit**	**Very Much**
1. Have you had bone aches or pain?	1	2	3	4
2. Have you had bone pain in your back?	1	2	3	4
3. Have you had pain in your hip?	1	2	3	4
4. Have you had pain in your arm or shoulder?	1	2	3	4
5. Have you had pain in your chest?	1	2	3	4
6. If you had pain did it increase with activity?	1	2	3	4
7. Did you feel drowsy?	1	2	3	4
8. Did you feel thirsty?	1	2	3	4
9. Have you felt ill?	1	2	3	4
10. Have you had a dry mouth?	1	2	3	4

11. Have you lost any hair?	1	2	3	4
12. Answer this question only if you lost any hair: were you upset by the loss of your hair?	1	2	3	4
13. Did you have tingling hands or feet?	1	2	3	4
14. Did you feel restless or agitated?	1	2	3	4
15. Have you had acid indigestion or heartburn?	1	2	3	4
16. Have you had burning or sore eyes?	1	2	3	4
17. Have you felt physically less attractive as a result of your disease or treatment?	1	2	3	4
18. Have you been thinking about your illness?	1	2	3	4
19. Have you been worried about dying?	1	2	3	4
20. Have you worried about your health in the future?	1	2	3	4

© Copyright 1999 EORTC Study Group on Quality of Life. All rights reserved (phase III module)

J. Questionnaire EQ-5D-5L

Under each heading, please check the ONE box that best describes your health TODAY.

MOBILITY

I have no problems walking ☐
I have slight problems walking ☐
I have moderate problems walking ☐
I have severe problems walking ☐
I am unable to walk ☐

SELF-CARE

I have no problems washing or dressing myself ☐
I have slight problems washing or dressing myself ☐
I have moderate problems washing or dressing myself ☐
I have severe problems washing or dressing myself ☐
I am unable to wash or dress myself ☐

USUAL ACTIVITIES *(e.g. work, study, housework, family or leisure activities)*

I have no problems doing my usual activities ☐
I have slight problems doing my usual activities ☐
I have moderate problems doing my usual activities ☐
I have severe problems doing my usual activities ☐
I am unable to do my usual activities ☐

PAIN / DISCOMFORT

I have no pain or discomfort ☐
I have slight pain or discomfort ☐
I have moderate pain or discomfort ☐
I have severe pain or discomfort ☐
I have extreme pain or discomfort ☐

ANXIETY / DEPRESSION

I am not anxious or depressed ☐
I am slightly anxious or depressed ☐
I am moderately anxious or depressed ☐
I am severely anxious or depressed ☐
I am extremely anxious or depressed ☐

SPECIMEN

- We would like to know how good or bad your health is TODAY.
- This scale is numbered from 0 to 100.
- 100 means the <u>best</u> health you can imagine.
  0 means the <u>worst</u> health you can imagine.
- Mark an X on the scale to indicate how your health is TODAY.
- Now, please write the number you marked on the scale in the box below.

YOUR HEALTH TODAY = [ ]

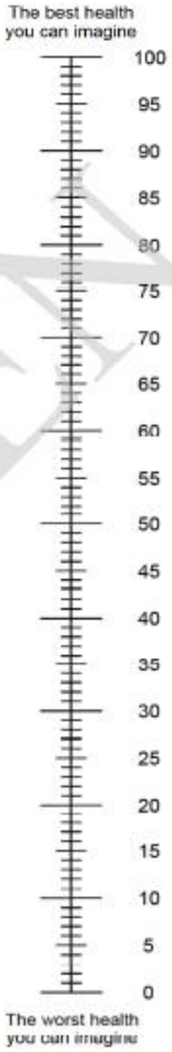

SPECIMEN

K. FACT/GOG ntx questionnaire (version 4.0)

Below is a list of statements that other people with your illness have said are important. **Please circle or mark one number per line to indicate your response as it applies to the past 7 days.**

	PHYSICAL WELL-BEING	Not at all	A little bit	Some -what	Quite a bit	Very much
GP1	I have a lack of energy	0	1	2	3	4
GP2	I have nausea	0	1	2	3	4
GP3	Because of my physical condition, I have trouble meeting the needs of my family	0	1	2	3	4
GP4	I have pain	0	1	2	3	4
GP5	I am bothered by side effects of treatment	0	1	2	3	4
GP6	I feel ill	0	1	2	3	4
GP7	I am forced to spend time in bed	0	1	2	3	4

	SOCIAL/FAMILY WELL-BEING	Not at all	A little bit	Some -what	Quite a bit	Very much
GS1	I feel close to my friends	0	1	2	3	4
GS2	I get emotional support from my family	0	1	2	3	4
GS3	I get support from my friends	0	1	2	3	4
GS4	My family has accepted my illness	0	1	2	3	4
GS5	I am satisfied with family communication about my illness	0	1	2	3	4
GS6	I feel close to my partner (or the person who is my main support)	0	1	2	3	4
Q1	*Regardless of your current level of sexual activity, please answer the following question. If you prefer not to answer it, please mark this box* ☐ *and go to the next section.*					
GS7	I am satisfied with my sex life	0	1	2	3	4

Please circle or mark one number per line to indicate your response as it applies to the past 7 days.

	**EMOTIONAL WELL-BEING**	**Not at all**	**A little bit**	**Some -what**	**Quite a bit**	**Very much**
GE1	I feel sad	0	1	2	3	4
GE2	I am satisfied with how I am coping with my illness	0	1	2	3	4
GE3	I am losing hope in the fight against my illness	0	1	2	3	4
GE4	I feel nervous	0	1	2	3	4
GE5	I worry about dying	0	1	2	3	4
GE6	I worry that my condition will get worse	0	1	2	3	4

	**FUNCTIONAL WELL-BEING**	**Not at all**	**A little bit**	**Some -what**	**Quite a bit**	**Very much**
GF1	I am able to work (include work at home)	0	1	2	3	4
GF2	My work (include work at home) is fulfilling	0	1	2	3	4
GF3	I am able to enjoy life	0	1	2	3	4
GF4	I have accepted my illness	0	1	2	3	4
GF5	I am sleeping well	0	1	2	3	4
GF6	I am enjoying the things I usually do for fun	0	1	2	3	4
GF7	I am content with the quality of my life right now	0	1	2	3	4

Please circle or mark one number per line to indicate your response as it applies to the past 7 days.

	**ADDITIONAL CONCERNS**	**Not at all**	**A little bit**	**Some -what**	**Quite a bit**	**Very much**
NTX 1	I have numbness or tingling in my hands	0	1	2	3	4
NTX 2	I have numbness or tingling in my feet	0	1	2	3	4
NTX 3	I feel discomfort in my hands	0	1	2	3	4
NTX 4	I feel discomfort in my feet	0	1	2	3	4

NTX 5	I have joint pain or muscle cramps……………………..	0	1	2	3	4
HI12	I feel weak all over…………………………………….	0	1	2	3	4
NTX 6	I have trouble hearing……………………………….	0	1	2	3	4
NTX 7	I get a ringing or buzzing in my ears…………………	0	1	2	3	4
NTX 8	I have trouble buttoning buttons………………………	0	1	2	3	4
NTX 9	I have trouble feeling the shape of small objects when they are in my hand……………………………………….	0	1	2	3	4
An6	I have trouble walking……………………………….	0	1	2	3	4

L. Correlative studies (central labs)

It is obligatory to send in bone marrow material (aspirate) and peripheral blood for the biobank for participation in this study. The biobank includes bone marrow cells, peripheral blood cells, and peripheral plasma amongst others. All material is stored according to biobank laws in the separate countries. Bone marrow samples and peripheral blood samples will be collected at entry. Bone marrow and peripheral blood are taken at all MRD timepoints, and in case of progression/relapse. For further details on logistics and laboratory procedures see lab manual.

The analyses will include:

i. Molecular and biological profiling

Whole genome transcriptional profiling will be used to establish the level of either all present and detectable RNA species by RNAseq (after depletion of ribosomal RNA, or selection of mRNA) or over 47,000 transcripts, representing 20,000 genes by Affymetrix U133 Plus 2.0 array. Moreover, genomic profiling by next generation sequencing (NGS) approach will be performed. Aim of this exploratory analysis is validate prognostic markers identified in previous studies and identification of novel candidate markers that predict patients response to the specific treatment used in the current study by correlations with clinical outcome (several papers Sonneveld lab; Broyl A Blood 2013, Kuiper R Leukemia 2012, Broyl A Blood 2010). Importantly, bone marrow samples depleted of myeloma cells will be used to characterize microenvironmental components, such as CD271 positive mesenchymal cells. Cell free DNA will be collected to assess the value of this parameter in the context of MM at diagnosis.

iii. CTC analysis.

At diagnosis, peripheral blood is collected and tested for the presence of circulating MM cells. Cryopreservation of peripheral blood allows for the molecular analysis of sorted CTCs. In addition, CTCs will be assessed at MRD timepoints.

iv. Future analyses to be determined

Other analyses may appear to be relevant at a later stage and the biobank is left open to interested groups related to EMN. The procedure and what analyses to be performed will be decided later. In addition to cryopreserved bone marrow cells and DNA of peripheral blood cells, peripheral blood plasma will be stored.

Ad i-iv) Including gene expression profiling and other molecular analyses

At least one day before the bone marrow aspiration will take place, it is required to notify the laboratory of the Erasmus Medical Center.

M. Mass spectrometry project

Immune enrichment-coupled mass spectrometry (MS) represents a novel sensitive approach for the accurate identification of serum monoclonal immunoglobulins in patients with monoclonal gammopathies (1). The technique shows potential as a replacement to electrophoretic approaches for monitoring response and may be complementary to bone marrow methods for minimal residual disease (MRD) assessment (2-5). The EMN will investigate the application of mass spectrometry for the detection of monoclonal proteins in serum samples from myeloma patients included in the EMN24 clinical trial of newly diagnosed multiple myeloma patients.

Objectives

1. To perform concordance analyses between MS, bone marrow MRD assessments (at least 10^{-5} sensitivity) and PET/CT for patients on study in a prospective fashion.
2. To compare the sensitivity of MS in patients achieving IMWG complete response or better (6).
3. Determine the ability of MS to discern between the M-protein and the therapeutic monoclonal antibody Isatuximab in patients treated in the Isatuximab-Carfilzomib-lenalidomide-dexamethasone treatment arm.

When possible, other research questions may include gaining an understanding on glycosylation patterns of the monoclonal protein.

Study Design

MS assessments of monoclonal immunoglobulins and/or FLCs (IgG, IgA, IgM, total kappa, total lambda, free kappa, free lambda) will be performed using matrix-assisted laser desorption/ionisation (MALDI) analysis on baseline and follow-up samples with MRD assessment provided by EMN.
Samples negative for MALDI mass spectrometry may be tested using liquid-chromatography mass spectrometry (LC-MS) (7).

MS will be performed on serum samples at enrollment and at specific time-points during treatment, including at the time of bone marrow MRD assessment, achievement of complete response and at relapse.

Additional immunoglobulin and FLC turbidimetric measurements may also be carried out on selected samples.

Materials

Analyses will be performed on serum samples from myeloma patients at baseline and at various time-points during treatment.

The samples will be stored at the time of collection at -80°C.

Methods

Serum immunoglobulins are immune-enriched using paramagnetic microparticles covalently coated with polyclonal sheep antibodies monospecific for human IgG, IgA, IgM, total κ and λ, and free κ and λ light chains (8, 9). Mass spectra is acquired separately for each isotype-specific immune-enriched sample. MALDI-TOF-MS for intact immunoglobulins and free light chains is performed using a Microflex-LT/SH-Smart (Bruker, GmbH) (8, 9). The same eluates are analyzed by MALDI-MS and LC-MS using a microLC-Q-TOF mass spectrometer (7). Mass spectra are inspected visually using data analysis software. An M-protein is defined at baseline by the presence of a sharp peak distinguishable from the polyclonal background in the light chain mass-to-charge (m/z) range, with a peak-height signal/noise value >3. Follow-up samples are investigated for the presence of an M-protein with the same m/z as determined at baseline.

References

1. Ashby J, North S, Barnidge D, Brusseau S, Patel R, Du Chateau B, Wallis G, Harding S, Sakrikar D. 2018. QIP-MS: A specific, sensitive, accurate, and quantitative alternative to electrophoresis for the identification of intact monoclonal immunoglobulins. Presented at AACC: A-321a

2. Puig N, Mateos MV, Contreras T, Paiva B, Cedena MT, Pérez JJ, Aires I, Agullo C, Martinez-Lopez J, Rodriguez Otero P, Gonzalez De La Calle V, Gonzalez MS, Oriol A, Gutierrez NC, Rios R, Rosiñol L, Alvarez MA, Calasanz MJ, Bargay J, Gonzalez AP, Alegre A, Escalante F, Martínez R, de la Rubia J, Teruel AI, De Arriba F, Palomera L, Hernández MT, López J, Martín J, García Mateo A, García-Sanz R, Ocio EM, Bladé J, Lahuerta JJ, San-Miguel JF. 2019. QIP-Mass Spectrometry in High Risk Smoldering Multiple Myeloma Patients Included in the GEM-CESAR Trial: Comparison with Conventional and Minimal Residual Disease IMWG Response Assessment. Blood 134: 581-

3. Puig N, Contreras T, Paiva B, Cedena MT, Perez JJ, Aires I, Agullo C, Martinez-Lopez J, Rodriguez-Otero P, Gonzalez-Calle V, Gonzalez MS, Oriol A, Gutierrez NC, Rios R, Rosinol L, Alvarez MA, Calasanz MJ, Bargay J, Gonzalez AP, Alegre A, Escalante F, Martinez R, De la Rubia J, Teruel AI, De Arriba F, Palomera L, Hernandez MT, Lopez J, Martin J, Garcia-Mateo A, Garcia-Sanz R, Ocio EM, Blade J, Lahuerta JJ, San Miguel JF, Mateos MV. 2020. QIP-Mass spectrometry including free light chains in high-risk myeloma patients enrolled in the GEM-CESAR trial: Comparison with conventional and MRD disease IMWG response assessment. Presented at EHA: EP946a

4. Spencer A, Khong T, Yuen F, Giles HV, Gorniak M, Quach H, Horvath N, Kerridge IH, Lee ES-H, Bergin K, Sridesai S, Kalff A, Reynolds J. 2019. A Longitudinal Evaluation of Euroflow and Combined

Quantitative Immunoprecipitation (QIP) and Free Light Chain (FLC) Mass Spectometry (MS) in Functional High Risk Multiple Myeloma. Blood 134: 3090-

5. Spencer A, Khong T, Yuen F, Giles HV, Gorniak M, Quach H, Horvath N, Kerridge IH, Sze-Hung Lee E, Bergin K, Sridesai S, Kalff A, Jenner E, Reynolds J. 2020. Mass spectrometry free light chain assessment for comprehensive monoclonal protein evaluation during myeloma monitoring. Presented at EHA: EP972a

6. Kumar S, Paiva B, Anderson KC, Durie B, Landgren O, Moreau P, Munshi N, Lonial S, Blade J, Mateos MV, Dimopoulos M, Kastritis E, Boccadoro M, Orlowski R, Goldschmidt H, Spencer A, Hou J, Chng WJ, Usmani SZ, Zamagni E, Shimizu K, Jagannath S, Johnsen HE, Terpos E, Reiman A, Kyle RA, Sonneveld P, Richardson PG, McCarthy P, Ludwig H, Chen W, Cavo M, Harousseau JL, Lentzsch S, Hillengass J, Palumbo A, Orfao A, Rajkumar SV, San Miguel J, Avet-Loiseau H. 2016. International Myeloma Working Group consensus criteria for response and minimal residual disease assessment in multiple myeloma. Lancet Oncol 17: e328-46

7. Derman BA, Stefka AT, McIver A, Jiang K, Kubicki T, Jasielec J, Jakubowiak A. 2020. Measurable Residual Disease Assessed by Mass Spectrometry in Peripheral Blood vs Next Generation Sequencing in Bone Marrow in Multiple Myeloma Treated on Phase II Trial of KRd+ASCT. J Clin Oncol 38: 8513a

8. North S, Barnidge D, Brusseau S, Patel R, Haselton M, Du Chateau B, Wallis G, Harding S, Sakrikar D, Ashby J. 2019. QIP-MS: A specific, sensitive, accurate, and quantitative alternative to electrophoresis that can identify endogenous m-proteins and distinguish them from therapeutic monoclonal antibodies in patients being treated for multiple myeloma. Clinica Chimica Acta 493: S433

9. Sharpley FA, Fontana M, Martinez-Naharro A, Manwani R, Mahmood S, Sachchithanantham S, Lachmann HJ, Gillmore JD, Whelan CJ, Hawkins PN, Wechalekar AD. 2019. Cardiac biomarkers are prognostic in systemic light chain amyloidosis with no cardiac involvement by standard criteria. Haematologica

N. Humoral and cellular immune response to SARS-CoV-2 vaccine/infection in MM patients treated within the phase III EMN24 trial

Background and study aim

Multiple myeloma (MM) patients are at risk of severe pneumonia after SARS-CoV-2 infection. Preliminary data suggest that MM patients have an impaired response to vaccination, and anti-CD38 immunotherapy can play a role in the immune responses to anti-SARS-CoV-2 vaccination and infection. The aim of the study is to evaluate the effect of SARS-CoV-2 vaccine/infection in MM patients treated within the phase III EMN24/IsKia trial in terms of induction of both humoral and cellular antigen (Ag)-specific immune responses.

Primary objective

The objective of this study is to assess the ability of SARS-CoV-2 vaccine/infection to elicit Ag-specific immune responses in MM patients treated with or without anti-CD38 immunotherapy.

Primary endpoint

Change of serum concentration of receptor binding domain (RBD)-specific IgG before treatment and after induction/ASCT/consolidation/light consolidation in patients treated with or without anti-CD38 immunotherapy.

Secondary endpoints

- Change of serum concentration of SARS-CoV-2 Nucleocapsid (N)-specific IgG before treatment and after induction/ASCT/consolidation/light consolidation in patients treated with or without anti-CD38 immunotherapy
- Change of the Spike (S)-specific T-cell response before treatment and after induction/ASCT/consolidation/light consolidation in patients treated with or without anti-CD38 immunotherapy
- Change of N-specific T-cell response before treatment and after induction/ASCT/consolidation/light consolidation in patients treated with or without anti-CD38 immunotherapy
- Correlation of anti-RBD serologic responses with T-cell-mediated responses
- Correlation of humoral and cellular Ag-specific immune responses with SARS-CoV-2 vaccine timing, vaccine type, SARS-CoV-2 infection occurrence, SARS-CoV-2 disease severity and MM-specific disease parameters (disease burden, disease response, polyclonal immunoglobulin levels)

Study design and methods

Samples collected from the patients enrolled in the III EMN24 trial will be used for this correlative study. Peripheral blood (PB) samples collected at screening and after induction, post-ASCT, post-ASCT

consolidation and after light consolidation in patients achieving at least a very good partial response (VGPR) will be evaluated. Only samples from patients at least in VGPR will be analyzed, since it is expected that the majority of the patients will achieve this type of response.

At specified time points, serum is isolated from PB samples by centrifugation and stored at -80 °C until use. PB mononuclear cells (PBMC) are isolated by density gradient centrifugation, cryopreserved in 90% fetal bovine serum and 10% dimethyl sulfoxide (DMSO) and stored in liquid nitrogen until use.

Humoral response to SARS-CoV-2 will be measured on serum samples by a Luminex 2 Plex IgG SARS-Cov-2 RBD, the target of the mRNA vaccines (semi-quantitative detection). In addition, IgG to SARS-CoV-2 N will be detected by a Luminex 2 Plex qualitative assay to determine a potential virus contact. The analyses will be performed by Labospace srl (Milan, Italy).

To evaluate T-cell mediated immune responses to SARS-CoV-2, thawed PBMC will be cultured in the presence or absence of a pool of peptides covering the immunodominant sequence domains of S protein of SARS-CoV-2 (15-mer sequences with 11 amino acids overlap, PepTivator® SARS-CoV-2 Prot_S, Miltenyi Biotec, Italy) in a 37 °C humidified incubator with 5% CO2 for 12 hours. In parallel, PBMC will be also exposed to a peptide pool covering the complete sequence of the N protein (15-mer sequences with 11 amino acids overlap, PepTivator® SARS-CoV-2 Prot_N, Miltenyi Biotec) to detect potential cellular responses to a viral infection. At the end of the culture, S- or N-specific T-cell response will be evaluated through the enumeration of cells secreting human IFN-γ in response to SARS-CoV-2 peptides using an Enzyme-Linked immunoSPOT (ELISPOT) assay (Human IFN-γ ELISpotPLUS kit (HRP), strips — Mabtech, #3420-4HST) following manufacturer instructions.

The correlation of humoral and–cellular Ag-specific immune responses with SARS-CoV-2 vaccine, COVID-19 disease and MM-specific disease parameters (treatment, response, polyclonal immunoglobulin levels) will be performed as well. All analyses will be done with Prism GraphPad or R.

Sample size

The expected number of serum samples to be analyzed, considering a VGPR rate of 80% after induction, 90% after ASCT, 90% after ASCT consolidation and 90% after light consolidation will be: 302 at baseline, 241 after induction, 272 after ASCT, 272 after ASCT consolidation and 272 after light consolidation (total 1359).

T-cell mediated immune response will be evaluated in a cohort of 50 patients, at 3 different timepoints (total 150). This cohort will be selected based on the availability of an adequate amount of biological sample at the Center, with an equal distribution of samples between the two study arms.

Redacted statistical analysis plan

STATISTICAL ANALYSIS PLAN

EMN24

Phase III study of Isatuximab-Carfilzomib-Lenalidomide-Dexamethasone (Isa-KRd) versus Carfilzomib-Lenalidomide-Dexamethasone (KRd) in newly diagnosed myeloma patients eligible for autologous stem cell transplantation (IsKia TRIAL)

**Study Acronym**	IsKia
**Study Code**	EMN24
**Sponsor**	EMN
**Co-Sponsor**	EMN Trial Office
**Version and Date:**	1.1, 27 May 2024
**Protocol Version and Date:**	2.0, 05 Jul 2021

TABLE OF CONTENTS

TABLE OF CONTENTS 2

1 DOCUMENT INFORMATION 4

2 INTRODUCTION 7

2.1 BACKGROUND AND RATIONALE 7
2.2 RESEARCH HYPOTHESIS, PRIMARY AND KEY SECONDARY OBJECTIVES 7
2.3 OBJECTIVES 7

3 STUDY METHODS 9

3.1 TRIAL DESIGN 9
3.2 TREATMENT DETAILS 9
3.3 RANDOMIZATION 11
3.4 SAMPLE SIZE 12
3.5 FRAMEWORK 13
3.6 INTERIM ANALYSES AND STOPPING GUIDANCE 13

4 STATISTICAL PRINCIPLES 14

4.1 CONFIDENCE INTERVALS AND P VALUES 14
4.2 ADHERENCE AND PROTOCOL DEVIATIONS 14
4.2.1 Multiplicity 14
4.2.2 Protocol deviations 14
4.3 ANALYSIS POPULATIONS 14
4.3.1 Intention-to-treat population 14
4.3.2 Safety population 14
4.3.3 Induction population 15
4.3.4 Mobilization population 15
4.3.5 Post ASCT consolidation population 15
4.3.6 Light consolidation population 15
4.3.7 Maintenance population 15
4.4 GENERAL PRINCIPLES 15

5 TRIAL POPULATION 16

5.1 SCREENING DATA 16
5.2 ELIGIBILITY 16
5.2.1 Inclusion Criteria 16
5.2.2 Exclusion Criteria 16
5.3 RECRUITMENT 16
5.4 WITHDRAWAL/FOLLOW-UP 18
5.5 BASELINE PATIENT CHARACTERISTICS 18

6 ANALYSIS 20

6.1 OUTCOME DEFINITIONS 20
6.1.1 Primary Endpoint (NGS MRD-negativity after the end of ASCT consolidation) 20
6.1.2 First key secondary endpoint (NGS MRD-negativity within the end of induction) 21
6.1.3 Second key secondary endpoint (Progression free survival) 22
6.1.4 Secondary Endpoints 23
6.2 ANALYSIS METHODS 40
6.2.1 Supplementary analyses 40
6.3 STATISTICAL SOFTWARE 40

A. TREATMENT SCHEMA 41

B. CONSORT DIAGRAM 42
C. REFERENCES 42

1 Document Information

DOCUMENT APPROVALS				
	**Name and surname**	**Job Title Affiliation**	**Signature**	**Date (*dd/mm/yyyy*)**
**Author**	Andrea Capra	Statistics Area Operator	[redacted]	
**Reviser**	Stefano Spada	Statistics Area Responsible		
**Approver**	Mario Boccadoro	EMN Representative		

VERSION HISTORY				
**Version N.**	**In force from**	**In force to**	**Replaced by**	**Summary of changes**
1.0	26/05/2023	27/05/2024	1.1	
1.1	27/05/2024			- Added new efficacy endpoints: IMWG best response within each treatment phase (post induction, post ASCT, post ASCT consolidation and post light consolidation)<br>- Section 6.1.4.3.3 Death rewritten

LIST OF ABBREVIATION	
AE	Adverse Event
ALT	Alanine Aminotransferase
ANC	Absolute Neutrophil Count
ASCT	Autologous Stem Cell Transplantation
AST	Aspartate Aminotransferase
Bj	Bence Jones
CI	Confidence Interval
CTCAE	Common Terminology Criteria for Adverse Events
ECOG	Eastern Cooperative Oncology Group
eCRF	electronic Case Report Form
FISH	Fluorescence In Situ Hybridization
FLC	Free Light Chain
IDMC	Independent Data Monitoring Committee
IQR	Inter-Quartile Range
ISS	International Staging System score
ITT	Intention-To-Treat
LDH	Lactate dehydrogenase
LTFU	Long-term Follow-up
MM	Multiple Myeloma
MRD	Minimal Residual Disease
MS	Mass Spectrometry
NGS	Next Generation Sequencing
NS	Non Secretory
PD	Progressive Disease
PT	Preferred Term
PFS	Progression Free Survival
RISS	Revised International Staging System score
SAE	Serious Adverse Event
SAP	Statistical Analysis Plan
SOC	System Organ Class
ULN	Upper Limit Normal

2 Introduction

2.1 Background and rationale

This Statistical Analysis Plan (SAP) provides details for the analysis of the trial EMN24 and it was produced according to Guidelines for the Content of Statistical Analysis Plans in Clinical Trials (JAMA. 2017;318(23):2337-2343. doi:10.1001/jama.2017.18556).

2.2 Research hypothesis, primary and key secondary objectives

This protocol is a phase III study designed to compare the efficacy and the safety of Isa-KRd induction, ASCT, Isa-KRd post ASCT consolidation and Isa-KRd light consolidation vs KRd induction, ASCT, KRd post ASCT consolidation and KRd light consolidation.
In particular, it will be tested: the null hypothesis that there is no difference in the rate of MRD negativity ($\geq 10^{-5}$ sensitivity level) after ASCT consolidation between two arms (primary objective), the null hypothesis that there is no difference in the rate of MRD negativity ($\geq 10^{-5}$ sensitivity level) after induction between two arms (first key secondary objective), the null hypothesis that there is no difference in the progression free survival between two arms (second key secondary objective).
Regarding interim analysis, the hypothesis is focus on the second key secondary endpoint.

More information is provided in the protocol.

2.3 Objectives

The primary objective of the study is:

- To compare rate of Minimal Residual Disease (MRD) negativity by NGS between Isa-KRd and KRd in post ASCT consolidation treatment

Key secondary objectives are:

- Rate of MRD negativity after induction by NGS
- To compare progression-free survival (PFS) in the 2 treatment arms.

Other secondary objectives are:

- Rate of MRD negativity after light consolidation by NGS
- Determine the overall response rate (ORR), VGPR, CR, sCR rate after induction, ASCT, post ASCT consolidation, light consolidation in the 2 treatment arms
- Determine the rate of MRD negativity (by NGS) after ASCT
- Determine the rate of MRD negativity (by NGF) after induction, ASCT, post ASCT consolidation and light consolidation.
- Determine the duration of response (DOR) in the 2 treatment arms
- Determine the duration of MRD negativity (by NGS and NGF)
- Determine the rate of sustained for 1-year MRD negativity (by NGF and NGS) (from post ASCT consolidation to post light consolidation)
- Determine the time to progression (TTP) in the 2 treatment arms
- Determine the overall survival (OS) in the 2 treatment arms
- Determine the time to next therapy (TNT) in the 2 treatment arms
- Determine the PFS2 in the 2 treatment arms

- Determine whether tumor response and outcome may change in subgroups with different prognosis according to current prognostic factors
- Determine safety in the 2 treatment arms
- Determine the success of stem cell harvest
- Determine the success of engraftment after ASCT
- Determine Quality of life in the two treatment arms

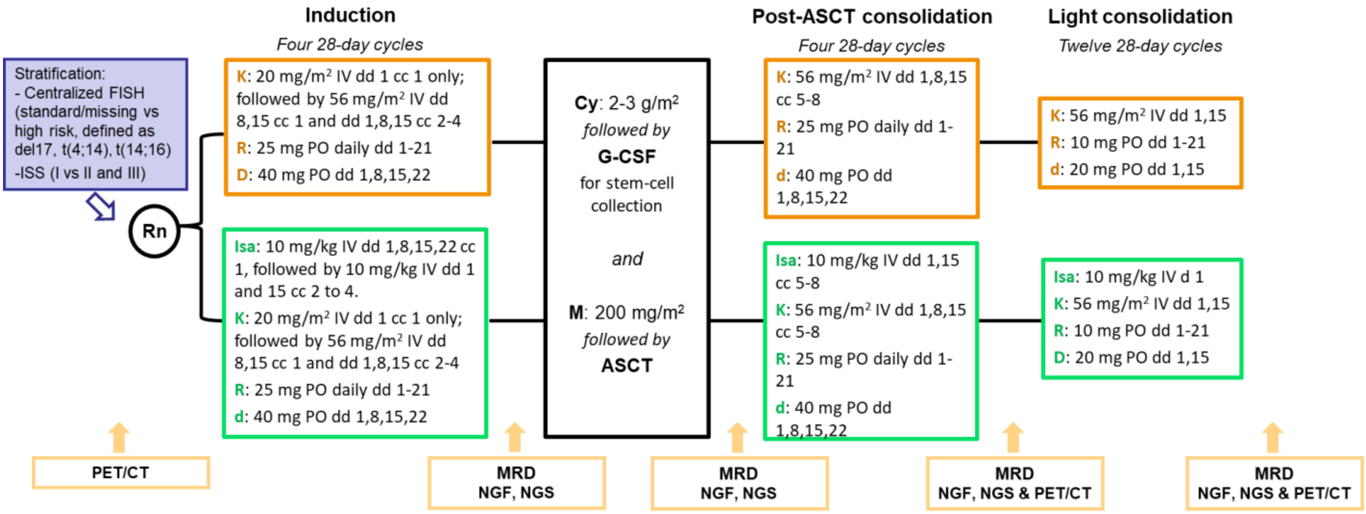

3 Study Methods

3.1 Trial design

This is an open-label randomized phase III study that enrolls newly diagnosed MM patient eligible for high-dose chemotherapy and ASCT. Patients will be randomized at enrolment into 2 treatment arms:

- Isa-KRd: induction with 4 cycles of Isatuximab-Carfilzomib-Lenalidomide-dexamethasone (Isa-KRd) followed by cyclophophamide and stem cell collection, chemotherapy with Melphalan 200 mg/m2 followed by ASCT (Mel200-ASCT), 4 cycles of Isa-KRd post ASCT consolidation and 12 cycles of Isatuximab-Lenalidomide-Carfilzomib-dexamethasone (Isa-KRd) light consolidation;
- KRd: induction with 4 cycles of Carfilzomib-Lenalidomide-dexamethasone (KRd) followed by cyclophophamide and stem cell collection, chemotherapy with Melphalan 200 mg/m2 followed by ASCT (Mel200-ASCT), 4 cycles of KRd post ASCT consolidation and 12 cycles of Carfilzomib-Lenalidomide-dexamethasone (KRd) light consolidation.

After light consolidation patients are allowed to receive Lenalidomide maintenance as per standard of care.

This study consists of 4 phases for each study subject: screening, treatment, observation period and long term follow up (LTFU).
The pre-treatment period includes screening visits, performed at study entry. After providing written informed consent to participate in the study, patients will be evaluated for study eligibility. The screening period includes the availability of inclusion criteria described in the study protocol.
The treatment period for the control arm includes administration of four 28-day cycles of induction with KRd followed by ASCT, administration of four 28-day cycles of post ASCT consolidation with KRd and administration of twelve 28-day cycles of light consolidation with KRd.
The treatment period for the experimental arm includes administration of four 28-day cycles of induction with Isa-KRd followed by ASCT, administration of four 28-day cycles of post ASCT consolidation with Isa-KRd and administration of twelve 28-day cycles of light consolidation with Isa-KRd.
Response assessment will be performed after each cycle.
The observation period will start after the end of light consolidation until progression of disease. Data on response assessment and treatment administered should be provided every 2 months.
The LTFU period will start after development of confirmed progressive disease (PD), all patients are to be followed for subsequent treatment and survival status during the LTFU period every 3 months via telephone or office visit.
Study Design is reported in Appendix A: Treatment Schema.

3.2 Treatment details

Arm A: Isa- KRd arm
Induction schedule
Repeat for 4 28-day cycles of induction
Table 1: Induction phase dose and schedule in Arm A

Agent	Dose/day	Route of administration	Days
Isatuximab	10 mg/kg	IV	1, 8, 15, and 22 Cycle 1; days 1 and 15 Cycles 2 to 4.

Carfilzomib	20 mg/m²	IV	1 cycle 1
Carfilzomib	56 mg/m²	IV	8, 15 cycle 1 and on days 1, 8, 15 for cycles 2-4
Lenalidomide	25 mg	OS	1-21
Dexamethasone	40 mg	OS/IV	1, 8, 15, 22

Transplant (as per Standard of Care)
All patients will be given Cyclophosphamide at the dose of 2 to 3 g/m², followed by G-CSF for stem cell collection, or other treatment according to local protocols. Cyclophosphamide will start 4-6 weeks after day 21 of cycle 4.

Stem cell collection will be performed as soon as CD34+ cells are present in peripheral blood. Stem cells will be harvested at a minimum of 4 x 10^6 CD34+ cells/kg and cryopreserved. A second course of mobilization will be performed as per local clinical practice. Plerixafor is allowed according to local standard of care.
Subjects will receive melphalan 200 mg/m² as conditioning therapy, according to the standard of care. Melphalan may be given at a lower dose of 140mg/m², per institutional standards, if the subject has renal insufficiency

Consolidation schedule
Repeat for 4 28-day cycles of consolidation.
Consolidation will start 6-8 weeks after autologous transplant.
Table 2: Consolidation phase dose and schedule in the Isa KRd arm

**Agent**	**Dose/day**	**Route of administration**	**Days**
Isatuximab	10 mg/kg	IV	1, 15
Carfilzomib	56 mg/m²	IV	1, 8, 15
Lenalidomide	25 mg	OS	1-21
Dexamethasone	40 mg	OS/IV	1, 8, 15, 22

Light consolidation schedule
Repeat for 12 28-day cycles.
Table 3: Light consolidation phase dose and schedule in the Isa KRd arm

**Agent**	**Dose/day**	**Route of administration**	**Days**
Isatuximab	10 mg/kg	IV	1
Carfilzomib	56 mg/m²	IV	1, 15
Lenalidomide	10 mg	OS	1-21
Dexamethasone	20 mg	OS/IV	1, 15

Maintenance
After light consolidation physicians are advised to continue with lenalidomide maintenance as per standard of care, since the current standard of care after ASCT with or without consolidation is lenalidomide maintenance.

Arm B: KRd arm
Induction schedule
Repeat for 4 28-day cycles of induction
Table 4: Induction phase dose and schedule in the KRd arm

Agent	Dose/day	Route of administration	Days
Carfilzomib	20 mg/m^2	IV	1 cycle 1
Carfilzomib	56 mg/m^2	IV	8, 15 cycle 1 and on days 1, 8, 15 for cycles 2-4
Lenalidomide	25 mg	OS	1-21
Dexamethasone	40 mg	OS/IV	1, 8, 15, 22

Transplant (as per Standard of Care)
All patients will be given Cyclophosphamide at the dose of 2 to 3 g/m^2, followed by G-CSF for stem cell collection, or other treatment according to local protocols. Cyclophosphamide will start 4-6 weeks after day 21 of cycle 4.

Stem cell collection will be performed as soon as CD34+ cells are present in peripheral blood. Stem cells will be harvested at a minimum of 4 x 10^6 CD34+ cells/kg and cryopreserved. A second course of mobilization will be performed as per local clinical practice. Plerixafor is allowed according to local standard of care.
Subjects will receive melphalan 200 mg/m^2 as conditioning therapy, according to the standard of care. Melphalan may be given at a lower dose of 140mg/m^2, per institutional standards, if the subject has renal insufficiency

Consolidation schedule
Repeat for 4 28-day cycles of consolidation.
Consolidation will start 6-8 weeks after autologous transplant.
Table 5: Consolidation phase dose and schedule in the KRd arm

Agent	Dose/day	Route of administration	Days
Carfilzomib	56 mg/m^2	IV	1, 8, 15
Lenalidomide	25 mg	OS	1-21
Dexamethasone	40 mg	OS/IV	1, 8, 15, 22

Light consolidation schedule
Repeat for 12 28-day cycles.
Table 6: Light consolidation phase dose and schedule in the Isa KRd arm

Agent	Dose/day	Route of administration	Days
Carfilzomib	56 mg/m^2	IV	1, 15
Lenalidomide	10 mg	OS	1-21
Dexamethasone	20 mg	OS/IV	1, 15

Maintenance
After light consolidation physicians are advised to continue with lenalidomide maintenance as per standard of care, since the current standard of care after ASCT with or without consolidation is lenalidomide maintenance.

3.3 Randomization

All patients eligible will be randomized at enrolment in a 1:1 ratio into the 2 treatment arms.

Patients will be stratified according to ISS International Staging System [3 levels: I vs II vs III] and cytogenetic risk FISH [2 levels: high-risk vs standard risk/missing], high risk is defined as having Deletion 17p13.1, [≥10%] or Translocation 4;14 (p16.3;q32.3), [≥15%] or Translocation 14;16 (q32.3;q23) [≥15%]) and then randomized by eCRF; this procedure is completely concealed to study participants.

Randomizations will be performed based on a randomization list provided by statisticians; it was created using R software (R Foundation for Statistical Computing, Vienna, Austria - Version 3.6.0) by the function blockrand of the blockrand package. The list was created using dynamic sizes of blocks, from 2 to 6, for each stratum.

3.4 Sample size

The calculation of the sample size for the primary endpoint was done with the following assumptions, consider ITT population:

- α= 0.05 (two sided)
- β= 0.10
- Allocation ratio: 1:1
- post ASCT consolidation MRD negativity (10^{-5}) (NGS) rate Isa-KRd arm: 64%
- post ASCT consolidation MRD negativity (10^{-5}) (NGS) rate KRd arm: 45%

The total number of patients required is 300 (by the X2 test with Yates' continuity correction).

A hierarchical testing procedure (using the Fixed-Sequence Method) will be used for the key secondary endpoints to achieve control of the overall familywise Type I error rate at a two-sided significance level of 0.05. The details of the testing procedure will be prespecified in multiplicity section.

The power of 85% (β= 0.15) for the first key secondary endpoint (MRD negativity rate after induction by NGS) was done with the following assumptions by the X2 test with Yates' continuity correction, consider ITT population:

- α= 0.05 (two sided)
- Allocation ratio: 1:1
- post induction MRD negativity (10-5) (NGS) rate Isa-KRd arm: 30%
- post induction MRD negativity (10-5) (NGS) rate KRd arm: 15%

The power of 92% (β= 0.08) for the second key secondary endpoint (PFS) was done with the following assumptions by Schoenfeld formula, consider ITT population:

- α= 0.05 (two sided)
- Allocation ratio: 1:1
- 60 months PFS Isa-KRd arm: 80%
- 60 months PFS KRd arm: 60% (HR:0.44)
- Accrual time: 10 months
- Follow-up time: 42 months
- Lost to follow-up: 5%

To achieve 92% power, 68 PFS events are needed, expected after 42 months of last randomized patient.

Long-term follow-up for survival will continue until approximately 170 deaths have been observed or 9 years have elapsed after the last subject is randomized. This will provide approximately 71% power to detect a reduction of the risk of death (HR = 0.68) with a 2-sided alpha of 0.05 by Schoenfeld formula.

3.5 Framework

The EMN24 trial testing the superiority of Isa-KRd vs KRd in terms of MRD rate post ASCT consolidation, MRD rate post induction and PFS.

3.6 Interim analyses and stopping guidance

The first interim analysis is planned, primarily to describe Adverse Events observed during induction. This analysis will be conducted when complete data of the first 75 patients regarding the 4 cycles of induction therapy are available.

Data on stem cell collection, after mobilization, will be also analyzed after the first 75 patients completed that phase (second interim analysis).

A third interim analysis is planned, primarily to describe Adverse Events observed during post ASCT consolidation. This analysis will be conducted when complete data of the first 75 patients regarding the 4 cycles of post ASCT consolidation therapy are available.

A fourth interim analysis is planned, primarily to describe Adverse Events observed during light-consolidation. This analysis will be conducted when complete data of the first 75 patients regarding the 4 cycles of light-consolidation therapy are available.

Since safety is not the principal aim of the study, for interim analysis 1-4 no statistical correction of the sample size or of the alpha error have been done.

A fifth interim analysis is planned for PFS.

Results of interim analyses will be presented to the principal investigators, steering committee and to an independent data monitoring committee that can decide to continue or stop the trial.

The statistical analysis plan for the interim analyzes has already been presented and reviewed by IDMC. The four interim analyzes for safety have been done.

4 Statistical Principles

4.1 Confidence intervals and P values

All applicable statistical tests will be 2-sided and will be performed using a 5% significance level. All confidence intervals (CIs) presented will be 95% and two-sided. P-values will be rounded to 3 decimal places. P-values that round to 0.000 will be presented as '<0.001'.

4.2 Adherence and protocol deviations

4.2.1 Multiplicity

A hierarchical testing procedure will be used for primary (H1 post ASCT consolidation) and key secondary endpoints (H2 as MRD post induction and H3 as PFS) to achieve control of the overall familywise Type I error rate at a two-sided significance level of 0.05.
Regarding H3, an interim analysis (the fifth interim) will be performed when 40% of PFS event required for the final analysis.
The critical P-values at the interim analyses will be 0.0008, while the critical P-value for the final analysis will be 0.0492 using O'Brien and Fleming alpha spending function. (DeMets & Lan, 1994)

Critical p-value was computed using the "gsDesign" package of R language and environment for statistical computing (R Foundation for Statistical Computing, Vienna, Austria - Version 3.6.1).

4.2.2 Protocol deviations

Protocol deviations are explained and summarized in the Protocol Deviation Handling Document of the trial and stored in a dedicated section of eCRF. The number (and percentage) of patients with major and minor protocol deviations will be summarized by treatment arm with details of type of deviation provided. The patients that are included in the ITT analysis data set will be used as the denominator to calculate the percentages. No formal statistical testing will be undertaken.

4.3 Analysis populations

4.3.1 Intention-to-treat population

The Intention-to-treat population (ITT) includes all subjects who are eligible and randomized to the study. However, in case of patients initially randomized but considered ineligible afterwards based on information that should have been available before randomization, will be excluded from the respective analyses.

4.3.2 Safety population

The Safety population includes subjects of ITT population who received at least one dose of study drugs at induction.

4.3.3 Induction population

Induction safety population considers patients of ITT population who received at least one dose of study drugs at induction.

4.3.4 Mobilization population

Mobilization population considers patients of ITT population who received at least one dose of study drugs at induction and proceed to mobilization phases (= got at least G-CSF at mobilization dose).

4.3.5 Post ASCT consolidation population

Post ASCT consolidation population considers patients of ITT population who received at least one dose of study drugs at post ASCT consolidation phase.

4.3.6 Light consolidation population

Light consolidation population considers patients of ITT population who received at least one dose of study drugs at light consolidation phase.

4.3.7 Maintenance population

Maintenance population considers patients of ITT population who complete light consolidation phase and start maintenance as per standard of care, or proceeded to follow-up without maintenance treatment

4.4 General principles

Discrete variables will be tabulated as numbers and percentages, continuous variables will be summarized using median and inter-quartile range (IQR).
An MRD assessment (by NGS and NGF) will be considered valid if it reaches at least $\leq 10^{-5}$ sensitivity level.
All analyses related to MRD by NGS will be replicated using MRD by NGF.

5 Trial Population

5.1 Screening data

Patients screened are collected on eCRF. Patients screened and not randomized will be reported on CONSORT diagram; the reason for non-recruitment will be tabulated.

5.2 Eligibility

5.2.1 Inclusion Criteria

See section 7.1 of the study protocol.

5.2.2 Exclusion Criteria

See section 7.1 of the study protocol.

5.3 Recruitment

A CONSORT flow diagram (Appendix B: Consort Diagram) will be adopted for the final analysis to summarize the number of patients included in any phase of the study. The CONSORT will summarize:

- Screened patients
- Randomized patients
- Patients who started induction phase and number and causes of discontinuation
- Patients who started mobilization phase and number and causes of discontinuation
- Patients who started ASCT and number and causes of discontinuation
- Patients who started post ASCT consolidation phase and number and causes of discontinuation
- Patients who started light consolidation phase and number and causes of discontinuation
- Patients who started standard of care maintenance and number and causes of discontinuation and patients who proceeded with follow-up without starting maintenance
- Patients who are on treatment at data of analysis
- Patients who are on maintenance or in follow-up without maintenance at data of analysis

Patients who went off protocol at any phase will be listed and grouped by exclusion reason. Median follow-up will be also reported.

In addition, the following data will be reported:

I. Patients that didn't start treatment and cause of study discontinuation (Progression of disease vs Death vs Adverse Event vs Lost to follow-up vs Protocol violation vs Mobilization failure vs Withdrawal of consent vs Suspected pregnancy vs Medical Decision vs Other);
II. Patients that start treatment and discontinued with the cycle and the reason (Progression of disease vs Death vs Adverse Event vs Lost to follow-up vs Protocol violation vs Mobilization failure vs Withdrawal of consent vs Suspected pregnancy vs Medical Decision vs Other)
III. For patients that discontinued due to Other, Medical decision, Adverse Events and Death additional details will be provided.

Descriptive statistics will be presented by arm and overall.

Moreover, duration of treatment defined as time from the date of start therapy to date of discontinuation therapy/end of light consolidation will be calculated. Median duration of treatment and still-on-treatment rate estimates at relevant timepoints along with the 95% CI, will be derived using the Kaplan-Meier method. Actuarial estimates of the reasons for discontinuation of treatment (i.e., “Progression of disease” and “Other”) along with the 95% confidence interval (CI) at relevant timepoints will be calculated using the compering risk method.

5.4 Withdrawal/follow-up

The reason for withdrawal from treatment will be documented in the eCRF. Post-treatment follow-up for disease status and survival will continue until death unless any of the criteria for early study withdrawal are met. Patients who drop-out for reasons other than PD except consent withdrawal are followed until PD for response assessment.

5.5 Baseline patient characteristics

Each population will be described for its baseline characteristics as follow:

<u>Demographics:</u>
- Age(years) as continuous
- Age [≤ 60 vs > 60]
- Sex [Male vs Female]

<u>Disease characteristics:</u>
- Isotype [IgA vs IgG vs IgD vs IgE vs IgM vs BJ]
- Light Chain [Kappa vs Lambda]
- Extramedullary disease [Yes vs No]

<u>Prognostic factors</u>
- International Staging System score (ISS) [I vs II vs III]
- Revised International Staging System score (RISS) [I vs II vs III vs NE]
- Eastern Cooperative Oncology Group performance status score (ECOG) [0 vs 1 vs 2]
- Serum LDH [≤ upper normal limit vs > upper normal limit]
- Albumin [g/dL]
- B2-microglobulin [mg/L]

<u>Laboratory parameters</u>
- Haemoglobin [g/dL]
- White Blood Cells [10^9L]
- Absolute Neutrophils Count [10^9L]
- Platelets [10^9L]
- Creatinine [mg/dL]
- Creatinine clearance [≤ 45 vs > 45 ml/min]
- AST [≤ upper normal limit vs > upper normal limit]
- ALT [≤ upper normal limit vs > upper normal limit]
- Total Bilirubin [≤ upper normal limit vs > upper normal limit]
- Corrected Calcium [mmol/L]
- C reactive protein [mg/L]

<u>Cytogenetics</u>
- Deletion 17p13.1 [Yes vs No vs NE]
- Translocation 4;14 (p16.3;q32.3) [Yes vs No vs NE]
- Translocation 14;16 (q32.3;q23) [Yes vs No vs NE]
- Gain 1q21 (3 copies of 1q21) [Yes vs No vs NE]
- Amplification 1q21 (>3 copies of 1q21) [Yes vs No vs NE]

- presence of at least one of Deletion 17p13.1, Translocation 4;14 (p16.3;q32.3) or Translocation 14;16 (q32.3;q23) (cytogenetic risk FISH) [Standard vs High vs NE]
- presence of at least one of Deletion 17p13.1, Translocation 4;14 (p16.3;q32.3), Translocation 14;16 (q32.3;q23) or Gain 1q21/Amplification 1q21 (Gain 1q21 + Amplification 1q21) [Standard vs High vs NE]

The results of all characteristics will be summarized both overall and separately for the two arms.
Categorical data will be summarised by numbers and percentages. Continuous data will be summarised by median and interquartile range (IQR).
Tests of statistical significance will not be undertaken for baseline characteristics; rather the clinical importance of any imbalance will be noted.

6 Analysis

6.1 Outcome definitions

6.1.1 Primary Endpoint (NGS MRD-negativity after the end of ASCT consolidation)

Definition
The primary endpoint is the achievement of the NGS MRD-negativity at ≤10^{-5} sensitivity level (NGS_conASCT).
At patient' level, NGS_conASCT is defined as subject who obtains an NGS MRD negative sample within the end of ASCT consolidation.
Subject will be classified as NGS MRD-positive if he/she will not achieve any NGS MRD-negativity.
Estimand

	**NGS MRD-negativity after the end of ASCT consolidation**
**Treatment**	Arm A vs Arm B
**Population**	ITT population
**Variable**	NGS_conASCT
**Intercurrent Event (Strategy)**	**Treatment discontinuation** (treatment policy strategy, i.e., response evaluation will be considered irrespective of the treatment discontinuation)<br>**Subsequent antimyeloma therapy:** The "while on treatment strategy" will be used. i.e., response evaluation after the start of subsequent antimyeloma therapy will not be considered)
**Population-level summary**	Odds Ratio

Main analysis and related methods
The following data will be reported:

I. Logistic regression model will be used to estimate adjusted Odds Ratio (OR) and the 95% CI for the treatment comparison. The adjustment factors will be ISS and cytogenetic risk FISH (the stratification factor for the randomization). P-value for the endpoint evaluation will be based on Wald's test.
II. NGS MRD negativity rate will be calculated for each treatment group and the corresponding 95% Clopper-Pearson CI will be provided.
III. The subgroup analyses will be performed to determine the treatment effect in different subgroups using interaction terms. The null hypothesis examined with the interaction test is that the OR would be the same in each subgroup. Forest plots will be used to display possible heterogeneity of the treatment effect. The following subgroups will be investigated: ISS, cytogenetic risk FISH.
IV. Time to NGS MRD negativity defined as the time from randomization date to the date of first NGS MRD negative sample. If a patient is on treatment and without NGS MRD negativity, she/he will be censored at the date of last contact. Actuarial estimates of the rate along with the 95% confidence interval (CI) at relevant timepoints will be calculated considering treatment discontinuation as compering event.

6.1.1.1 Supplementary analysis

A supplementary analysis will be performed by changing the analyzed population:

**Population**	subset of ITT population who achieve ≥VGPR within the end of post ASCT consolidation and have an NGS-MRD sample available and evaluable (Positive or Negative)

6.1.2 First key secondary endpoint (NGS MRD-negativity within the end of induction)

<u>Definition</u>
The first key secondary endpoint is the achievement of the NGS MRD-negativity at $\leq 10^{-5}$ sensitivity level (NGS_ind).
At patient' level, NGS_ind is defined as subject who obtains an NGS MRD negative sample within the end of induction.
Subject will be classified as NGS MRD-positive if he/she will not achieve any NGS MRD-negativity.
<u>Estimand</u>

	**NGS MRD-negativity after the end of induction**
**Treatment**	Arm A vs Arm B
**Population**	ITT population
**Variable**	NGS_ind
**Intercurrent Event (Strategy)**	**Treatment discontinuation** (treatment policy strategy, i.e., response evaluation will be considered irrespective of the treatment discontinuation)<br>**Subsequent antimyeloma therapy:** The "while on treatment strategy" will be used. i.e., response evaluation after the start of subsequent antimyeloma therapy will not be considered)
**Population-level summary**	Odds Ratio

<u>Main analysis and related methods</u>
The following data will be reported:

I. Logistic regression model will be used to estimate adjusted Odds Ratio (OR) and the 95% CI for the treatment comparison. The adjustment factors will be ISS and cytogenetic risk FISH (the stratification factor for the randomization). P-value for the endpoint evaluation will be based on Wald's test.
II. NGS MRD negativity rate will be calculated for each treatment group and the corresponding 95% Clopper-Pearson CI will be provided.
III. The subgroup analyses will be performed to determine the treatment effect in different subgroups using interaction terms. The null hypothesis examined with the interaction test is that the OR would be the same in each subgroup. Forest plots will be used to display possible heterogeneity of the treatment effect. The following subgroups will be investigated: ISS, cytogenetic risk FISH.

6.1.2.1 Supplementary analysis

A supplementary analysis will be performed by changing the analyzed population:

**Population**	subset of ITT population who achieve ≥VGPR within the end of induction and have an NGS-MRD sample available and evaluable (Positive or Negative)

6.1.3 Second key secondary endpoint (Progression free survival)

Definition

The second key secondary endpoint is the Progression free survival (PFS).
PFS is defined as the time from randomization date to the date of progression of disease or death due to any cause, whichever occurs first. If the subject has not progressed and is alive, data will be censored at the last disease evaluation.

Estimand

	**Progression free survival**
**Treatment**	Arm A vs Arm B
**Population**	ITT population
**Variable**	PFS
**Intercurrent Event (Strategy)**	**All ICEs** (treatment policy strategy, i.e., pure ITT strategy)
**Population-level summary**	Hazard Ratio

Main analysis and related methods

The following data will be reported:

I. Cox's proportional hazard regression model will be used to estimate adjusted HR and the 95% CI for the treatment comparison. The adjustment factors will be ISS and cytogenetic risk FISH (the stratification factor for the randomization). P-value for the endpoint evaluation will be based on the Wald's test. Grambsch and Therneau test will be used for testing the proportional hazard assumption.
II. Median PFS and PFS rate estimates at relevant timepoints after randomization date along with the 95% CI, will be derived using the Kaplan-Meier method.
III. Actuarial estimates of the reasons for PFS event (i.e., "progression" and "death without progression") at relevant timepoints along with the 95% CI, will be derived using the Aalen-Johansen method.
IV. The subgroup analyses will be performed to determine the treatment effect in different subgroups using interaction terms. The null hypothesis examined with the interaction test is that HR would be the same in each subgroup. Forest plots will be used to display possible heterogeneity of the treatment effect. The following subgroups will be investigated: ISS, cytogenetic risk FISH.

Noninformative censoring for administrative censoring and lost to follow up patients is assumed.

6.1.3.1 Sensitivity analysis

A sensitivity analysis will be performed discarding Death and Progression after an extended lost-to-follow-up time (6 months or more with missed scheduled assessments). The subject will be censored on the date of last adequate assessment with evidence of no progression.

6.1.3.2 Supplementary analysis

In order to controlling for possible confounding due to the maintenance therapy assignment policy, a Cox proportional hazards regression model also adjusted for maintenance therapy will be fit. Based on this, a hypothetical scenario in which different maintenance (based on the collected information on eCRF) therapies are balanced between the two study arms will be investigated.

6.1.4 Secondary Endpoints

6.1.4.1 Efficacy Endpoints

6.1.4.1.1 Time to Progression (TTP)

Definition

A secondary endpoint is the time to progression (TTP).
TTP is defined as the time from the randomization date to the date of progression disease. If the subject has not progressed, data will be censored at the date of last disease evaluation.

Estimand

	**Time to Progression**
**Treatment**	Arm A vs Arm B
**Population**	ITT population
**Variable**	TTP
**Intercurrent Event (Strategy)**	**Subsequent antimyeloma therapy** (treatment policy strategy, i.e., progression disease will be considered irrespective of the start of subsequent antimyeloma therapy) **Treatment discontinuation** (treatment policy strategy, i.e., progression disease will be considered irrespective of treatment discontinuation) **Death** (hypothetical strategy, i.e., Patient will be censored at the date of last disease evaluation)
**Population-level summary**	Hazard Ratio

Main analysis and related methods

The following data will be reported:

I. Cox's proportional hazard regression model will be used to estimate adjusted HR and the 95% CI for the treatment comparison The adjustment factors will be ISS and cytogenetic risk FISH (the stratification factor for the randomization). P-value for the endpoint evaluation will be based on Wald's test. Grambsch and Therneau test will be used for testing the proportional hazard assumption.
II. Median TTP and TTP rate estimates at relevant time points after randomization date along with the 95% CI, will be derived using the Kaplan-Meier method.
III. The subgroup analyses will be performed to determine the treatment effect in different subgroups using interaction terms. The null hypothesis examined with the interaction test is that HR would be the same in each subgroup. Forest plots will be used to display possible heterogeneity of the treatment effect. The following subgroups will be investigated: ISS, cytogenetic risk FISH.

Noninformative censoring for administrative censoring and lost to follow up subjects is assumed.

Supplem**entary analysis**

A supplementary analysis will be performed by changing strategies to handle ICEs:

**Intercurrent Event (Strategy)**	**Treatment discontinuation** (treatment policy strategy, i.e., progression disease will be considered irrespective of treatment discontinuation) **Subsequent antimyeloma therapy** (hypothetical strategy, i.e., patients will be censored at the last disease assessment before starting next line of therapy) **Death** (hypothetical strategy, i.e., Patient will be censored at the date of last disease evaluation)

6.1.4.1.2 Time to next treatment (TNT)

Definition

A secondary endpoint is the time to next treatment (TNT).

TNT is defined as the time from the randomization date to the date of event, which is defined as the start of the next line of therapy or death from any cause, whichever occurs first. If subject is alive and have not started the next line of therapy, subject's data will be censored at the date of last contact.

Estimand

	**Time to next treatment**
**Treatment**	Arm A vs Arm B
**Population**	ITT population
**Variable**	TNT
**Intercurrent Event (Strategy)**	**All possible events** (treatment policy strategy**,** i.e., pure ITT strategy)
**Population-level summary**	Hazard Ratio

Main analysis and related methods

The following data will be reported:

I. Cox's proportional hazard regression model will be used to estimate adjusted HR and the 95% CI for the treatment comparison. The adjustment factors will be ISS and cytogenetic risk FISH (the stratification factor for the randomization). P-value for the endpoint evaluation will be based on Wald's test. Grambsch and Therneau test will be used for testing the proportional hazard assumption.

II. Median TNT and TNT rate estimates at relevant timepoints after randomization date along with the 95% CI, will be derived using the Kaplan-Meier method.

III. Actuarial estimates of competing risks for TNT (i.e., " death" and "start next line of therapy") at relevant timepoint along with the 95% CI, will be derived using the Aalen-Johansen method.

IV. The subgroup analyses will be performed to determine the treatment effect in different subgroups using interaction terms. The null hypothesis examined with the interaction test is that HR would be the same in each subgroup. Forest plots will be used to display possible heterogeneity of the treatment effect. The following subgroups will be investigated: ISS, cytogenetic risk FISH.

Noninformative censoring for administrative censoring and lost to follow up subjects is assumed.

6.1.4.1.3 Progression free survival 2 (PFS2)

Definition

A secondary endpoint is the Progression free survival 2 (PFS2).

PFS2 is defined as the time from the randomization date to the date of event, which is defined as death from any cause or progression disease after the next line of therapy, whichever occurs first. In case of date of second progression is not available, date of start of third line treatment can be used. If subject is alive and without progression on study treatment, subject's data will be censored at the last disease assessment. If subject starts next line of therapy after progression on study treatment and is still alive and not yet progress on next line of therapy, subject's data will be censored on the last date of follow-up.

Estimation

	**Progression free survival 2**
**Treatment**	Arm A vs Arm B
**Population**	ITT population

Variable	PFS2
**Intercurrent Event (Strategy)**	**Subsequent antimyeloma therapy without progression during the study** (hypothetical strategy, i.e., patients will be censored at the last disease assessment before starting next line of therapy)
**Population-level summary**	Hazard Ratio

Main analysis and related methods

The following data will be reported:

I. Cox's proportional hazard regression model will be used to estimate adjusted HR and the 95% CI for the treatment comparison. The adjustment factors will be ISS and cytogenetic risk FISH (the stratification factor for the randomization). P-value for the endpoint evaluation will be based on Wald's test. Grambsch and Therneau test will be used for testing the proportional hazard assumption.

II. Median PFS2 and PFS2 rate estimates at relevant timepoints after randomization date along with the 95% CI, will be derived using the Kaplan-Meier method.

III. Actuarial estimates of competing risks for PFS2 (i.e., " 2nd progression" and "death without 2nd progression") at relevant along with the 95% CI, will be derived using the Aalen-Johansen method.

IV. The subgroup analyses will be performed to determine the treatment effect in different subgroups using interaction terms. The null hypothesis examined with the interaction test is that HR would be the same in each subgroup. Forest plots will be used to display possible heterogeneity of the treatment effect. The following subgroups will be investigated: ISS, cytogenetic risk FISH.

Noninformative censoring for administrative censoring and lost to follow up subjects is assumed.

6.1.4.1.4 Overall survival (OS)

Definition

A secondary endpoint is the Overall survival (OS).
OS is defined as the time from the randomization date to the date of death due to any cause. If the subject is alive at the time of the analysis, data will be censored at the date of last contact.

Estimand

	**Overall survival**
**Treatment**	Arm A vs Arm B
**Population**	ITT population
**Variable**	OS
**Intercurrent Event (Strategy)**	**All possible events** (treatment policy strategy, i.e., pure ITT strategy)
**Population-level summary**	Hazard Ratio

Main analysis and related methods

The following data will be reported:

I. Cox's proportional hazard regression model will be used to estimate adjusted HR and the 95% CI for the treatment comparison. The adjustment factors will be ISS and cytogenetic risk FISH (the stratification factor for the randomization). P-value for the endpoint evaluation will be based on Wald's test. Grambsch and Therneau test will be used for testing the proportional hazard assumption.
II. Median OS and OS rate estimates at relevant timepoints after randomization date along with the 95% CI, will be derived using the Kaplan-Meier method.
III. Actuarial estimates of competing risks for OS (i.e., " death for PD" and "death for any other reason") at relevant timepoints along with the 95% CI, will be derived using the Aalen-Johansen method.
IV. The subgroup analyses will be performed to determine the treatment effect in different subgroups using interaction terms. The null hypothesis examined with the interaction test is that HR would be the same in each subgroup. Forest plots will be used to display possible heterogeneity of the treatment effect. The following subgroups will be investigated: ISS, cytogenetic risk FISH.

Noninformative censoring for administrative censoring and lost to follow up patients is assumed.

[redacted]

6.1.4.1.5 Duration of NGS MRD negativity (DMRD)

Definition

A secondary endpoint is the duration of MRD negativity (DMRD).

DMRD is defined as the time from the date of first NGS MRD negative sample to the date of progression disease or subsequent NGS MRD positive sample, whichever occurs first. If the subject has not progressed and without subsequent NGS MRD positive sample, data will be censored at the last disease evaluation.

Estimand

	**Duration of NGS MRD negativity**
**Treatment**	Arm A vs Arm B
**Population**	ITT population who achieves NGS MRD negativity
**Variable**	DMRD
**Intercurrent Event (Strategy)**	**Treatment discontinuation** (treatment policy strategy, i.e., progression disease/MRD positivity will be considered irrespective of treatment discontinuation)<br>**Death** (hypothetical strategy, i.e., Patient will be censored at the date of last disease evaluation)
**Population-level summary**	Hazard Ratio

Main analysis and related methods

The following data will be reported:

I. Cox's proportional hazard regression model will be used to estimate adjusted HR and the 95% CI for the treatment comparison. The adjustment factors will be ISS and cytogenetic risk FISH (the stratification factor for the randomization). P-value for the endpoint evaluation will be based on the Wald's test. Grambsch and Therneau test will be used for testing the proportional hazard assumption.

II. Median DMRD and DMRD rate estimates at relevant timepoints after the date of first NGS MRD negative sample along with the 95% CI, will be derived using the Kaplan-Meier method.

III. Actuarial estimates of the reasons for DMRD event (i.e., "progression" and "MRD positivity") at relevant timepoints along with the 95% CI, will be derived using the Aalen-Johansen method.

IV. The subgroup analyses will be performed to determine the treatment effect in different subgroups using interaction terms. The null hypothesis examined with the interaction test is that HR would be the same in each subgroup. Forest plots will be used to display possible heterogeneity of the treatment effect. The following subgroups will be investigated: ISS, cytogenetic risk FISH.

6.1.4.1.6 Duration of response (DOR)

Definition

A secondary endpoint is the duration of response (DOR).

DOR is defined as the time from the date of first documented ≥PR response to the date of progression disease or death due to any cause, whichever occurs first. If the subject has not progressed and is alive, data will be censored at the last disease evaluation.

Estimand

	**Duration of response**
**Treatment**	Arm A vs Arm B
**Population**	ITT population who achieves ≥PR response
**Variable**	DOR
**Intercurrent Event (Strategy)**	**Subsequent antimyeloma therapy** (treatment policy strategy, i.e., progression disease/death will be considered irrespective of the start of subsequent antimyeloma therapy)<br>**Treatment discontinuation** (treatment policy strategy, i.e., progression disease/death will be considered irrespective of treatment discontinuation)
**Population-level summary**	Hazard Ratio

Main analysis and related methods

The following data will be reported:

I. Cox's proportional hazard regression model will be used to estimate adjusted HR and the 95% CI for the treatment comparison. The adjustment factors will be ISS and cytogenetic risk FISH (the stratification factor for the randomization). P-value for the endpoint evaluation will be based on the Wald's test. Grambsch and Therneau test will be used for testing the proportional hazard assumption.

II. Median DOR and DOR rate estimates at relevant timepoints after the date of first documented ≥PR response along with the 95% CI, will be derived using the Kaplan-Meier method.

III. Actuarial estimates of the reasons for DOR event (i.e., "progression" and "death without progression") at relevant timepoints along with the 95% CI, will be derived using the Aalen-Johansen method.

IV. The subgroup analyses will be performed to determine the treatment effect in different subgroups using interaction terms. The null hypothesis examined with the interaction test is that HR would be the same in each subgroup. Forest plots will be used to display possible heterogeneity of the treatment effect. The following subgroups will be investigated: ISS, cytogenetic risk FISH

6.1.4.1.7 NGS MRD-negativity after the end of ASCT/ light consolidation

Definition

Secondary endpoints are the achievement of the NGS MRD-negativity at ≤10^{-5} sensitivity level (NGS_asct, NGS_light).

At patient' level, NGS_asct and NGS_light are defined as subject who obtains an NGS MRD negative sample within the end of ASCT/ light consolidation respectively.

Subject will be classified as NGS MRD-positive if he/she will not achieve any NGS MRD-negativity at the end of ASCT/ light consolidation respectively.

Estimand

	**NGS MRD-negativity after the end of ASCT/ light consolidation**
**Treatment**	Arm A vs Arm B
**Population**	ITT population
**Variable**	NGS_asct, NGS_light
**Intercurrent Event (Strategy)**	**Treatment discontinuation** (treatment policy strategy, i.e., response evaluation will be considered irrespective of the treatment discontinuation)<br>**Subsequent antimyeloma therapy:** The "while on treatment strategy" will be used. i.e., response evaluation after the start of subsequent antimyeloma therapy will not be considered)
**Population-level summary**	Odds Ratio

Main analysis and related methods

The following data will be reported:

I. Logistic regression model will be used to estimate adjusted Odds Ratio (OR) and the 95% CI for the treatment comparison. The adjustment factors will be ISS and cytogenetic risk FISH (the stratification factor for the randomization). P-value for the endpoint evaluation will be based on Wald's test.

II. NGS MRD negativity rate will be calculated for each treatment group and the corresponding 95% Clopper-Pearson CI will be provided.

III. The subgroup analyses will be performed to determine the treatment effect in different subgroups using interaction terms. The null hypothesis examined with the interaction test is that the OR would be the same in each subgroup. Forest plots will be used to display possible heterogeneity of the treatment effect. The following subgroups will be investigated: ISS, cytogenetic risk FISH.

Supplementary analysis

A supplementary analysis will be performed by changing the analyzed population:

**Population**	subset of ITT population who achieve ≥VGPR within the end of ASCT/light consolidation and have an NGS-MRD sample available and evaluable (Positive or Negative)

6.1.4.1.8 NGS MRD sustained negativity

<u>Definition</u>

A secondary endpoint is the NGS MRD sustained negativity (NGSsust) at $\leq 10^{-5}$ sensitivity level.
At patient's level, NGSsust is defined as subject who achieved two consecutive NGS-MRD (6,12,18 month apart or more).
Subject will be classified as no-sustained if he/she will not achieve an NGS MRD sustained negativity.

<u>Estimand</u>

	**NGS MRD sustained negativity**
**Treatment**	Arm A vs Arm B
**Population**	ITT population
**Variable**	NGSsust
**Intercurrent Event (Strategy)**	**Treatment discontinuation** (treatment policy strategy, i.e., response evaluation will be considered irrespective of the treatment discontinuation)<br>**Subsequent antimyeloma therapy:** The "while on treatment strategy" will be used. i.e., response evaluation after the start of subsequent antimyeloma therapy will not be considered)
**Population-level summary**	Odds Ratio

<u>Main analysis and related methods</u>

The following data will be reported:

I. Logistic regression model will be used to estimate adjusted OR and the 95% CI for the treatment comparison. The adjustment factors will be ISS and cytogenetics risk FISH (the stratification factor for the randomization). P-value for the endpoint evaluation will be based on Wald's test.
II. NGS MRD sustained negativity rate will be calculated for each treatment group and the corresponding 95% Clopper-Pearson CI will be provided.
III. The subgroup analyses will be performed to determine the treatment effect in different subgroups using interaction terms. The null hypothesis examined with the interaction test is that the odds ratio (OR) would be the same in each subgroup. Forest plots will be used to display possible heterogeneity of the treatment effect. The following subgroups will be investigated: ISS, cytogenetic risk FISH.

6.1.4.1.9 IMWG Best response

<u>Definition</u>

A secondary endpoint is the IMWG Best response (resp).
At patient's level, IMWG Best response is defined as the IMWG best response achieved by a patient during the study.
Subject will be classified as Not Evaluable (NE) if he/she will not have any evaluable response assessment.

<u>Estimand</u>

	**IMWG Best response**
**Treatment**	Arm A vs Arm B
**Population**	ITT population
**Variable**	resp

**Intercurrent Event (Strategy)**	**Treatment discontinuation** (treatment policy strategy, i.e., response evaluation will be considered irrespective of the treatment discontinuation)<br>**Subsequent antimyeloma therapy:** The “while on treatment strategy” will be used. i.e., response evaluation after the start of subsequent antimyeloma therapy will not be considered)
**Population-level summary**	Odds Ratio

<u>**Main analysis and related methods**</u>

The following data will be reported:

I. Logistic regression model will be used to estimate adjusted OR the 95% CI for the treatment comparison. The adjustment factors will be ISS and cytogenetics risk FISH (the stratification factor for the randomization). P-value for the endpoint evaluation will be based on Wald’s test. The following binary category will be considered: sCR vs ≤CR, ≥CR vs ≤VGPR, ≥VGPR vs ≤PR, ≥PR vs ≤SD.

II. sCR, ≥CR, ≥nCR, ≥VGPR, ≥PR rate will be calculated for each treatment group and the corresponding 95% Clopper-Pearson CI will be provided.

III. The subgroup analyses will be performed to determine the treatment effect in different subgroups using interaction terms. The null hypothesis examined with the interaction test is that the OR would be the same in each subgroup. Forest plots will be used to display possible heterogeneity of the treatment effect. The following subgroups will be investigated: ISS, cytogenetic risk FISH

IV. Time to sCR, CR, nCR, VGPR and PR defined as the time from randomization date to the date of first documented sCR, CR, nCR, VGPR and PR. Actuarial estimates of the rate along with the 95% confidence interval (CI) at relevant timepoints will be calculated considering treatment discontinuation as compering event. If a patient is on treatment and without achieving a specific response, she/he will be censored at the date of last contact.

6.1.4.1.10 IMWG Best response within the end of each treatment phase

<u>**Definition**</u>

Secondary endpoints are the IMWG Best response within the end of each treatment phase (induction [resp_IND], ASCT [resp_ASCT], ASCT consolidation [resp_CON] and light consolidation [resp_LIGH])
At patient’s level, IMWG Best response is defined as the IMWG best response achieved by a patient within the end of each treatment phase. Response evaluations after an early termination of the study treatment will be discarded.
Subject will be classified as Not Evaluable (NE) if he/she will not have any evaluable response assessment.

<u>**Estimand**</u>

	**IMWG Best response within the end of each treatment phase**
**Treatment**	Arm A vs Arm B
**Population**	ITT population
**Variable**	resp_IND, resp_ASCT, resp_CON, resp_LIGH
**Intercurrent Event (Strategy)**	**Treatment discontinuation** (The “while on treatment strategy” will be used. i.e., response evaluation after a treatment discontinuation will not be considered).
**Population-level summary**	Odds Ratio

Main analysis and related methods

The following data will be reported:

I. Logistic regression model will be used to estimate adjusted OR the 95% CI for the treatment comparison. The adjustment factors will be ISS and cytogenetics risk FISH (the stratification factor for the randomization). P-value for the endpoint evaluation will be based on Wald's test. The following binary category will be considered: sCR vs ≤CR, ≥CR vs ≤VGPR, ≥VGPR vs ≤PR, ≥PR vs ≤SD.

II. sCR, ≥CR, ≥nCR, ≥VGPR, ≥PR rate will be calculated for each treatment group and the corresponding 95% Clopper-Pearson CI will be provided.

III. The subgroup analyses will be performed to determine the treatment effect in different subgroups using interaction terms. The null hypothesis examined with the interaction test is that the OR would be the same in each subgroup. Forest plots will be used to display possible heterogeneity of the treatment effect. The following subgroups will be investigated: ISS, cytogenetic risk FISH

[redacted]

6.1.4.2 Safety Endpoints

6.1.4.2.1 Adverse Events

Adverse Events (AEs) will be summarized by arm and for the safety, induction, post ASCT consolidation and light consolidation population.
AEs are documented on the eCRF together with their grade, according to the NCI CTCAE version 5.0.
For the categorization, the MedDRA® dictionary (version 23.1 or higher) will be used to group AEs for SOC (System Organ Class) and PT (Preferred Term).
AEs will be considered related to the study drugs when the relation to trial medication is suspected according to the investigator's opinion. In particular, AEs will be considered related if the value (for at least one of the study drugs) reported on eCRF is one of the following: Possibly related, Probably related, Definitely related.
Treatment Emergent Adverse Events (TEAEs) are defined:

I. as any AE/SAE that occurs at or after the start of study treatment until 30 days after the last dose of study treatment or the start of subsequent therapy, whichever occurs first.
II. any AE/SAE that is considered related to any study drug regardless of the start date of the event.
III. any AE/SAE that is present at baseline but worsens in toxicity grade or is subsequently considered treatment-related by the investigator.

The analysis of treatment toxicity will be done primarily by tabulation of the incidence of adverse CTCAE grade 2, 3 or 4. A subject having the same event more than once will be counted only once for the worst grade.
AEs will be summarized as follow:

I. TEAEs in each treatment phase (induction, mobilization +ASCT, post ASCT consolidation and light consolidation) separately, during premaintenance, maintenance and overall.
II. TEAEs related in each treatment phase (induction, mobilization +ASCT, post ASCT consolidation and light consolidation) separately, during premaintenance, maintenance and overall.
III. SAEs in each treatment phase (induction, mobilization +ASCT, post ASCT consolidation and light consolidation) separately, during premaintenance, maintenance and overall.

In the case that the Adverse Events or event frequencies are judged to be clinically important, an exact test will be used to analyze the difference between treatment arms.
TEAEs, having CTCAE grade or onset date missing on eCRF, will be summarize and excluded from the main analysis. TAES with 'Relationship to Isa/Carf/Len/Dex/Cyclo/Mel' missing on eCRF, will be considered Not related. TAES with 'Action taken with Isa/Carf/Len/Dex/Cyclo/Mel' missing on eCRF, will be considered as 'Dose not changed'.
TEAEs will be also considered as proportion of patients experiencing at least one of:

I. hematologic Adverse Event (AE)
II. non-hematologic AE
III. Adverse Event leading to treatment discontinuation
IV. Adverse Event leading to treatment reduction

In addition, details of administration of G-CSF, EPO, transfusion and infection prophylaxis will be tabulated.

6.1.4.2.2 Serious Adverse Events

For Serious Adverse Events (SAEs) the following additional tables will be displayed:

I. At the patient level, a table will be generated with the number of SAEs per patient reported.

[redacted]

II. At the SAE level, a table will be generated with

- seriousness criteria [Death vs Life-threatening vs (Prolongation of) hospitalization vs Significant /persistent disability vs Congenital anomaly/birth defect vs Other medically important condition/ AESI]
- outcome [Recovered vs Recovered with sequelae vs Not yet recovered vs Death vs Ongoing at the end of the study vs Unknown (lost to follow up) vs Not applicable]
- CTCAE grade
- relation to each study drug [Not related, Unlikely, Possible, Probable, Definite, Not assessable]
- Cycle/Phase when the SAE occurred
- SAE leading to drugs reduction
- SAE leading to drugs discontinuation
- Type of report

Moreover, a list with all SAEs will be shown with the following information:

I. SAE progressive number
II. Type of report
III. Date of awareness
IV. SAE verbatim and SOC
V. Onset date and cycle
VI. Seriousness criteria
VII. Severity of AE
VIII. Relationship to drugs
IX. Action taken
X. Outcome
XI. Date SAE recovered/Date of death
XII. SAE description and comments

6.1.4.2.3 Death

Death data will be summarized by arm for the safety population and for the patients who will not start treatment separately. The following information will be tabulated:

I. The number of patients who died and the reason.
II. For deaths due to AE, an additional table showing SOC and PT will be displayed
III. For deaths due to Other, an additional table showing narratives will be displayed

Moreover, a list with all patients died will be shown with the following information:

I. Participants who died and baseline characteristics.
II. Causes of death [Adverse Event vs Progression disease vs Other vs Unknown]
III. If the cause of death is an AE, some details of the AE will be reported (relation to the study treatment and TEAE indicator). If the cause of death is Other, additional details will be reported.
IV. Time from randomization to death
V. Time from randomization to discontinuation
VI. Time from randomization to progression disease (if present)
VII. Type of subsequent line of therapy after EMN24 (if present)

6.1.4.2.4 Study drug exposure

The following measures of drugs exposure will be analyzed for the induction population, post ASCT consolidation population and light consolidation population and for each study drug separately:

I. Patients that started treatment, each treatment phase or other relevant timepoint;
II. Treatment duration defined as the time from the start of therapy to the end of last cycle or treatment/drug discontinuation, if the patient discontinued treatment/drug; treatment duration could be defined for a restricted number of cycles excluding data not related to these cycles and applying the previous definition;
III. Cumulative dose defined as the sum of all doses taken during relevant time windows;
IV. Dose exposure defined as the number of administration days during relevant time windows;
V. Average Daily dose defined as the cumulative dose divided by dose exposure;
VI. Dose intensity defined as the cumulative dose divided by treatment duration;
VII. Relative dose intensity is defined as the dose intensity divided by the planned dose intensity
VIII. Number of drug reductions and causes during relevant time windows;
IX. Drug discontinuation and reason.

Descriptive statistics will be presented by arm and overall.
Relevant time windows include all treatment phase separately and the entire treatment period.

In addition, time to first reduction calculated as time from start therapy to first reduction of any study drug will be computed; actuarial estimates of the rate of reduction along with the 95% confidence interval (CI) at relevant timepoints will be calculated using the compering risk method.

6.1.4.3 Patient Reported Outcomes

Definition
Symptoms, functional status and well-being will be assessed using the EORTC QLQ-C30.
The EORTC QLQ-C30 includes 30 items, with a 1-week recall, resulting in 5 functional scales (physical functioning, role functioning, emotional functioning, cognitive functioning, and social functioning), 1 Global Health Status scale, 3 symptom scales (fatigue, nausea and vomiting, and pain), and 6 single items (dyspnea, insomnia, appetite loss, constipation, diarrhea, and financial difficulties).
The EQ 5D 5L is a generic measure of health status. The EQ 5D 5L is a 5-item questionnaire that assesses 5 domains including mobility, self-care, usual activities, pain/discomfort and anxiety/depression plus a visual analog scale rating (VAS) with values ranging from 0 (worst) to 100 (best).
Estimand

	**Duration of response**
**Treatment**	Arm A vs Arm B
**Population**	Safety population with baseline and at least one post-baseline questionnaire.
**Variable**	• EORTC QLQ-C30 variables: physical functioning, role functioning, emotional functioning, cognitive functioning, and social functioning, Global Health Status scale, dyspnea, insomnia, appetite loss, constipation, diarrhea, and financial difficulties<br>• EQ 5D 5L: VAS
**Intercurrent Event (Strategy)**	**Subsequent antimyeloma therapy** The "while on treatment strategy" will be used. i.e., questionnaire after start of subsequent antimyeloma therapy will not be considered) **Treatment discontinuation** (treatment policy strategy, i.e., questionnaire will be considered irrespective of treatment discontinuation)
**Population-level summary**	Difference in Least square mean (LS Mean) changes from baseline

Main analysis and related methods

I. Compliance rates for completion of each PRO at each time point will be generated based on number and percentage of expected, received and missing PRO assessments. Descriptive statistics will be provided for all PRO endpoints at each time point.
II. Mixed effects model with repeated measures will be conducted. Mixed effects models will include the following factors: participants as a random effect, baseline value, randomization factors, timepoint, treatments arms and the interaction between time and treatment arms (as fixed effects).
III. In addition, time to event analyses will be conducted for the key PRO. Time to first clinically meaningful deterioration relative to baseline a change of ≥ 8 points for the EORTC QLQ-C30 GHS score; a change of ≥ 10 in the EORTC QLQ-C30 functional and symptom scores and a change of ≥ 7 points for the EQ-5D-5L VAS score

6.1.4.4 Other Endpoints

6.1.4.4.1 Mobilization

For each stem cells collection session and overall, the following data will be shown:

I. Patients that started the procedure;

II. Time from the end date of Induction to the date of first drug administration for mobilization
III. Type of collection method (PBSC pheresis or bone marrow harvest)
IV. Details of drugs administration (G-CSF, Cyclophosphamide, Plerixafor, Other);
V. Number of CD34+ cells collected [0-2 vs 2-4 vs > 4] million cells and as continuous number.
VI. Days of leukapheresis;
VII. Patients unable to receive ASCT due to poor stem cells collection [Yes vs No];

Descriptive statistics will be presented by arm and eventually by site and country.
Moreover, the effect of treatment arms (and other factors) on mobilization outcome will be tested. These factors include baseline features and pre-mobilization efficacy/safety outcome (NGS-MRD negativity, IMWG response..)

6.1.4.4.2 ASCT

The following data about ASCT will be show:

I. Patients that started the procedure;
II. Details of Melphalan administration;
III. Patients with stem cells infusion [Yes vs No];
IV. Total number of CD34+ cells infused;
V. Time from Melphalan administration date to the date of hematologic recovery (3 consecutive days with at least ANC 0.5 10^9/L and 7 consecutive days with at least Platelet count 20 10^9/L without transfusion or 3 consecutive days with at least Platelet count 50 10^9/L without transfusion)

Descriptive statistics will be presented by arm.

6.1.4.4.3 Maintenance

The following data about maintenance will be show:

I. Patients that started maintenance or not;
II. Type of maintenance [fixed therapy vs continuous therapy];
III. Details of drugs administered during maintenance therapy;
IV. Maintenance duration defined as the time from the start of maintenance to the end of maintenance, if a patient is on maintenance at time of analysis she/he will be censored at the date of last contact; actuarial estimates of the reasons for end of maintenance (i.e., "progression" and "no progression") at relevant timepoints along with the 95% CI, will be derived using the Aalen-Johansen method.
V. Reason for end of maintenance.

Descriptive statistics will be presented by arm.

6.1.4.4.4 Subsequent lines of therapy

For patients who relapsed during EMN24 treatment (or during maintenance) and so before the start of a subsequent therapy, the following data will be shown:

I. Patient status [Death within 3 months from progression vs Death after 3 months from progression vs Next line of therapy within 3 months from progression vs Next line of therapy

after 3 months from progression vs Censored within 3 months from progression vs Censored after 3 months from progression]

II. Details of next line of treatment
III. Time to next therapy from relapse calculated as time from the progression to the start of a subsequent therapy or death whichever occurs first. Actuarial estimates of the reasons for TNT event (i.e., “death” and “start subsequent line”) at relevant timepoints from relapse will be calculated using the compering risk method.

For patients who relapsed after the start of a subsequent therapy (excluding maintenance which is considered here part of EMN24 treatment), the following data will be shown:

I. Causes of early treatment discontinuation
II. Details of next line of treatment
III. Response at the date of treatment discontinuation

6.1.4.4.5 Agreement between MRD techniques

Agreement and disagreement in the MRD measurements by NGS, NGF and MS will be investigated by calculating the proportion of samples with concordant results over all samples that will be simultaneously assessed. Analyses will be performed by treatment arm, by treatment phase and overall.

The Cohen’s kappa (κ) statistic will be also presented for evaluating the concordance between MRD techniques.

6.2 Analysis methods

6.2.1 Supplementary analyses

To support the study results, the primary analyses will be reproduced not only for the ITT population, but also for the modified ITT population that includes all screened patients.

6.3 Statistical software

Data will be analyzed using R language and environment for statistical computing (R Foundation for Statistical Computing, Vienna, Austria - Version 4.2.1 or higher).

A. Treatment Schema

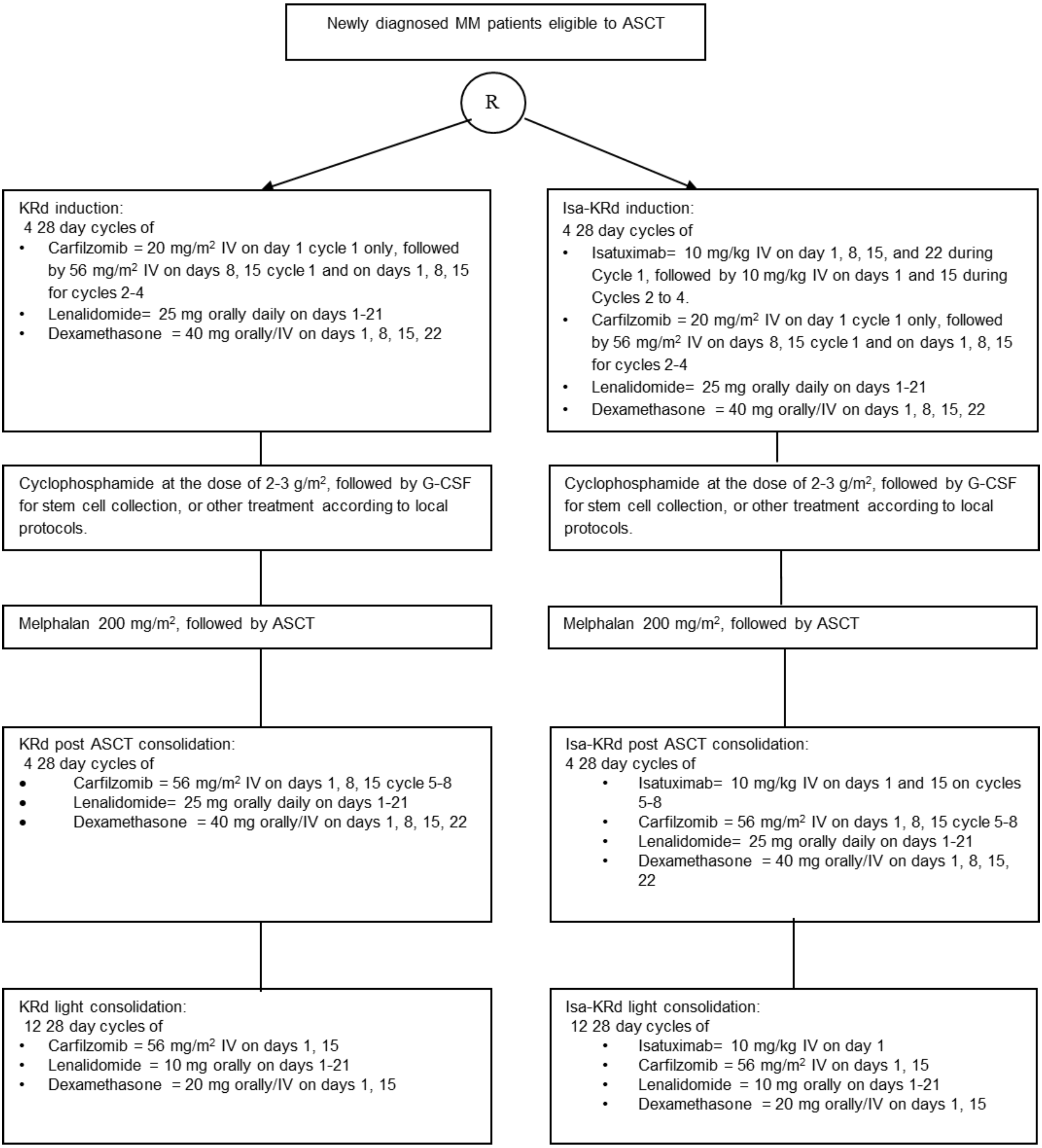

B. Consort Diagram

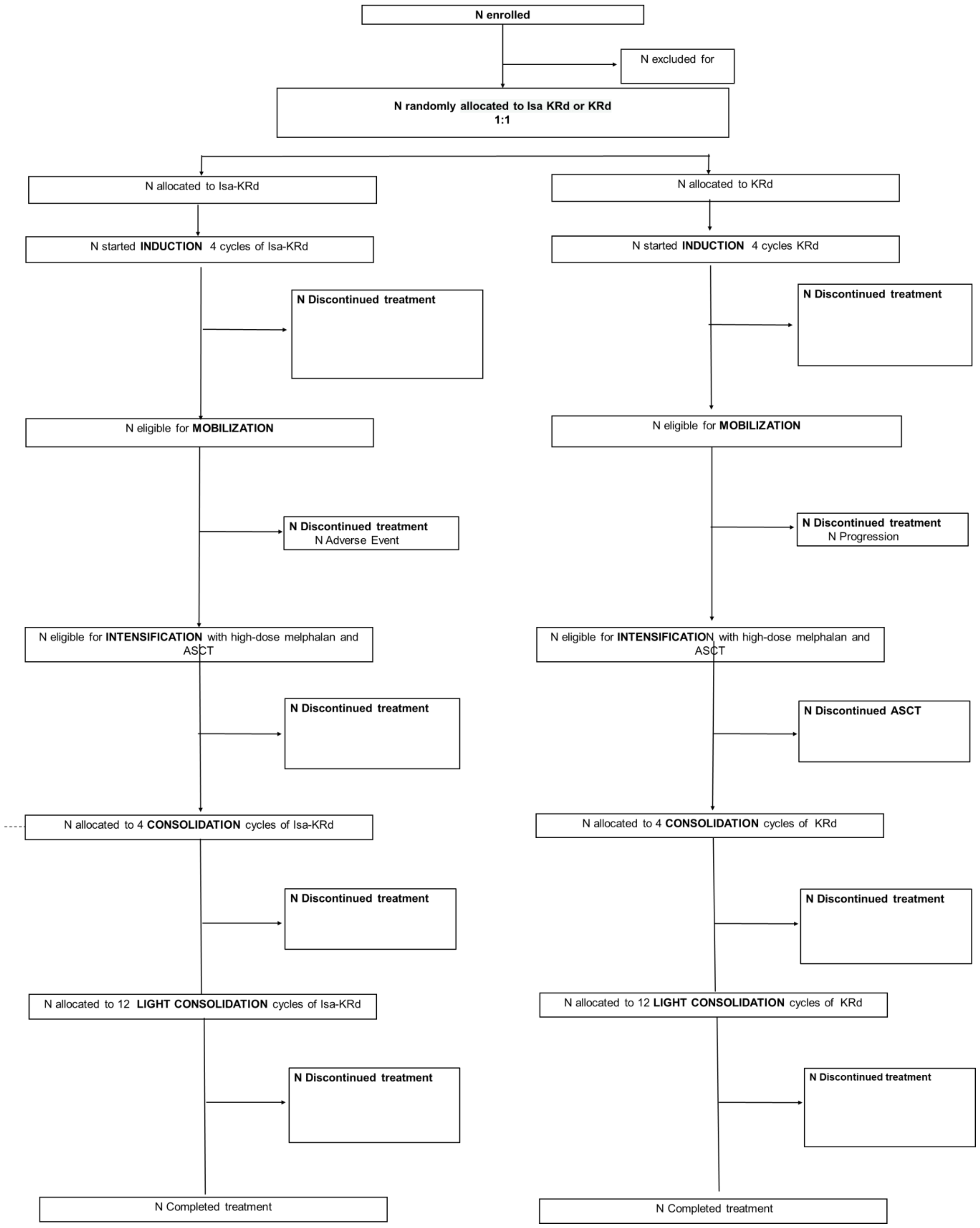

C. References

I. Gamble C, Krishan A, Stocken D, et al. Guidelines for the Content of Statistical Analysis Plans in Clinical Trials. JAMA. 2017;318(23):2337–2343. doi:10.1001/jama.2017.18556

II. Mallinckrodt, Craig & Molenberghs, Geert & Lipkovich, Ilya & Ratitch, Bohdana. (2019). Estimands, Estimators and Sensitivity Analysis in Clinical Trials. 10.1201/9780429488825.

III. Perrot, Aurore & Facon (2020). Health-Related Quality of Life in Transplant-Ineligible Patients With Newly Diagnosed Multiple Myeloma: Findings From the Phase III MAIA Trial. Journal of Clinical Oncology. 39. JCO.20.01370. 10.1200/JCO.20.01370.

IV. Lokhnygina Y, Helterbrand JD. Cox regression methods for two-stage randomization designs. Biometrics 2007; 63: 422–28.
