# Supplementary material for: Isatuximab, carfilzomib, lenalidomide and dexamethasone in newly diagnosed multiple myeloma: a randomized phase 3 trial
Source: Nat Med. 2026 Apr 6;32(5):1773–82. doi: 10.1038/s41591-026-04282-0 (PMC13190300; doi:10.1038/s41591-026-04282-0)
Supplement: Supplementary file 2 — Reporting Summary [file 41591_2026_4282_MOESM2_ESM.pdf]

Corresponding author(s): Francesca Gay
Last updated by author(s): Mar 3, 2026

Reporting Summary

Nature Portfolio wishes to improve the reproducibility of the work that we publish. This form provides structure for consistency and transparency in reporting. For further information on Nature Portfolio policies, see our Editorial Policies and the Editorial Policy Checklist.

Statistics

For all statistical analyses, confirm that the following items are present in the figure legend, table legend, main text, or Methods section.

n/a	Confirmed	
☐	☒	The exact sample size (*n*) for each experimental group/condition, given as a discrete number and unit of measurement
☐	☒	A statement on whether measurements were taken from distinct samples or whether the same sample was measured repeatedly
☐	☒	The statistical test(s) used AND whether they are one- or two-sided<br>*Only common tests should be described solely by name; describe more complex techniques in the Methods section.*
☐	☒	A description of all covariates tested
☐	☒	A description of any assumptions or corrections, such as tests of normality and adjustment for multiple comparisons
☐	☒	A full description of the statistical parameters including central tendency (e.g. means) or other basic estimates (e.g. regression coefficient) AND variation (e.g. standard deviation) or associated estimates of uncertainty (e.g. confidence intervals)
☐	☒	For null hypothesis testing, the test statistic (e.g. *F*, *t*, *r*) with confidence intervals, effect sizes, degrees of freedom and *P* value noted<br>*Give P values as exact values whenever suitable.*
☒	☐	For Bayesian analysis, information on the choice of priors and Markov chain Monte Carlo settings
☐	☒	For hierarchical and complex designs, identification of the appropriate level for tests and full reporting of outcomes
☒	☐	Estimates of effect sizes (e.g. Cohen's *d*, Pearson's *r*), indicating how they were calculated

Our web collection on statistics for biologists contains articles on many of the points above.

Software and code

Policy information about availability of computer code

Data collection	The EMN24 IsKia trial was conducted using the Electronic Case Report Form (eCRF). Study data were collected and managed using REDCap (Research Electronic Data Capture, developed by the REDCap Consortium) electronic data capture tools.
Data analysis	All statistical analyses were performed using R software (v4.2.1).

For manuscripts utilizing custom algorithms or software that are central to the research but not yet described in published literature, software must be made available to editors and reviewers. We strongly encourage code deposition in a community repository (e.g. GitHub). See the Nature Portfolio guidelines for submitting code & software for further information.

Data

Policy information about availability of data

All manuscripts must include a data availability statement. This statement should provide the following information, where applicable:

- Accession codes, unique identifiers, or web links for publicly available datasets
- A description of any restrictions on data availability
- For clinical datasets or third party data, please ensure that the statement adheres to our policy

Data supporting this article are part of an ongoing clinical trial and are consequently not publicly available. After the publication of this article, deidentified data collected for this analysis and related documents will be made available to others upon reasonably justified request, which needs to be written and addressed to the attention of the sponsor of the EMN24 IsKia trial, the European Myeloma Network (EMN), at the following e-mail address: info@emn.life. The European

Myeloma Network (EMN) is responsible to evaluate and eventually accept or refuse every request to disclose data and their related documents, in compliance with the ethical approval conditions, in compliance with applicable laws and regulations, and in conformance with the agreements in place with the involved subjects, the participating institutions, and all the other parties directly or indirectly involved in the participation, conduct, development, management and evaluation of this analysis. Response will typically be given in 3 months. The trial Protocol and Statistical analysis plan can be found in the Supplementary Information file.

Research involving human participants, their data, or biological material

Policy information about studies with human participants or human data. See also policy information about sex, gender (identity/presentation), and sexual orientation and race, ethnicity and racism.

Reporting on sex and gender	Information about patient sex was requested at registration. Patient sex was self-reported; the provided options were "male" or "female". The research findings did not apply to only one sex or gender. A disaggregated analysis by sex was not performed because sex is not thought to be a major confounder in the multiple myeloma field. Sex was collected and reported (see Table 1).
Reporting on race, ethnicity, or other socially relevant groupings	N/A
Population characteristics	Transplant-eligibile patients with symptomatic newly diagnosed multiple myeloma aged 18–70 years with measurable disease defined according to standard criteria were eligible. Other inclusion criteria were Eastern Cooperative Oncology Group Performance Status ≤2, life expectancy >3 months, absolute neutrophil count ≥1×10⁹/L, platelet count ≥75×10⁹/L, left ventricular ejection fraction ≥40%, and creatinine clearance ≥30 ml/min. Exclusion criteria included other malignancies within the past 3 years, peripheral neuropathy of grade >2 or grade 2 with pain, unstable angina or myocardial infarction within 4 months before randomization, New York Heart Association functional class III/IV heart failure, uncontrolled angina, uncontrolled hypertension, pulmonary embolism within the last 5 years, history of severe coronary artery disease, severe uncontrolled ventricular arrhythmias, sick sinus syndrome or electrocardiographic evidence of acute ischemia or grade 3 conduction system abnormalities unless the patient had a pacemaker, and any notable clinical condition that placed the patient at a substantial risk if they participated in the trial. A complete list of inclusion and exclusion criteria is reported in the Redacted Trial Protocol (included in the Supplementary Information file).
Recruitment	The EMN24 IsKia trial is a randomized, open-label, phase III trial that enrolled patients from 42 European centers in 8 countries (see the Supplementary Information file). Eligible patients were enrolled during the enrollment phase, which is now closed, and gave written informed consent before participating in the trial. Patients were informed of the trial enrollment phase by study staff.
Ethics oversight	This trial and its protocol and amendments were approved by the ethics or institutional review boards at each of the participating centers (ethics committee of the coordinating center Comitato etico territoriale Lombardia 6, Italy). For a complete list of the participating centers, please see p. 2 of the Supplementary Information file. All patients gave written informed consent before participating in the trial, which was conducted in accordance with the Declaration of Helsinki and Good Clinical Practice guidelines.

Note that full information on the approval of the study protocol must also be provided in the manuscript.

Field-specific reporting

Please select the one below that is the best fit for your research. If you are not sure, read the appropriate sections before making your selection.

☒ Life sciences ☐ Behavioural & social sciences ☐ Ecological, evolutionary & environmental sciences

For a reference copy of the document with all sections, see nature.com/documents/nr-reporting-summary-flat.pdf

Life sciences study design

All studies must disclose on these points even when the disclosure is negative.

Sample size	The calculation of the sample size for the primary endpoint was done with the following assumptions, considering the intention-to-treat (ITT) population: α=0.05 (two-sided); β=0.10; post-autologous stem-cell transplantation (post-ASCT) full-dose consolidation measurable residual disease (MRD) negativity (sensitivity of 10^-5, by next-generation sequencing [NGS]) rate: isatuximab-carfilzomib-lenalidomide-dexamethasone (Isa-KRd) 64% vs. carfilzomib-lenalidomide-dexamethasone (KRd) 45%. The total number of patients required was 300 (by the χ2 test with Yates' continuity correction).<br>The power of 85% (β=0.15) for the first key secondary endpoint (post-induction MRD negativity rate by NGS) was evaluated with the χ2 test with Yates' continuity correction, with the following assumptions, considering the ITT population: α=0.05 (two-sided); post-induction MRD negativity (sensitivity of 10-5; by NGS) rate: Isa-KRd 30% vs. KRd 15%. The power of 92% (β=0.08) for the second key secondary endpoint (progression-free survival [PFS]) was evaluated with the Schoenfeld formula, with the following assumptions, considering the ITT population: α=0.05 (two-sided), 60-month PFS: Isa-KRd 80% vs. KRd: 60% (HR 0.44). According to the latest protocol amendment (currently under approval), the achievement of 92% of power requires 68 PFS events, which are expected to occur 53 months after the date of the last randomization.<br>A hierarchical testing procedure was used for the primary and key secondary endpoints to achieve control of the overall familywise type I error rate at a two-sided significance level of 0.05.

Data exclusions	No data exclusion was performed.
Replication	N/A (clinical trial)
Randomization	At enrollment, a computer system randomly assigned patients (1:1) to treatment into one of the two induction/consolidation arms. Patients were stratified according to International Staging System (ISS) stage (I vs. II vs. III) and cytogenetic risk assessed by fluorescence in situ hybridization (high risk [presence of t(4;14) and/or t(14;16) and/or del(17p)] vs. standard risk/missing [none of these abnormalities]) and then randomized using a web-based, computer-generated procedure completely concealed from study participants. Randomization was performed according to a randomization list, which was created by statisticians, using dynamic sizes of blocks (from 2 to 6) for each stratum.
Blinding	N/A (this is an open-label trial).

Reporting for specific materials, systems and methods

We require information from authors about some types of materials, experimental systems and methods used in many studies. Here, indicate whether each material, system or method listed is relevant to your study. If you are not sure if a list item applies to your research, read the appropriate section before selecting a response.

Materials & experimental systems

n/a	Involved in the study
☒	☐ Antibodies
☒	☐ Eukaryotic cell lines
☒	☐ Palaeontology and archaeology
☒	☐ Animals and other organisms
☐	☒ Clinical data
☒	☐ Dual use research of concern
☒	☐ Plants

Methods

n/a	Involved in the study
☒	☐ ChIP-seq
☒	☐ Flow cytometry
☒	☐ MRI-based neuroimaging

Clinical data

Policy information about clinical studies

All manuscripts should comply with the ICMJE guidelines for publication of clinical research and a completed CONSORT checklist must be included with all submissions.

Clinical trial registration	This trial is registered with ClinicalTrials.gov (NCT04483739).
Study protocol	The Redacted Trial Protocol and Redacted Statistical Analysis Plan (SAP) are included in the Supplementary Information file.
Data collection	The EMN24 IsKia trial is a randomized, open-label, phase III trial that enrolled patients from 42 European centers in 8 countries (a list of study sites that enrolled at least 1 patient is reported in the Supplementary Information file). All patients were enrolled between October 7, 2020 and November 15, 2021. Study recruitment is complete.
Outcomes	The primary study endpoint was the rate of MRD negativity (sensitivity of 10^-5 or better) detected by NGS in the ITT population after post-ASCT full-dose consolidation.<br>Key secondary endpoints were the rates of NGS-MRD negativity after induction (sensitivity of 10^-5 or better) and PFS in the ITT population. Other secondary endpoints included overall response rate, MRD negativity after ASCT, MRD negativity after light consolidation, rate of 1-year sustained MRD negativity, safety, and comparative analyses in patient subgroups defined according to known prognostic factors. In particular, the analyses focused on: high-risk cytogenetics per International Myeloma Working Group (IMWG) criteria [defined as the presence of t(4;14), t(14;16), or del(17p)]; number of HRCA [1 HRCA was defined as the presence of one of the following HRCA: del(17p13.1), t(4;14) (p16.3;q32.3), t(14;16) (q32.3;q23), gain(1q21), or amp(1q21); 2+ HRCA was defined as the presence of at least two HRCA); Revised ISS [R-ISS; R-ISS stage I included ISS stage I (serum β2-microglobulin level <3.5 mg/L and serum albumin level ≥3.5 g/dL), no HRCA [del(17p) and/or t(4;14) and/or t(14;16)], and normal lactate dehydrogenase (LDH) level (less than the upper limit of normal range); R-ISS stage III included ISS stage III (serum β2-microglobulin level >5.5 mg/L) and HRCA or high LDH level; and R-ISS stage II included all the other possible combinations]; and Second Revision of the ISS [R2-ISS; in which a value was assigned to each risk feature according to their impact on overall survival (ISS III 1.5, ISS II 1, del(17p) 1, high LDH 1, t(4;14) 1, and 1q+ 0.5 points), and patients were stratified into four risk groups according to the total additive score: low (R2-ISS-I, 0 points), low-intermediate (II, 0.5–1 points), intermediate-high (III, 1.5–2.5 points), and high (IV, 3–5 points)]. In addition, we aimed to analyze outcomes according to the recent IMS/IMWG Consensus Recommendations. Since data on t(14;20), TP53 mutations, and differentiation between monoallelic vs. biallelic del(1p32) were not available for the current analyses, we defined high risk as per modified IMS/IMWG Consensus Recommendations as the presence of at least one of these abnormalities: (1) del(17p) with a cut-off of >20% clonal fraction; (2) an IgH translocation including t(4;14) or t(14;16) along with 1q+ and/or del(1p32); (3) del(1p32) along with 1q+; (4) β2 microglobulin ≥5.5 mg/L with normal creatinine (<1.2 mg/dL). This analysis was exploratory and not pre-specified in the trial protocol, as this risk stratification was very recently published. A post-hoc analysis, focusing on patients with t(11;14) as subgroup of interest, was also performed. A disaggregated analysis by sex was not pre-specified in the trial Protocol and was not performed because sex is not thought to be a major confounder in the MM field.<br>Additional planned secondary endpoints not reported in this contribution were time to progression, time to next treatment, PFS 2, OS, total duration of MRD negativity assessed by NGS, duration of response, patient-reported outcomes, maintenance details, subsequent lines of therapy, agreement between MRD techniques (see the Redacted Statistical Analysis Plan in the Supplementary

Information file).
All efficacy analyses were based on the ITT principle: all patients eligible to receive treatment and randomly assigned to one of the treatment arms were included.

Plants

Seed stocks	N/A
Novel plant genotypes	N/A
Authentication	N/A
